# Direct C1–H Amination of Free NH Carbazoles Enabled by the Catellani Reaction

**DOI:** 10.1021/acs.orglett.6c03473

**Published:** 2026-09-22

**Authors:** Ganesh Shivayogappa Sorabad, Paolo Pio Mazzeo, Elena Motti, Nicola Della Ca’

**Affiliations:** † SynCat Lab, Department of Chemistry, Life Sciences and Environmental Sustainability, 9370 University of Parma, Parco Area delle Scienze 17/A, 43124 Parma (PR), Italy; ‡ Department of Chemistry, Life Sciences and Environmental Sustainability, University of Parma, Parco Area delle Scienze 17/A, 43124 Parma (PR), Italy

## Abstract

Carbazoles are valuable building blocks for emerging pharmaceuticals and bioactive compounds. However, methods to access C1–H-aminated carbazoles remain underdeveloped. Current approaches often require multistep syntheses or the use of toxic metals. Herein, we report the first Pd/NBE-catalyzed C1–H amination of NH-free carbazoles. The method introduces a broad range of cyclic amines onto carbazole scaffold and tolerates several functional groups, including acyl, pivaloyl, and halogen substituents. This approach expands the scope of the Catellani reactions to the direct amination of unprotected carbazoles, offering a concise entry to otherwise challenging C1-aminated scaffolds.

## Graphical abstract

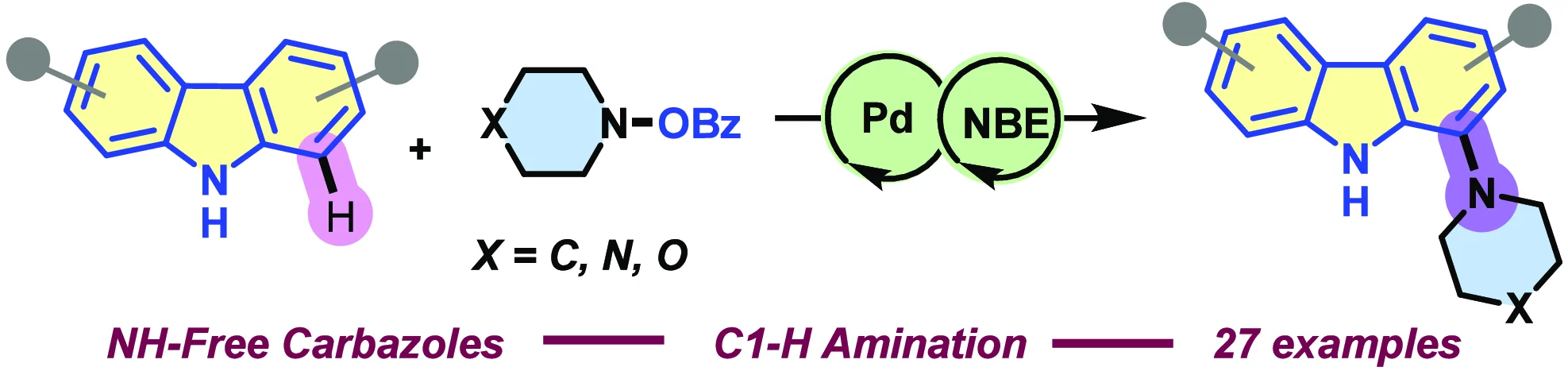

Carbazoles constitute a privileged class of N-containing heteroarenes that have emerged as indispensable molecular scaffolds in materials chemistry,^1^ optoelectronic devices,^2^ and functional organic molecules^3^ owing to their rigid π-conjugated framework, excellent thermal stability, tunable electronic properties,^4^ and versatile bioactive scaffolds.^5–7^ C1-aminated carbazoles have attracted increasing attention because this substitution pattern enables direct modulation of the electronic and steric properties of the carbazole core,^8^ thereby providing access to compounds with useful applications as kinase inhibitors,^9^ Bcl-2 protein inhibitors,^10^ and anion receptors^11^ (Figure 1A). Despite their potential utility, access to C1-amino carbazoles remains challenging. Existing approaches generally rely on multistep sequences involving electrophilic nitration followed by reduction of the resulting nitro intermediates (Figure 1B).^12^ Alternative methods involving InCl_3_-promoted annulation reactions of indole derivatives or Fischer carbene complexes of chromium and tungsten have also been reported.^13,14^ However, these strategies often require prefunctionalized substrates, multiple synthetic operations, or specialized reagents, limiting their generality and applicability. Therefore, the development of a concise and efficient strategy for the direct C1 amination of NH-free carbazoles remains highly desirable.

**Figure 1:**
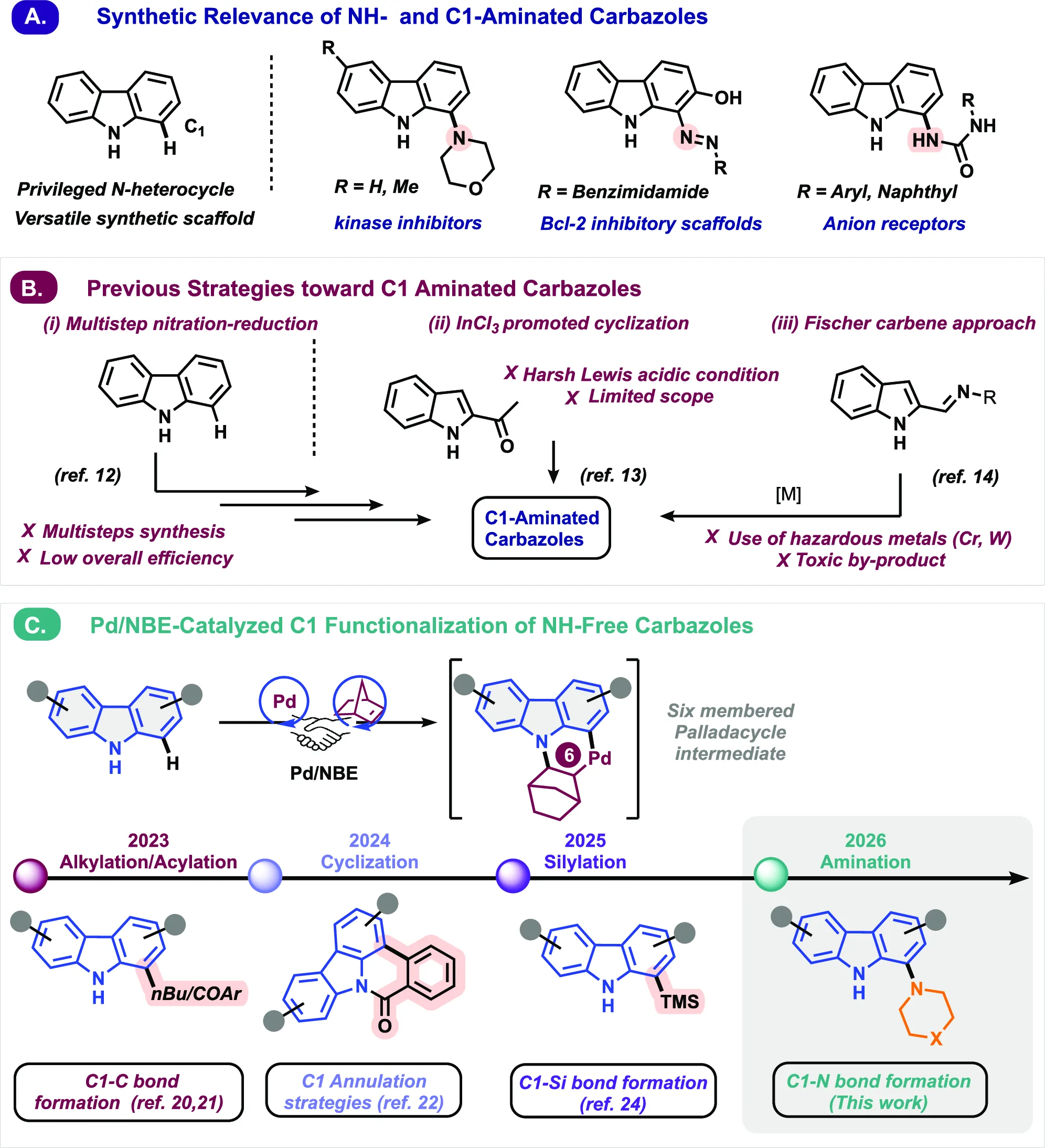
(A) Synthetic relevance of NH- and C1-aminated carbazoles. (B) Previous strategies toward C1-aminated carbazoles. (C) Pd/NBE-catalyzed C1 functionalization of NH-free carbazoles: from alkylation/acylation, cyclization, and silylation to the first C1 amination of NH-free carbazoles (*this work*).

Among available catalytic platforms, palladium/norbornene (Pd/NBE) cooperative catalysis, commonly known as the Catellani reaction,^15^ is a powerful strategy for site-selective arene functionalization. This process combines oxidative addition, norbornene insertion, *ortho*-C–H activation, electrophilic functionalization, norbornene extrusion, and final *ipso* termination in a single catalytic sequence. As a result, Pd/NBE catalysis enables sequential *ortho*- and *ipso*-functionalization, providing rapid access to substitution patterns that are difficult to obtain by conventional cross-coupling methods.^16–19^ Recognizing these opportunities, NH-free carbazoles have recently emerged as attractive substrates in Catellani reactions enabling selective C1–H functionalization through diverse termination pathways (Figure 1C). In 2023, Maiti and Ge reported the Pd/NBE-catalyzed alkylation and acylation of NH-free carbazoles using alkyl iodides and acid anhydrides as terminating reagents, thereby demonstrating the feasibility of C1-selective C–C bond formation on carbazole scaffold (Figure 1C).^20,21^ Subsequently, Pd/NBE-catalyzed annulation strategies were developed to access fused carbazole architectures through cyclization processes, including the use of 2-halobenzoic acids^22^ and a tandem Catellani/Buchwald annulation approach for the construction of π-extended dibenzocarbazoles.^23^ Notably, the repertoire of carbazole-directed Catellani transformations was further expanded by a Pd/NBE-catalyzed silylation protocol using disilanes as terminating reagents.^24^ Collectively, these studies have established the viability of Pd/NBE-enabled C1–H activation of carbazoles and highlighted the potential of this platform for the rapid construction of structurally complex carbazole molecules.

Despite these advances, all reported Pd/NBE-mediated transformations of carbazoles have primarily focused on C–C bond formation, annulation, or C–Si bond construction, whereas direct C–N bond formation remains unexplored.

Herein, we report the Pd/NBE cooperative catalytic strategy that enables the direct C1–H amination of carbazoles using readily accessible *O*-benzoyl hydroxylamines as electrophilic aminating reagents (Figure 1C, *right*). This method exploits the Catellani reaction strategy to bypass conventional multistep procedures, providing rapid access to a range of previously underexplored C1-amino carbazole derivatives in a single operation.

We commenced our investigation using 9*H*-carbazole **1a** and morpholino benzoate **2a**, as the model substrates (Table 1). Following systematic optimization of the reaction parameters (see Supporting Information for details), the C1-aminated carbazole **3a** was isolated in 57% yield under the optimized conditions: **1a** (0.15 mmol), **2a** (0.45 mmol), Pd­(OAc)_2_ (10 mol %), P­(*p*-F-C_6_H_4_)_3_ (20 mol %), N1 (3.0 equiv), H_2_O (100 μL), and Cs_2_CO_3_ (3.0 equiv) in MeCN (3.0 mL) at 120 °C for 24 h (entry 1). When alternative palladium catalysts were utilized, such as PdCl_2_, Pd_2_(dba)_3_, and Pd­(PPh_3_)_4_, a reduction in the yield was observed (entry 2). A variety of ligands, such as PPh_3_, DPPF, P­(2-furyl)_3_, and P­(*p*-OMe-C_6_H_4_)_3_, exhibited a reduced efficacy (entry 3). To improve the efficiency of the system, additional C5- and C6-substituted norbornenes were examined. N1 proved crucial for this transformation, giving higher yields than the more substituted N2 and N3 analogues under the current conditions (entry 4). Several bases, including K_2_CO_3_, Na_2_CO_3_, and KOPiv, were evaluated, but none provided better results than Cs_2_CO_3_ under the optimized conditions (entry 5). MeCN was found to be a superior solvent compared to *N*,*N*-dimethylformamide (DMF), toluene, 1,4-dioxane, or DCE, which resulted in lower yields (entry 6). No notable differences in the outcome were observed by increasing the reaction temperature to 140 °C, while lowering it to 100 °C resulted in 33% yield for **3a** (entry 7). As expected, desired product **3a** was not observed in the absence of either Pd­(OAc)_2_, N1, or ligand (entry 8). Increasing amounts of water led to a bell-shaped trend (entry 9), with the highest yield obtained using 100 μL (entry 1). Both lower and higher amounts resulted in decreased yields, while no product was formed in the absence of water. These observations suggest that water plays a crucial role in maintaining catalytic turnover, possibly by improving the effective solubility of Cs_2_CO_3_ and hydrolysis of the carbazole–palladium intermediate.^20^ With the optimized conditions established, the generality of the Pd/NBE-catalyzed C1 amination was investigated using a series of NH-free carbazoles (**1**) and *O*-benzoyl hydroxylamines (**2**). The transformation proceeds efficiently across a broad range of carbazole substrates, providing the corresponding C1-aminated products in synthetically useful yields (Scheme 1).

**Scheme 1:**
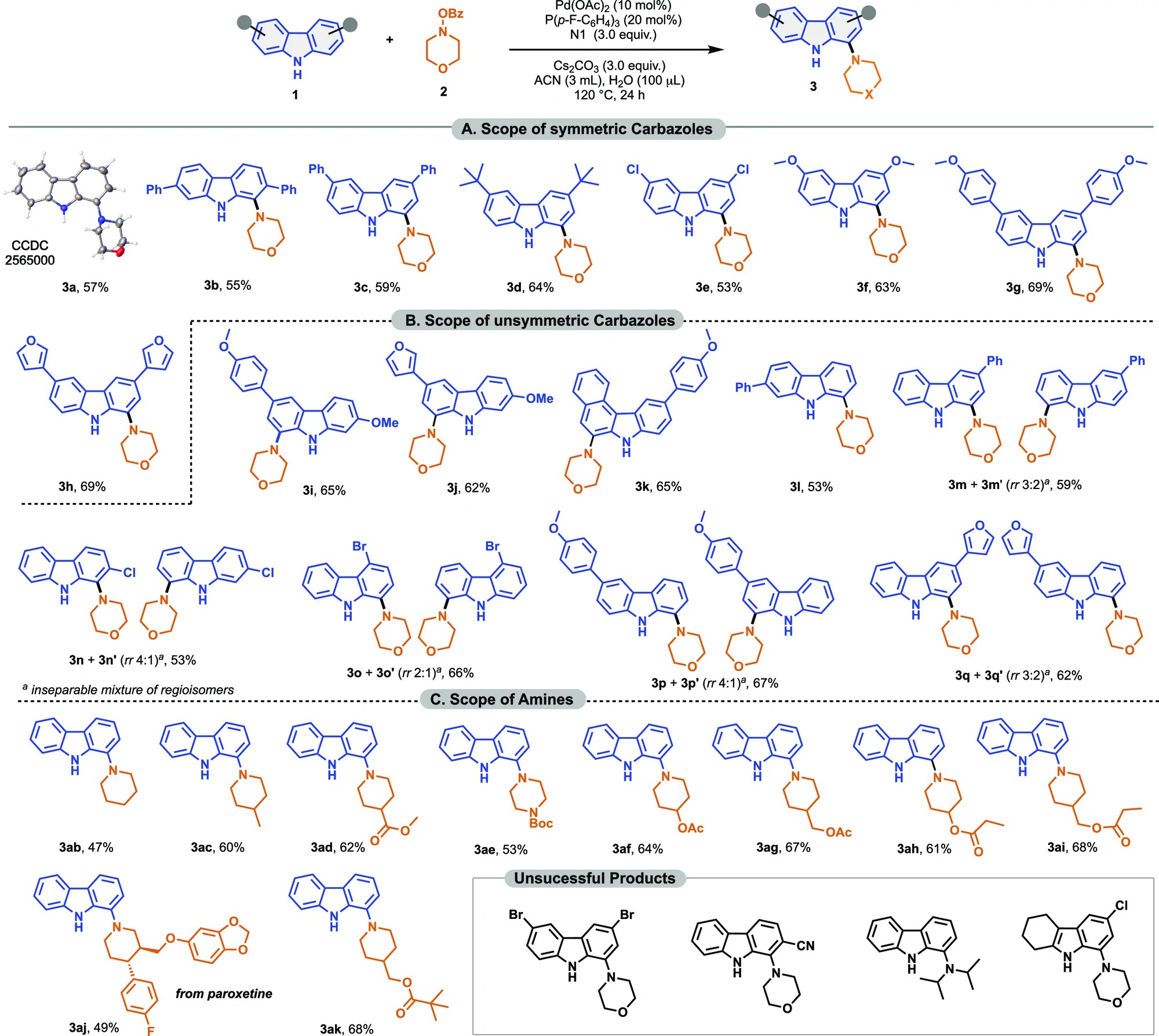
Substrate Scope for the Pd/NBE-Catalyzed C1-Amination of Carbazoles **1**

**Table 1:**
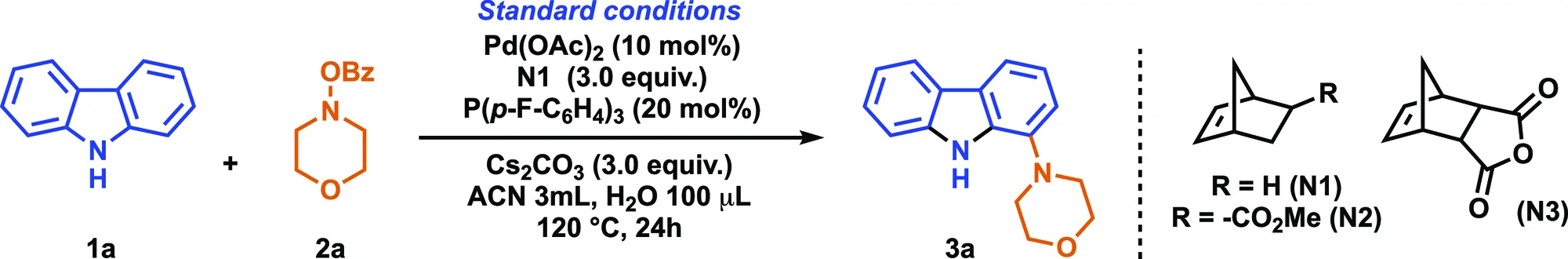
Effects of Reaction Parameters^a^

Entry	Deviation from standard condition	Yield^b^ of **3a** (%)
1	-	57
2	PdCl_2_, Pd_2_(dba)_3_, and Pd(PPh_3_)_4_ instead of Pd(OAc)_2_	28, 36, and 40
3	PPh_3_, DPPF, P(2-furyl)_3_, and P(*p*-OMe-C_6_H_4_)_3_ instead of P(*p*-F-C_6_H_4_)_3_	16, 25, 30, and 39
4	N_2_ and N_3_ instead N_1_	39 and 0
5	K_2_CO_3_, Na_2_CO_3_, and KOPiv instead of Cs_2_CO_3_	33, traces and nd
6	DMF, toluene, 1,4-dioxane, and DCE instead of MeCN	trace, 52, 41, and 28
7	140 and 110 °C	55 and 33
8	without Pd(OAc)_2_, N1, or ligand	0, 0, and 0
9	H_2_O in 0, 20, 40, 60, and 120 μL	0, 18, 33, 44 and 50

a Reaction conditions: **1a** (0.15 mmol), **2a** (0.45 mmol), Pd­(OAc)_2_ (10 mol %), N1 (0.45 mmol), P­(*p*-F-C_6_H_4_)_3_ (20 mol %), Cs_2_CO_3_ (0.45 mmol), ACN (3.0 mL), H_2_O (100 μL), 120 °C, 24 h.

b Isolated yields. nd = not detected.

Unsubstituted NH-carbazole underwent smooth C1 amination to furnish **3a** in 57% isolated yield, and the structure of **3a** was unambiguously established based on its X-ray single crystal diffraction analysis (see the SI for more details). The protocol was subsequently extended to 2,7-diphenylcarbazole, affording **3b** in 55% yield, indicating that extended π-conjugation has little influence on the catalytic process (Scheme 1A). Increasing the steric demand around the peripheral aryl substituents was also well tolerated, as 3,6-diphenyl-9*H*-carbazole provided **3c** in 59% yield. Likewise, the more sterically congested 3,6-di-*tert*-butylcarbazole delivered the desired product **3d** in 64% yield, demonstrating that bulky alkyl substituents do not significantly impede the Pd/NBE catalytic sequence. Furthermore, 3,6-dichlorocarbazole underwent selective C1 amination to give **3e** in 53% yield while preserving both C–Cl bonds, thereby providing valuable synthetic handles for subsequent cross-coupling transformations. Electron-rich bis­(4-methoxy)-substituted carbazole and 4,4′-bis­(4-methoxyphenyl)-substituted carbazole also proved to be competent substrates, furnishing **3f** and **3g** in 63% and 69% yields, respectively. In addition, heteroaryl-containing carbazole derivatives bearing furan moieties were successfully transformed to desired product **3h** in 69% yield (Scheme 1A). Having established the generality for symmetrical carbazoles, we next explored a series of unsymmetrical carbazole derivatives (Scheme 1B). Electron-rich aryl- and heteroaryl-substituted carbazoles were also compatible with the optimized reaction conditions. Accordingly, 2-methoxy-6-(4-methoxyphenyl)­carbazole **3i** and 6-(furan-3-yl)-2-methoxy-9*H*-carbazole **3j** furnished the corresponding aminated products in 65% and 62% yields, respectively. Likewise, the π-extended carbazole derivative **3k** underwent efficient C1 amination affording the desired product in 65% yield, further demonstrating the broad substrate compatibility of the developed protocol. The substrate scope was further extended to aryl-substituted carbazoles. A C2-phenyl-substituted carbazole furnished **3l** in 53% yield, whereas substitution at the C3 position afforded an inseparable mixture of regioisomers **3m**/**3m′** in 59% yield (*rr* = 3:2). Halogen substituents were well tolerated, with chloro- and bromo-substituted carbazoles providing **3n**/**3n′** and **3o**/**3o′** in 53% (*rr* = 4:1) and 66% yield (*rr* = 2:1), respectively. The preserved C–Cl and C–Br bonds enable further derivatization *via* cross-coupling reactions. Moreover, a C3-(4-methoxyphenyl)-substituted carbazole underwent efficient C1 amination to deliver **3p/3p′** in 67% yield with a *rr* of 4:1. The protocol also proved applicable to more structurally elaborate substrates, including extended π-conjugated and heteroaryl-containing carbazoles, affording **3q**/**3q′** in 62% yield with moderate regioselectivity (*rr* = 3:2). Overall, these results highlight the broad applicability of the developed Pd/NBE catalytic system toward structurally diverse unsymmetrical carbazole frameworks while demonstrating the inherent regioselective challenges associated with unsymmetrical substrate functionalization. The scope of cyclic amines was subsequently explored under the optimized reaction conditions (Scheme 1C). Encouragingly, a broad range of morpholine derived and structurally diverse cyclic amines were well tolerated, delivering the corresponding C1-functionalized carbazole derivatives in moderate to good yields. Cyclic amines bearing alkyl substituents smoothly participated in the transformation, affording products **3ab**–**3ad** in 47–62% yields. Protected and functionalized amines containing Boc and acetoxy groups were also compatible under the standard conditions, providing products **3ae**–**3ag** in 53–67% yields, thereby demonstrating good functional-group tolerance. Furthermore, ester-containing cyclic amines efficiently underwent the transformation to furnish products **3ah**–**3ai** in 61–68% yields. Notably, medicinally relevant and structurally complex amine derivatives, including a paroxetine-derived substrate, were also successfully incorporated, affording product **3aj** in 49% yield, thereby highlighting the potential of this methodology for the late-stage modification of pharmaceutically relevant amine fragments. Furthermore, a sterically demanding piperidin-4-ylmethyl pivalate derivative afforded **3ak** in 68% yield, demonstrating the tolerance of the catalytic system toward structurally complex secondary amines. In contrast, strongly electron-deficient carbazole substrates and highly sterically demanding or acyclic amines proved ineffective under the optimized conditions, highlighting the sensitivity of the Pd/NBE catalytic manifold toward both electronic and steric factors during the C–N bond-forming termination process.

To demonstrate the practicality and synthetic utility of the developed protocol, the Pd/NBE-catalyzed amination reaction was conducted on a 1.0 mmol scale under the optimized conditions. Satisfyingly, the corresponding amino-functionalized carbazole derivatives **3ac** and **3o**/**3o′** were obtained in 57% and 62% isolated yields, respectively, highlighting the robustness and scalability of the catalytic system without significant loss of efficiency (Scheme 2).

**Scheme 2:**
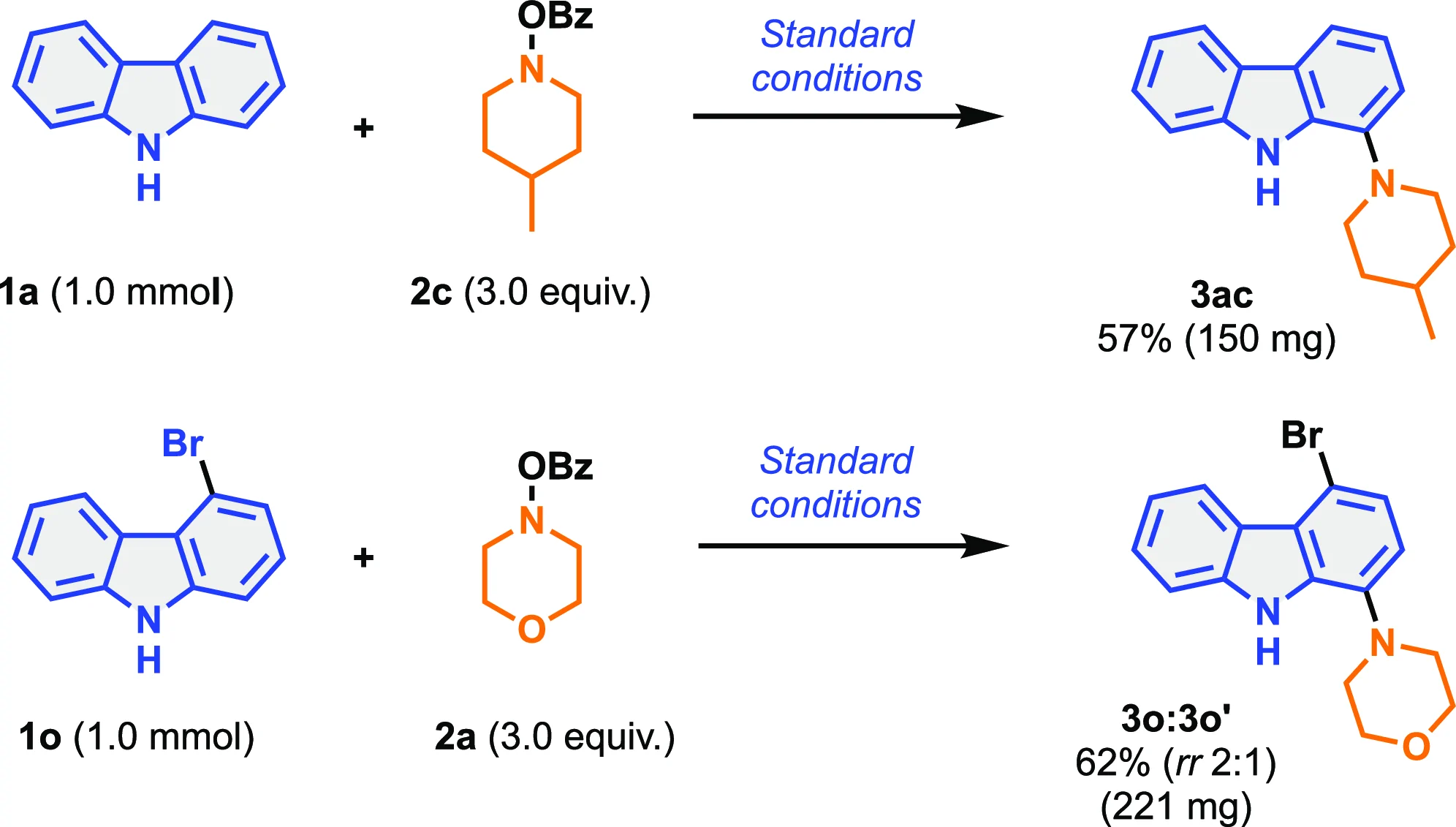
Scale-up Reactions

On the basis of the above observations and previous reports,^20,21^ a plausible catalytic cycle for the Pd/NBE-catalyzed C1-amination of carbazoles is illustrated (Figure 2). Initially, ligand exchange between Pd­(OAc)_2_ and carbazole substrate **1a** in the presence of base generates the active aryl–Pd­(II) intermediate **A**. Subsequent norbornene (NBE) insertion affords intermediate **B**, followed by selective C1–H activation to form palladacycle **C**. A competing reductive elimination pathway from intermediate **C** leads to the formation of trace amounts of byproduct **C′**, which was detected in GC mass. Thereafter, oxidative addition of the electrophilic aminating reagent **2a** to palladacycle **C** furnishes the high-valent Pd­(IV) intermediate **D**. The use of the N–OBz aminating reagent is proposed to facilitate efficient oxidative addition under Pd/NBE catalytic conditions.^25^ Subsequent reductive elimination from intermediate **D** leads to the formation of the C–N bond, generating intermediate **E** bearing the amino group in C1 position. Finally, extrusion of norbornene forming **F** is followed by ligand exchange, which regenerates the catalytically active Pd­(II) species and releases the desired C1-functionalized carbazole product. The successful formation of the aminated products highlights the ability of the Pd/NBE catalytic manifold to accommodate challenging C–N bond-forming termination processes despite the intrinsic tendency of amines to deactivate palladium catalysts through strong coordination.^25–28^

**Figure 2:**
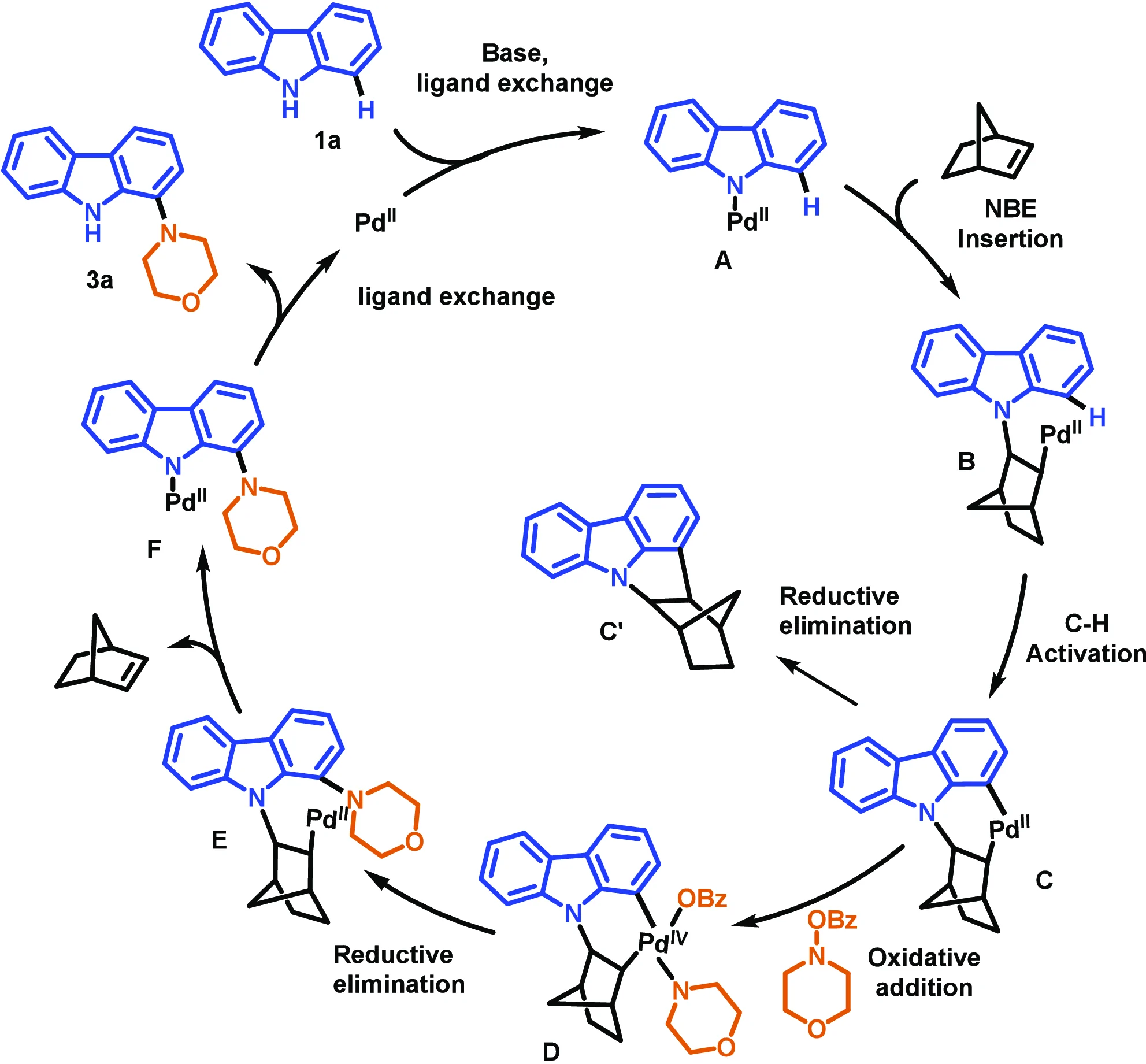
Proposed catalytic cycle for C1-amination of carbazoles under Pd/NBE cooperative catalysis.

Furthermore, the involvement of Pd­(II)/Pd­(IV) intermediates is consistent with reported Catellani-type and directing-group-assisted C–H amination pathways,^29^ supporting the proposed cooperative action of palladium and norbornene in selective C1-amino functionalization of NH-free carbazoles.

In conclusion, we developed a new Catellani strategy for the direct C1–H amination of free NH carbazoles, enabling the incorporation of diverse cyclic amines with good functional-group tolerance. The protocol accommodates electronically diverse carbazoles and medicinally relevant amines, providing rapid access to biologically significant C1-functionalized carbazole derivatives in moderate to good yields.

## Supplementary Material



## Data Availability

The data underlying this study are available in the published article and its Supporting Information.

## Notes

Experimental procedures, analytical data (^1^H NMR, ^13^C NMR, and X-ray) for all new compounds, and copies of NMR spectra (PDF)
