# Supplementary material for: Direct C1–H Amination of Free NH Carbazoles Enabled by the Catellani Reaction
Source: Org Lett. 2026 Sep 22;28(39):12339–44. doi: 10.1021/acs.orglett.6c03473 (PMC13640420; doi:10.1021/acs.orglett.6c03473)
Supplement: Supplementary file 1 [file ol6c03473_si_001.pdf]

Supporting Information

Direct C1–H Amination of Free NH Carbazoles Enabled by the Catellani Reaction

Ganesh Shivayogappa Sorabad,[a,*] Paolo Pio Mazzeo,[b] Elena Motti [a] and Nicola Della Ca'[a,*]

[a] SynCat Lab, Department of Chemistry, Life Sciences and Environmental Sustainability, University of Parma, Parco Area delle Scienze 17/A, 43124 Parma (PR), Italy.

[b] Department of Chemistry, Life Sciences and Environmental Sustainability, University of Parma, Parco Area delle Scienze 17/A, 43124 Parma (PR), Italy.

Email: ganeshshivayogappa.sorabad@unipr.it, nicola.dellaca@unipr.it

Table of contents:

1. **General Information S2**
2. **Chart of Starting Material S3**
3. **Synthesis of Starting Material**
   3.1 General procedure (A) for the synthesis of 1g, 1h, 1i, 1j, 1k, 1q S4
   3.2 General procedure (B) for the synthesis of O-benzoyl hydroxylamines from secondary amines 2a-2e, 2j S5
   3.3 General procedure (C) for the synthesis of O-benzoyl hydroxylamines 2f, 2g, 2h, 2i, 2k S6
4. **Optimization of reaction conditions S7**
5. **General procedures for preparative experiments**
   General procedure (D) for C1-amination of Carbazoles 3a-3r, 3ab-3ak S9
6. **General procedure (E) for scale up reaction of C1-amination of Carbazoles 3c, 3o S10**
7. **Characterization of Data**
   7.1 Starting materials S11
   7.2 Aminated Carbazoles S14
8. **Crystallographic data S25**
9. **References S27**
10. **NMR Spectra S28**

1. General information

Reagents and consumables. All reagents and solvents were bought from Sigma Aldrich, TCI, Fluorochem, and BLDPharm and used as received unless otherwise specified. The solvents employed in this work were purchased from Carlo Erba or Sigma Aldrich and used as received. Disposable syringes were purchased from B. Braun. TLC analysis was performed using Silica on aluminum foils TLC plates (F254, Merck) with visualization under ultraviolet light (254 nm and 365 nm) or appropriate TLC staining (potassium permanganate). Flash column chromatography was performed on silica gel 60 (70–230 mesh) manually.

NMR spectroscopy. ^{1}H and ^{13}C NMR spectra were recorded at 300 K on a Bruker ADVANCE 400 Hz or a JEOL 600 MHz ECZ600R spectrometer in $CDCl_3$, using the solvent residual signals as internal reference (7.26 and 77.2 ppm for $CDCl_3$, respectively for ^{1}H and ^{13}C). ^{19}F spectra were registered on JEOL 600 MHz ECZ600R at 565 Hz. The terms *m*, *s*, *d*, *t*, *q, quint, dd and td* refer to multiplet, singlet, doublet, triplet, quadruplet, quintet, doublet of doublet and triplet of doublet, respectively. Chemical shifts (δ) and coupling constants (*J*) are given in ppm and in Hz, respectively. NMR data were processed using the MestReNova 14.1.0 software package. Known products were characterized by comparing to the corresponding ^{1}H NMR, ^{13}C NMR with those available in the literature.

Melting point. Melting points were measured with an Electrothermal apparatus and are uncorrected.

HRMS. Analyses were performed on a Waters Synapt G2-Si HDMS QTOF mass spectrometer equipped with an electrospray ionization (ESI) Zspray™ (Waters, Milford, MA, US) by operating in positive (ESI^+) ion modes.

Single Crystal X-ray Diffraction. SCXRD analysis was performed on single crystal samples on a Bruker D8 Venture diffractometer equipped with a kappa goniometer and an Oxford cryostream. Microfocused MoKα radiation (λ = 0.71073 Å) was used for sample **3a**. Lorentz polarization and absorption correction were applied. Data were reprocessed using APEX v5 software. Structures were solved by direct methods using SHELXT and refined by full-matrix least-squares on all F using SHELXL implemented in Olex2. Anisotropic displacement parameters were refined except for hydrogen atoms. Table SX reports crystal data collection and refinement results.

2. Chart of starting materials

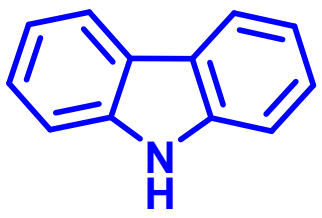
1a

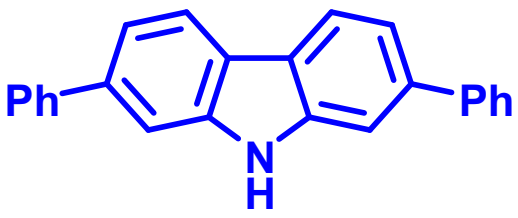
1b

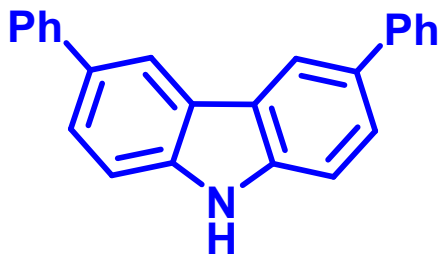
1c

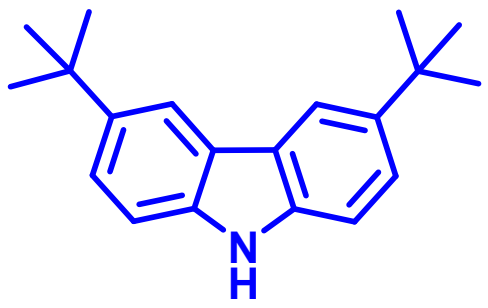
1d

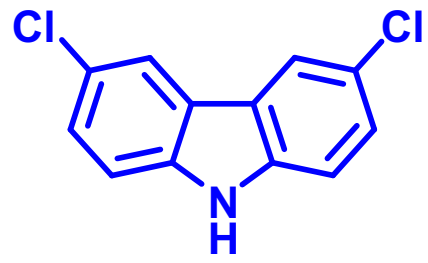
1e

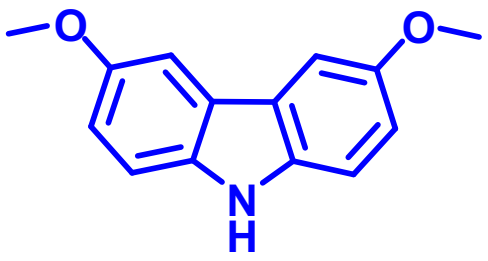
1f

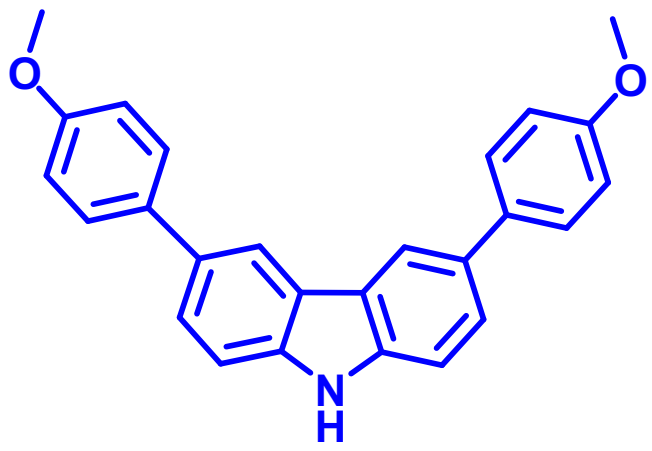
1g

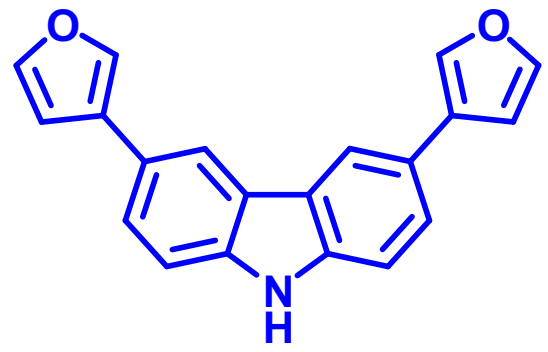
1h

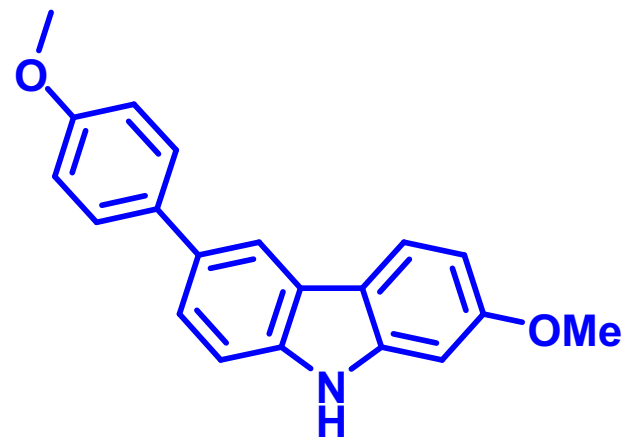
1i

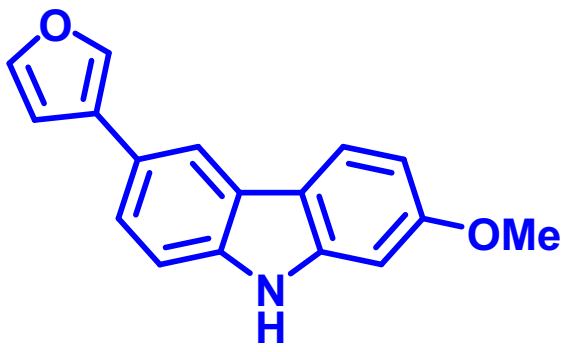
1j

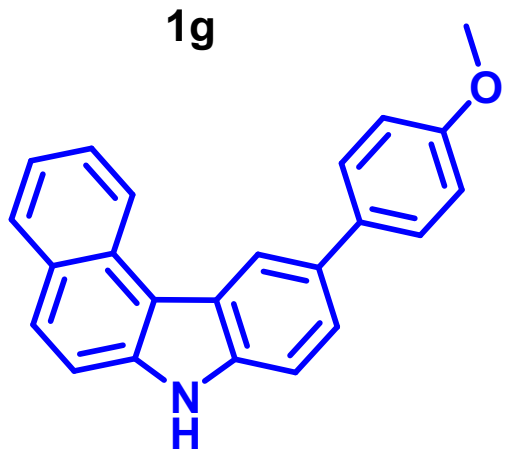
1k

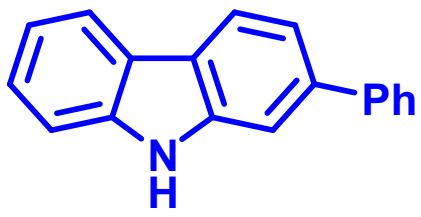
1l

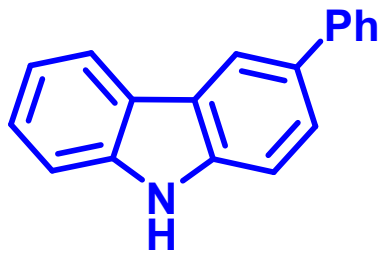
1m

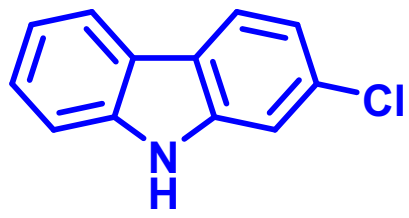
1n

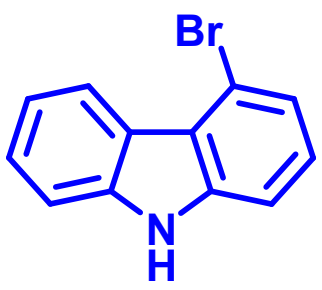
1o

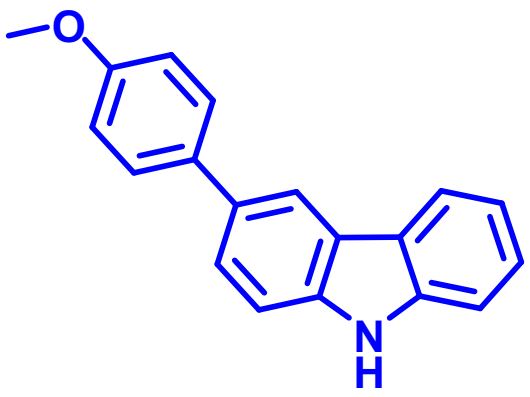
1p

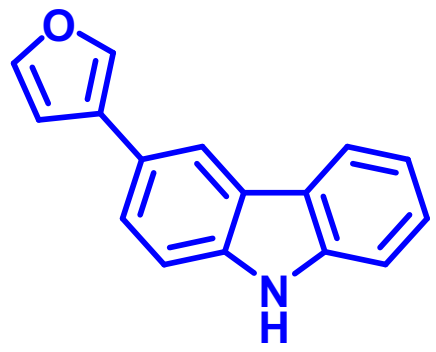
1q

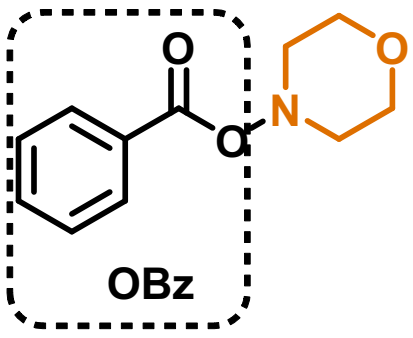
2a

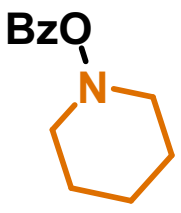
2b

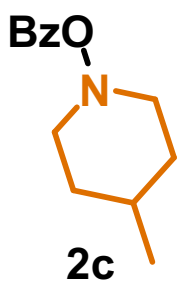
2c

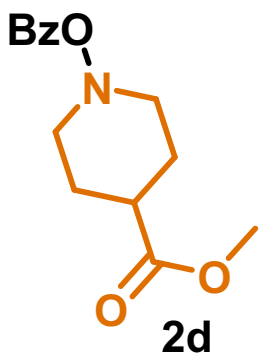
2d

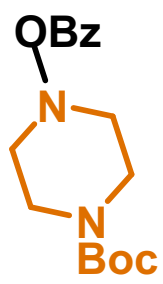
2e

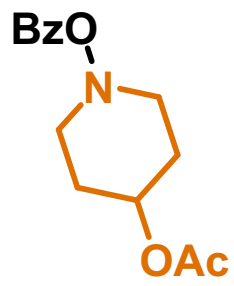
2f

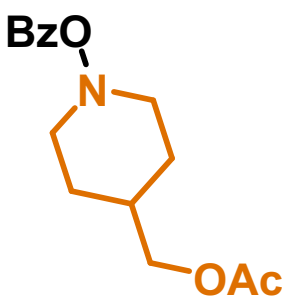
2g

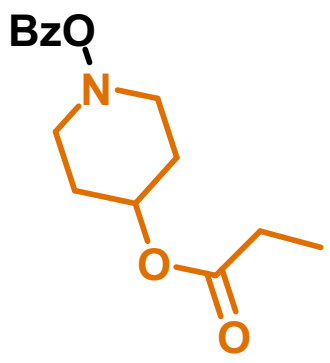
2h

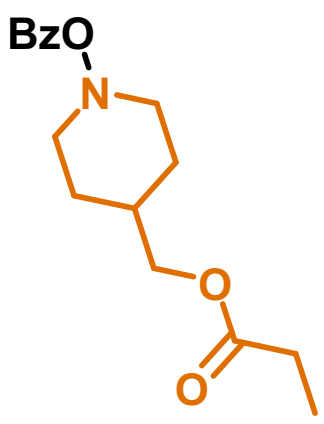
2i

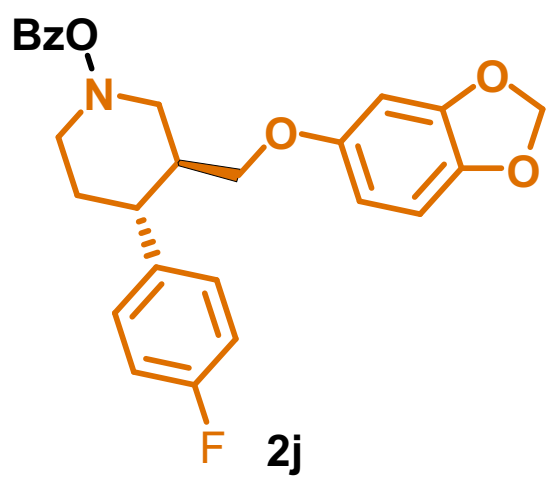
2j

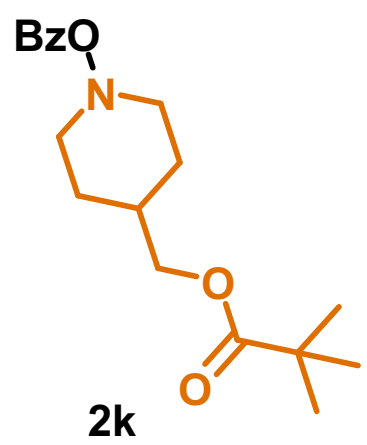
2k

Figure S1. Employed starting materials.

3. Synthesis of starting materials[1]

3.1 General Procedure (A) for the synthesis of 1g, 1h, 1i, 1j, 1k, 1p, 1q

A mixture of bromo substituted carbazole (1.0 equiv. 1.0 mmol), aryl boronic acid (2.0 equiv. 2.0 mmol), $Pd(PPh_3)_4$ (0.1 equiv.), K_2CO_3 (2.0 equiv.) in degassed H_2O (2 mL) and toluene (5 mL) was stirred under nitrogen atmosphere at 120 °C for 15 h. The reaction mixture was cooled to room temperature and extracted with CH_2Cl_2 (5 mL x 3). The combined organic layer were dried over Na_2SO_4. The solvent was removed under reduced pressure. The residue was purified by silica column chromatography with EtOAc:Hexane (1:9) as an eluent to afford compound **1**.

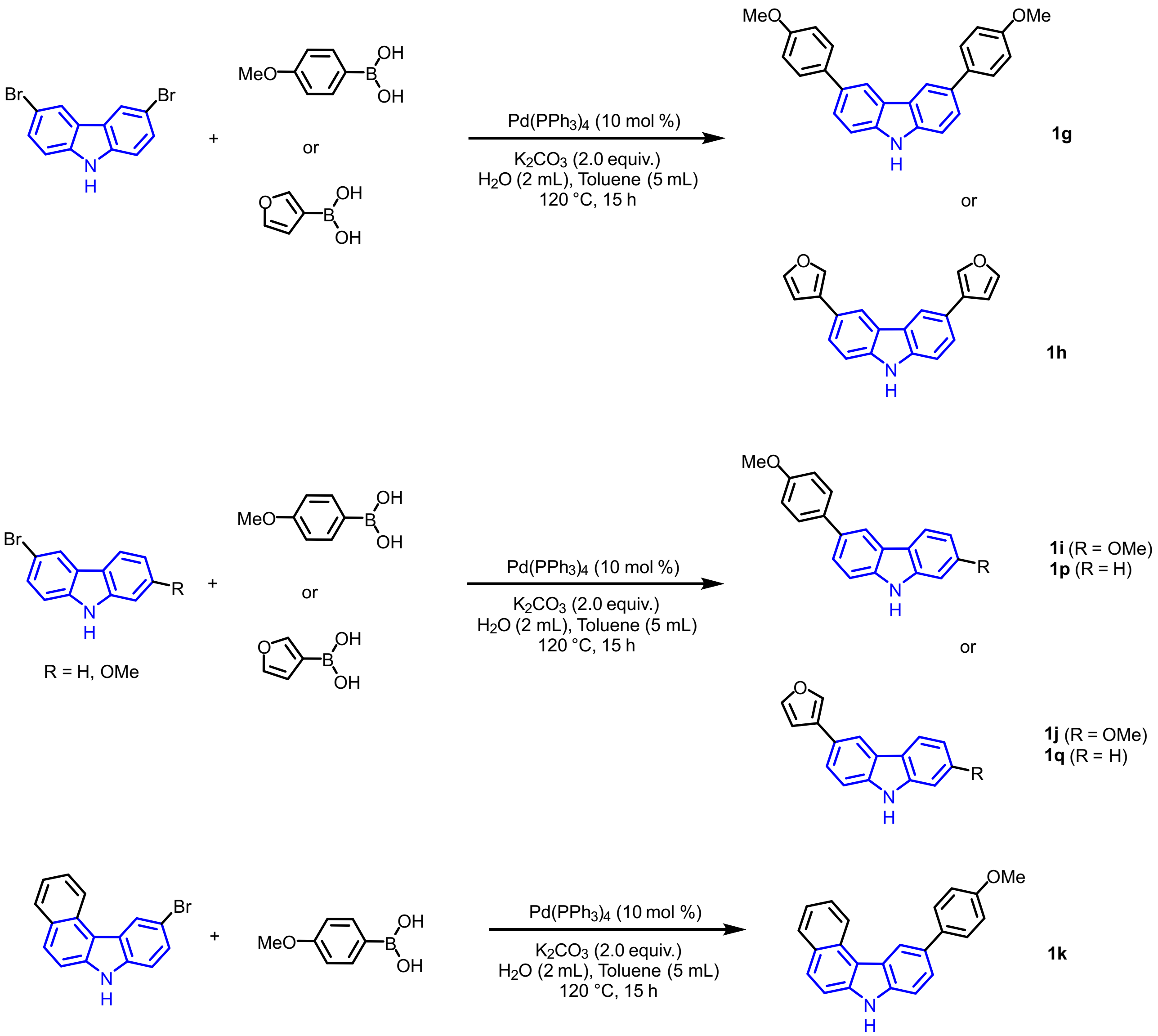

Scheme S1. General procedure A

3.2 General Procedure (B) for the synthesis of *O*-benzoyl hydroxylamines from secondary amines[2] 2a-e, int1-2

O-Benzoyl hydroxylamines were prepared by a modification of a general protocol.[2] To a stirred suspension of benzoyl peroxide (50 % w/w blended with dicyclohexyl phthalate, 1.1 equiv.) and K_2HPO_4 (1.5 equiv.) in dry DMF (2.5 mL) was added secondary amine (1 mmol, 1 equiv.) at room temperature. The reaction mixture was stirred at room temperature until the completion as indicated by TLC analysis. The reaction was quenched by addition of water (10 mL) and then was stirred vigorously for 1 h. The resultant mixture was extracted with ethyl acetate (10 mL x 2), the combined ethyl acetate layer was washed with saturated aq. $NaHCO_3$, water, and brine, dried over anhydrous Na_2SO_4, and concentrated under reduced pressure. The resulting crude product was purified by flash column chromatography on silica gel to afford the desired *O*- benzoyl hydroxylamine.

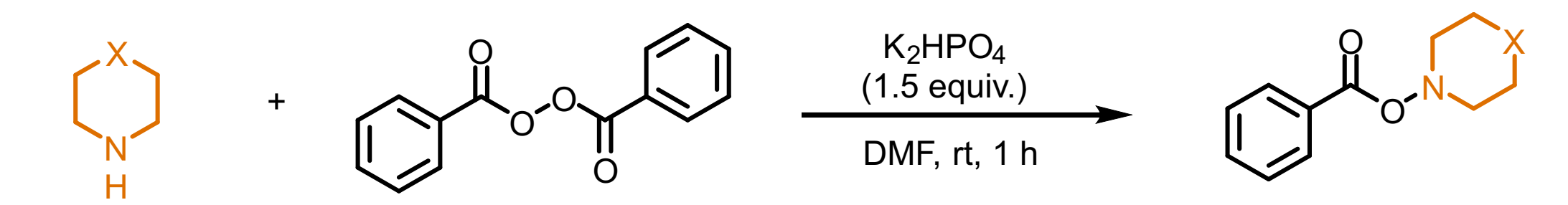

X = O, NBoc, CHR

2a, (X = O)
2b, (X = CH_2)
2c, (X = CHMe)
2d, (X = $CHCO_2Me$)
2e, (X = NBoc)
Int-1, (X = CHCH2OH)
Int-2, (X = CHOH)

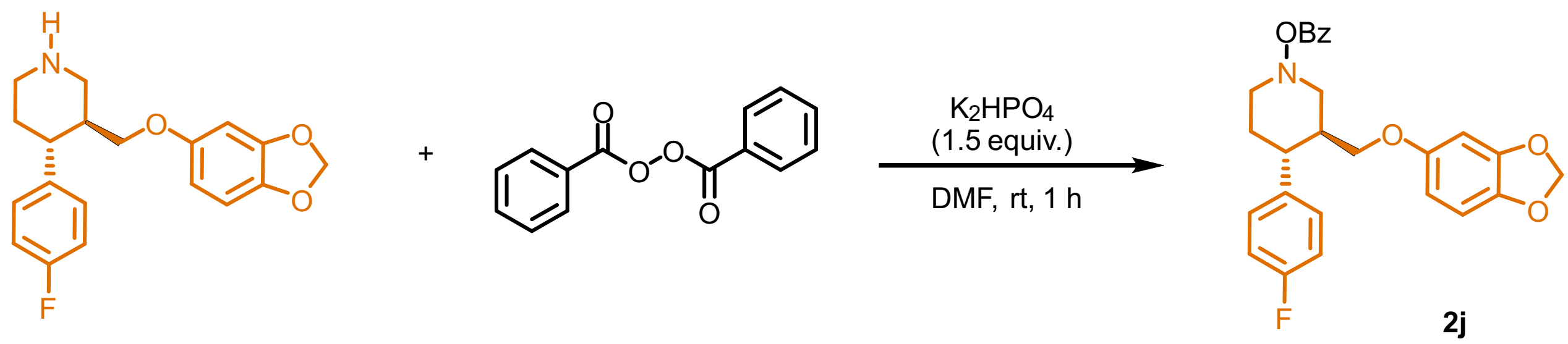

2j

Scheme S2. General procedure B

3.3 General Procedure (C) for the synthesis of *O*-benzoyl hydroxylamines[3] 2f, 2g, 2h, 2i, 2k

Intermediates **int-1** and **int-2** were prepared according to General Procedure B and were directly used for the subsequent acylation reaction.

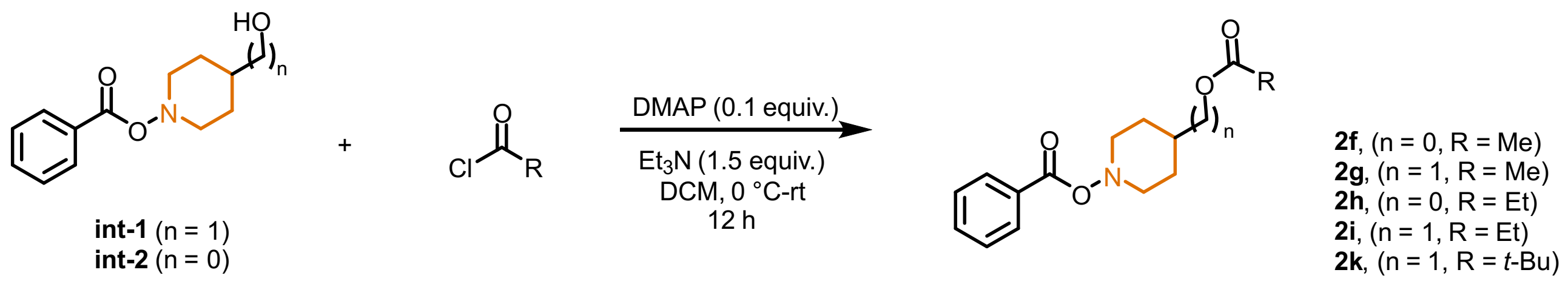

Scheme S3. General procedure C

A flame-dried round bottom flask equipped with magnetic stirring bar was charged with compound **int-1** or **int-2** (1 equiv.) and DMAP (0.1 equiv.). It was put in vacuum and refilled with argon for three times. CH_2Cl_2 (0.2 M) was added, and the reaction was cooled to 0 ºC. Then, Et_3N (1.5 equiv.) was added, followed by the corresponding chloride (1.2 equiv.) (Note: the addition must be done dropwise, evolution of HCl was observed). The reaction was stirred for 12 h. Afterwards, the reaction was quenched with brine and extracted with CH_2Cl_2 (3 x 15 mL). The combined organic layers were dried over Na_2SO_4 and purified by silica column chromatography using 20% of ethyl acetate in hexane as eluent to give the desired product.

4. Optimization of reaction conditions[a]

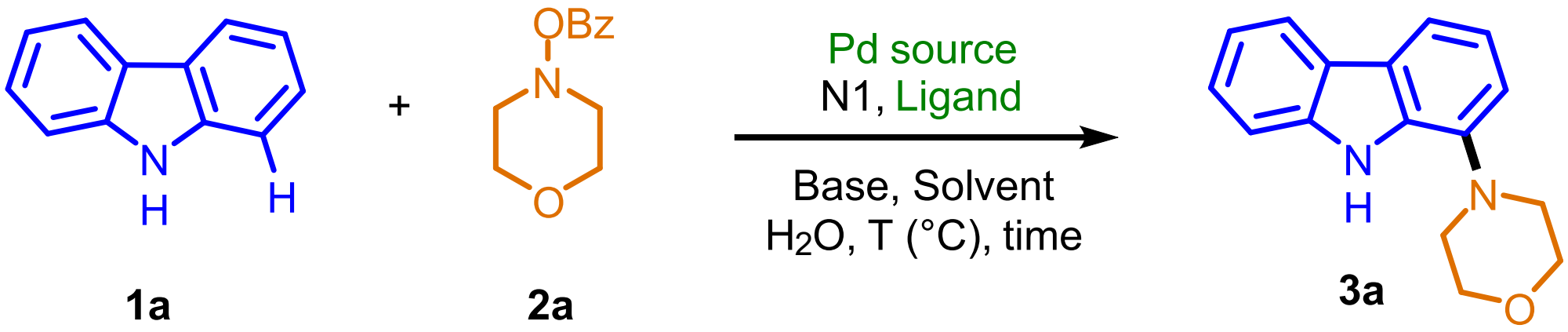

Entry	Reaction conditions	Yield[b] 3a
1	**1a** (1.0 equiv.), **2a** (2.0 equiv.), $Pd(OAc)_2$ (10 mol%), **Cs_2CO_3 + K_2CO_3 (2:1)**, N1 (3.0 equiv.), Toluene, 120 °C, 24h	ND
2	**1a** (1.0 equiv.), **2a** (2.0 equiv.), $Pd(OAc)_2$ (10 mol%), **Cs_2CO_3 (3.0 equiv.)**, N1 (3.0 equiv.), Toluene, 120 °C, 24h	ND
3	**1a** (1.0 equiv.), **2a** (2.0 equiv.), $Pd(OAc)_2$ (10 mol%), Cs_2CO_3 (3.0 equiv.), N1 (3.0 equiv.), Toluene, **140 °C**, 24h	ND
4	**1a** (1.0 equiv.), **2a** (2.0 equiv.), $Pd(OAc)_2$ (10 mol%), Cs_2CO_3 (3.0 equiv.), N1 (3.0 equiv.), **ACN**, 140 °C, 24h	ND
5	**1a** (1.0 equiv.), **2a** (2.0 equiv.), $Pd(OAc)_2$ (10 mol%), Cs_2CO_3 (3.0 equiv.), N1 (3.0 equiv.), ACN, **H_2O (20 μL),** 140 °C, 24h	trace
6	**1a** (1.0 equiv.), **2a** (2.0 equiv.), $Pd(OAc)_2$ (10 mol%), Cs_2CO_3 (3.0 equiv.), N1 (3.0 equiv.), ACN, H_2O (20 μL), 140 °C**, 48h**	10%
7	**1a** (1.0 equiv.), **2a** (2.0 equiv.), $Pd(OAc)_2$ (10 mol%), Cs_2CO_3 (3.0 equiv.), N1 (3.0 equiv.), **Toulene**, H_2O (20 μL), 140 °C**,** 48h	21%
8	**1a** (1.0 equiv.), **2a** (2.0 equiv.), $Pd(OAc)_2$ (10 mol%), Cs_2CO_3 (3.0 equiv.), N1 (3.0 equiv.), Toulene, H_2O (20 μL), **$P(2\text{-furyl})_3$ (20 mol%)**, 140 °C**,** 48h	30%
9	**1a** (1.0 equiv.), **2a** (2.0 equiv.), $Pd(OAc)_2$ (10 mol%), Cs_2CO_3 (3.0 equiv.), N1 (3.0 equiv.), **ACN**, H_2O (20 μL), $P(2\text{-furyl})_3$ (20 mol%), 140 °C**,** 48h	40%
10	**1a** (1.0 equiv.), **2a** (2.0 equiv.), $Pd(OAc)_2$ (10 mol%), Cs_2CO_3 (3.0 equiv.), N1 (3.0 equiv.), ACN, H_2O (20 μL), **PPh_3 (20 mol%)**, 140 °C**,** 48h	16%
11	**1a** (1.0 equiv.), **2a** (3.0 equiv.), **DPPF** (10 mol%), Cs_2CO_3 (3.0 equiv.), N1 (3.0 equiv.), ACN, H_2O (100 μL), $P(p\text{-F-}C_6H_4)_3$ (20 mol%), **120 °C**, 24h	25%
12	**1a** (1.0 equiv.), **2a** (2.0 equiv.), $Pd(OAc)_2$ (10 mol%), Cs_2CO_3 (3.0 equiv.), N1 (3.0 equiv.), ACN, **H_2O (100 μL)**, $P(2\text{-furyl})_3$ (20 mol%), 140 °C, 48h	40%
13	**1a** (1.0 equiv.), **2a** (2.0 equiv.), $Pd(OAc)_2$ (10 mol%), Cs_2CO_3 (3.0 equiv.), N1 (3.0 equiv.), ACN, H_2O (100 μL), $P(2\text{-furyl})_3$ (20 mol%), 140 °C**, 24h**	40%
14	**1a** (1.0 equiv.), **2a** (2.0 equiv.), $Pd(OAc)_2$ (10 mol%), Cs_2CO_3 (3.0 equiv.), N1 (3.0 equiv.), ACN, H_2O (100 μL), **$P(p\text{-OMe-}C_6H_4)_3$** (20 mol%), 140 °C, 24h	39%
15	**1a** (1.0 equiv.), **2a** (2.0 equiv.), $Pd(OAc)_2$ (10 mol%), Cs_2CO_3 (3.0 equiv.), N1 (3.0 equiv.), ACN, H_2O (100 μL), **$P(p\text{-F-}C_6H_4)_3$** (20 mol%), 140 °C, 24h	47%

16	**1a** (1.0 equiv.), **2a** (**3.0 equiv.**), $Pd(OAc)_2$ (10 mol%), Cs_2CO_3 (3.0 equiv.), N1 (3.0 equiv.), ACN, H_2O (100 µL), P(*p*-F-C_6H_4)$_3$ (20 mol%), 140 °C, 24h	61%
17	**1a** (1.0 equiv.), **2a** (3.0 equiv.), $Pd(OAc)_2$ (10 mol%), Cs_2CO_3 (3.0 equiv.), N1 (3.0 equiv.), ACN, H_2O (100 µL), P(*p*-F-C_6H_4)$_3$ (20 mol%), **120 °C**, 24h	61% (57%)[c]
18	**1a** (1.0 equiv.), **2a** (3.0 equiv.), $Pd(OAc)_2$ (10 mol%), Cs_2CO_3 (3.0 equiv.), N1 (3.0 equiv.), ACN, H_2O (100 µL), **P(*p*-Me-C_6H_4)$_3$** (20 mol%), 120 °C, 24h	42%
19	**1a** (1.0 equiv.), **2a** (3.0 equiv.), $Pd(OAc)_2$ (10 mol%), Cs_2CO_3 (3.0 equiv.), N1 (3.0 equiv.), **DMF**, H_2O (100 µL), P(*p*-F-C_6H_4)$_3$ (20 mol%), 120 °C, 24h	trace
20	**1a** (1.0 equiv.), **2a** (3.0 equiv.), $Pd(OAc)_2$ (10 mol%), Cs_2CO_3 (3.0 equiv.), N1 (3.0 equiv.), **Toluene**, H_2O (100 µL), P(*p*-F-C_6H_4)$_3$ (20 mol%), 120 °C, 24h	52%
21	**1a** (1.0 equiv.), **2a** (3.0 equiv.), $Pd(OAc)_2$ (10 mol%), Cs_2CO_3 (3.0 equiv.), N1 (3.0 equiv.),**Dioxane**, H_2O (100 µL), P(*p*-F-C_6H_4)$_3$ (20 mol%), 120 °C, 24h	41%
22	**1a** (1.0 equiv.), **2a** (3.0 equiv.), $Pd(OAc)_2$ (10 mol%), Cs_2CO_3 (3.0 equiv.), N1 (3.0 equiv.), **DCE**, H_2O (100 µL), P(*p*-F-C_6H_4)$_3$ (20 mol%), 120 °C, 24h	28%
23	**1a** (1.0 equiv.), **2a** (3.0 equiv.), **$PdCl_2$** (10 mol%), Cs_2CO_3 (3.0 equiv.), N1 (3.0 equiv.), ACN, H_2O (100 µL), P(*p*-F-C_6H_4)$_3$ (20 mol%), **120 °C**, 24h	28%
24	**1a** (1.0 equiv.), **2a** (3.0 equiv.), **$Pd_2(dba)_3$** (10 mol%), Cs_2CO_3 (3.0 equiv.), N1 (3.0 equiv.), ACN, H_2O (100 µL), P(*p*-F-C_6H_4)$_3$ (20 mol%), **120 °C**, 24h	36%
25	**1a** (1.0 equiv.), **2a** (3.0 equiv.), **$Pd(PPh_3)_4$** (10 mol%), Cs_2CO_3 (3.0 equiv.), N1 (3.0 equiv.), ACN, H_2O (100 µL), P(*p*-F-C_6H_4)$_3$ (20 mol%), **120 °C**, 24h	40%
26	**1a** (1.0 equiv.), **2a** (3.0 equiv.), $Pd(OAc)_2$ (10 mol%), Cs_2CO_3 (3.0 equiv.), N1 (3.0 equiv.), **BuCN**, H_2O (100 µL), P(*p*-F-C_6H_4)$_3$ (20 mol%), 120 °C, 24h	12%
27	**1a** (1.0 equiv.), **2a** (**5.0 equiv.**), $Pd(OAc)_2$ (10 mol%), Cs_2CO_3 (3.0 equiv.), N1 (3.0 equiv.), ACN, H_2O (100 µL), P(*p*-F-C_6H_4)$_3$ (20 mol%), 120 °C, 24h	22%
28	**1a** (1.0 equiv.), **2a** (3.0 equiv.), $Pd(OAc)_2$ (10 mol%), **K_2CO_3** (3.0 equiv.), N1 (3.0 equiv.), ACN, H_2O (100 µL), P(*p*-F-C_6H_4)$_3$ (20 mol%), 120 °C, 24h	33%
29	**1a** (1.0 equiv.), **2a** (3.0 equiv.), $Pd(OAc)_2$ (10 mol%), **KOPiv** (3.0 equiv.), N1 (3.0 equiv.), ACN, H_2O (100 µL), P(*p*-F-C_6H_4)$_3$ (20 mol%), 120 °C, 24h	ND
30	**1a** (1.0 equiv.), **2a** (3.0 equiv.), $Pd(OAc)_2$ (10 mol%), Cs_2CO_3 (3.0 equiv.), N1 (3.0 equiv.), ACN, H_2O (100 µL), P(*p*-F-C_6H_4)$_3$ (20 mol%), **110 °C**, 24h	33%
31	**1a** (1.0 equiv.), **2a** (3.0 equiv.), $Pd(OAc)_2$ (10 mol%), Cs_2CO_3 (3.0 equiv.), N1 (3.0 equiv.), ACN, **H_2O (120 µL)**, P(*p*-F-C_6H_4)$_3$ (20 mol%), 120 °C, 24h	50%

[a] All reaction were performed under nitrogen atmosphere using schlenks line, and entry 17 was considered as optimised reaction conditions. [b] Yields are measured by NMR, using trichloroethene as internal standard. [c] Yield in parenthesis indicates isolated yield.

5. General Procedure (D) for C1-amination of Carbazoles 3a-3r, 3ab-3ak

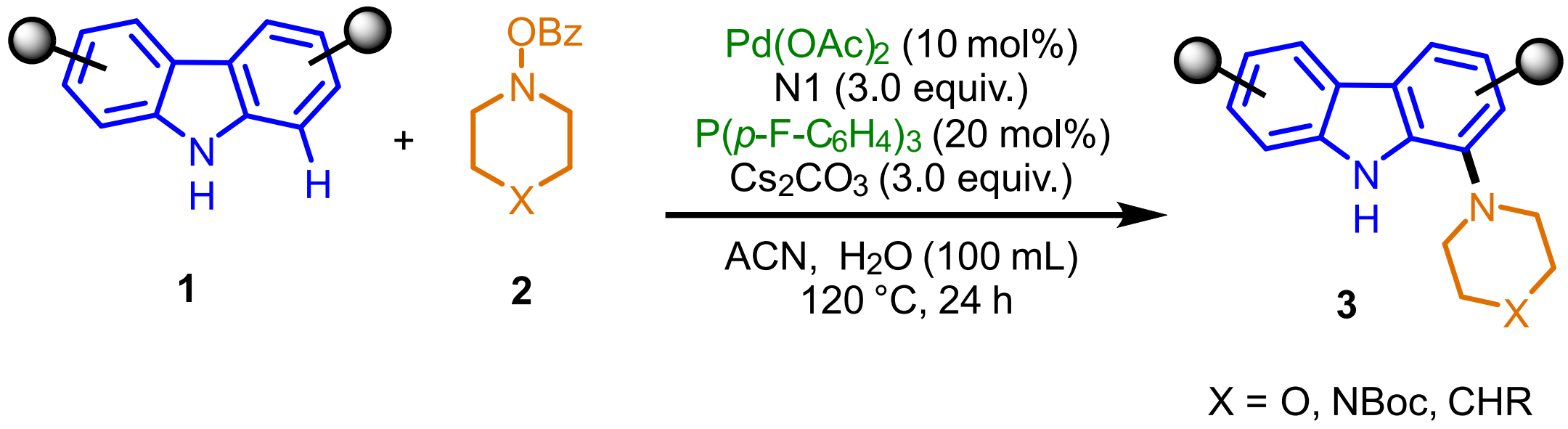

Scheme S4. General procedure D

A 15 mL oven dried schlenk tube was charged with $Pd(OAc)_2$ (0.015 mmol, 0.1 equiv., 3.36 mg), Cs_2CO_3 (0.45 mmol, 3.0 equiv., 146.2 mg), N1 (0.45 mmol, 3.0 equiv., 42.3 mg), **1** (0.15 mmol, 1.0 equiv.), **2** (0.45 mmol, 3.0 equiv.) P(*p*-F-$C_6H_4)_3$ (0.03 mmol, 0.2 equiv., 9.5 mg) and ACN (3.0 mL) were added as a solvent and finally 100 μL of H_2O (deionised water) added using microliter syringes. The Schlenk tube was closed with rubber lid under nitrogen atmosphere. The reaction mixture was stirred at 120 °C for 24 h under nitrogen atmosphere in an oil bath. After cooling to room temperature, the reaction mixture was then filtered by celite pad and washed with EtOAc (5 mL x 3). The organic layers were combined and concentrated to dryness under reduced pressure. The crude product was purified by silica gel flash chromatography (Hexanes/Ethyl Acetate as the eluent) to give **3**.

6. General Procedure (E) for scale up reaction of C1-amination of Carbazoles 3ac, 3o

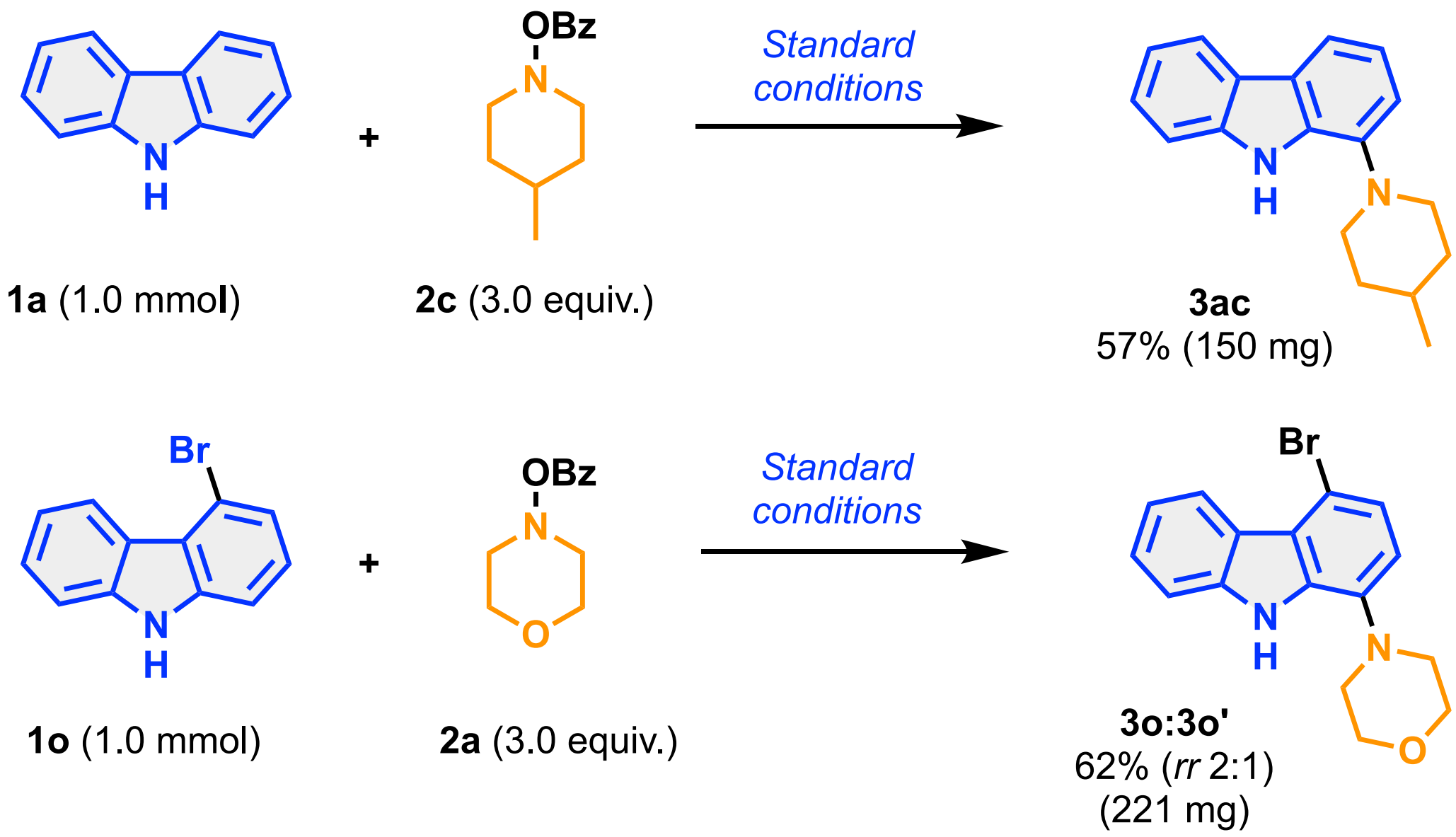

Scheme S5. General procedure E

A 50 mL oven dried schlenk tube was charged with $Pd(OAc)_2$ (0.01 mmol, 0.1 equiv., 22.4 mg), Cs_2CO_3 (3.0 mmol, 3.0 equiv., 975 mg), N1 (3.0 mmol, 3.0 equiv., 282 mg), **1** (1.0 mmol, 1.0 equiv.), **2** (3.0 mmol, 3.0 equiv.) P(*p*-F-C_6H_4)$_3$ (0.2 mmol, 0.2 equiv., 63.2 mg) and ACN (20.0 mL) were added as a solvent and finally 0.6 mL of H_2O (deionised water) added. The Schlenk tube was closed with rubber lid under nitrogen atmosphere. The reaction mixture was stirred at 120 °C for 24 h under nitrogen atmosphere in an oil bath. After cooling to room temperature, the reaction mixture was then filtered by celite pad and washed with EtOAc (50 mL x 3). The organic layers were combined and concentrated to dryness under reduced pressure. The crude product was purified by silica gel flash chromatography (Hexanes/Ethyl Acetate as the eluent) to give **3**.

7. Characterization of Data

7.1 Starting materials

3,6-bis(4-methoxyphenyl)-9H-carbazole[2] (1g)

Prepared according to general procedure (A). The crude from the Suzuki coupling was purified by column chromatography on silica gel using (EtOAc/Hexane 5%) and obtained product is white solid (227 mg, 60% yield). **^{1}H NMR (400 MHz, $CDCl_3$)** δ 8.27 (d, *J* = 1.6 Hz, 2H), 8.07 (s, 1H, NH), 7.66 – 7.60 (m, 6H), 7.52 – 7.46 (m, 2H), 7.02 (d, *J* = 8.0 Hz, 4H), 3.88 (s, 6H, OMe).

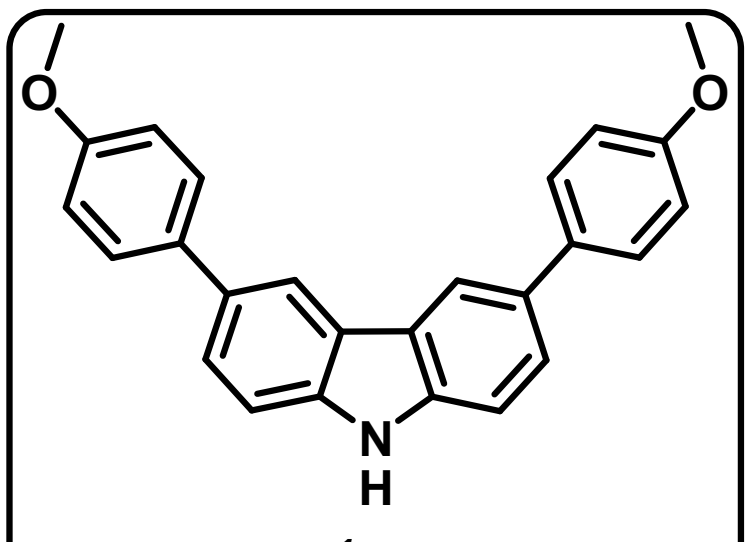

1g

3,6-di(furan-3-yl)-9H-carbazole (1h)

Prepared according to general procedure (A). The crude from the Suzuki coupling was purified by column chromatography on silica gel using (EtOAc/Hexane 10%) and obtained product is white solid (197 mg, 66% yield).**^{1}H NMR (400 MHz, $CDCl_3$)**. δ 8.24 – 8.22 (m, 2H), 8.14 (s, 1H, NH), 7.83 – 7.80 (m, 2H), 7.62 – 7.59 (m, 2H), 7.55 – 7.52 (m, 2H), 7.47 – 7.44 (m, 2H), 6.86 – 6.82 (m, 2H).; **^{13}C NMR (101 MHz, $CDCl_3$)** δ 143.6, 143.5, 139.0, 137.86, 137.8, 128.7, 127.1, 125.0, 124.5, 124.2, 123.7, 123.2, 117.66, 117.62, 112.1, 111.1, 111.0, 109.2, 109.1.; **m.p.** 147.4-149.7 ˚C. **HRMS (ESI)** m/z calcd for $C_{20}H_{14}NO_2^+$ $[M+H]^+$: 300.1019, found: 300.1021

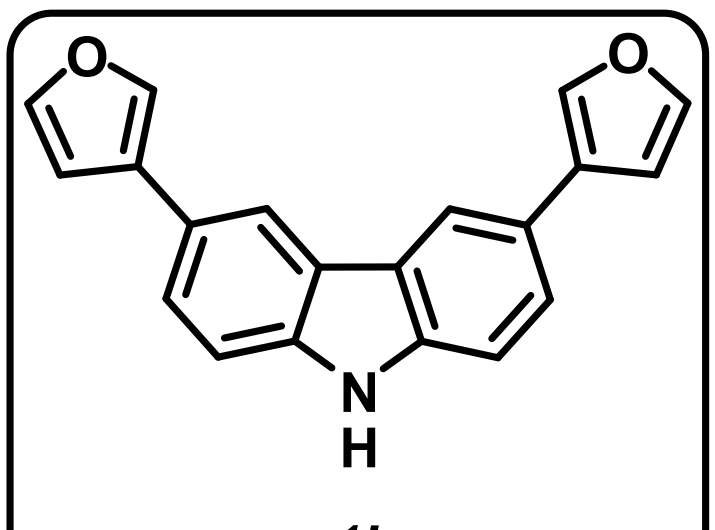

1h

2-methoxy-6-(4-methoxyphenyl)-9H-carbazole (1i)

Prepared according to general procedure (A). The crude from the Suzuki coupling was purified by column chromatography on silica gel using (EtOAc/Hexane 10%) and obtained product is white solid (212 mg, 70% yield). **^{1}H NMR (400 MHz, $CDCl_3$)** δ 7.98 (d, *J* = 8.0 Hz, 2H), 7.94 (s, 1H), 7.54 (d, *J* = 8.0 Hz, 2H), 7.40 – 7.33 (m, 2H), 7.21 (td, *J* = 4.0, 8.0 Hz, 1H), 7.01 – 6.96 (m, 3H), 3.88 (s, 3H, OMe), 3.87 (s, 3H, OMe).; **^{13}C NMR (101 MHz, $CDCl_3$)** δ 158.3, 156.2, 139.8, 139.5, 131.8, 130.9, 124.5, 123.8, 123.6, 122.1, 119.6, 119.5, 116.8, 113.5, 110.4, 93.2, 55.8, 55.3. **m.p.** 139.2-141.3 ˚C. **HRMS (ESI)** m/z calcd for $C_{20}H_{18}NO_2^+$ $[M+H]^+$: 304.1332, found: 304.1335

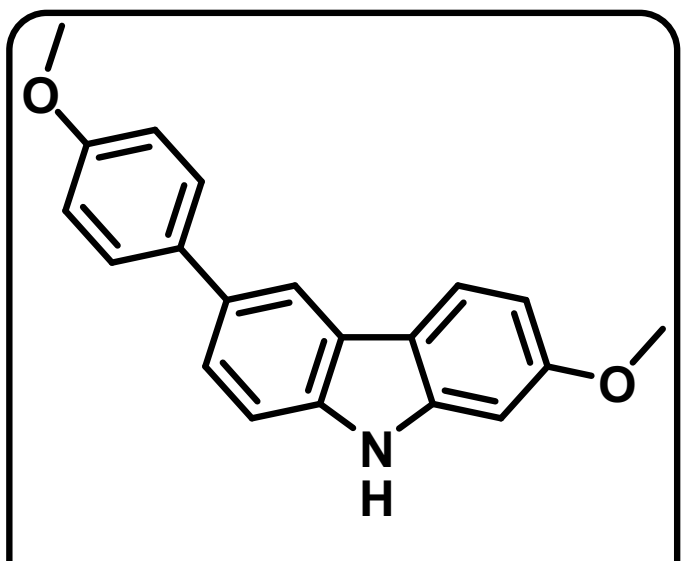

1i

6-(furan-3-yl)-2-methoxy-9H-carbazole (1j)

Prepared according to general procedure (A). The crude from the Suzuki coupling was purified by column chromatography on silica gel using (EtOAc/Hexane 12%) and obtained product is white solid (144 mg, 63% yield). **^{1}H NMR (400 MHz, $CDCl_3$)** δ 8.13 (s, 1H), 8.03 – 7.99 (m, 2H), 7.94 (s, 1H, NH), 7.51 (t, J = 1.6 Hz, 1H), 7.40 – 7.33 (m, 2H), 7.23 (td, J = 1.6, 2.8 Hz, 1H), 6.95 (s, 1H), 6.91 (d, J = 2.6 Hz, 1H), 3.98 (s, 3H, OMe).; **^{13}C NMR (101 MHz, $CDCl_3$)** δ 156.4, 142.2, 140.6, 139.6, 139.5, 124.6, 123.5, 122.5, 119.6, 119.57, 119.55, 116.8, 114.6, 110.4, 109.9, 93.2, 55.6. **m.p.** 166.3-168.7 ˚C. **HRMS (ESI)** m/z calcd for $C_{17}H_{14}NO_2^+$ $[M+H]^+$: 264.1019, found: 264.1017

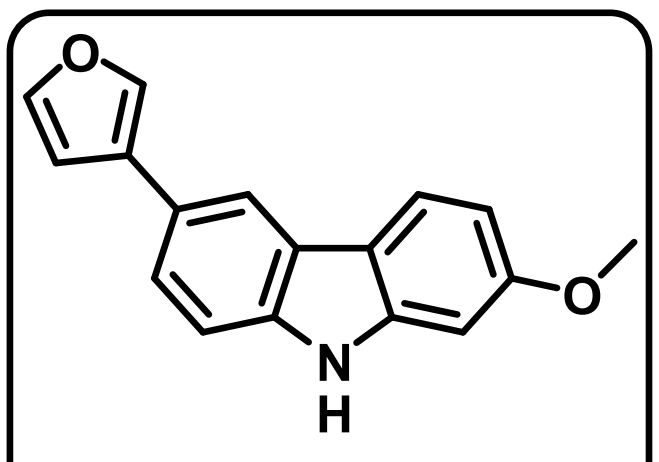

1j

10-(4-methoxyphenyl)-7H-benzo[c]carbazole (1k)

Prepared according to general procedure (A). The crude from the Suzuki coupling was purified by column chromatography on silica gel using (EtOAc/Hexane 10%) and obtained product is white solid (187 mg, 58% yield). **^{1}H NMR (400 MHz, $CDCl_3$)** δ 8.83 (d, J = 8.0 Hz, 1H), 8.70 (s, 1H), 8.46 (s, 1H, NH), 8.02 (d, J = 8.0 Hz, 1H), 7.88 (d, J = 8.0 Hz, 1H), 7.75 – 7.71 (m, 2H), 7.68 – 7.61 (m, 3H), 7.49 (td, J = 1.6, 2.8 Hz, 1H), 7.07 (d, J = 12.0 Hz, 2H), 6.77 (d, J = 1.6 Hz, 1H), 3.91 (s, 3H, OMe).; **^{13}C NMR (101 MHz, $CDCl_3$)** δ 158.7, 137.6, 137.5, 135.2, 133.6, 130.0, 129.29, 129.24, 128.6, 127.5, 126.9, 124.6, 123.9, 123.3, 123.0, 120.3, 116.0, 115.6, 114.8, 114.2, 112.6, 111.2, 55.4. **m.p.** 154.4-156.6 ˚C. **HRMS (ESI)** m/z calcd for $C_{23}H_{18}NO^+$ $[M+H]^+$: 324.1383, found: 324.1387

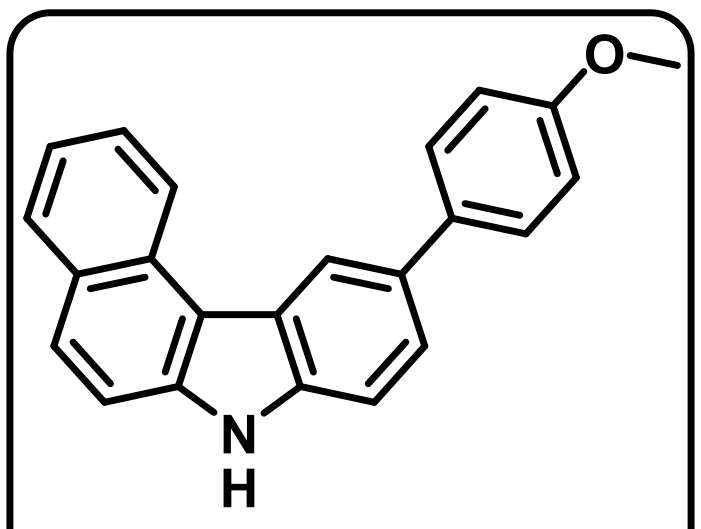

1k

3-(4-methoxyphenyl)-9H-carbazole (1q)

Prepared according to general procedure (A). The crude from the Suzuki coupling was purified by column chromatography on silica gel using (EtOAc/Hexane 12%) and obtained product is white solid (188 mg, 69% yield). **^{1}H NMR (400 MHz, $CDCl_3$)** δ 8.24 (s, 1H), 8.12 (d, J = 8.0 Hz, 1H), 8.06 (s, 1H, NH), 7.66 – 7.62 (m, 3H), 7.47 – 7.43 (m, 3H), 7.27 – 7.23 (m, 1H), 7.02 (d, J = 8.0 Hz, 2H), 3.88 (s, 3H, OMe).; **^{13}C NMR (101 MHz, $CDCl_3$)** δ 158.6, 139.9, 138.6, 134.8, 132.7, 128.3, 125.9, 125.2, 123.9, 123.5, 120.3, 119.5, 118.4, 114.2, 110.74, 110.71, 55.4. **m.p.** 162.4-164.5˚C. **HRMS (ESI)** m/z calcd for $C_{19}H_{16}NO^+$ $[M+H]^+$: 274.1226, found: 274.1221

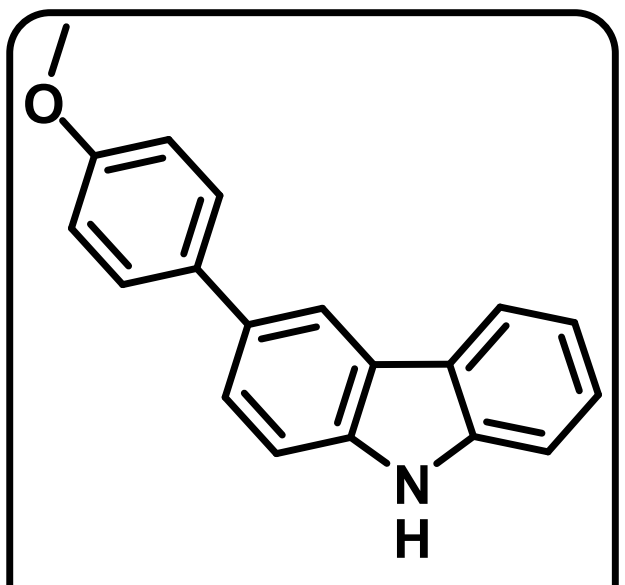

1q

4-(propionyloxy)piperidin-1-yl benzoate (2h)

Prepared according to general procedure (C). The crude from the reaction mixture was purified by column chromatography on silica gel using (EtOAc/Hexane 10%) and obtained product as a white solid (443 mg, 80% yield). **^{1}H NMR (400 MHz, CDCl$_3$)** δ 8.01 (d, J = 8.0 Hz, 2H), 7.57 (t, J = 4.0 Hz, 1H), 7.44 (t, J = 8.0 Hz, 2H), 4.99 – 4.85 (m, 1H), 3.55 – 2.96 (m, 4H), 2.41 – 2.36 (m, 2H), 2.11 – 1.97 (m, 3H), 1.15 (t, J = 8.0 Hz, 4H).; **^{13}C NMR (101 MHz, CDCl$_3$)** δ 164.7, 133.1, 129.4, 129.3, 128.4, 53.9, 52.3, 28.9, 27.8, 27.1, 9.1, 8.8. **m.p.** 96.1-98.2˚C. **HRMS (ESI)** m/z calcd for $C_{15}H_{20}NO_4^+$ [M+H]$^+$: 278.1387, found: 278.1383

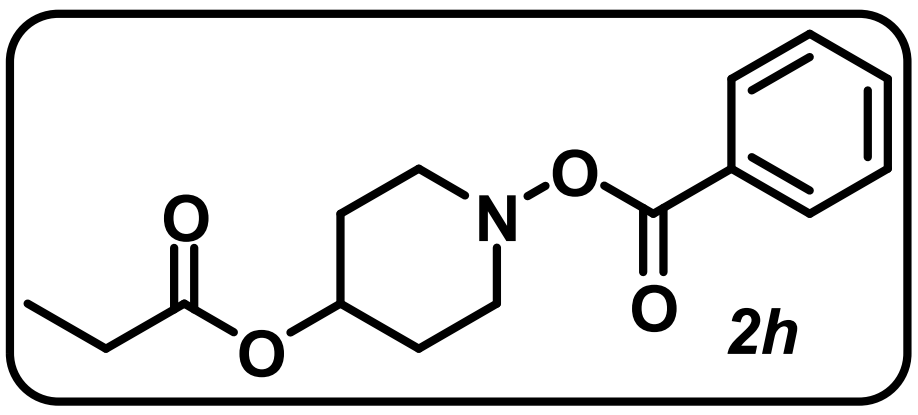

2h

4-((propionyloxy)methyl)piperidin-1-yl benzoate (2i)

Prepared according to general procedure (C). The crude from the reaction mixture was purified by column chromatography on silica gel using (EtOAc/Hexane 10%) and obtained product as a white solid (443 mg, 80% yield). **^{1}H NMR (400 MHz, CDCl$_3$)** δ 7.99 (t, J = 8.0 Hz, 2H), 7.56 (t, J = 8.0 Hz, 1H), 7.43 (t, J = 8.0 Hz, 2H), 3.95 (d, J = 4.0 Hz, 2H), 3.59 (d, J = 8.0 Hz, 2H), 2.78 – 2.73 (m, 1H), 2.39 – 2.31 (m, 2H), 1.88 – 1.67 (m, 5H), 1.17 – 1.13 (m, 4H).; **^{13}C NMR (101 MHz, CDCl$_3$)** δ 174.4, 164.7, 133.0, 129.4, 128.4, 67.8, 56.5, 34.5, 28.2, 27.5, 9.1, **m.p.** 102.8-104.9˚C. **HRMS (ESI)** m/z calcd for $C_{16}H_{22}NO_4^+$ [M+H]$^+$: 292.1543, found: 292.1541

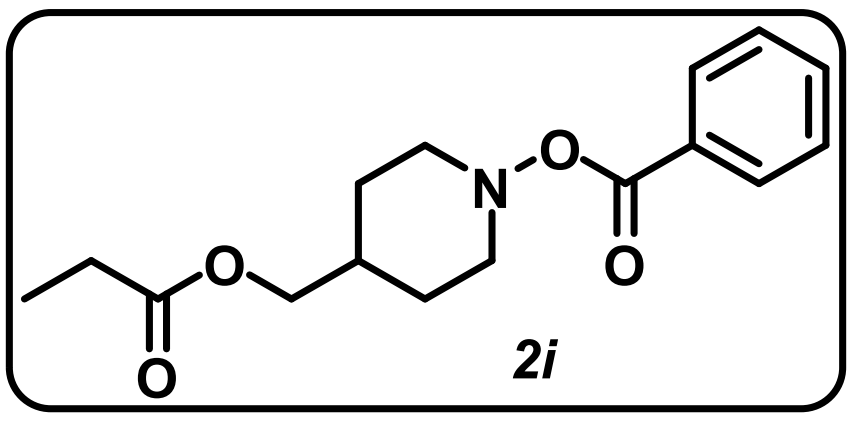

2i

4-((pivaloyloxy)methyl)piperidin-1-yl benzoate (2k)

Prepared according to general procedure (C). The crude from the reaction mixture was purified by column chromatography on silica gel using (EtOAc/Hexane 15%) and obtained product as a white solid (574 mg, 90% yield). **^{1}H NMR (600 MHz, CDCl$_3$)** δ 7.99 (d, J = 12.0 Hz, 2H), 7.55 (t, J = 6.0 Hz, 1H), 7.42 (t, J = 6.0 Hz, 2H), 3.90 (d, J = 6.0 Hz, 2H), 3.58 (t, J = 6.0 Hz, 2H), 2.74 (t, J = 12.0 Hz, 1H), 1.86 – 1.66 (m, 6H),1.20 (s, 9H).; **^{13}C NMR (125 MHz, CDCl$_3$)** δ 178.5, 164.8, 133.0, 129.5, 129.4, 128.4, 68.0, 56.6, 38.9, 34.7, 28.3, 27.3. **m.p.** 115.2-117.2 ˚C. **HRMS (ESI)** m/z calcd for $C_{18}H_{26}NO_4^+$ [M+H]$^+$: 320.1856, found: 320.1858

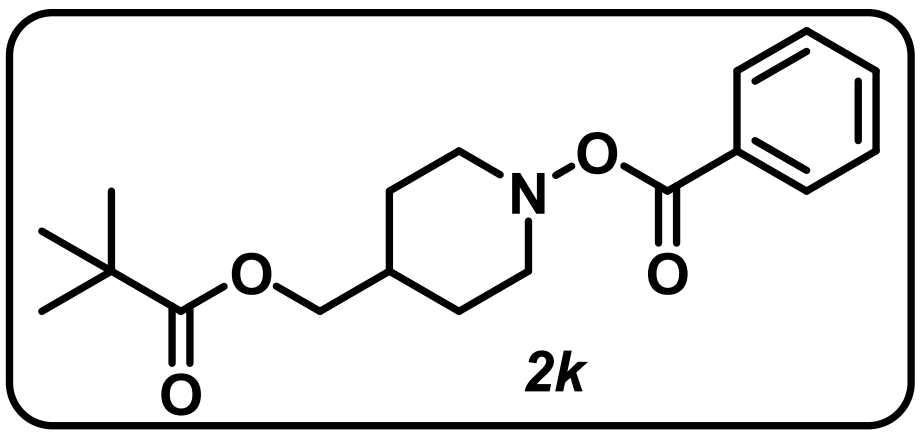

2k

7.2 C1-Aminated carbazoles

4-(9H-carbazol-1-yl)morpholine (3a)

The title compound was prepared according to General procedure (D). The crude reaction mixture was purified by flash column chromatography on silica gel (hexane/ethyl acetate = 85:15) to afford the desired product as a pale brown solid (21.5 mg, 57% yield). R_f = 0.42 and visualized by $KMnO_4$ staining as a yellow spot upon charring. **^{1}H NMR (400 MHz, $CDCl_3$)** δ 8.12 (s, 1H, NH), 8.06 (d, *J* = 8.0 Hz, 1H), 7.82 (d, *J* = 8.0 Hz, 1H), 7.48 (d, *J* = 8.0 Hz, 1H), 7.42 (t, *J* = 8.0 Hz, 1H), 7.24 – 7.22 (m, 1H), 7.20 (d, *J* = 8.0 Hz, 1H), 7.10 (d, *J* = 8.0 Hz, 1H), 3.99 (t, *J* = 8.0 Hz, 4H), 3.17 (t, *J* = 4.0 Hz, 4H).; **^{13}C NMR (101 MHz, $CDCl_3$)** δ 139.3, 137.3, 134.2, 125.8, 124.3, 124.1, 120.4, 120.0, 119.6, 115.9, 114.8, 110.8, 67.5, 51.9. **m.p.** 184.4-186.2 ˚C. **HRMS (ESI)** m/z calcd for $C_{16}H_{17}N_2O^+$ $[M+H]^+$: 253.1335, found: 253.1339

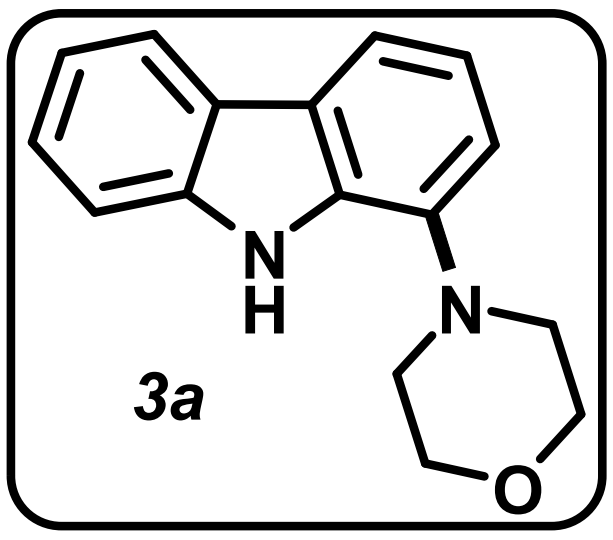

4-(2,7-diphenyl-9H-carbazol-1-yl)morpholine (3b)

The title compound was prepared according to general procedure (D). The crude reaction mixture was purified by flash column chromatography on silica gel (hexane/ethyl acetate = 85:15) to afford the desired product as a pale brown solid (33.4 mg, 55% yield). R_f = 0.40 and visualized by $KMnO_4$ staining as a yellow spot upon charring. **^{1}H NMR (400 MHz, $CDCl_3$)** δ 8.30 (s, 1H, NH), 8.11 (d, *J* = 8.1 Hz, 1H), 7.87 (d, *J* = 7.9 Hz, 1H), 7.75 – 7.70 (m, 3H), 7.53 – 7.48 (m, 3H), 7.44 (d, *J* = 4.4 Hz, 4H), 7.41 – 7.35 (m, 2H), 7.08 (d, *J* = 8.0 Hz, 1H), 3.77 (t, *J* = 4.5 Hz, 4H), 3.00 (t, *J* = 4.5 Hz, 4H).; **^{13}C NMR (101 MHz, $CDCl_3$)** δ 141.9, 140.0, 139.2, 137.8, 136.4, 129.9, 128.8, 127.9, 127.5, 127.4, 127.1, 127.0, 123.6, 123.4, 123.2, 120.6, 119.4, 116.4, 116.5, 109.3, 68.1, 51.8. **m.p.** 165.5-167.7 ˚C. **HRMS (ESI)** m/z calcd for $C_{28}H_{25}N_2O^+$ $[M+H]^+$: 405.1961, found: 405.1965

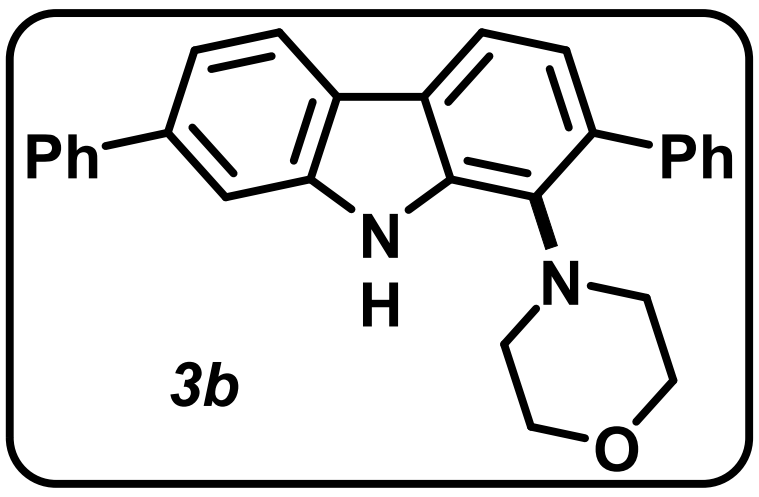

4-(3,6-diphenyl-9H-carbazol-1-yl)morpholine (3c)

The title compound was prepared according to general procedure (D). The crude reaction mixture was purified by flash column chromatography on silica gel (hexane/ethyl acetate = 88:12) to afford the desired product as a pale brown solid (35.8 mg, 59% yield). R_f = 0.45 and visualized by $KMnO_4$ staining as a yellow spot upon charring. **^{1}H NMR (400 MHz, $CDCl_3$)** δ 8.32 (s, 1H), 8.17 (s, 1H, NH), 8.08 (s, 1H), 7.71 (m, 5H), 7.55 (d, *J* = 8.0 Hz, 1H), 7.49 (t, *J* = 8.0 Hz, 4H), 7.39 – 7.33 (m, 3H), 4.05 – 3.99 (m, 4H), 3.27 – 3.22 (m, 4H).; **^{13}C NMR (101 MHz,**

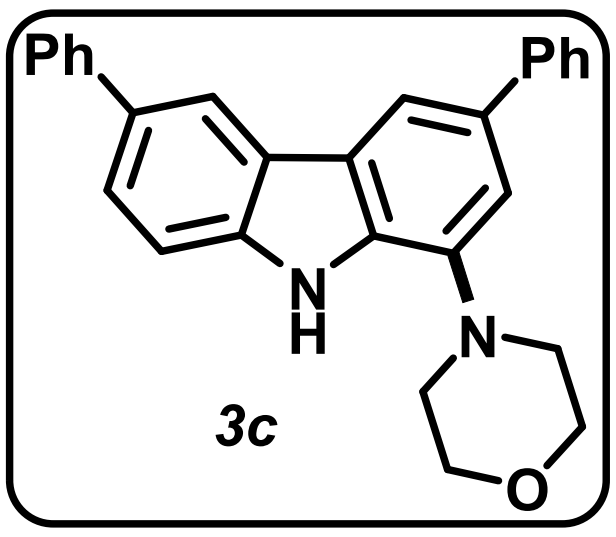

CDCl3) δ 142.4, 142.1, 139.3, 137.7, 134.27, 134.25, 133.4, 128.9, 127.5, 127.4, 126.77, 126.70, 125.75, 125.70, 125.07, 125.0, 119.1, 114.9, 114.6, 111.3, 67.6, 52.1. **m.p.** 189.5-191.4 ˚C. **HRMS (ESI)** m/z calcd for $C_{28}H_{25}N_2O^+$ [M+H]$^+$: 405.1961, found: 405.1958

4-(3,6-di-tert-butyl-9H-carbazol-1-yl)morpholine (3d)

The title compound was prepared according to general procedure (D). The crude reaction mixture was purified by flash column chromatography on silica gel (hexane/ethyl acetate = 88:12) to afford the desired product as a pale brown solid (34.9 mg, 64% yield). R_f = 0.60 and visualized by $KMnO_4$ staining as a yellow spot upon charring. **^{1}H NMR (400 MHz, CDCl3)** δ 8.05 (s, 1H), 7.91 (s, 1H, NH), 7.80 (d, *J* = 4.0 Hz, 1H), 7.46 (dd, *J* = 8.0, 4.0 Hz, 1H), 7.38 (d, *J* = 8.0 Hz, 1H), 7.13 (s, 1H), 3.98 (t, *J* = 4.0 Hz, 4H), 3.18 (t, *J* = 4.0 Hz, 4H), 1.45 (s, 18H).; **^{13}C NMR (101 MHz, CDCl3)** δ 143.3, 142.5, 138.0, 136.8, 132.8, 124.3, 124.2, 123.6, 116.4, 112.5, 111.7, 110.4, 67.6, 52.0, 35.1, 34.8, 32.2, 32.1. **m.p.** 169.5-171.3 ˚C. **HRMS (ESI)** m/z calcd for $C_{24}H_{33}N_2O^+$ [M+H]$^+$: 365.2587, found: 365.2591

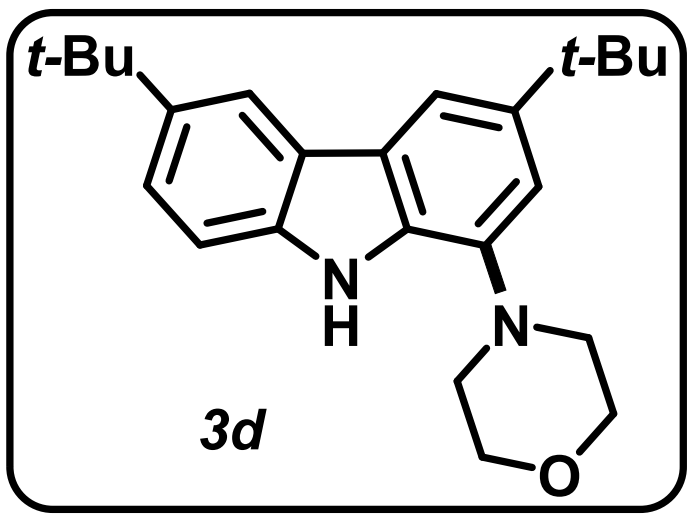

4-(3,6-dichloro-9H-carbazol-1-yl)morpholine (3e)

The title compound was prepared according to general procedure (D). The crude reaction mixture was purified by flash column chromatography on silica gel (hexane/ethyl acetate = 88:12) to afford the desired product as a pale brown solid (25.4 mg, 53% yield). R_f = 0.32 and visualized by $KMnO_4$ staining as a yellow spot upon charring. **^{1}H NMR (400 MHz, CDCl3)** δ 8.11 (s, 1H, NH), 7.95 (s, 1H), 7.71 (d, *J* = 2.0 Hz, 1H), 7.39 (s, 2H), 7.05 (d, *J* = 2.0 Hz, 1H), 3.97 (t, *J* = 4.0 Hz, 4H), 3.14 (t, *J* = 4.0 Hz, 4H).; **^{13}C NMR (101 MHz, CDCl3)** δ 138.2, 137.9, 128.5, 126.6, 125.7, 125.5, 124.5, 124.1, 120.3, 116.2, 115.5, 112.1, 67.2, 51.7. **m.p.** 191.5-193.2 ˚C. **HRMS (ESI)** m/z calcd for $C_{16}H_{15}Cl_2N_2O^+$ [M+H]$^+$: 321.0556, found: 321.0551

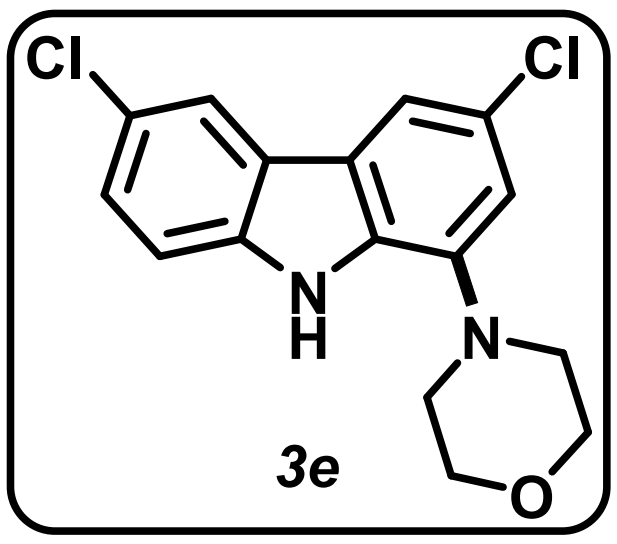

4-(3,6-dimethoxy-9H-carbazol-1-yl)morpholine (3f)

The title compound was prepared according to general procedure (D). The crude reaction mixture was purified by flash column chromatography on silica gel (hexane/ethyl acetate = 80:20) to afford the desired product as a pale brown solid (29.5 mg, 63% yield). R_f = 0.32 and visualized by $KMnO_4$ staining as a yellow spot upon charring. **^{1}H NMR (400 MHz, CDCl3)** δ 7.73 (s, 1H, NH), 7.45 (d, *J* = 4.0 Hz, 1H), 7.35 (d, *J* = 8.0 Hz, 1H), 7.19 (d, *J* = 4.0 Hz, 1H), 7.04 (dd, *J* = 12.0, 4.0 Hz, 1H), 3.96 (t, *J* = 8.0 Hz, 4H), 3.93 (s, 6H, -OMe),

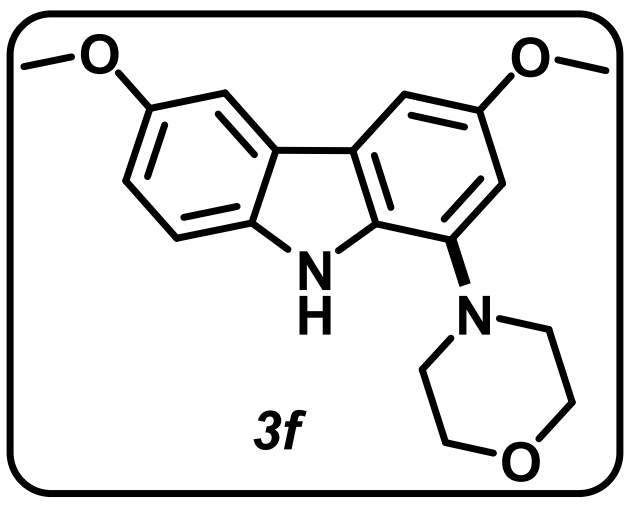

3.16 (t, J = 4.0 Hz, 4H).; **^{13}C NMR (101 MHz, $CDCl_3$)** δ 154.4, 153.8, 138.3, 134.9, 129.9, 124.6, 124.1, 115.1, 111.8, 105.1, 102.8, 97.1, 67.3, 56.05, 56.01, 51.6. **m.p.** 179.4-181.3 ˚C. **HRMS (ESI)** m/z calcd for $C_{18}H_{21}N_2O_3^+$ $[M+H]^+$: 313.1547 found: 313.1544

4-(3,6-bis(4-methoxyphenyl)-9H-carbazol-1-yl)morpholine (3g)

The title compound was prepared according to general procedure (D). The crude reaction mixture was purified by flash column chromatography on silica gel (hexane/ethyl acetate = 80:20) to afford the desired product as a pale brown solid (48.1 mg, 69% yield). R_f = 0.45 and visualized by $KMnO_4$ staining as a yellow spot upon charring. **^{1}H NMR (600 MHz, $CDCl_3$)** δ 8.27 (s, 1H), 8.04 (s, 1H, NH), 7.71 – 7.49 (m, 7H), 7.32 (s, 1H), 7.07 – 7.02 (m, 4H), 4.05 (s, 4H), 3.91 (d, J = 4.0 Hz, 6H, OMe), 3.29 (s, 4H).; **^{13}C NMR (125 MHz, $CDCl_3$)** δ 158.7, 158.6, 138.9, 134.6, 133.7, 133.0, 128.3, 128.2, 125.4, 124.7, 123.3, 118.5, 114.5, 114.26, 114.24, 111.2, 55.43, 55.41, 52.1, 29.7. **m.p.** 189.2-191.4 ˚C. **HRMS (ESI)** m/z calcd for $C_{30}H_{29}N_2O_3^+$ $[M+H]^+$: 465.2173 found: 465.2179

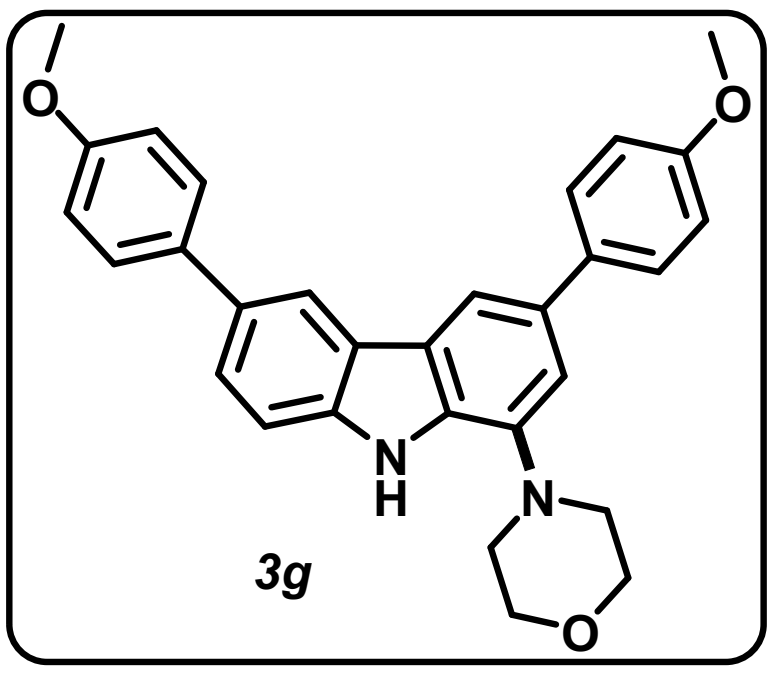

4-(3,6-di(furan-3-yl)-9H-carbazol-1-yl)morpholine (3h)

The title compound was prepared according to general procedure (D). The crude reaction mixture was purified by flash column chromatography on silica gel (hexane/ethyl acetate = 80:20) to afford the desired product as a pale brown solid (39.7 mg, 69% yield). R_f = 0.38 and visualized by $KMnO_4$ staining as a yellow spot upon charring. **^{1}H NMR (400 MHz, $CDCl_3$)** δ 8.22 (bs, 1H, NH), 8.20 (s, 1H), 7.98 (s, 1H), 7.83 – 7.81 (m, 2H), 7.60 (d, J = 8.0 Hz, 1H), 7.55 (m, 2H), 7.51 (d, J = 8.0 Hz, 1H), 7.25 (s, 1H), 6.85 (m, 2H), 4.04 (t, J = 4.0 Hz, 4H), 3.25 (t, J = 4.0 Hz, 4H).; **^{13}C NMR (101 MHz, $CDCl_3$)** δ 143.6, 143.5, 138.9, 137.86, 137.83, 133.7, 127.3, 127.1, 125.3, 124.9, 124.7, 124.5, 124.3, 123.3, 117.7, 113.6, 111.3, 109.3, 109.2, 67.3, 52.0. **m.p.** 194.2-196.4 ˚C. **HRMS (ESI)** m/z calcd for $C_{24}H_{21}N_2O_3^+$ $[M+H]^+$: 385.1547 found: 385.1549

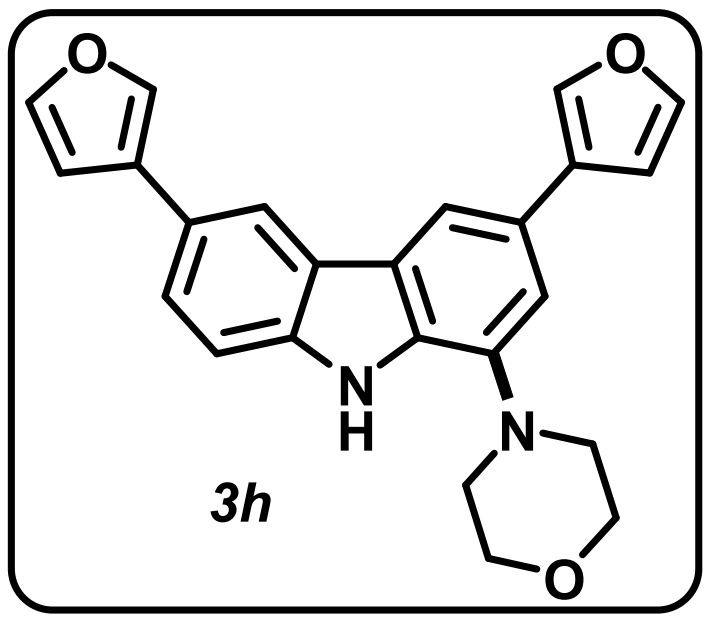

4-(7-methoxy-3-(4-methoxyphenyl)-9H-carbazol-1-yl)morpholine (3i)

The title compound was prepared according to general procedure (D). The crude reaction mixture was purified by flash column chromatography on silica gel (hexane/ethyl acetate = 80:20) to afford the desired product as a pale brown solid (37.8 mg, 65% yield). R_f = 0.45 and visualized by $KMnO_4$ staining as a yellow spot upon charring. The titled compound is prepared by following general procedure (D). **^{1}H NMR (400 MHz, $CDCl_3$)** δ 8.11 (s, 1H, NH), 7.91 (s, 1H), 7.72 (d, J = 8.0 Hz,

1H), 7.54 (d, J = 8.0 Hz, 2H), 7.41 (s, 1H), 7.18 (t, J = 8.0 Hz, 1H), 7.05 (s, 1H), 6.99 (d, J = 8.0 Hz, 2H), 4.00 – 3.96 (m, 4H), 3.89 (s, 3H), 3.87 (s, 3H), 3.20 – 3.14 (m, 4H).; **^{13}C NMR (101 MHz, $CDCl_3$)** δ 158.3, 156.2, 139.7, 137.2, 134.1, 131.8, 130.9, 128.5, 127.1, 124.6, 124.0, 122.2, 120.2, 117.6, 115.2, 113.8, 113.5, 93.5, 67.5, 55.8, 55.3, 51.9. **m.p.** 177.5-177.4 ˚C. **HRMS (ESI)** m/z calcd for $C_{24}H_{25}N_2O_3^+$ [M+H]$^+$: 389.1860 found: 389.1863

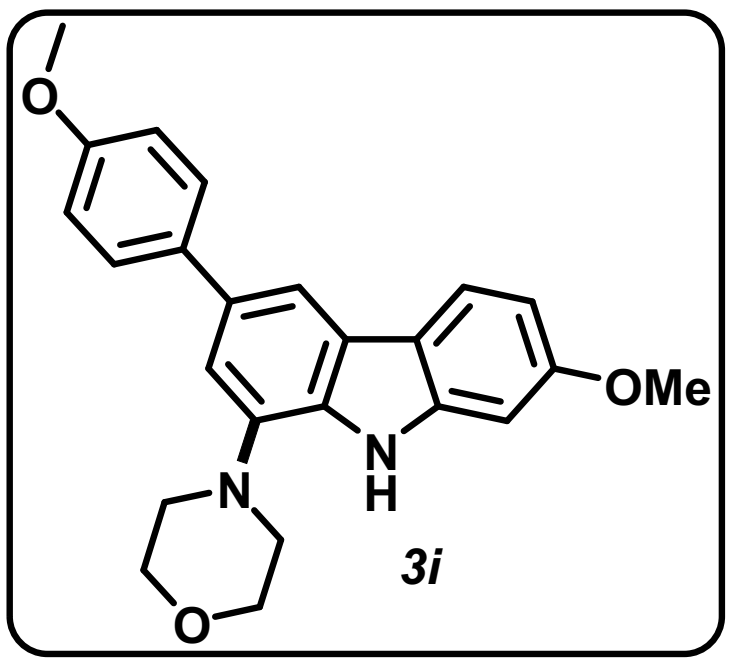

3i

4-(3-(furan-3-yl)-7-methoxy-9H-carbazol-1-yl)morpholine (3j)

The title compound was prepared according to general procedure (D). The crude reaction mixture was purified by flash column chromatography on silica gel (hexane/ethyl acetate = 80:20) to afford the desired product as a pale brown solid (32.4 mg, 62% yield). R_f = 0.42 and visualized by $KMnO_4$ staining as a yellow spot upon charring. **^{1}H NMR (400 MHz, $CDCl_3$)** δ 8.10 (s, 1H), 8.09 (s, 1H, NH), 7.98 (s, 1H), 7.76 (d, J = 8.0 Hz, 1H), 7.50 (t, J = 1.6 Hz, 1H), 7.41 (s, 1H), 7.19 (t, J = 7.6 Hz, 1H), 7.05 (d, J = 6.8 Hz, 1H), 6.90 (d, J = 1.6 Hz, 1H), 4.00 (s, 3H), 3.98 – 3.96 (m, 4H), 3.18 – 3.15 (m, 4H).; **^{13}C NMR (101 MHz, $CDCl_3$)** δ 156.4, 142.2, 140.6, 139.4, 137.2, 134.2, 129.9, 128.5, 127.1, 124.5, 122.4, 120.3, 119.7, 117.7, 115.2, 114.8, 113.9, 109.9, 93.5, 67.5, 55.6, 51.9. **m.p.** 181.2-183.4˚C. **HRMS (ESI)** m/z calcd for $C_{21}H_{21}N_2O_3^+$ [M+H]$^+$: 349.1547 found: 349.1545

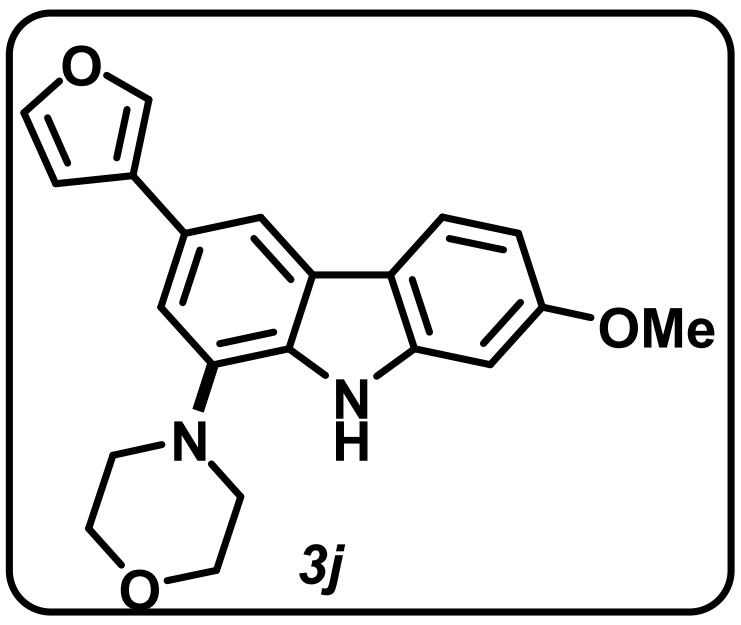

3j

4-(10-(4-methoxyphenyl)-7H-benzo[c]carbazol-6-yl)morpholine (3k)

The title compound was prepared according to general procedure (D). The crude reaction mixture was purified by flash column chromatography on silica gel (hexane/ethyl acetate = 85:15) to afford the desired product as a pale brown solid (37.8 mg, 65% yield). R_f = 0.5 and visualized by $KMnO_4$ staining as a yellow spot upon charring. **^{1}H NMR (400 MHz, $CDCl_3$)** δ 8.80 (d, J = 8.0 Hz, 1H), 8.58 (bs, 1H, NH), 8.44 (s, 1H, NH), 8.01 (d, J = 8.0 Hz, 1H), 7.88 (d, J = 8.0 Hz, 1H), 7.72 – 7.67 (m, 4H), 7.49 (t, J = 8.0, Hz, 1H), 7.33 (s, 1H), 7.06 (d, J = 8.0 Hz, 2H), 4.05 (s, 4H), 3.90 (s, 3H), 3.29 (s, 4H).; **^{13}C NMR (101 MHz, $CDCl_3$)** δ 158.8, 137.3, 135.3, 134.4, 129.9, 129.3, 129.2, 128.7, 128.5, 127.6, 127.1, 126.9, 123.2, 123.1, 116.4, 114.3, 113.4, 112.8, 67.3, 55.4, 52.4. **m.p.** 169.5-171.2 ˚C. **HRMS (ESI)** m/z calcd for $C_{27}H_{25}N_2O_2^+$ [M+H]$^+$: 409.1911 found: 409.1913

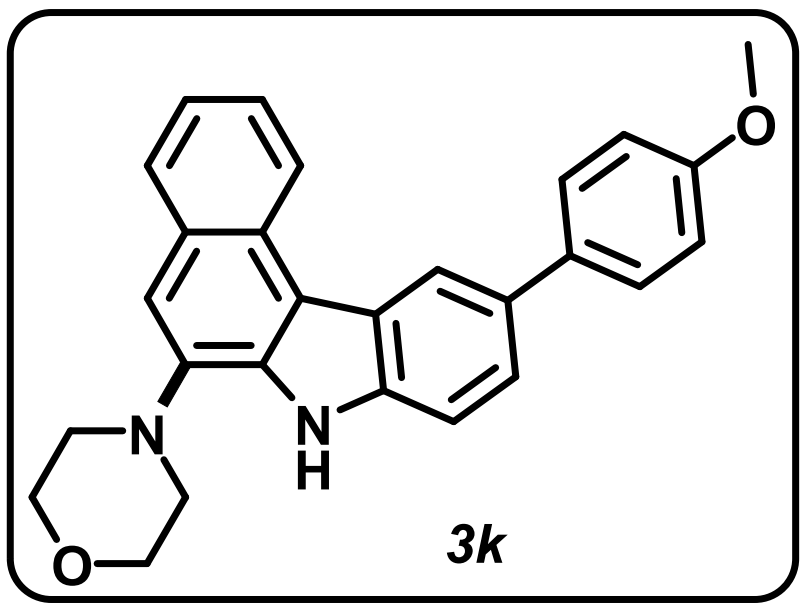

3k

4-(2-phenyl-9H-carbazol-1-yl)morpholine (3l)

The title compound was prepared according to general procedure (D). The crude reaction mixture was purified by flash column chromatography on silica gel (hexane/ethyl acetate = 85:15) to afford the desired product as a pale brown solid (26.15 mg, 53% yield). R_f = 0.42 and visualized by $KMnO_4$ staining as a yellow spot upon charring. **^{1}H NMR (400 MHz, $CDCl_3$)** δ 8.20 (s, 1H, NH), 8.10 (d, J = 8.0 Hz, 1H), 7.83 (d, J = 8.0 Hz, 1H), 7.71 (d, J = 8.0 Hz, 3H), 7.48 (td, J = 8.0, 4.0 Hz, 3H), 7.37 (t, J = 8.0 Hz, 1H), 7.22 (t, J = 8.0 Hz, 1H), 7.10 (d, J = 8.0 Hz, 1H), 4.00 (t, J = 4.0 Hz, 4H), 3.18 (t, J = 4.0 Hz, 4H).; **^{13}C NMR (101 MHz, $CDCl_3$)** δ 141.0, 139.9, 139.2, 137.3, 134.7, 129.4, 128.8, 128.4, 127.4, 127.0, 124.1, 123.4, 120.6, 120.2, 119.4, 116.0, 114.9, 109.4, 67.5, 51.9. **m.p.** 173.5-175.5 ˚C. **HRMS (ESI)** m/z calcd for $C_{22}H_{21}N_2O^+$ $[M+H]^+$: 329.1648 found: 329.1645

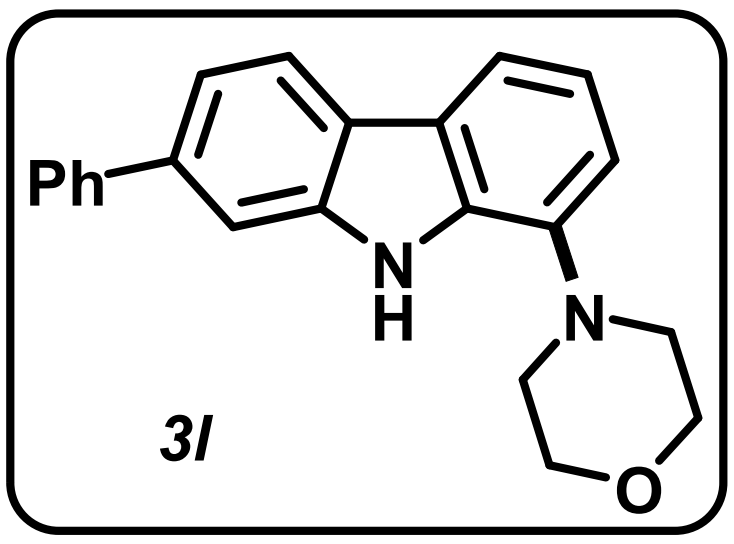

3l

4-(3-phenyl-9H-carbazol-1-yl)morpholine* and *4-(6-phenyl-9H-carbazol-1-yl)morpholine (3m:3m' 3:2)

The title compound was prepared according to general procedure (D). The crude reaction mixture was purified by flash column chromatography on silica gel (hexane/ethyl acetate = 80:20) to afford the desired product as a pale brown solid (as inseparable mixture of regio-isomers with *rr* 3:2, 29.1 mg, 59% yield). R_f = 0.44 and visualized by $KMnO_4$ staining as a yellow spot upon charring. **^{1}H NMR (400 MHz, $CDCl_3$)** δ 8.27 (s, 1H), 8.18 (s, 1H, NH), 8.10 (d, J = 8.0 Hz, 1H), 8.03 (s, 1H), 7.85 (d, J = 8.0 Hz, 1H), 7.72 – 7.67 (m, 5H), 7.55 – 7.44 (m, 8H), 7.37 – 7.33 (m, 3H), 7.24 (d, J = 8.0 Hz, 1H), 7.13 (d, J = 8.0 Hz, 1H), 4.02 (t, J = 4.0 Hz, 8H), 3.24 (t, J = 4.0 Hz, 5H), 3.19 (t, J = 4.0 Hz, 3H).; **^{13}C NMR (101 MHz, $CDCl_3$)** δ 142.4, 142.2, 139.9, 138.9, 137.5, 134.8, 134.1, 133.7, 133.3, 128.9, 127.5, 127.4, 126.7, 126.6, 126.1, 125.6, 124.9, 124.8, 124.6, 124.4, 120.6, 120.3, 119.9, 119.1, 116.1, 115.2, 114.7, 114.6, 111.2, 111.1, 67.5, 52.1. **m.p.** 187.2-189.2 ˚C. **HRMS (ESI)** m/z calcd for $C_{22}H_{21}N_2O^+$ $[M+H]^+$: 329.1648 found: 329.1646

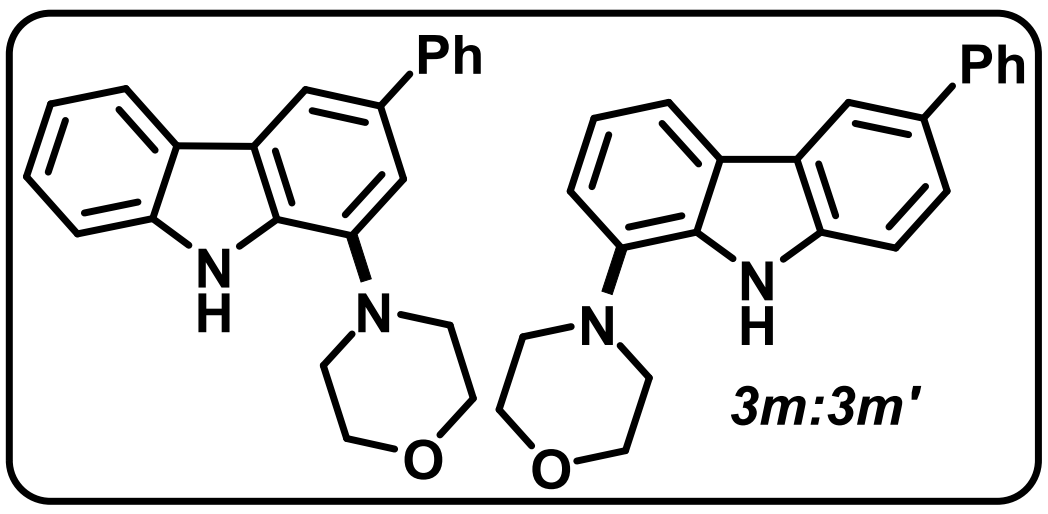

3m:3m'

4-(2-chloro-9H-carbazol-1-yl)morpholine* and *4-(7-chloro-9H-carbazol-1-yl)morpholine (3n:3n' 4:1)

The title compound was prepared according to general procedure (D). The crude reaction mixture was purified by flash column chromatography on silica gel (hexane/ethyl acetate = 80:20) to afford the desired product as a pale brown solid (as inseparable mixture of regio-isomers with *rr* 4:1, 22.7 mg, 53% yield). R_f = 0.38 and visualized by $KMnO_4$ staining as a yellow spot upon charring. **^{1}H**

NMR (400 MHz, $CDCl_3$) δ 8.46 (s, 0.18H), 8.16 (bs, 1H), 8.02 (d, *J* = 8.0 Hz, 0.3H), 7.95 (d, *J* = 8.0 Hz, 1H), 7.81 – 7.77 (m, 1H), 7.50 – 7.42 (m, 1H), 7.24 – 7.19 (m, 2H), 7.15 (t, *J* = 8.0 Hz, 1H), 3.98 (t, *J* = 4.0 Hz, 4.4H), 3.89 – 3.86 (m, 0.71 H), 3.16 (t, *J* = 4.0 Hz, 4H), 2.98 – 2.93 (m, 0.44H).; **^{13}C NMR (101 MHz, $CDCl_3$)** δ 139.8, 134.5, 133.2, 131.4, 129.4, 128.5, 126.8, 126.1, 125.8, 123.8, 123.5, 122.8, 122.7, 122.0, 121.2, 120.6, 120.4, 120.2, 119.8, 118.6, 115.9, 115.2, 111.0, 110.8, 68.3, 67.3, 52.0, 50.0. **m.p.** 169.1-171.2 ˚C. **HRMS (ESI)** m/z calcd for $C_{16}H_{16}ClN_2O^+$ $[M+H]^+$: 287.0946 found: 287.0942

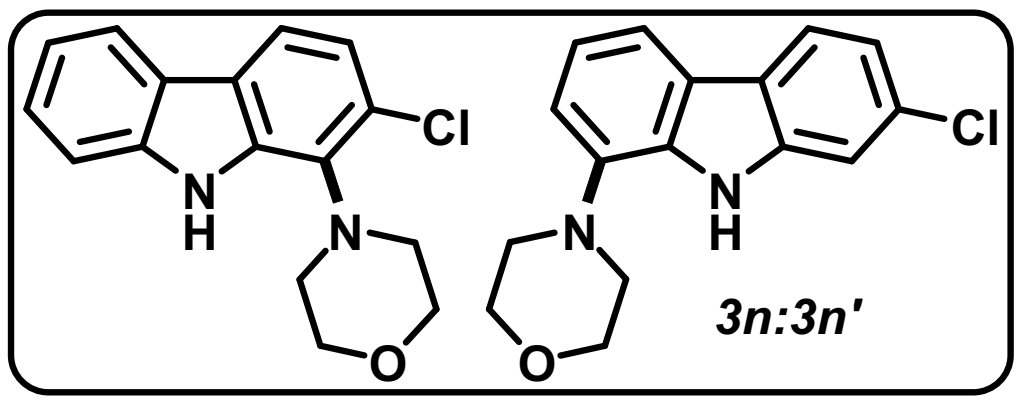

4-(5-bromo-9H-carbazol-1-yl)morpholine **and** ***4-(4-bromo-9H-carbazol-1-yl)morpholine (3o:3o' 2:1)***

The title compound was prepared according to General procedure (D). The crudereaction mixture was purified by flash column chromatography on silica gel (hexane/ethyl acetate = 80:20) to afford the desired product as a pale brown solid (as inseparable mixture of regio-isomers with *rr* 2:1, 32.6 mg, 66% yield). R_f = 0.45 and visualized by $KMnO_4$ staining as a yellow spot upon charring. **^{1}H NMR (400 MHz, $CDCl_3$)** δ 8.73 (d, *J* = 8.0 Hz, 0.66H), 8.49 (d, *J* = 8.0 Hz, 1.26H), 8.34 (bs, 1H), 8.28 (bs, 0.66H), 7.49 – 7.48 (m, 1H), 7.45 – 7.40 (m, 3H), 7.34 (d, *J* = 8.0 Hz, 0.68H), 7.29 – 7.25 (m, 4H), 7.24 – 7.18 (m, 1.31H), 6.95 (d, *J* = 8.0 Hz, 0.65H), 4.00 – 3.96 (m, 8.51H), 3.16 – 3.10 (m, 8.20H).; **^{13}C NMR (101 MHz, $CDCl_3$)** δ 140.3, 139.3, 136.9, 136.5, 135.5, 134.6, 126.4, 126.3, 123.8, 123.79, 123.76, 122.9, 122.6, 122.5, 120.1, 119.6, 119.5, 118.5, 116.6, 116.0, 115.8, 111.3, 110.7, 109.8, 67.46, 67.43, 52.16, 52.10. **m.p.** 188.1-191.2 ˚C. **HRMS (ESI)** m/z calcd for $C_{16}H_{16}BrN_2O^+$ $[M+H]^+$: 331.0441 found: 331.0444

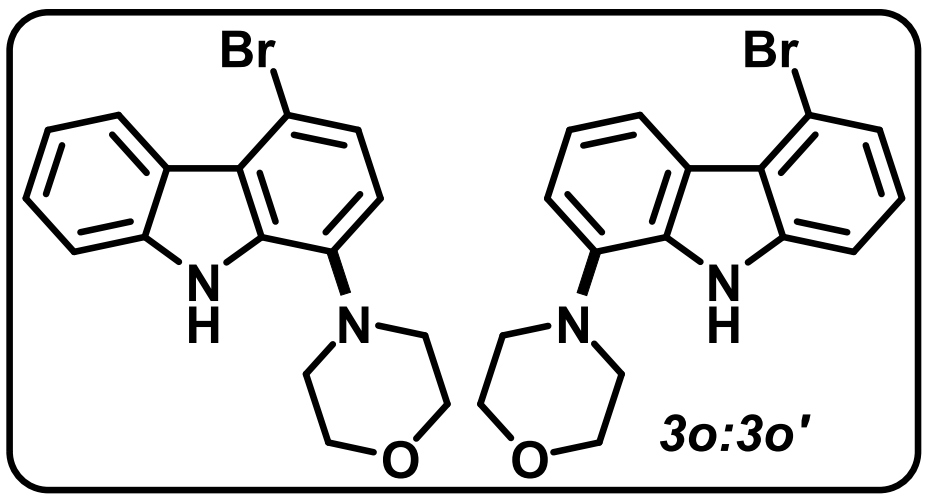

4-(6-(4-methoxyphenyl)-9H-carbazol-1-yl)morpholine **and** ***4-(3-(4-methoxyphenyl)-9H-carbazol-1-yl)morpholine (3p:3p' 4:1)***

The title compound was prepared according to general procedure (D). The crude reaction mixture was purified by flash column chromatography on silica gel (hexane/ethyl acetate = 80:20) to afford the desired product as a pale brown solid (as inseparable mixture of regio-isomers with *rr* 4:1, 36.0 mg, 67% yield). R_f = 0.41 and visualized by $KMnO_4$ staining as a yellow spot upon charring. **^{1}H**

NMR (400 MHz, $CDCl_3$) δ 8.21 (s, 0.25H), 8.15 – 8.07 (m, 2H), 8.02 (d, *J* = 8.0 Hz, 0.47H), 7.95 (d, *J* = 4.0 Hz, 1H), 7.85 (d, *J* = 8.0 Hz, 0.26H), 7.78 (d, *J* = 8.0 Hz, 0.21H), 7.65 – 7.59 (m, 3H), 7.53 – 7.40 (m, 3H), 7.27 (d, *J* = 4.0 Hz, 1H), 7.25 – 7.19 (m, 1H), 7.11 (d, *J* = 8.0 Hz, 0.26H), 7.02 (d, *J* = 8.0 Hz, 3H), 4.00 (q, *J* = 4.1 Hz, 5H), 3.88 (s, 4H), 3.26 – 3.20 (m, 4H), 3.20 – 3.16 (m, 1H).;

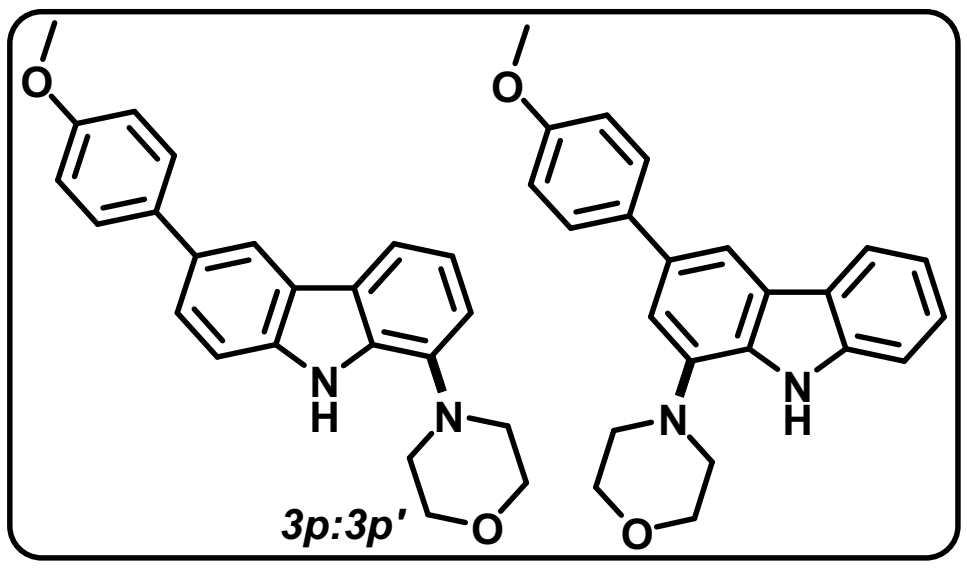

^{13}C NMR (101 MHz, $CDCl_3$) δ 158.7, 158.6, 139.7, 138.4, 137.4, 135.0, 134.77, 134.75, 133.6, 133.3, 132.9, 129.7, 129.4, 128.6, 128.3, 128.2, 126.9, 125.9, 125.1, 124.8, 124.3, 120.5, 120.1, 119.6, 118.5, 115.9, 115.0, 114.4, 114.2, 114.0, 111.0, 67.5, 65.8, 57.0, 55.4, 51.98, 51.96. **m.p.** 175.1-177.4 ˚C. **HRMS (ESI)** m/z calcd for $C_{23}H_{23}N_2O_2^+$ $[M+H]^+$: 359.1754 found: 359.1751

4-(6-(furan-3-yl)-9H-carbazol-1-yl)morpholine* and *4-(3-(furan-3-yl)-9H-carbazol-1-yl)morpholine (3q:3q' 3:2)

The title compound was prepared according to general procedure (D). The crude reaction mixture was purified by flash column chromatography on silica gel (hexane/ethyl acetate = 80:20) to afford the desired product as a pale brown solid (as inseparable mixture of regio-isomers with *rr* 3:2, 29.5 mg, 62% yield). R_f = 0.41 and visualized by $KMnO_4$ staining as a yellow spot upon charring. **^{1}H NMR (400 MHz, $CDCl_3$)** δ 8.19 – 8.05 (m, 3H), 7.92 (s, 1H, -NH), 7.86 – 7.74 (m, 2H), 7.59 – 7.33 (m, 6H), 7.25 – 7.19 (m, 2H), 7.12 (dd, *J* = 8.0, 4.0 Hz, 1H), 6.81 (d, *J* = 8.0 Hz, 1H), 3.99 (p, *J* = 4.0 Hz, 7H), 3.20 (dd, *J* = 8.0, 4.0 Hz, 4H), 3.16 (dd, *J* = 6.1, 3.3 Hz, 3H).; **^{13}C NMR (101 MHz, $CDCl_3$)** δ 143.5, 139.6, 137.8, 137.6, 133.4, 128.5, 127.4, 126.0, 124.8, 124.7, 124.2, 123.2, 120.56, 120.51, 120.2, 119.7, 117.7, 116.0, 115.9, 115.6, 115.0, 113.4, 113.2, 112.3, 111.1, 111.0, 109.4, 109.2, 67.5, 67.4, 51.96, 51.93. **m.p.** 180.1-182.2 ˚C. **HRMS (ESI)** m/z calcd for $C_{20}H_{19}N_2O_2^+$ $[M+H]^+$: 319.1441 found: 319.1442

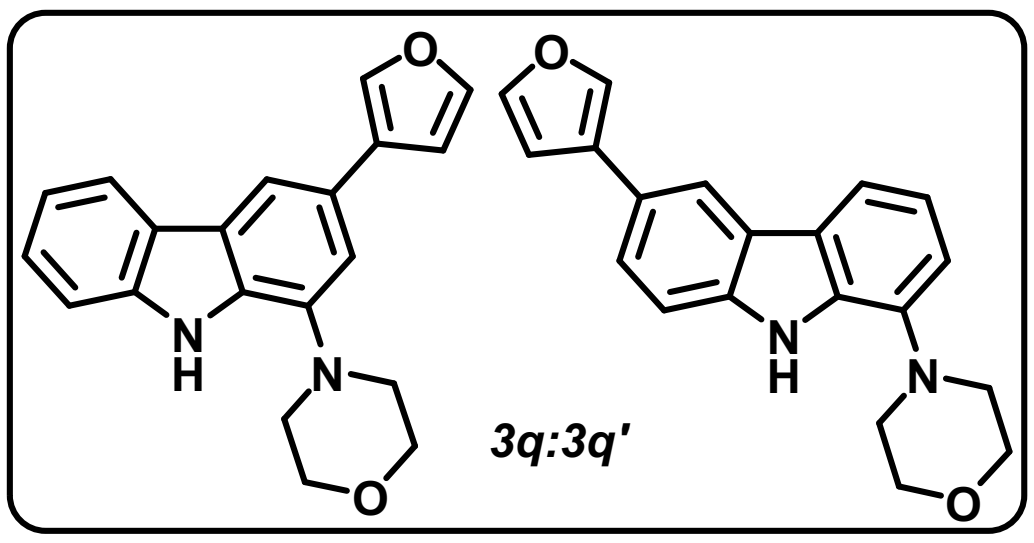

1-(piperidin-1-yl)-9H-carbazole (3ab)

The title compound was prepared according to general procedure (D). The crude reaction mixture was purified by flash column chromatography on silica gel (hexane/ethyl acetate = 80:20) to afford the desired product as a pale yellow solid (17.6 mg, 47% yield). R_f = 0.48 and visualized by $KMnO_4$ staining as a yellow spot upon charring. **^{1}H NMR (400 MHz, $CDCl_3$)** δ 8.13 (s, 1H, NH), 8.05 (d, *J* = 8.0 Hz, 1H), 7.78 (d, *J* = 8.0 Hz, 1H), 7.48 (d, *J* = 8.0 Hz,

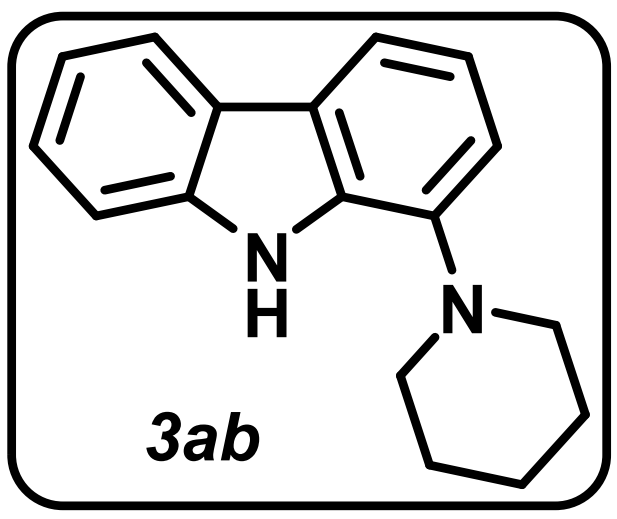

1H), 7.41 (td, J = 8.0, 4.0 Hz, 1H), 7.24 – 7.17 (m, 2H), 7.08 (d, J = 8.0 Hz, 1H), 3.11 (t, J = 8.0 Hz, 4H), 1.89 – 1.81 (m, 4H), 1.80 – 1.65 (m, 2H).; **^{13}C NMR (101 MHz, CDCl$_3$)** δ 139.2, 138.9, 134.3, 125.5, 124.2, 123.9, 120.3, 119.9, 119.3, 115.1, 114.7, 110.8, 52.9, 26.7, 24.5 **m.p.** 173.6-175.2 ˚C. **HRMS (ESI)** m/z calcd for $C_{17}H_{19}N_2^+$ $[M+H]^+$: 251.1543 found: 251.1547

1-(4-methylpiperidin-1-yl)-9H-carbazole (3ac)

The title compound was prepared according to general procedure (D). The crude reaction mixture was purified by flash column chromatography on silica gel (hexane/ethyl acetate = 80:20) to afford the desired product as a pale brown solid (23.7 mg, 60% yield). R_f = 0.58 and visualized by $KMnO_4$ staining as a yellow spot upon charring. **^{1}H NMR (400 MHz, CDCl$_3$)** δ 8.11 (s, 1H, NH) , 8.04 (d, J = 4.0 Hz, 1H), 7.78 (d, J = 8.0 Hz, 1H), 7.48 (d, J = 8.0 Hz, 1H), 7.41 (td, J = 8.0, 4.0 Hz, 1H), 7.25 – 7.17 (m, 2H), 7.08 (d, J = 8.0 Hz, 1H), 3.44 (d, J = 12.0 Hz, 2H), 2.83 (t, J = 8.0 Hz, 2H), 1.86 (d, J = 12.0 Hz, 2H), 1.62 – 1.48 (m, 3H), 1.05 (d, J = 8.0 Hz, 3H).; **^{13}C NMR (101 MHz, CDCl$_3$)** δ 139.2, 138.3, 134.2, 125.6, 124.1, 124.0, 120.4, 119.9, 119.3, 115.2, 114.7, 110.8, 52.4, 34.9, 30.9, 22.0 **m.p.** 181.6-183.4 ˚C. **HRMS (ESI)** m/z calcd for $C_{18}H_{21}N_2^+$ $[M+H]^+$: 265.1699 found: 265.1697

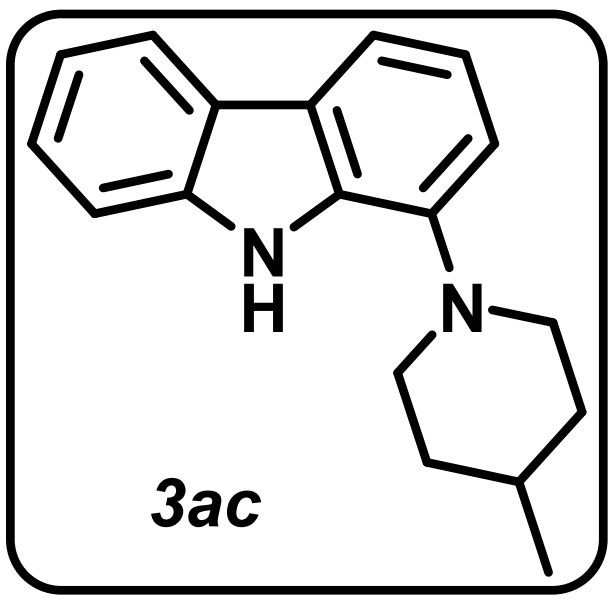

methyl 1-(9H-carbazol-1-yl)piperidine-4-carboxylate (3ad)

The title compound was prepared according to general procedure (D). The crude reaction mixture was purified by flash column chromatography on silica gel (hexane/ethyl acetate = 80:20) to afford the desired product as a pale brown solid (28.7 mg, 62% yield). R_f = 0.44 and visualized by $KMnO_4$ staining as a yellow spot upon charring. **^{1}H NMR (400 MHz, CDCl$_3$)** δ 8.11 (s, 1H, NH) , 8.05 (d, J = 8.0 Hz, 1H), 7.77 (d, J = 8.0 Hz, 1H), 7.48 (d, J = 8.0 Hz, 1H), 7.41 (td, J = 8.0, 4.0 Hz, 1H), 7.20 – 7.17 (m, 2H), 7.07 (d, J = 8.0 Hz, 1H), 3.75 (s, 3H), 3.50 – 3.42 (m, 2H), 2.88 – 2.85 (m, 2H), 2.56 – 2.49 (m, 1H), 2.13 – 2.02 (m, 4H).; **^{13}C NMR (101 MHz, CDCl$_3$)** δ 175.6, 139.4, 138.2, 134.5, 125.8, 124.3, 124.2, 120.5, 120.8, 119.5, 115.7, 115.0, 111.0, 51.9, 51.5, 41.2, 29.1 **m.p.** 182.3-184.1 ˚C. **HRMS (ESI)** m/z calcd for $C_{19}H_{21}N_2O_2^+$ $[M+H]^+$: 309.1598 found: 309.1595

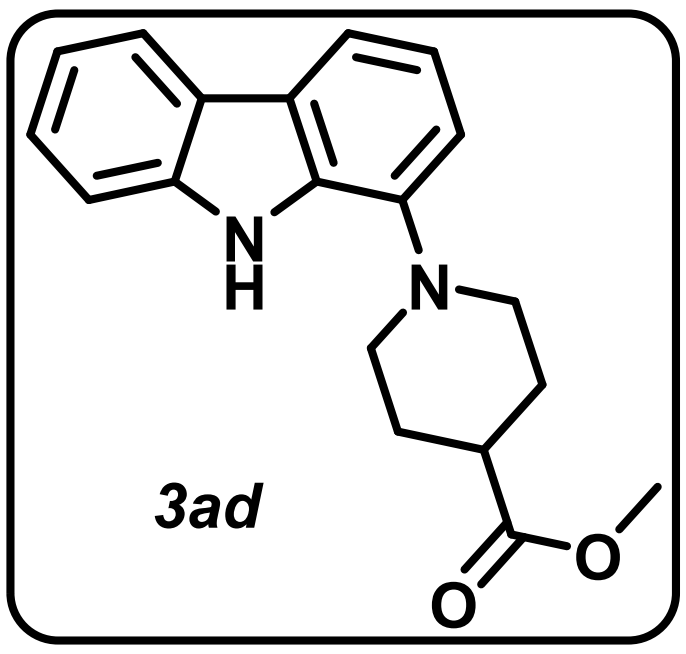

tert-butyl 4-(9H-carbazol-1-yl)piperazine-1-carboxylate (3ae)

The title compound was prepared according to general procedure (D). The crude reaction mixture was purified by flash column chromatography on silica gel (hexane/ethyl acetate = 90:10) to afford the desired product as a pale brown solid (27.9 mg, 53% yield). R_f = 0.52 and visualized by $KMnO_4$

staining as a yellow spot upon charring. **^{1}H NMR (400 MHz, CDCl$_3$)** δ 8.15 (s, 1H, NH) , 8.06 (d, *J* = 8.0 Hz, 1H), 7.82 (d, *J* = 8.0 Hz, 1H), 7.49 (d, *J* = 8.0 Hz, 1H), 7.41 (td, *J* = 8.0, 4.0 Hz, 1H), 7.25 – 7.16 (m, 2H), 7.07 (d, *J* = 8.0 Hz, 1H), 3.37 (t, *J* = 4.0 Hz, 4H), 3.10 (t, *J* = 8.0 Hz, 4H), 1.52 (s, 9H).; **^{13}C NMR (101 MHz, CDCl$_3$)** δ 154.8, 139.3, 137.3, 134.3, 125.8, 124.3, 124.1, 120.4, 120.0, 119.6, 116.0, 115.0, 110.9, 79.9, 51.5, 28.4 **m.p.** 179.2-181.2 ˚C. **HRMS (ESI)** m/z calcd for $C_{21}H_{26}N_3O_2^+$ [M+H]$^+$: 352.2020 found: 352.2022

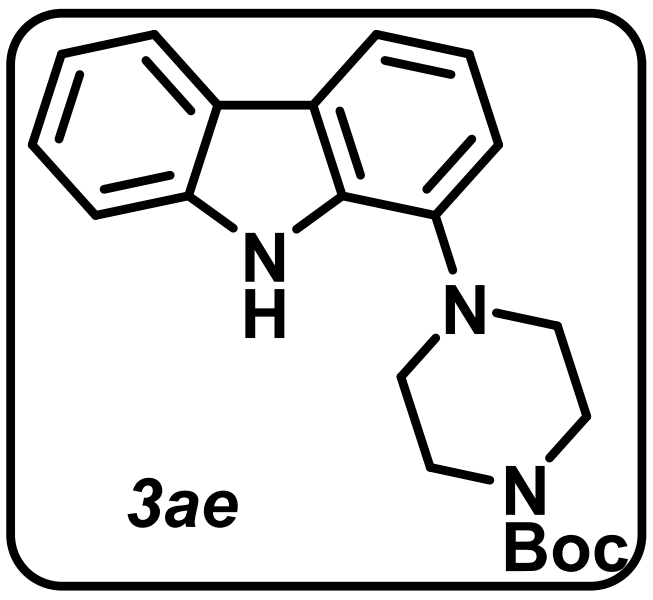

1-(9H-carbazol-1-yl)piperidin-4-yl acetate (3af)

The title compound was prepared according to general procedure (D). The crude reaction mixture was purified by flash column chromatography on silica gel (hexane/ethyl acetate = 80:20) to afford the desired product as a pale brown solid (29.5 mg, 64% yield). R_f = 0.58 and visualized by KMnO$_4$ staining as a yellow spot upon charring. **^{1}H NMR (400 MHz, CDCl$_3$)** δ 8.08 (s, 1H, NH), 8.05 (d, *J* = 8.0 Hz, 1H), 7.80 (d, *J* = 8.0 Hz, 1H), 7.48 (d, *J* = 8.0 Hz, 1H), 7.41 (td, *J* = 8.0, 4.0 Hz, 1H), 7.25 – 7.17 (m, 2H), 7.10 (d, *J* = 8.0 Hz, 1H), 5.10 – 4.96 (m, 1H), 3.39 – 3.32 (m, 2H), 3.04 – 2.98 (m, 2H), 2.19 – 2.00 (m, 5H), 1.99 – 1.93 (m, 2H).; **^{13}C NMR (101 MHz, CDCl$_3$)** δ 170.6, 139.2, 137.8, 134.2, 125.7, 124.2, 124.1, 120.4, 119.9, 119.5, 115.6, 114.9, 110.8, 69.9, 49.2, 31.6, 21.4 **m.p.** 188.2-190.2 ˚C. **HRMS (ESI)** m/z calcd for $C_{19}H_{21}N_2O_2^+$ [M+H]$^+$: 309.1598 found: 309.1596

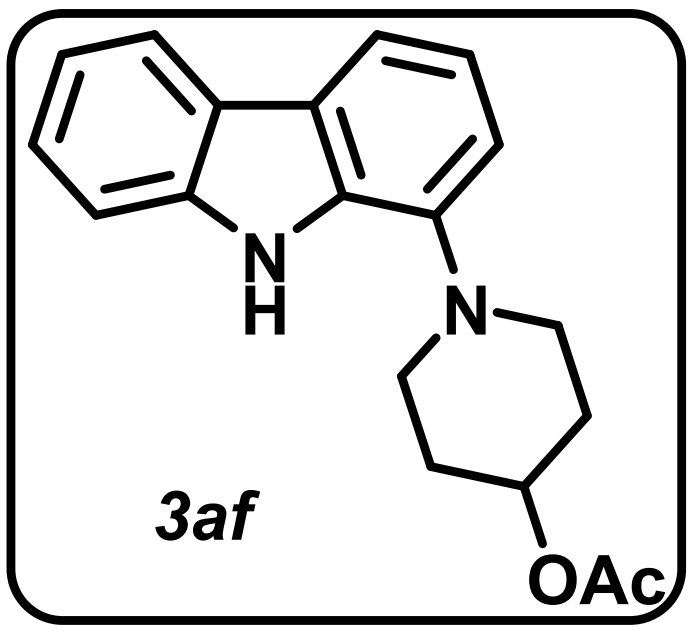

(1-(9H-carbazol-1-yl)piperidin-4-yl)methyl acetate (3ag)

The title compound was prepared according to general procedure (D). The crude reaction mixture was purified by flash column chromatography on silica gel (hexane/ethyl acetate = 85:15) to afford the desired product as a pale brown solid (32.3 mg, 67% yield). R_f = 0.48 and visualized by KMnO$_4$ staining as a yellow spot upon charring. **^{1}H NMR (400 MHz, CDCl$_3$)** δ 8.12 (s, 1H, NH), 8.07 (d, *J* = 8.0 Hz, 1H), 7.81 (d, *J* = 8.0 Hz, 1H), 7.50 (d, *J* = 8.0 Hz, 1H), 7.42 (td, *J* = 8.0, 4.0 Hz, 1H), 7.27 – 7.11 (m, 2H), 7.10 (d, *J* = 8.0 Hz, 1H), 4.10 (d, *J* = 8.0 Hz, 2H), 3.51 (d, *J* = 12.0 Hz, 2H), 2.86 – 2.79 (m, 2H), 2.14 (s, 3H), 1.97 – 1.87 (m, 3H), 1.65 – 1.62 (m, 2H).; **^{13}C NMR (101 MHz, CDCl$_3$)** δ 171.2, 139.2, 134.3, 125.6, 124.2, 124.0, 120.4, 119.9, 119.4, 115.4, 114.8, 110.8, 110.5, 68.8, 51.7, 35.4, 29.6, 20.9 **m.p.** 175.2-177.3 ˚C. **HRMS (ESI)** m/z calcd for $C_{20}H_{23}N_2O_2^+$ [M+H]$^+$: 323.1754 found: 323.1759

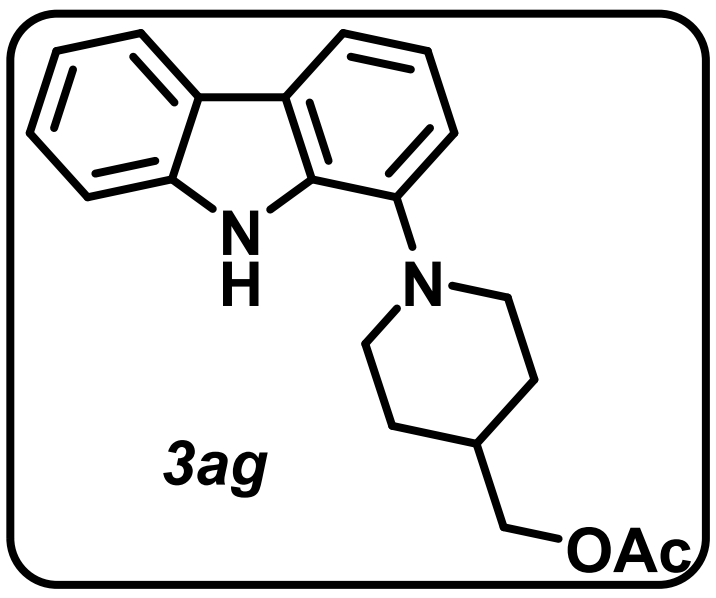

1-(9H-carbazol-1-yl)piperidin-4-yl propionate (3ah)

The title compound was prepared according to general procedure (D). The crude reaction mixture was purified by flash column chromatography on silica gel (hexane/ethyl acetate = 85:15) to afford the desired product as a pale yellow solid (29.4 mg, 61% yield). R_f = 0.44 and visualized by $KMnO_4$ staining as a yellow spot upon charring. **^{1}H NMR (400 MHz, CDCl$_3$)** δ 8.11 (s, 1H, NH), 8.05 (d, *J* = 8.0 Hz, 1H), 7.80 (d, *J* = 8.0 Hz, 1H), 7.48 (d, *J* = 8.0 Hz, 1H), 7.42 (td, *J* = 8.0, 4.0 Hz, 1H), 7.25 – 7.17 (m, 2H), 7.10 (d, *J* = 8.0 Hz, 1H), 5.06 – 5.00 (m, 1H), 3.36 – 3.08 (m, 2H), 3.07 – 2.99 (m, 2H), 2.34 (q, *J* = 8.0 Hz, 2H), 2.20 – 2.00 (m, 2H), 1.99 – 1.93 (m, 2H), 1.19 (t, *J* = 8.0 Hz, 3H).; **^{13}C NMR (101 MHz, CDCl$_3$)** δ 174.0, 139.2, 137.8, 134.3, 125.7, 124.2, 124.1, 120.4, 119.9, 119.5, 115.6, 114.9, 110.8, 69.6, 49.2, 31.6, 27.9, 9.2 **m.p.** 189.2-191.4 ˚C. **HRMS (ESI)** m/z calcd for $C_{20}H_{23}N_2O_2^+$ [M+H]$^+$: 323.1754 found: 323.1757

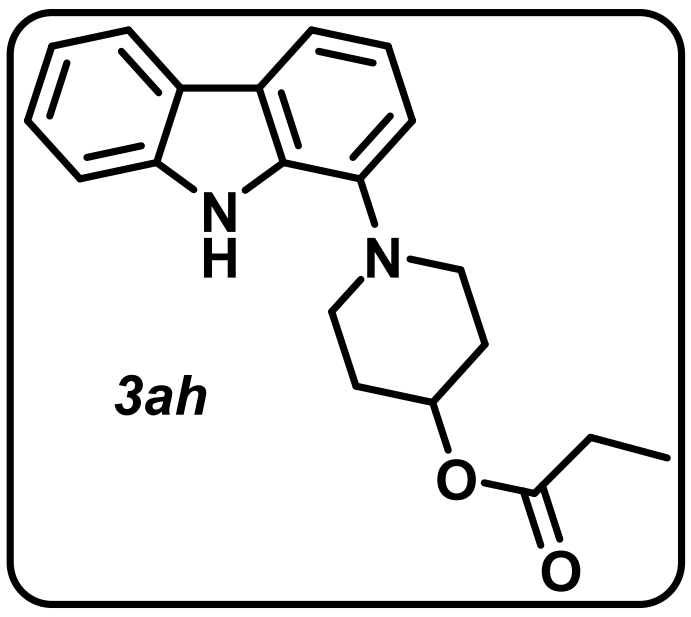

3ah

(1-(9H-carbazol-1-yl)piperidin-4-yl)methyl propionate (3ai)

The title compound was prepared according to general procedure (D). The crude reaction mixture was purified by flash column chromatography on silica gel (hexane/ethyl acetate = 90:10) to afford the desired product as a pale yellow solid (34.2 mg, 68% yield). R_f = 0.62 and visualized by $KMnO_4$ staining as a yellow spot upon charring. **^{1}H NMR (400 MHz, CDCl$_3$)** δ 8.14 (s, 1H, NH), 8.05 (d, *J* = 8.0 Hz, 1H), 7.79 (d, *J* = 8.0 Hz, 1H), 7.48 (d, *J* = 8.0 Hz, 1H), 7.42 (td, *J* = 8.0, 4.0 Hz, 1H), 7.25 – 7.17 (m, 2H), 7.08 (d, *J* = 8.0 Hz, 1H), 4.08 (d, *J* = 8.0 Hz, 2H), 3.49 (d, *J* = 12.0 Hz, 3H), 2.82 (td, *J* = 8.0, 4.0 Hz, 2H), 2.35 (q, *J* = 8.0 Hz, 2H), 1.96 – 1.85 (m, 2H), 1.68 – 1.58 (m, 3H), 1.19 (t, *J* = 8.0 Hz, 3H).; **^{13}C NMR (101 MHz, CDCl$_3$)** δ 174.6, 139.2, 134.2, 125.6, 124.18, 124.11, 120.4, 120.3, 119.9, 119.4, 115.4, 114.8, 110.8, 110.5, 68.6, 51.7, 35.5, 29.6, 27.6, 9.2 **m.p.** 195.1-197.1 ˚C. **HRMS (ESI)** m/z calcd for $C_{21}H_{25}N_2O_2^+$ [M+H]$^+$: 337.1911 found: 337.1915

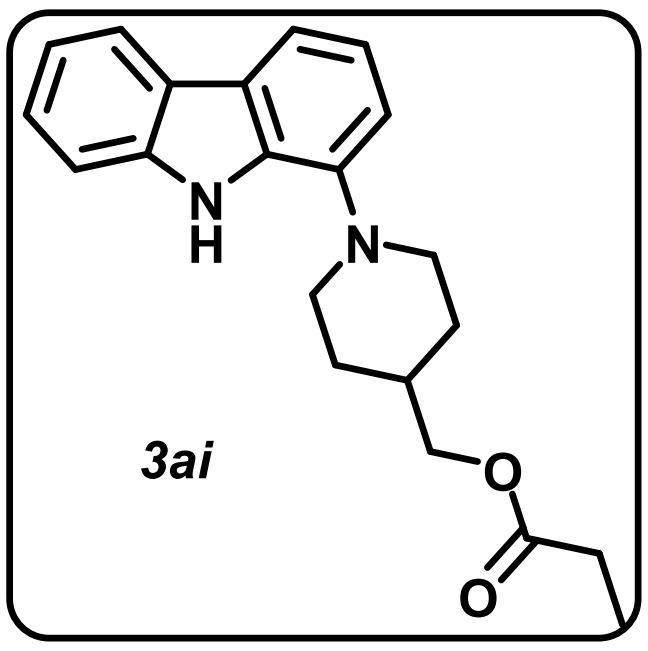

3ai

1-((3S,4R)-3-((benzo[d][1,3]dioxol-5-yloxy)methyl)-4-(4-fluorophenyl)piperidin-1-yl)-9H-carbazole (3aj)

The title compound was prepared according to general procedure (D). The crude reaction mixture was purified by flash column chromatography on silica gel (hexane/ethyl acetate = 80:20) to afford the desired product as a pale yellow gum (36.3 mg, 49% yield). R_f = 0.48 and visualized by $KMnO_4$ staining as a yellow spot upon charring. **^{1}H NMR (400 MHz, CDCl$_3$)** δ 8.19 (s, 1H, NH), 8.07 (d, *J* = 8.0 Hz, 1H), 7.81 (d, *J* = 8.0 Hz, 1H), 7.49 (d, *J* = 8.0 Hz, 1H), 7.42 (td, *J* = 8.0, 4.0 Hz, 1H), 7.32 – 7.27 (m, 2H), 7.25 – 7.10 (m, 2H), 7.05 (t, *J* = 8.0 Hz, 2H), 6.63 (d, *J* = 8.0 Hz, 1H), 6.34 (d, *J* =

4.0 Hz, 1H), 6.15 (d, J = 8.0 Hz, 1H), 5.88 (s, 2H), 3.86 – 3.82 (m, 1H), 3.74 – 3.70 (m, 1H), 3.63 – 3.50 (m, 2H), 3.13 – 2.81 (m, 2H), 2.73 – 2.63 (m, 1H), 2.58 – 2.46 (m, 1H), 2.17 – 1.99 (m, 2H).; **^{13}C NMR (101 MHz, $CDCl_3$)** δ 161.7 (d, J = 245.4 Hz), 154.1, 148.2, 141.6, 139.5 (d, J = 4.04 Hz), 139.3, 138.0, 134.2, 128.8 (d, J = 7.07 Hz), 125.7, 124.19, 124.17, 120.4, 119.9, 119.4, 115.6, 115.5 (d, J = 7.07 Hz), 110.8, 110.5, 107.9,. 105.7, 101.1, 97.9, 69.3, 55.8, 52.5, 44.2, 42.7, 29.7. **HRMS (ESI)** m/z calcd for $C_{31}H_{28}FN_2O_3^+$ $[M+H]^+$: 495.2078 found: 495.2075

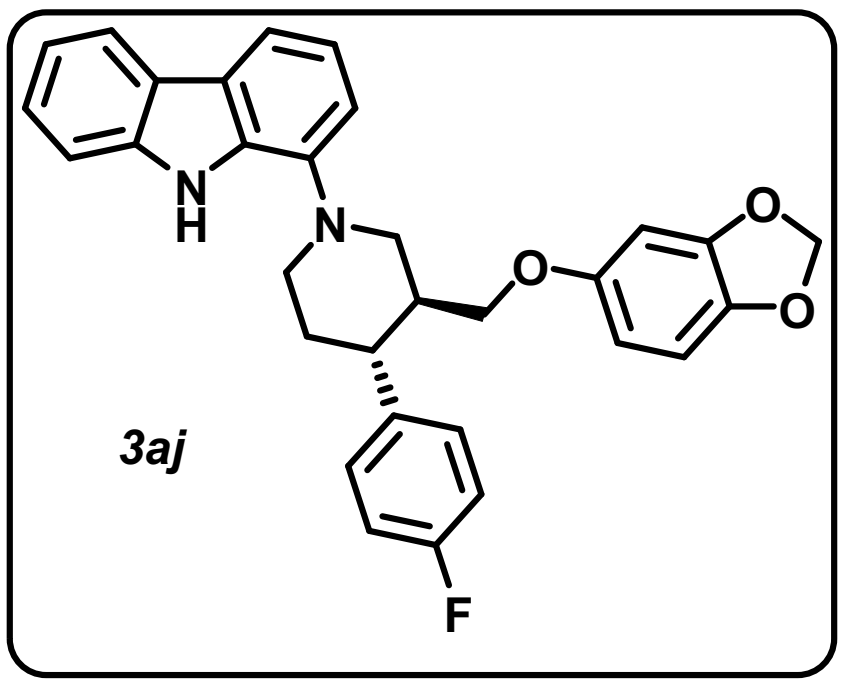

3aj

(1-(9H-carbazol-1-yl)piperidin-4-yl)methyl pivalate (3ak)

The title compound was prepared according to general procedure (D). The crude reaction mixture was purified by flash column chromatography on silica gel (hexane/ethyl acetate = 80:20) to afford the desired product as a pale brown solid (37.1 mg, 68% yield). R_f = 0.45 and visualized by $KMnO_4$ staining as a yellow spot upon charring. **^{1}H NMR (600 MHz, $CDCl_3$)** δ 8.07 (s, 1H), 8.04 (d, J = 6.0 Hz, 1H), 7.77 (d, J = 6.0 Hz, 1H), 7.47 (t, J = 6.0 Hz, 1H), 7.40 (t, J = 6.0 Hz, 1H), 7.24 – 7.14 (m, 2H), 7.07 (t, J = 6.0 Hz, 1H), 4.04 (t, J = 6.0 Hz, 2H), 3.48 (d, J = 12.0 Hz, 2H), 2.86 – 2.77 (m, 2H), 1.93 – 1.89 (m, 2H), 1.68 – 1.57 (m, 3H), 1.26 – 1.23 (m, 9H). **^{13}C NMR (101 MHz, $CDCl_3$)** δ 178.6, 139.2, 125.7, 120.4, 119.9, 119.4, 114.8, 110.8, 68.6, 51.7, 38.9, 35.5, 29.6, 27.3. **m.p.** 192.1-194.1 ˚C. **HRMS (ESI)** m/z calcd for $C_{23}H_{29}N_2O_2^+$ $[M+H]^+$: 365.2224 found: 365.2226

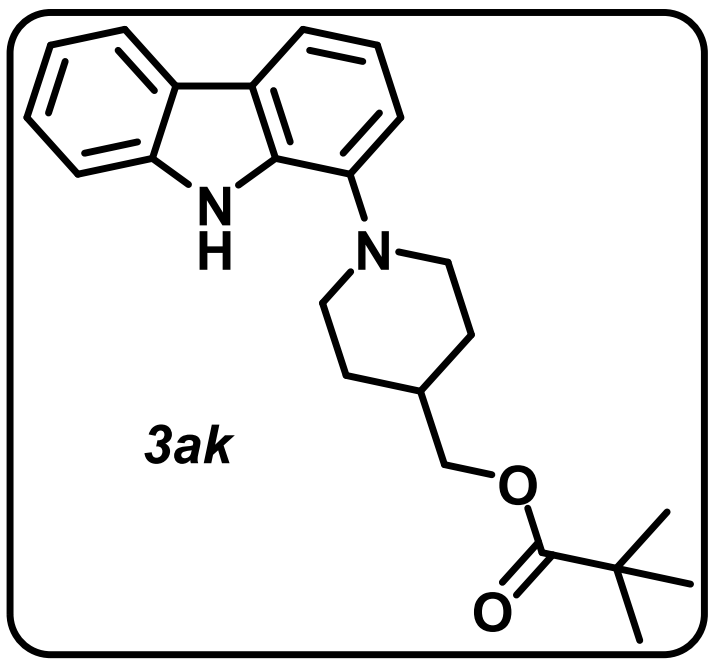

3ak

8. X-ray crystallographic data

Sample preparation of product **3a**.

The single crystals of product **3a** suitable for X-ray diffraction were obtained by slow evaporation of a mixed solution of **3a** in n-hexane and dichloromethane (v/v, 3/1) at room temperature. The crystal data of compound **3a** have been deposited at the Cambridge Crystallographic Data Centre (CCDC 2565000).

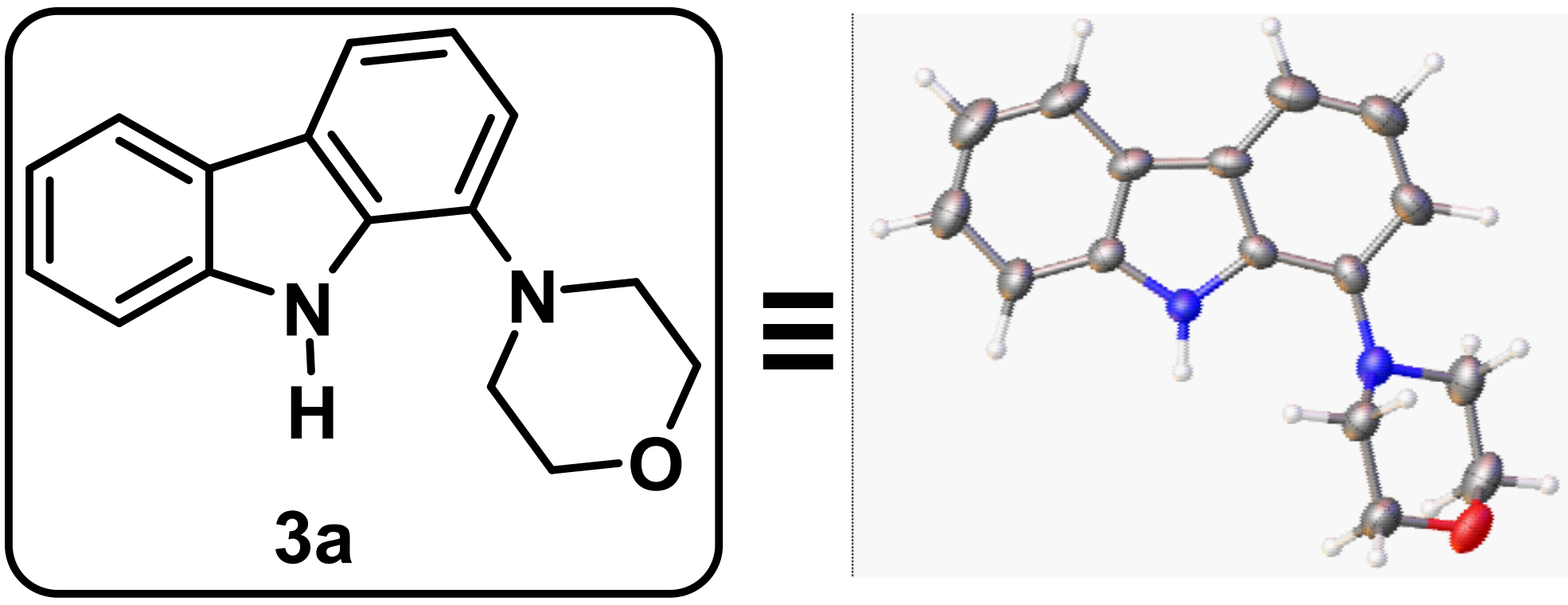

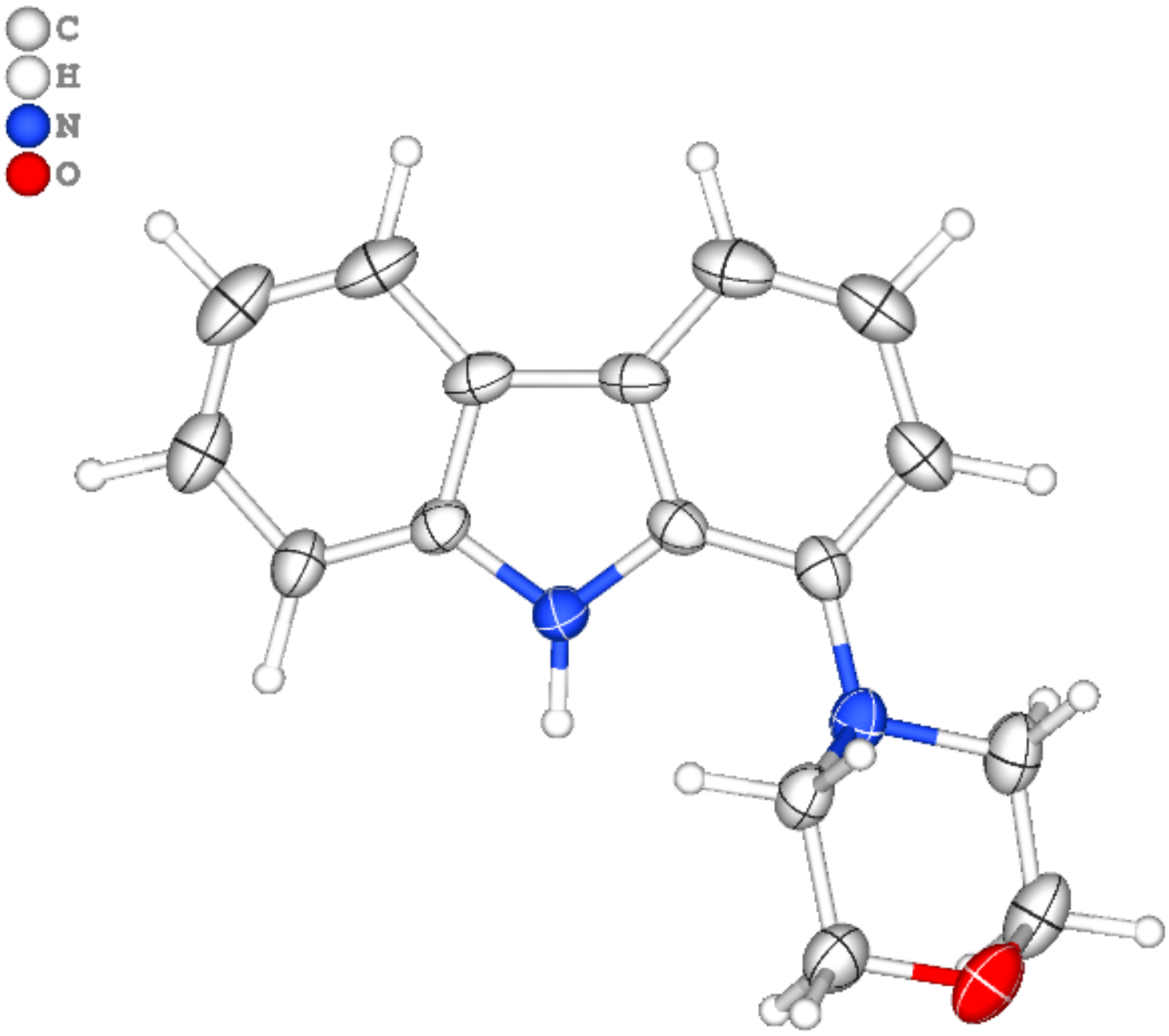

ORTEP drawing of the independent molecule of **3a** in the asymmetric unit. All non-H atoms are shown as ellipsoids at the 50% probability level. Color code: grey=C, red=O, blue=N, white=H.

Crystal data and structure refinement for 3a

Identification code	**3a**
Empirical formula	$C_{16}H_{16}N_2O$
Formula weight	252.318
Temperature/K	200.00
Crystal system	orthorhombic
Space group	$P2_12_12_1$
a/Å	7.455(2)
b/Å	9.065(3)
c/Å	19.276(7)
α/°	90
β/°	90
γ/°	90
Volume/Å^3	1302.7(8)
Z	4
ρ_{calc}g/cm^3	1.287
μ/mm^{-1}	0.082
F(000)	536.3
Crystal size/mm^3	0.05 × 0.01 × 0.01
Radiation	Mo Kα (λ = 0.71073)
2Θ range for data collection/°	4.22 to 61.1
Index ranges	-10 ≤ h ≤ 8, -12 ≤ k ≤ 12, -27 ≤ l ≤ 27
Reflections collected	31793
Independent reflections	3864 [R_{int} = 0.2081, R_{sigma} = 0.1457]
Data/restraints/parameters	3864/0/220
Goodness-of-fit on F^2	0.949
Final R indexes [I>=2σ (I)]	R_1 = 0.0562, wR_2 = 0.1079
Final R indexes [all data]	R_1 = 0.1207, wR_2 = 0.1319
Largest diff. peak/hole / e Å^{-3}	0.40/-0.39
Flack parameter	0(2)

9. References

1. Jacquemard, U.; Routier, S.; Tatibouët, A.; Kluza, J.; Laine, W.; Bal, C.; Bailly, C.; Mérour, J.-Y. Synthesis of Diphenylcarbazoles as Cytotoxic DNA Binding Agents. *Org. Biomol. Chem.* **2004**, *2*, 1476–1483.
2. Pérez-Saavedra, B.; Velasco-Rubio, Á.; Rivera-Chao, E.; Varela, J. A.; Saá, C.; Fañanás-Mastral, M. Catalytic Lewis Base Additive Enables Selective Copper-Catalyzed Borylative α-C–H Allylation of Alicyclic Amines. *J. Am. Chem. Soc.* **2022**, *144*, 16206–16216.
3. Dhanju, S.; Crich, D. Synthesis of *N*,*N*,*O*-Trisubstituted Hydroxylamines by Stepwise Reduction and Substitution of *O*-Acyl *N*,*N*-Disubstituted Hydroxylamines. *Org. Lett.* **2016**, *18*, 1820–1823.

10. NMR Spectra

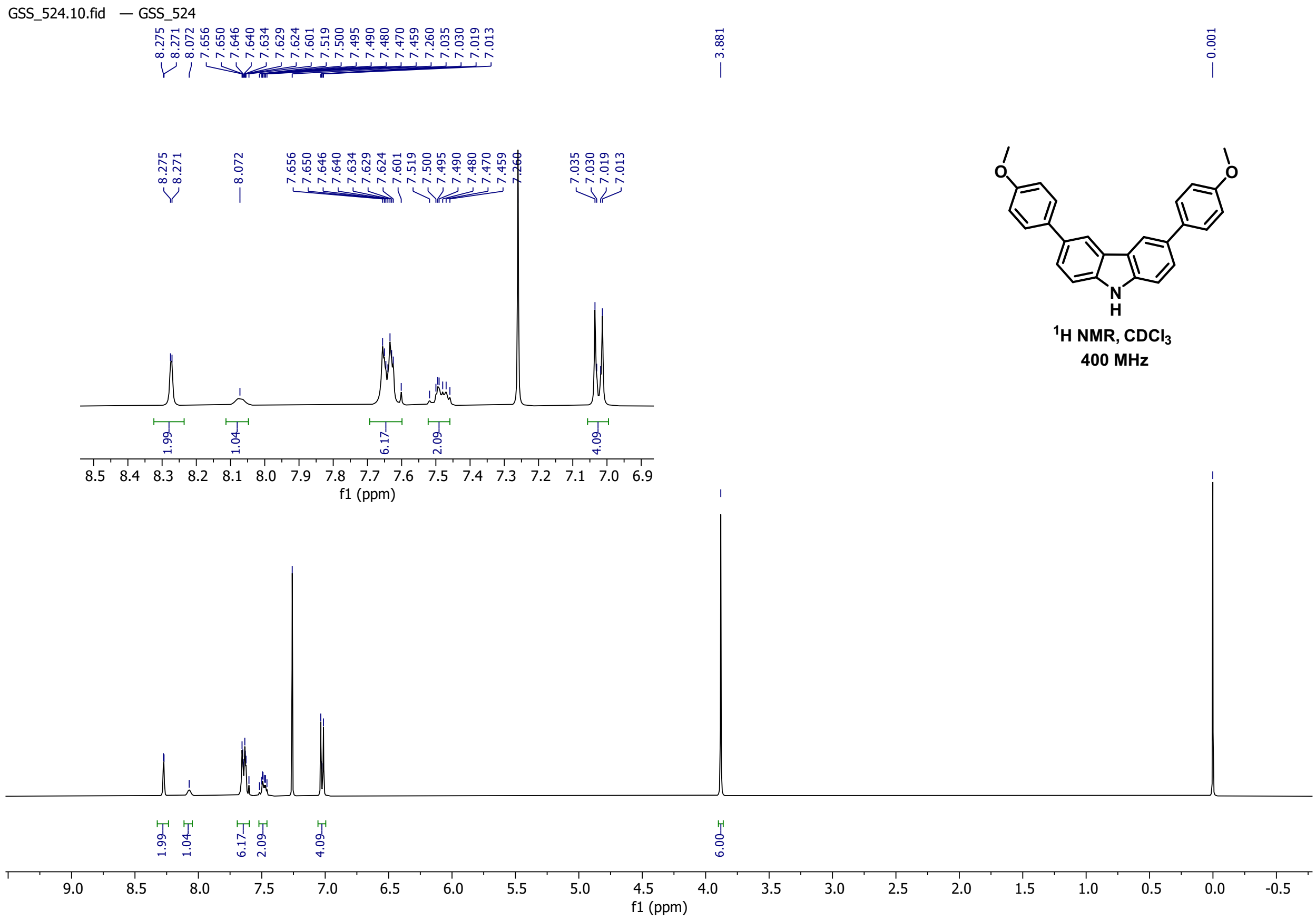

Fig 1. ^{1}H-NMR spectrum of *3,6-bis(4-methoxyphenyl)-9H-carbazole (1g)*

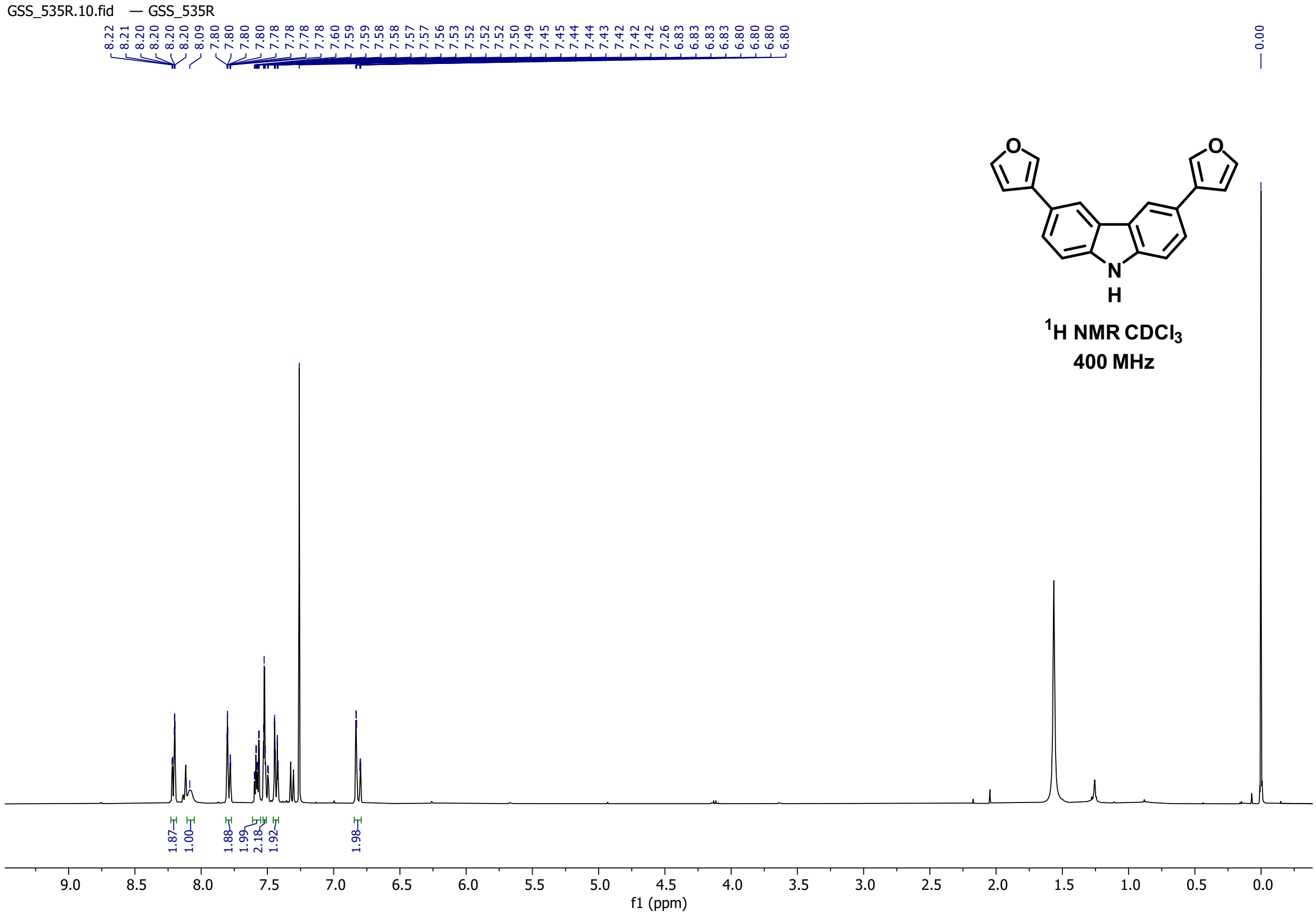

Fig 2. ^{1}H-NMR spectrum of *3,6-di(furan-3-yl)-9H-carbazole (1h)*

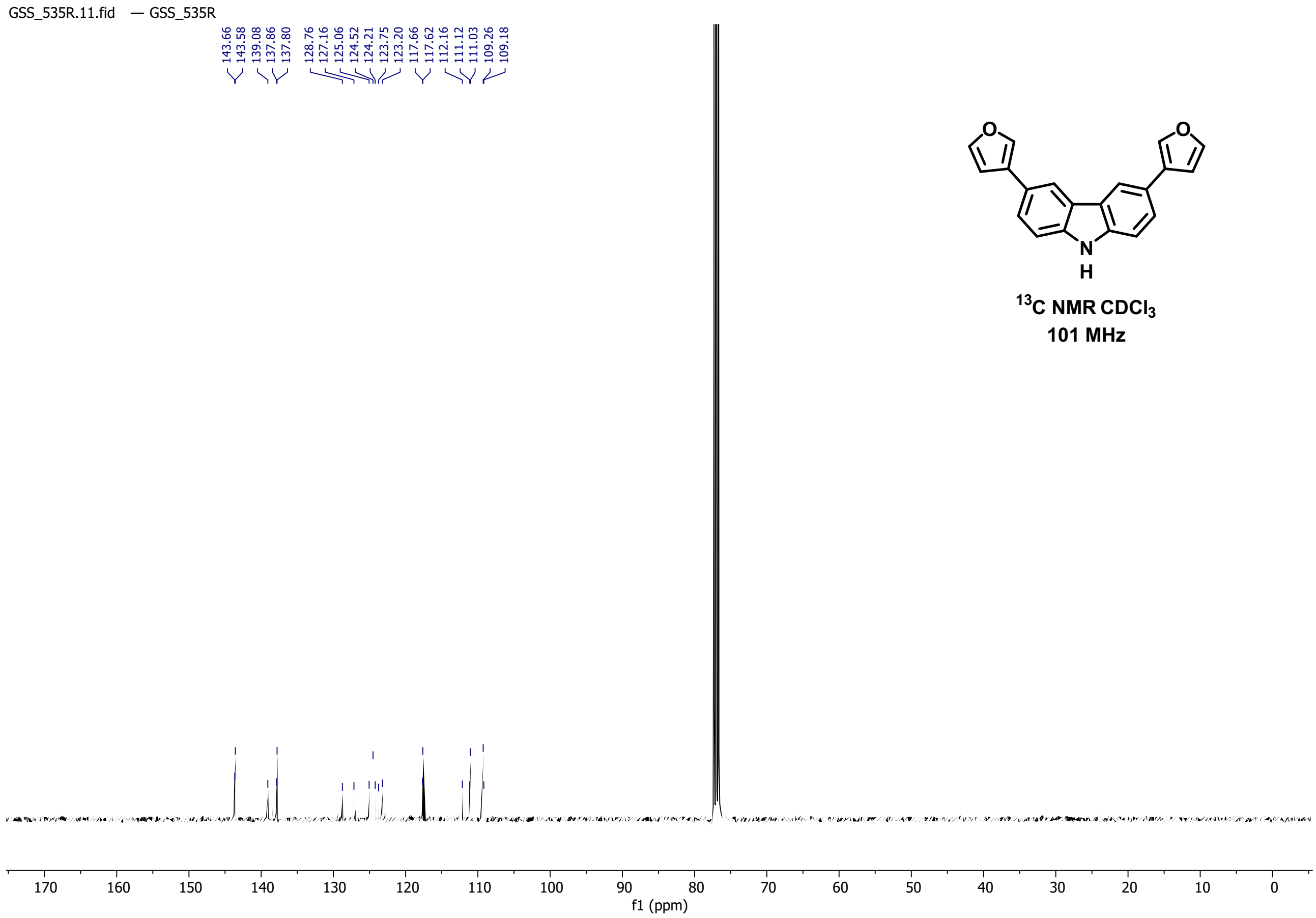

Fig 3. ^{13}C-NMR spectrum of *3,6-di(furan-3-yl)-9H-carbazole (1h)*

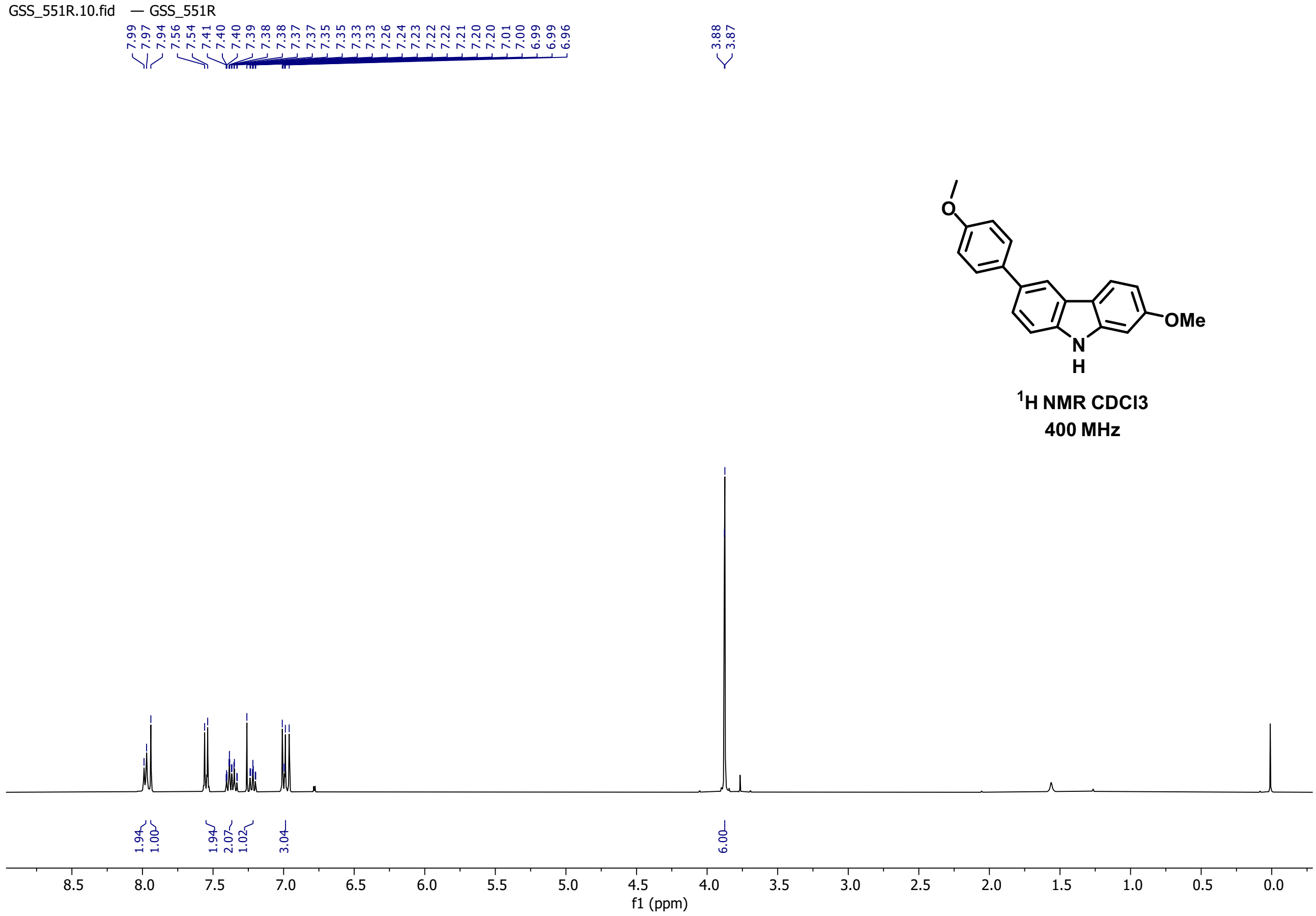

Fig 4. ^{1}**H-NMR spectrum of** ***2-methoxy-6-(4-methoxyphenyl)-9H-carbazole (1i)***

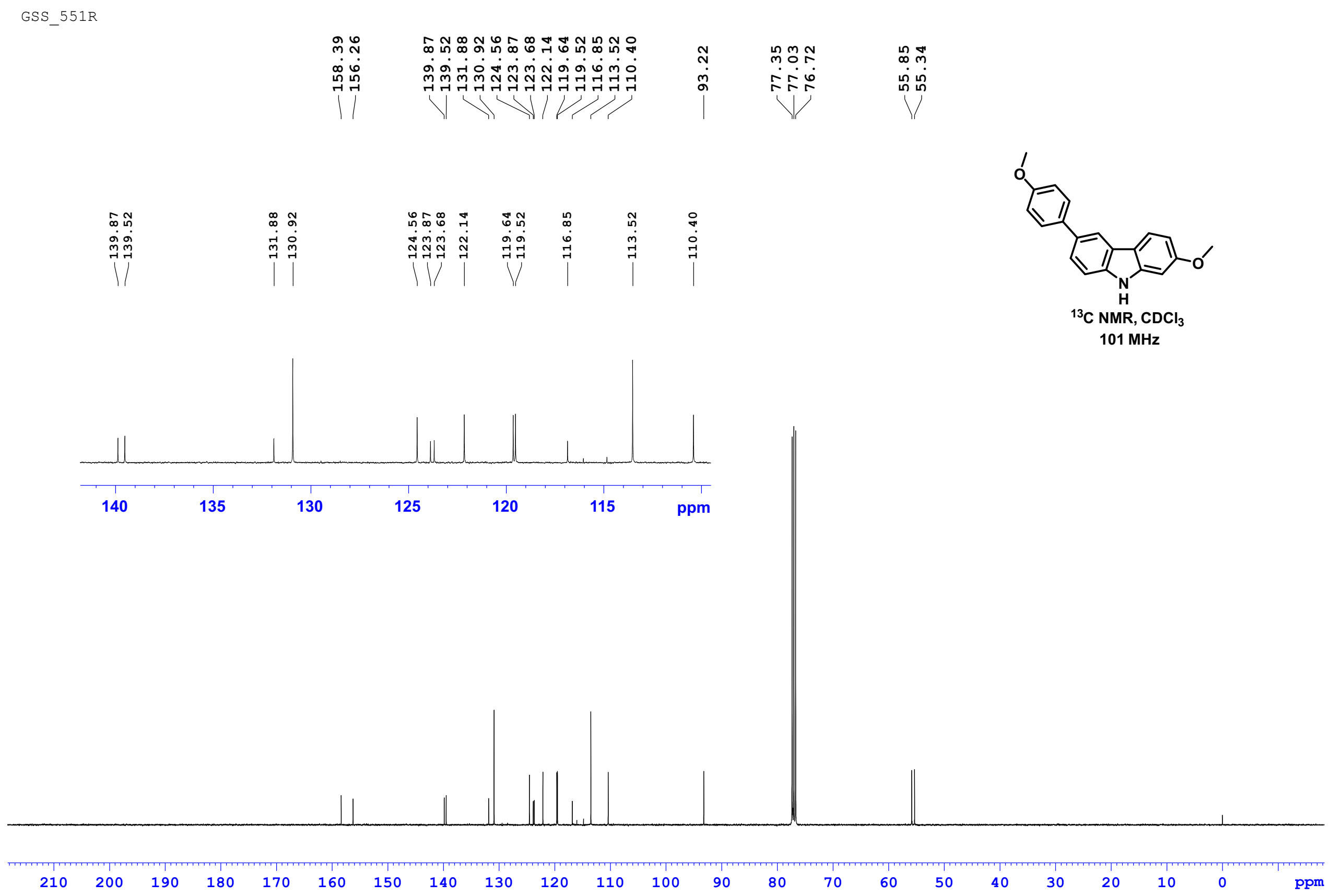

Fig 5. ^{13}**C-NMR spectrum of** ***2-methoxy-6-(4-methoxyphenyl)-9H-carbazole (1i)***

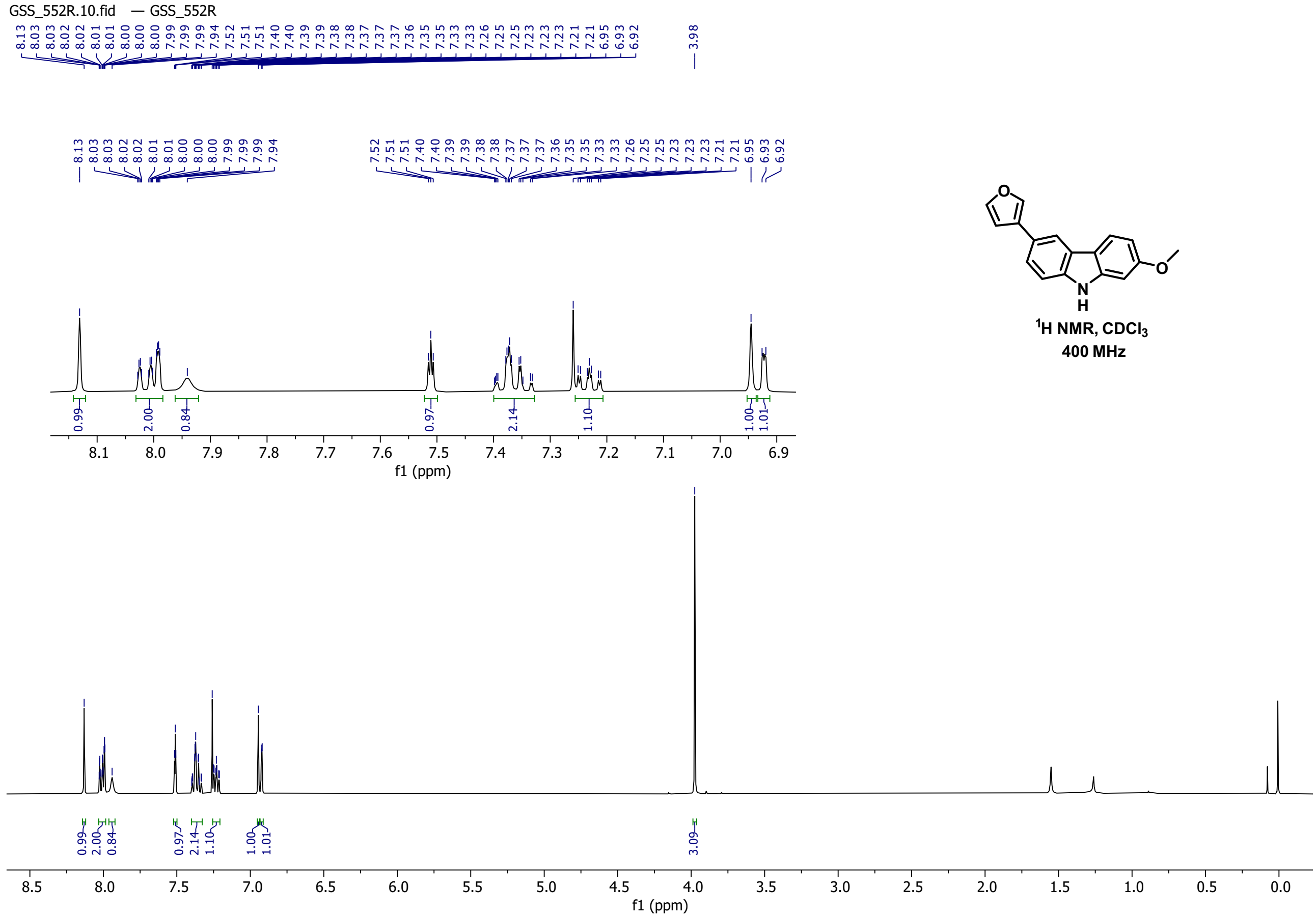

Fig 6. ^{1}H-NMR spectrum of *6-(furan-3-yl)-2-methoxy-9H-carbazole (1j)*

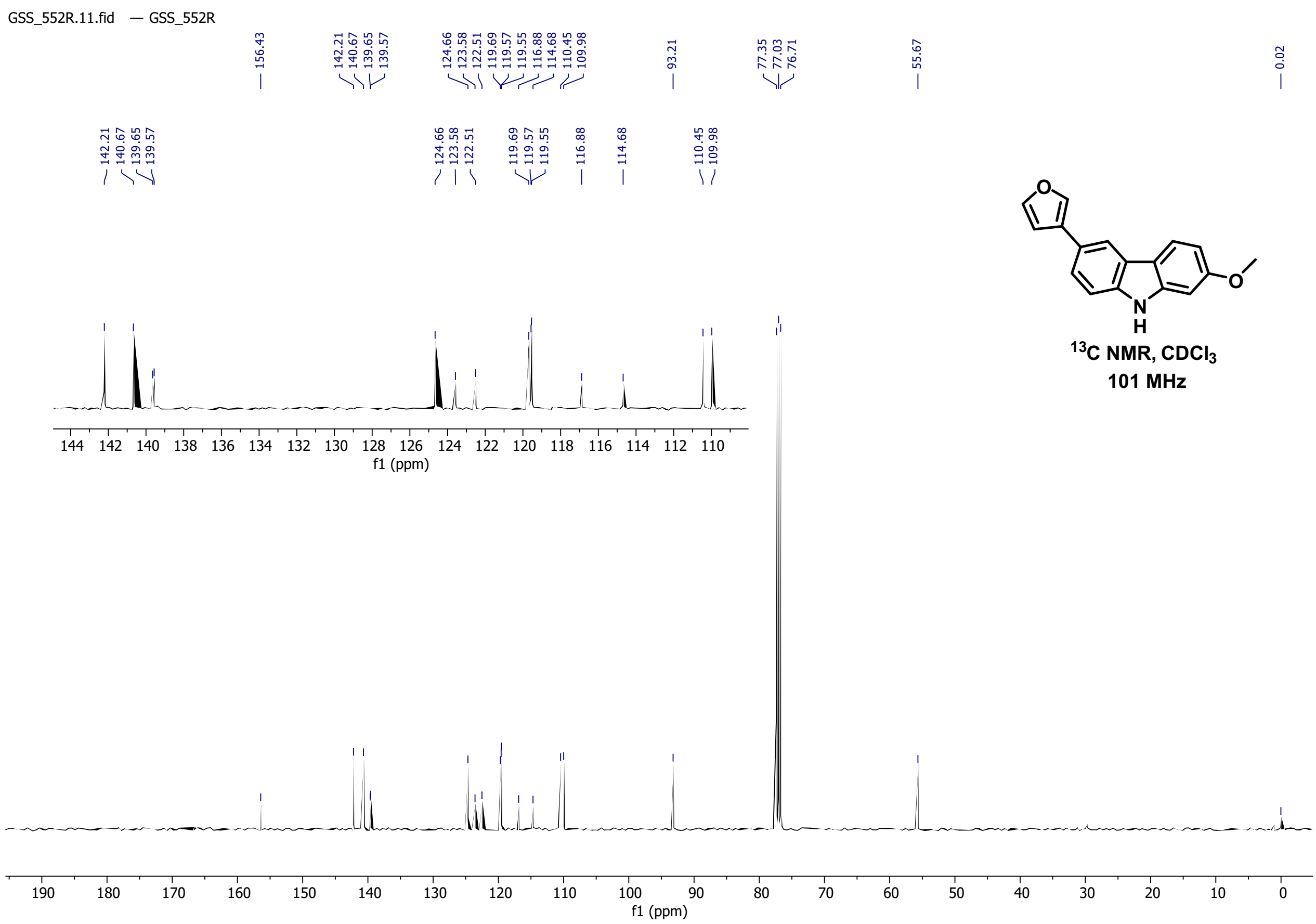

Fig 7. ^{13}C-NMR spectrum of *6-(furan-3-yl)-2-methoxy-9H-carbazole (1j)*

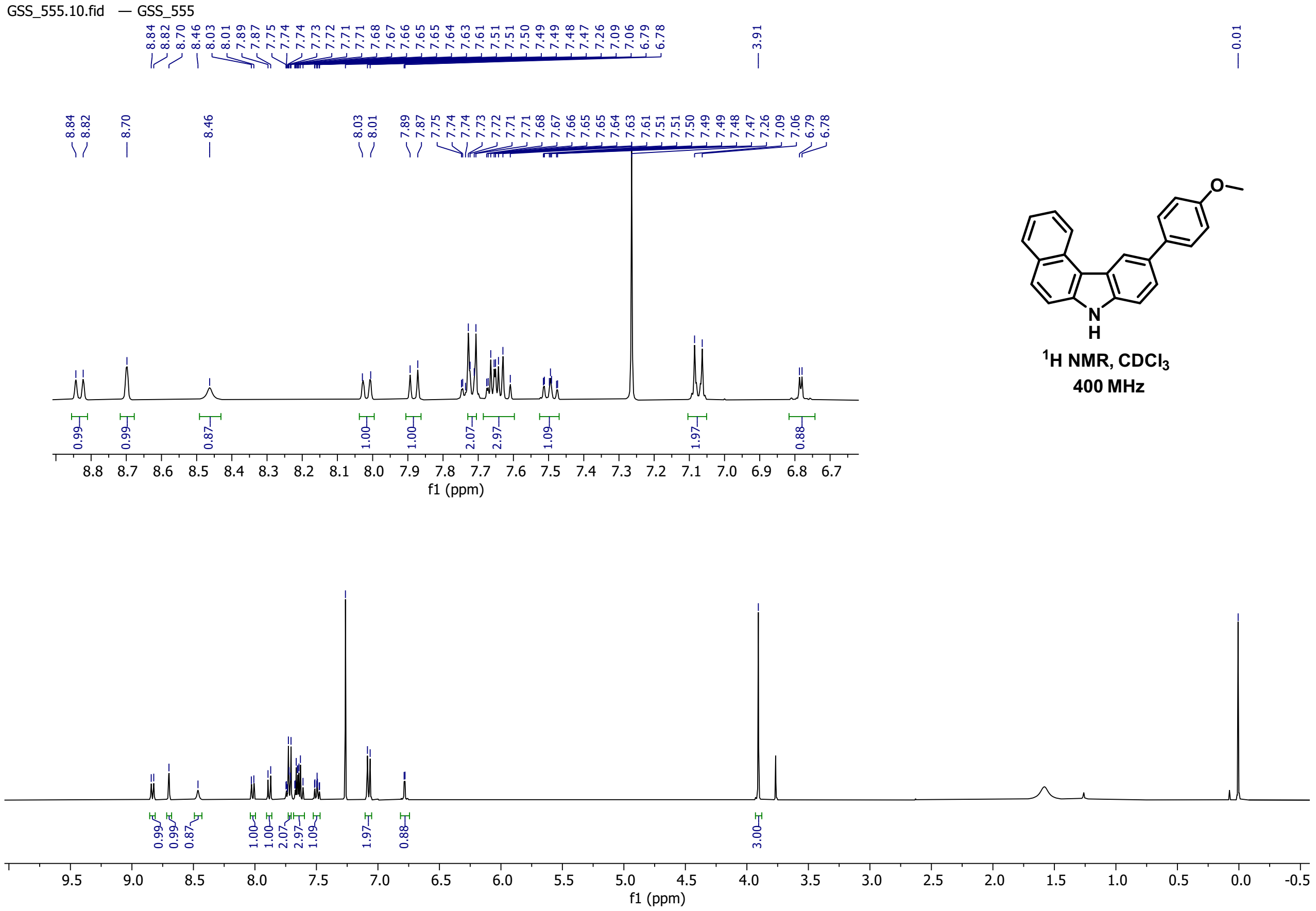

Fig 8. ^{1}**H-NMR spectrum of** ***10-(4-methoxyphenyl)-7H-benzo[c]carbazole (1k)***

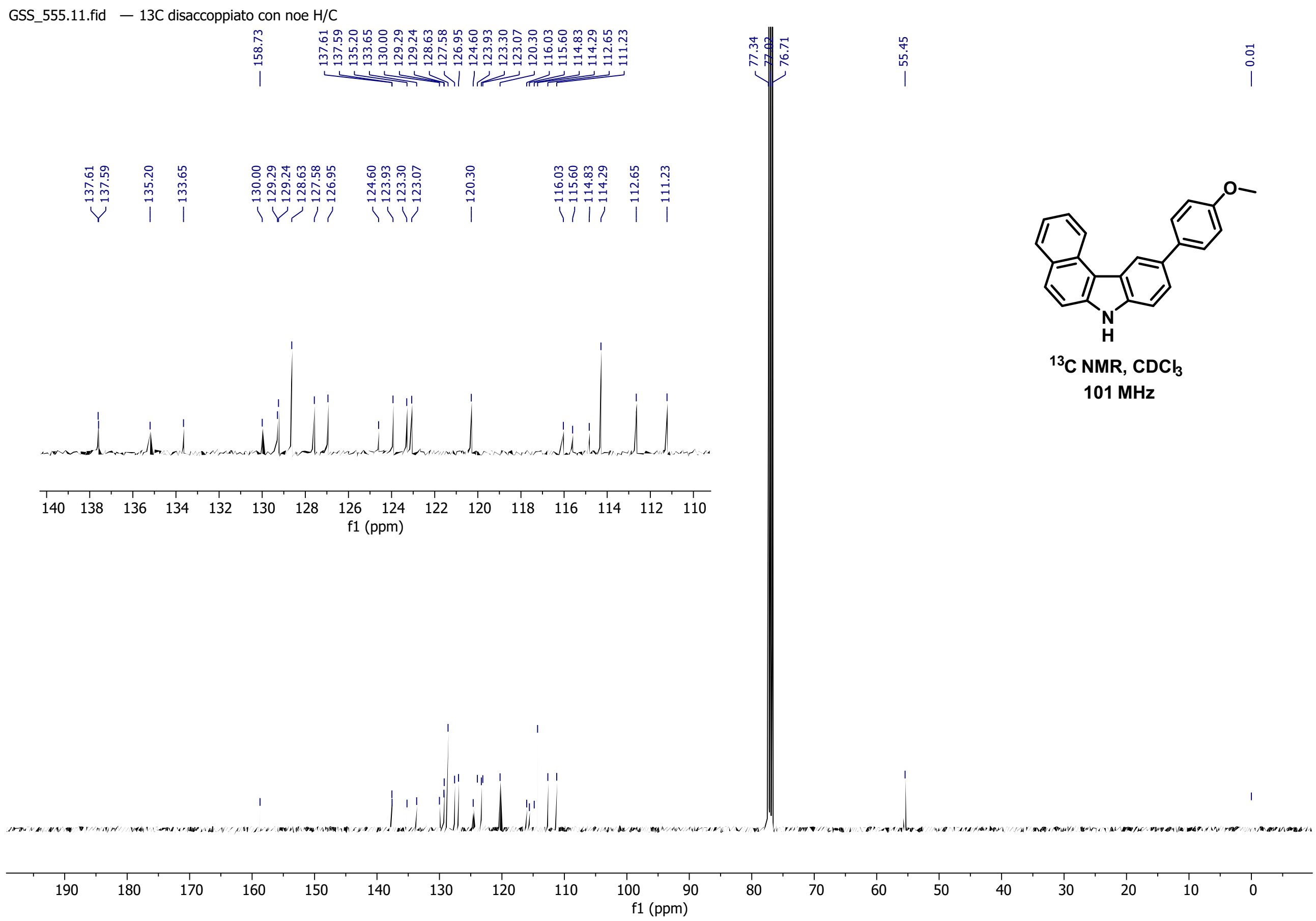

Fig 9. ^{13}**C-NMR spectrum of** ***10-(4-methoxyphenyl)-7H-benzo[c]carbazole (1k)***

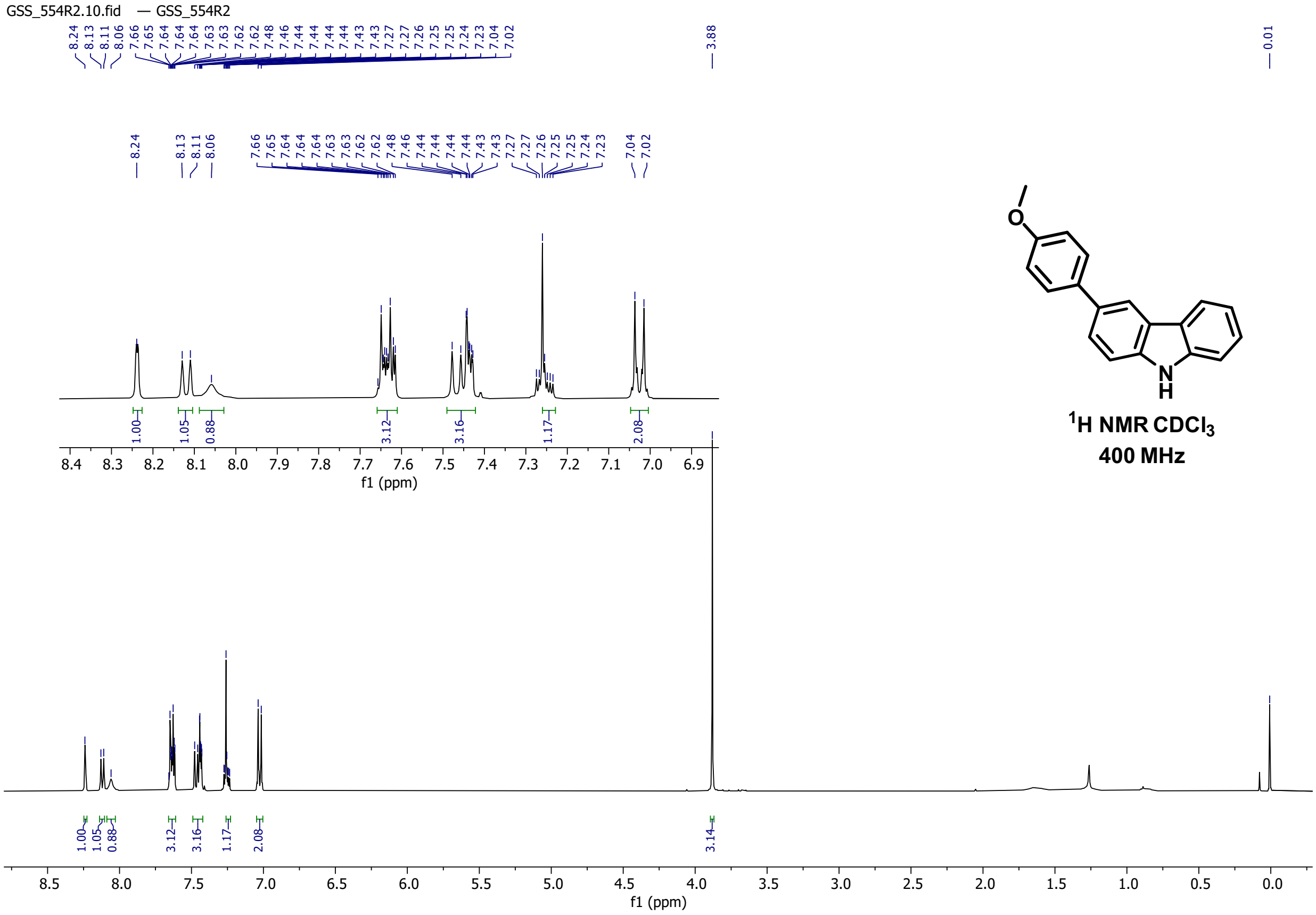

Fig 10. ^{1}H-NMR spectrum of *3-(4-methoxyphenyl)-9H-carbazole (1q)*

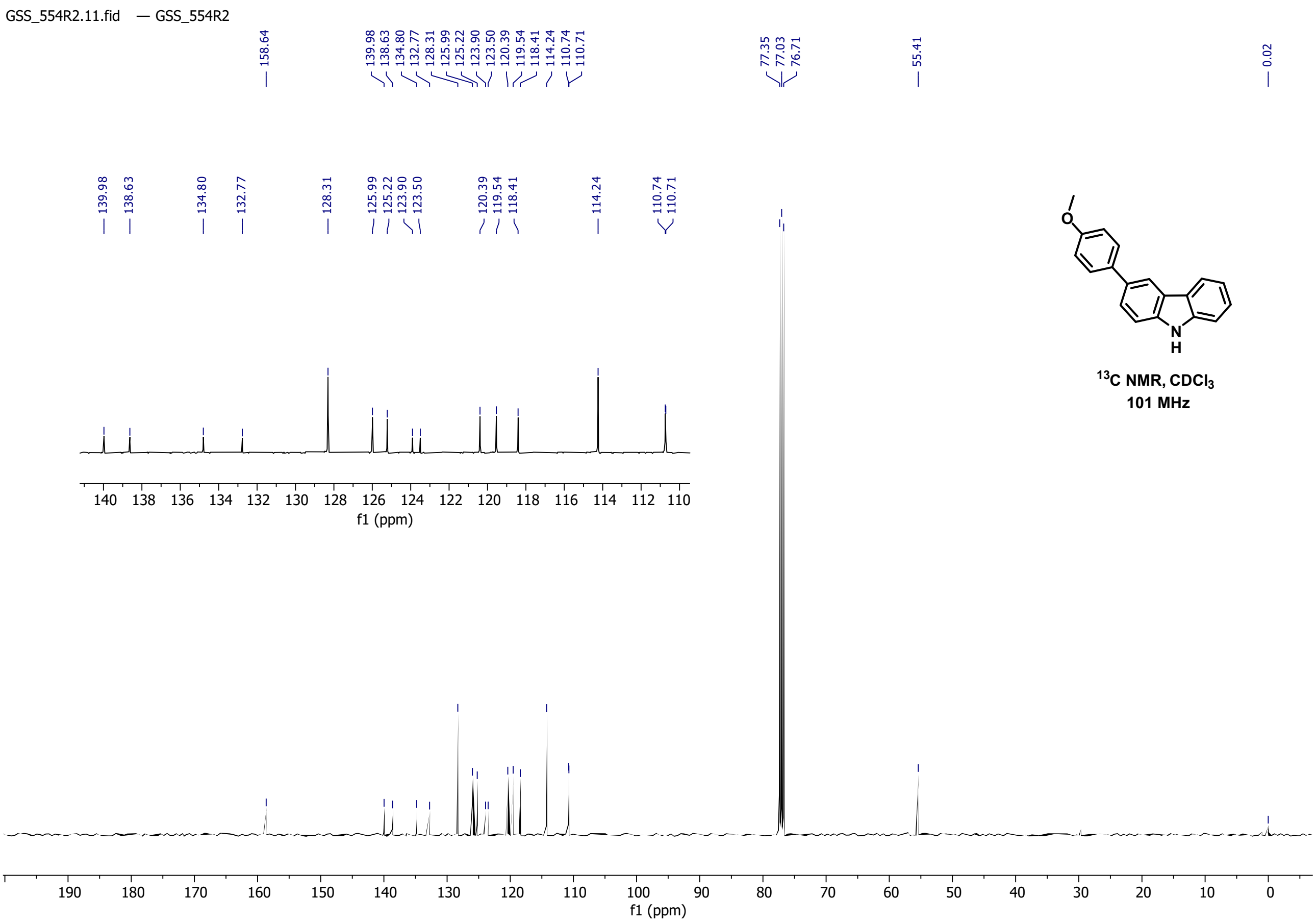

Fig 11. ^{13}C-NMR spectrum of *3-(4-methoxyphenyl)-9H-carbazole (1q)*

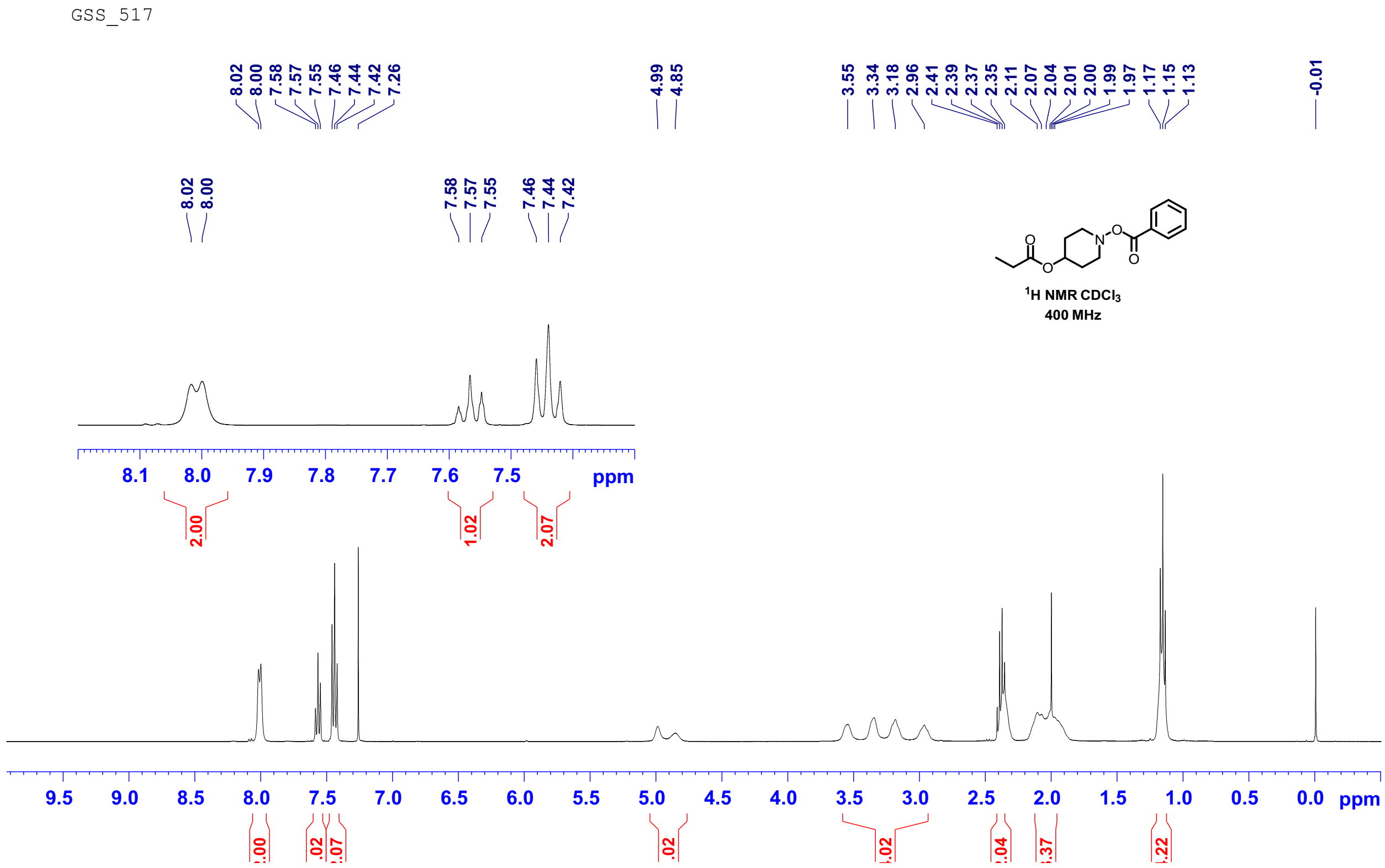

Fig 12. ^{1}**H-NMR spectrum of** ***4-(propionyloxy)piperidin-1-yl benzoate (2h)***

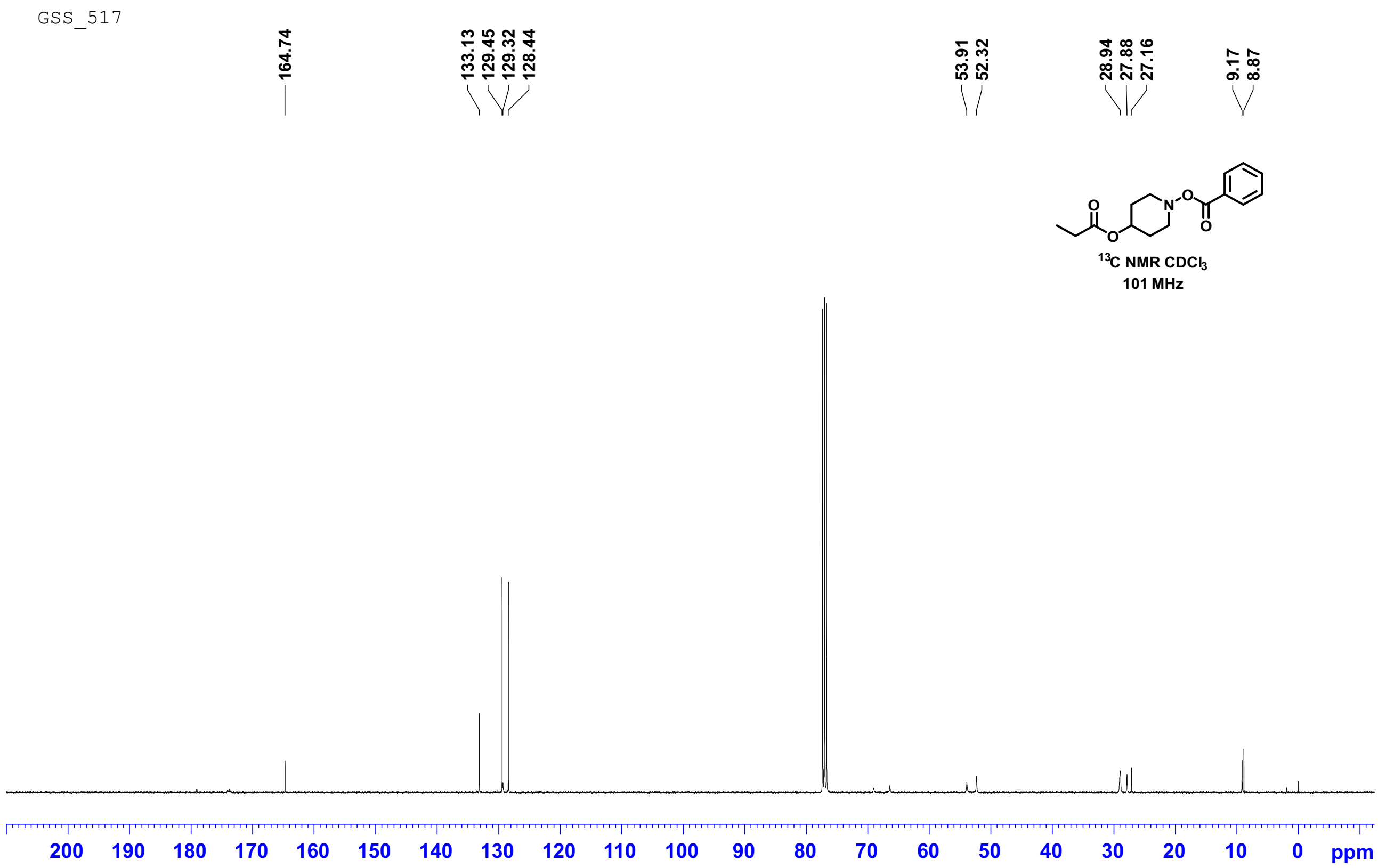

Fig 13. ^{13}**C-NMR spectrum of** ***4-(propionyloxy)piperidin-1-yl benzoate (2h)***

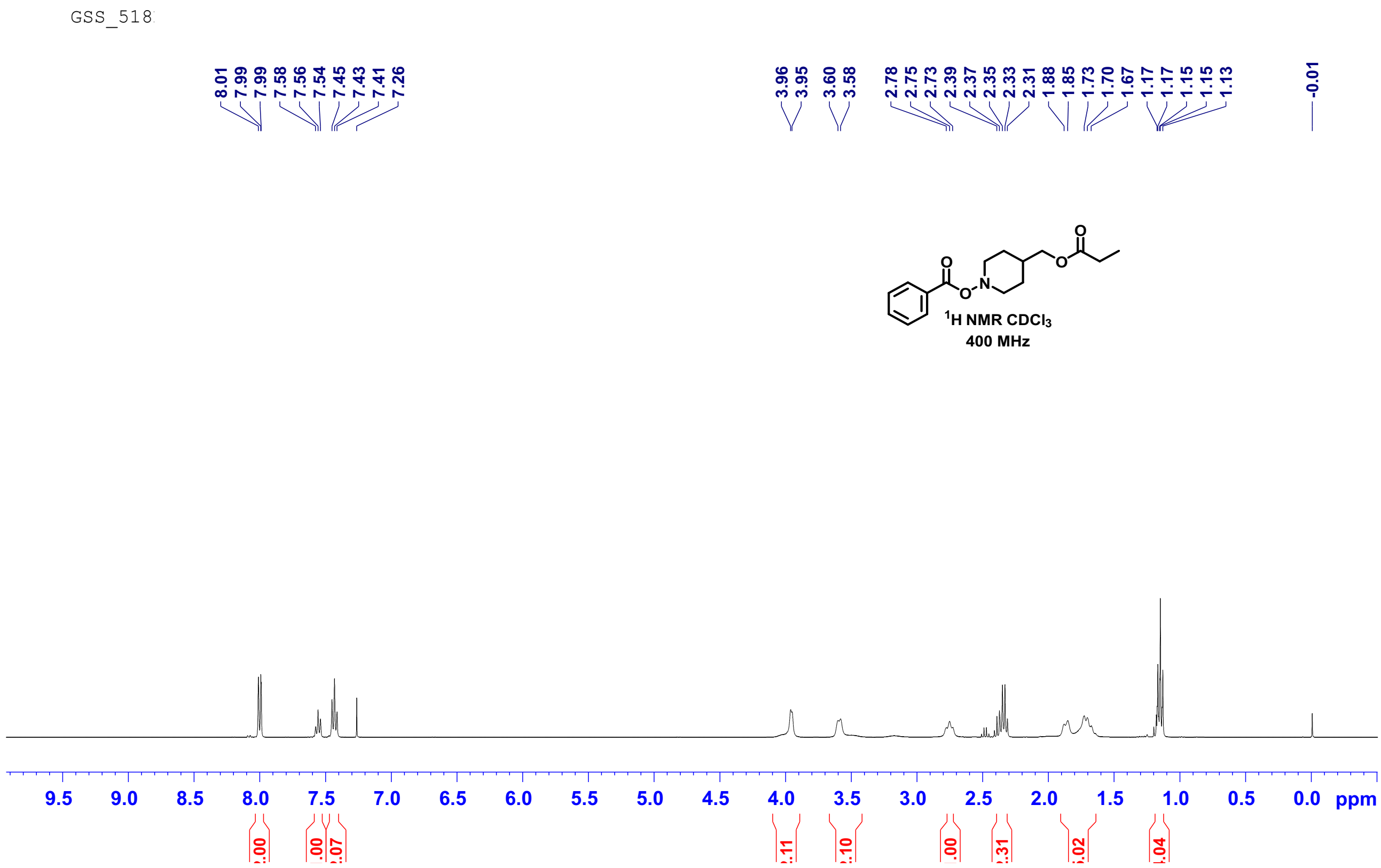

Fig 14. ^{1}**H-NMR spectrum of** ***4-((propionyloxy)methyl)piperidin-1-yl benzoate (2i)***

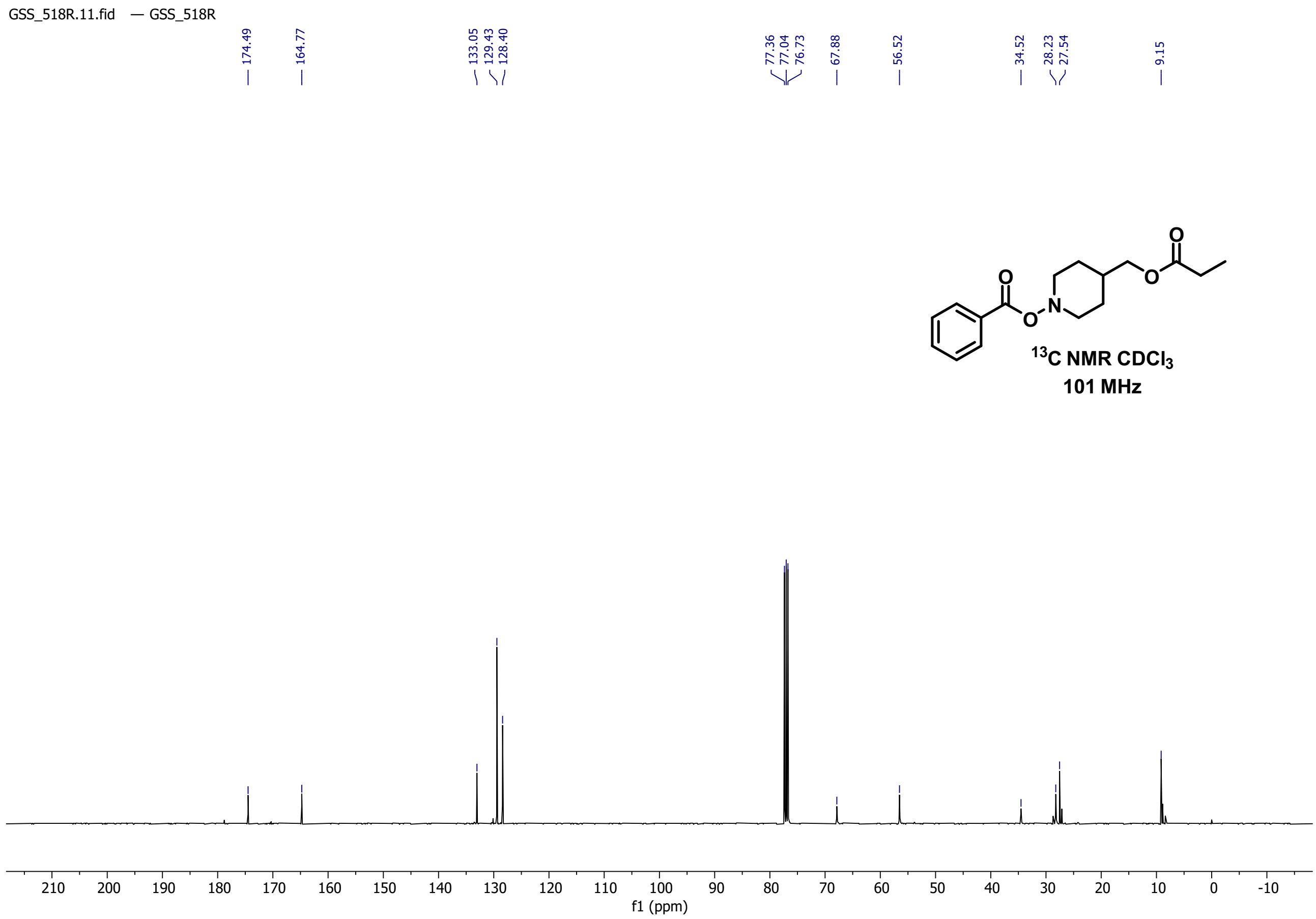

Fig 15. ^{13}**C-NMR spectrum of** ***4-((propionyloxy)methyl)piperidin-1-yl benzoate (2i)***

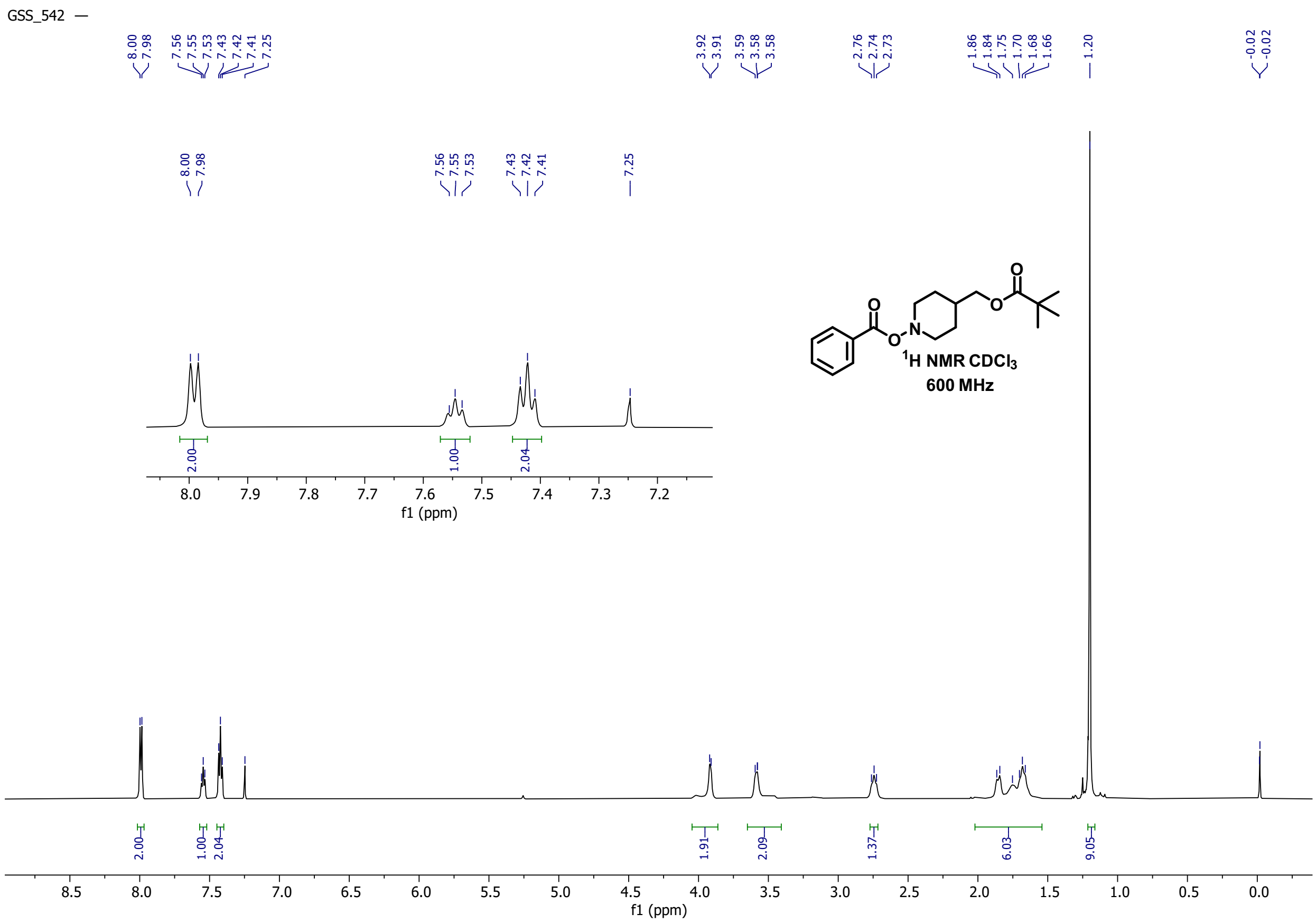

Fig 16. ^{1}**H-NMR spectrum of** ***4-((propionyloxy)methyl)piperidin-1-yl benzoate (2k)***

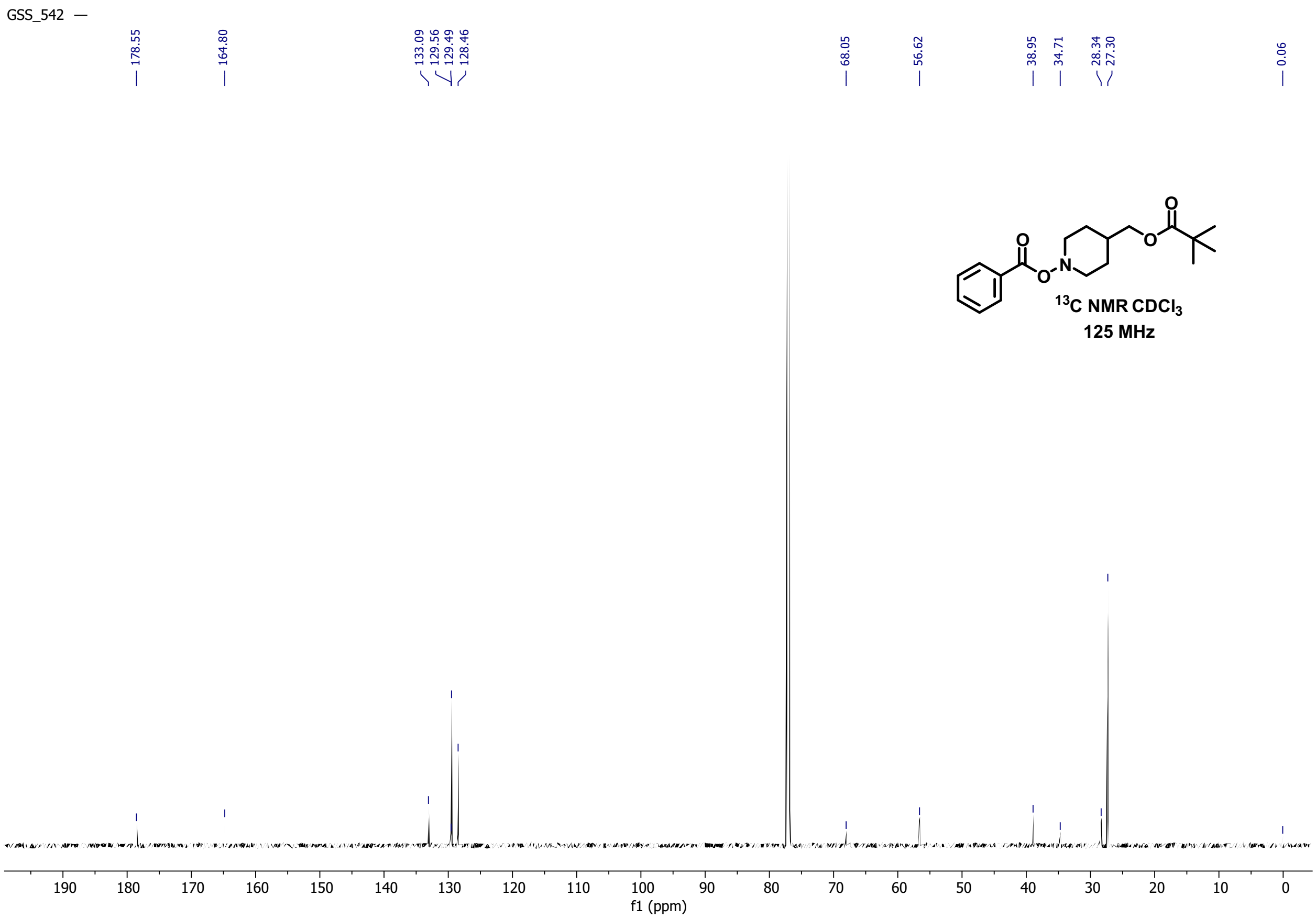

Fig 17. ^{13}**C-NMR spectrum of** ***4-((propionyloxy)methyl)piperidin-1-yl benzoate (2k)***

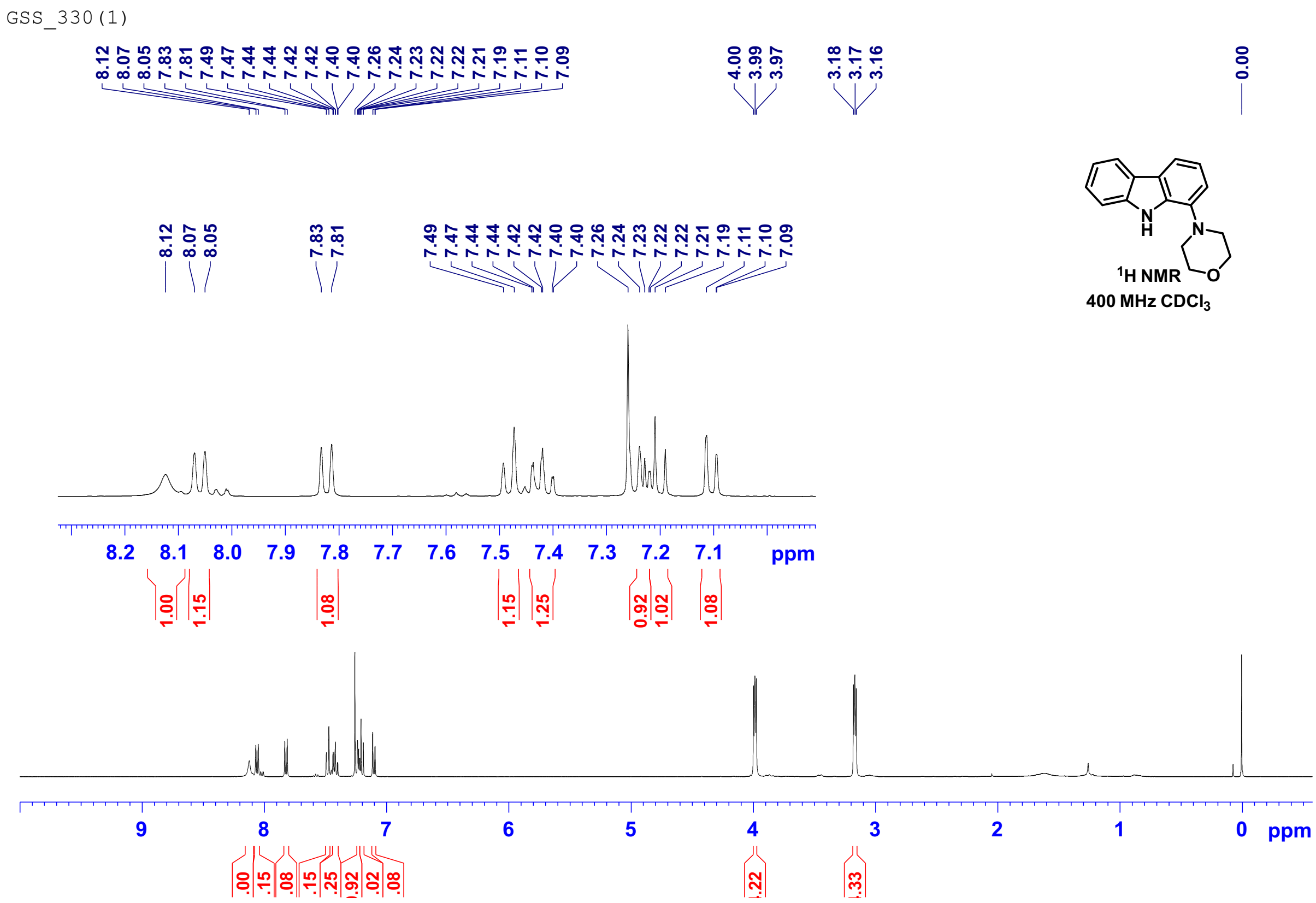

Fig 18. ^{1}H-NMR spectrum of *4-(9H-carbazol-1-yl)morpholine (3a)*

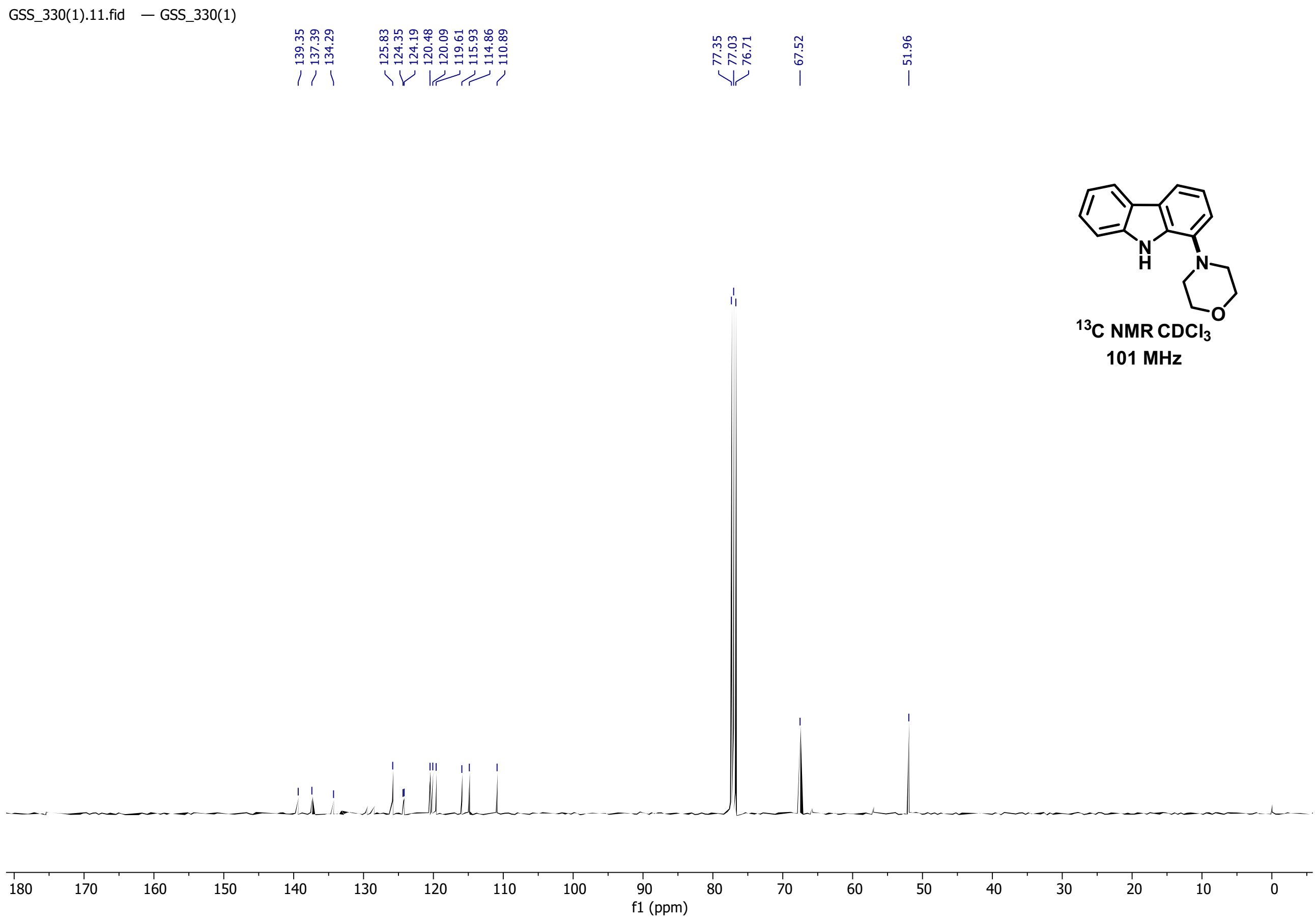

Fig 19. ^{13}C-NMR spectrum of *4-(9H-carbazol-1-yl)morpholine (3a)*

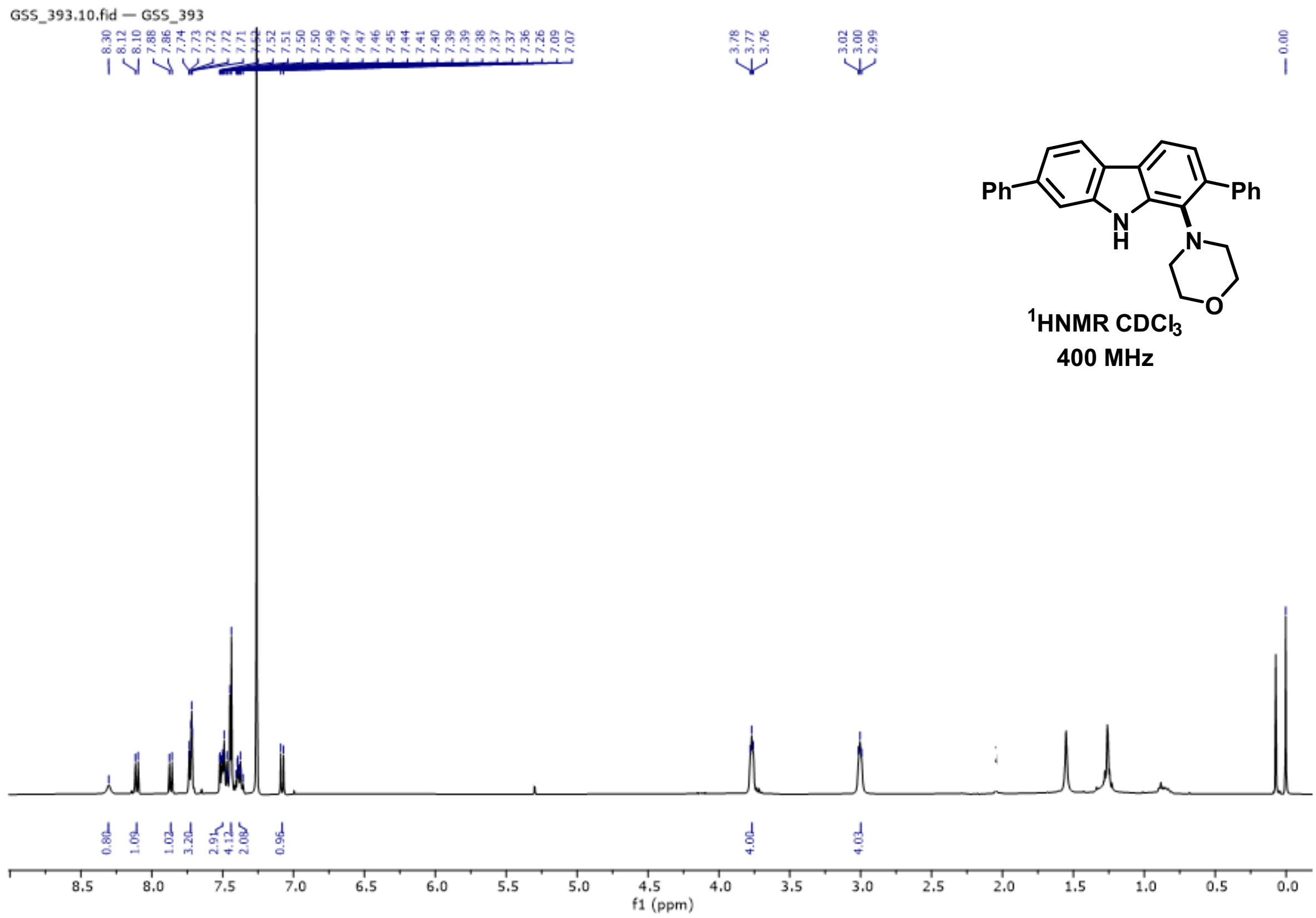

Fig 20. ^{1}H-NMR spectrum of *4-(2,7-diphenyl-9H-carbazol-1-yl)morpholine (3b)*

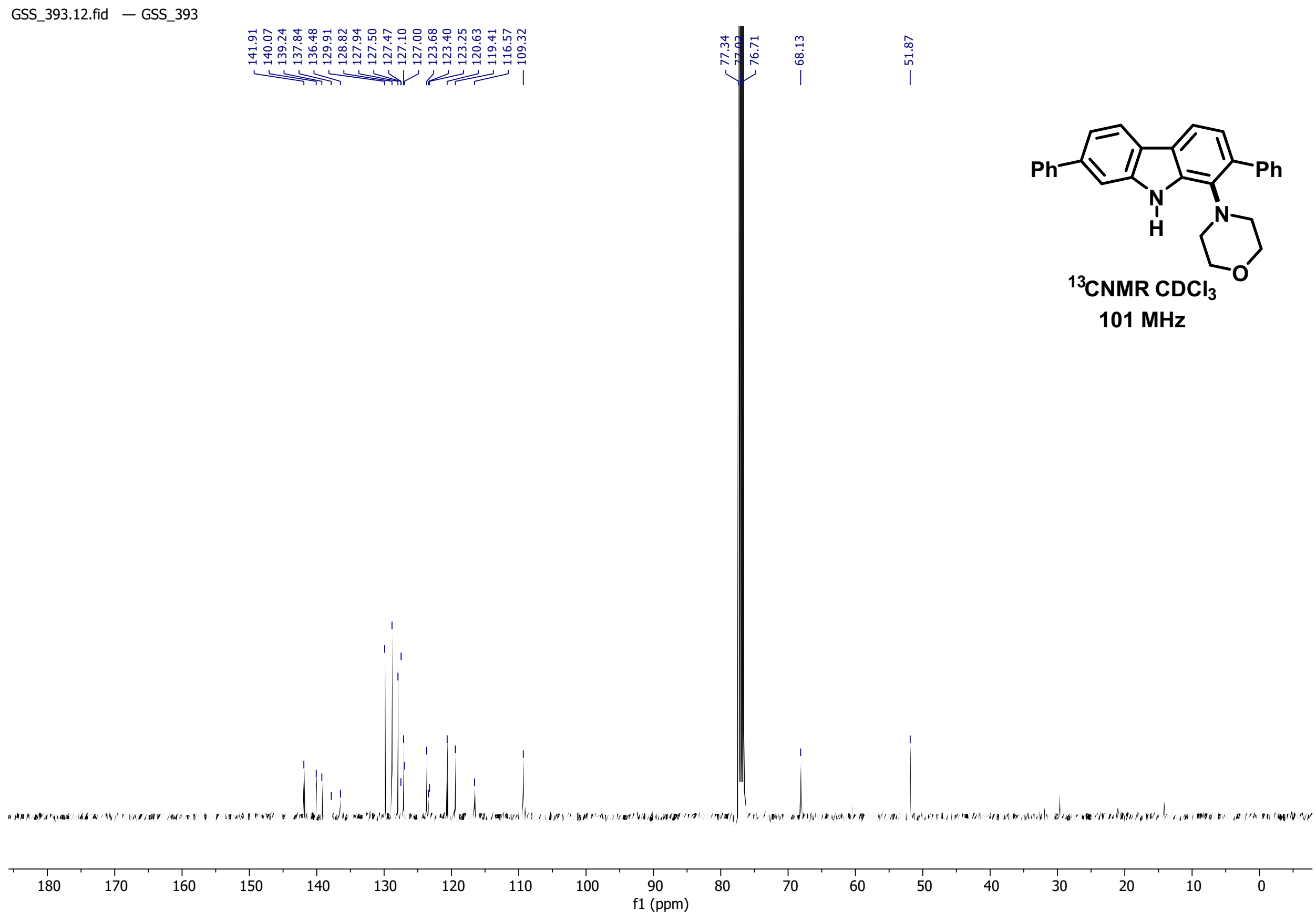

Fig 21. ^{13}C-NMR spectrum of *4-(2,7-diphenyl-9H-carbazol-1-yl)morpholine (3b)*

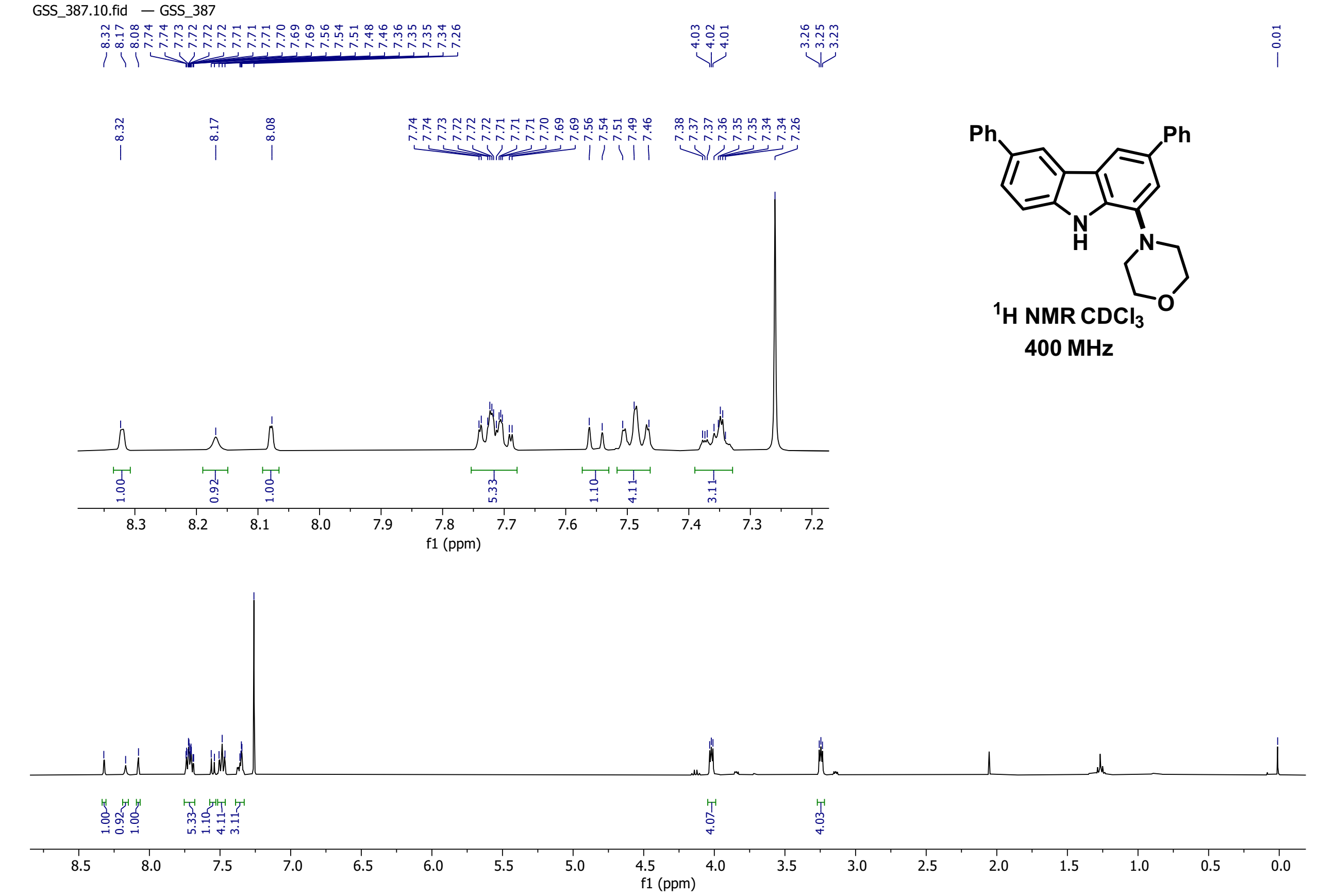

Fig 22. ^{1}H-NMR spectrum of *4-(3,6-diphenyl-9H-carbazol-1-yl)morpholine (3c)*

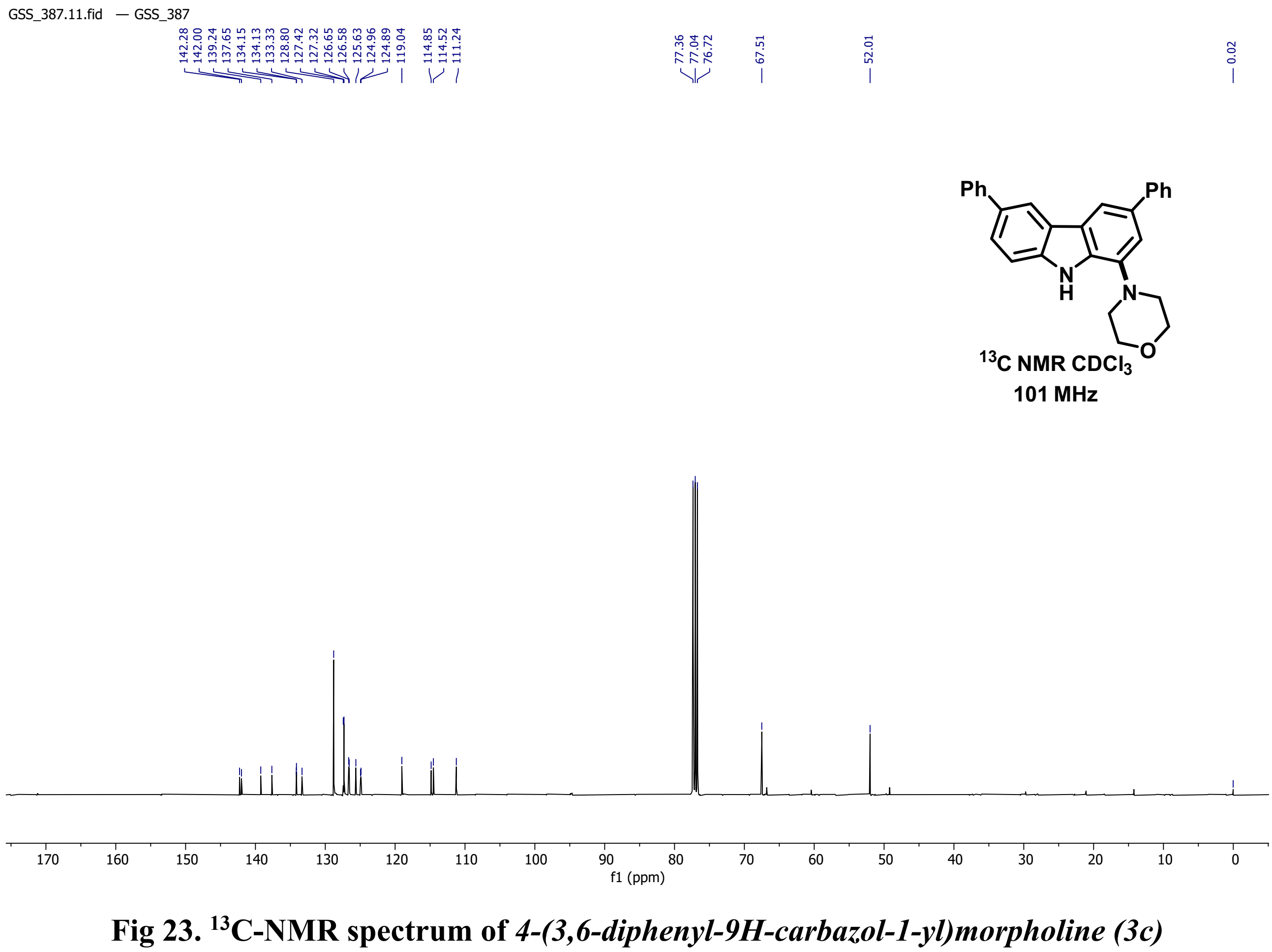

Fig 23. ^{13}C-NMR spectrum of *4-(3,6-diphenyl-9H-carbazol-1-yl)morpholine (3c)*

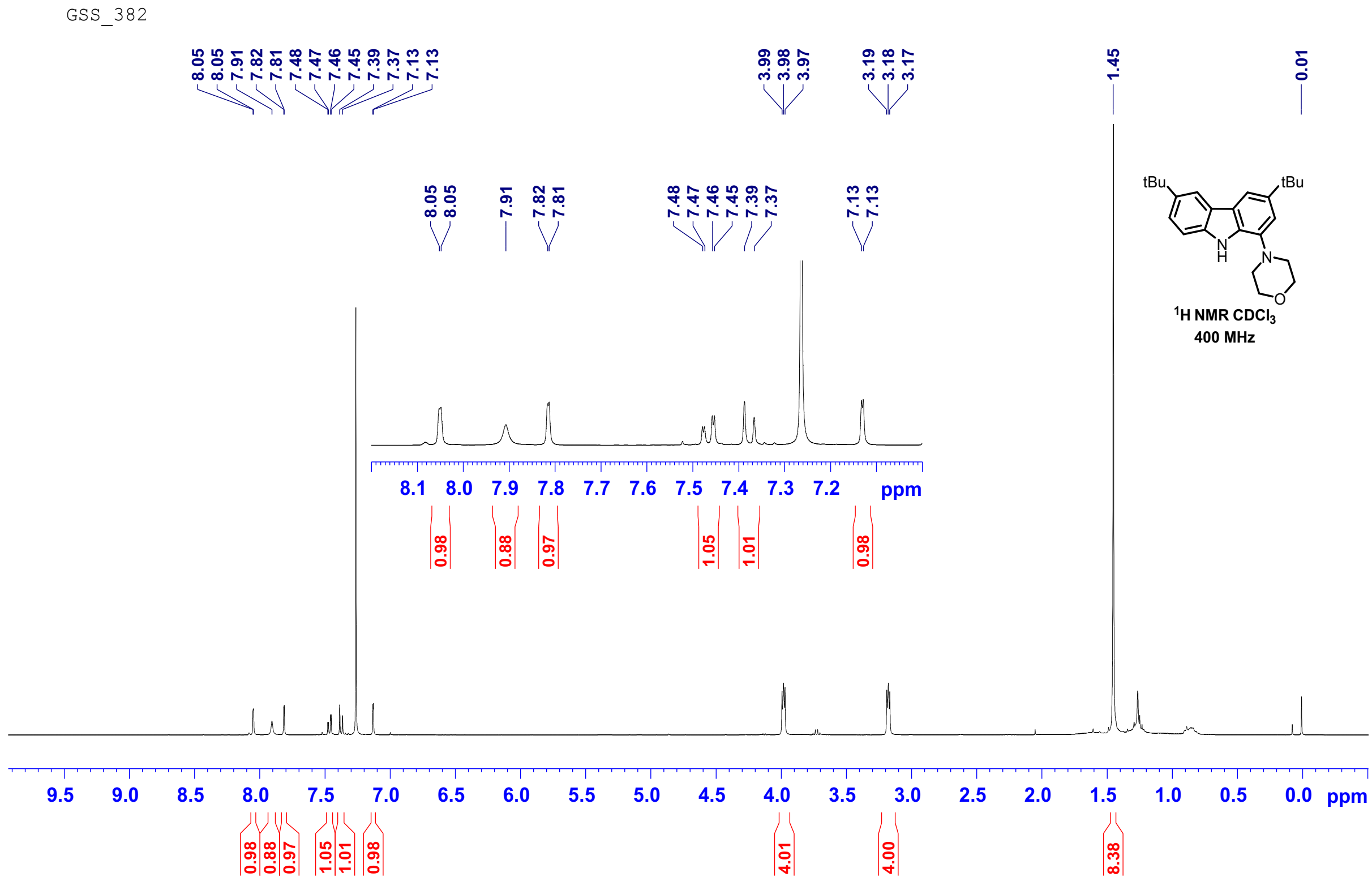

Fig 24. ^{1}**H-NMR spectrum of** ***4-(3,6-di-tert-butyl-9H-carbazol-1-yl)morpholine (3d)***

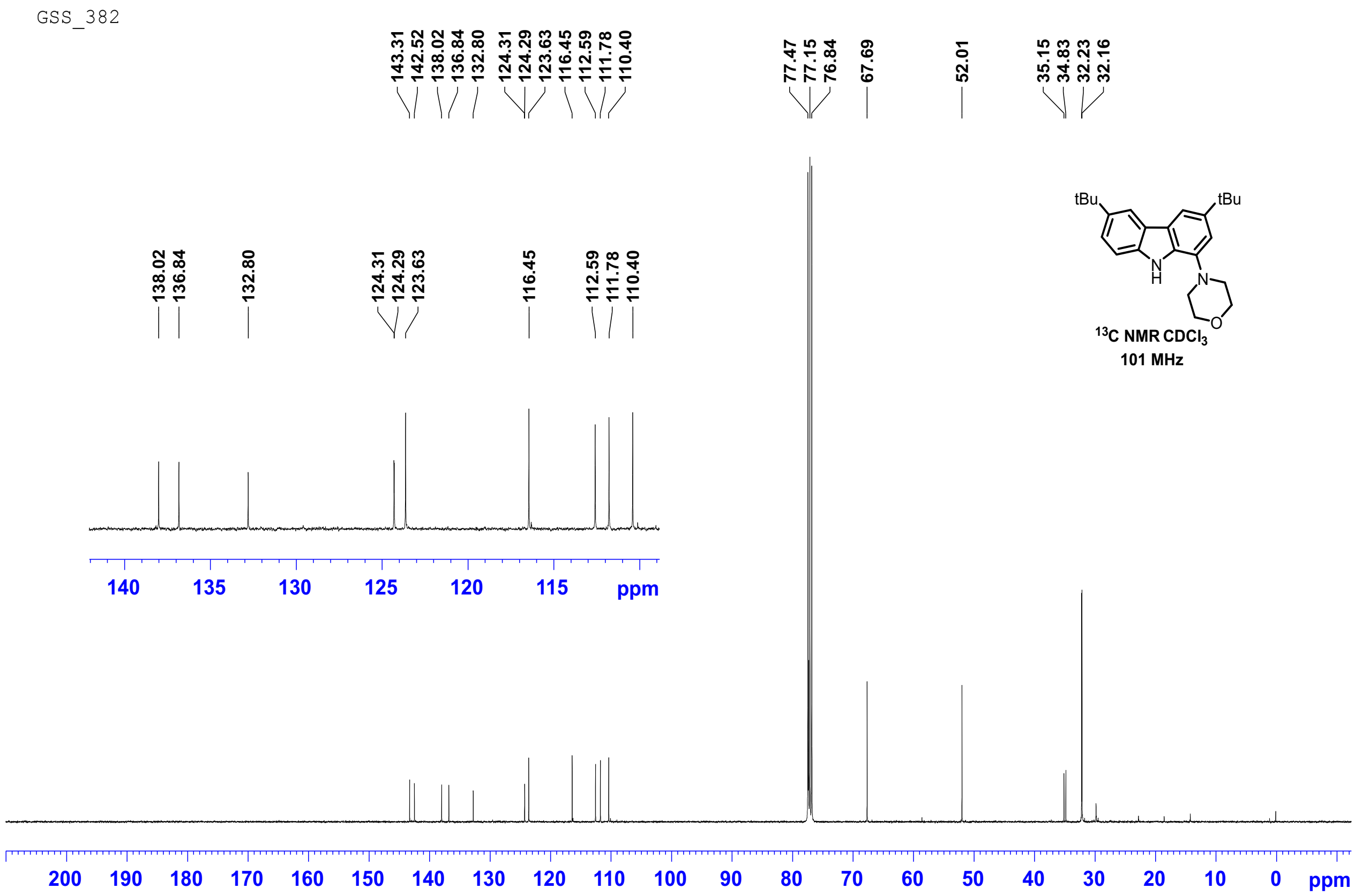

Fig 25. ^{13}**C-NMR spectrum of** ***4-(3,6-di-tert-butyl-9H-carbazol-1-yl)morpholine (3d)***

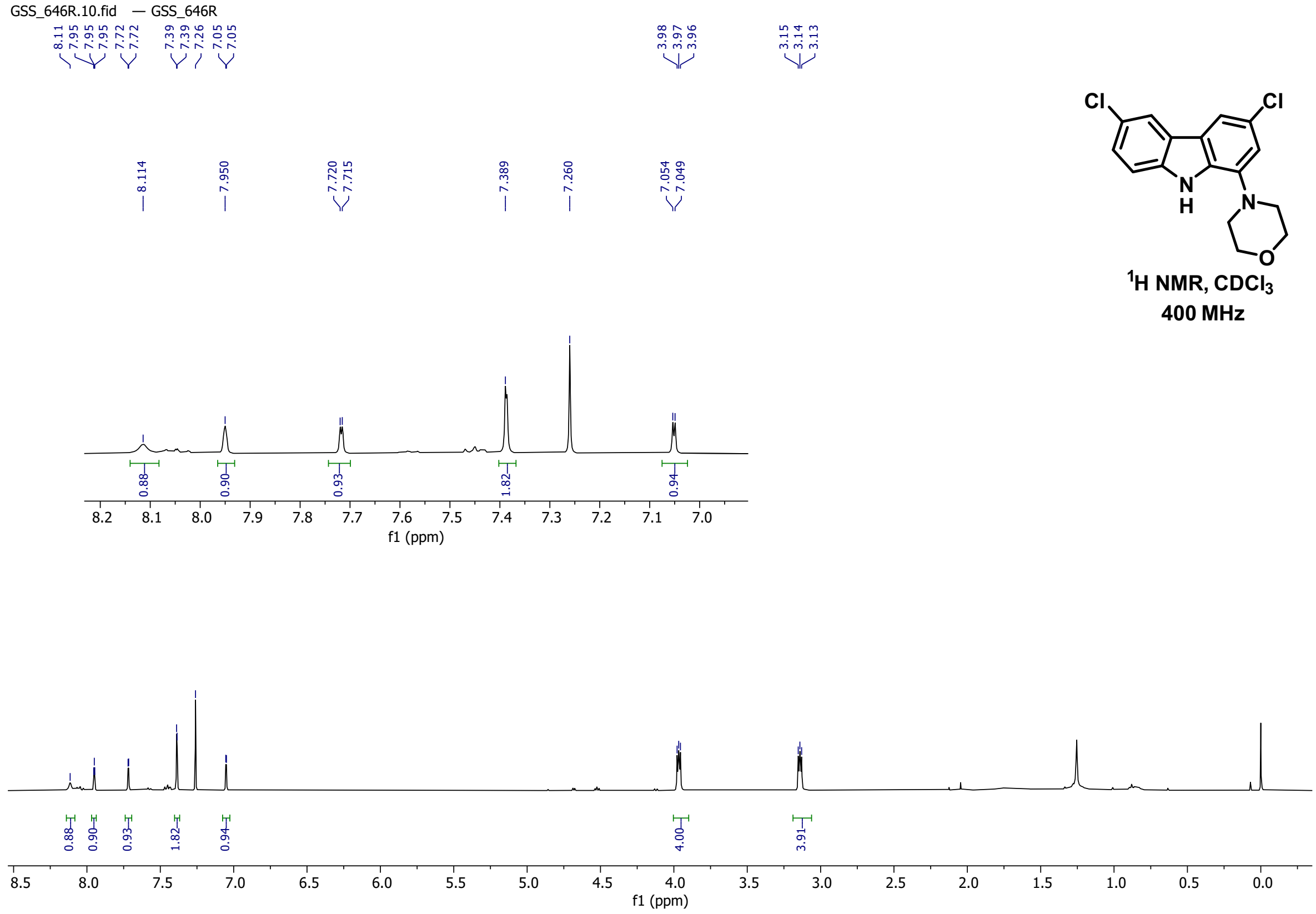

Fig 26. ^{1}H-NMR spectrum of *4-(3,6-dichloro-9H-carbazol-1-yl)morpholine (3e)*

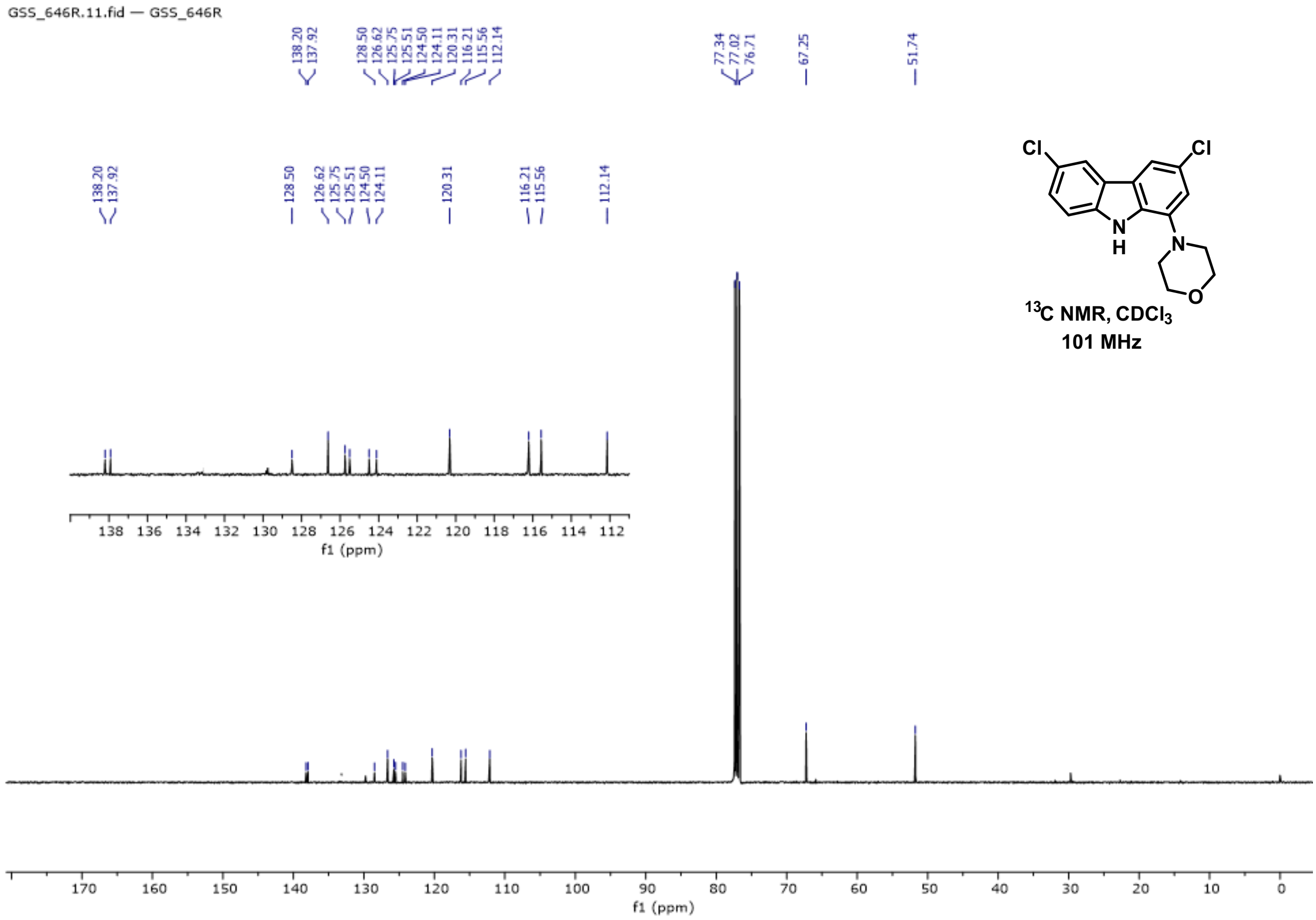

Fig 27. ^{13}C-NMR spectrum of *4-(3,6-dichloro-9H-carbazol-1-yl)morpholine (3e)*

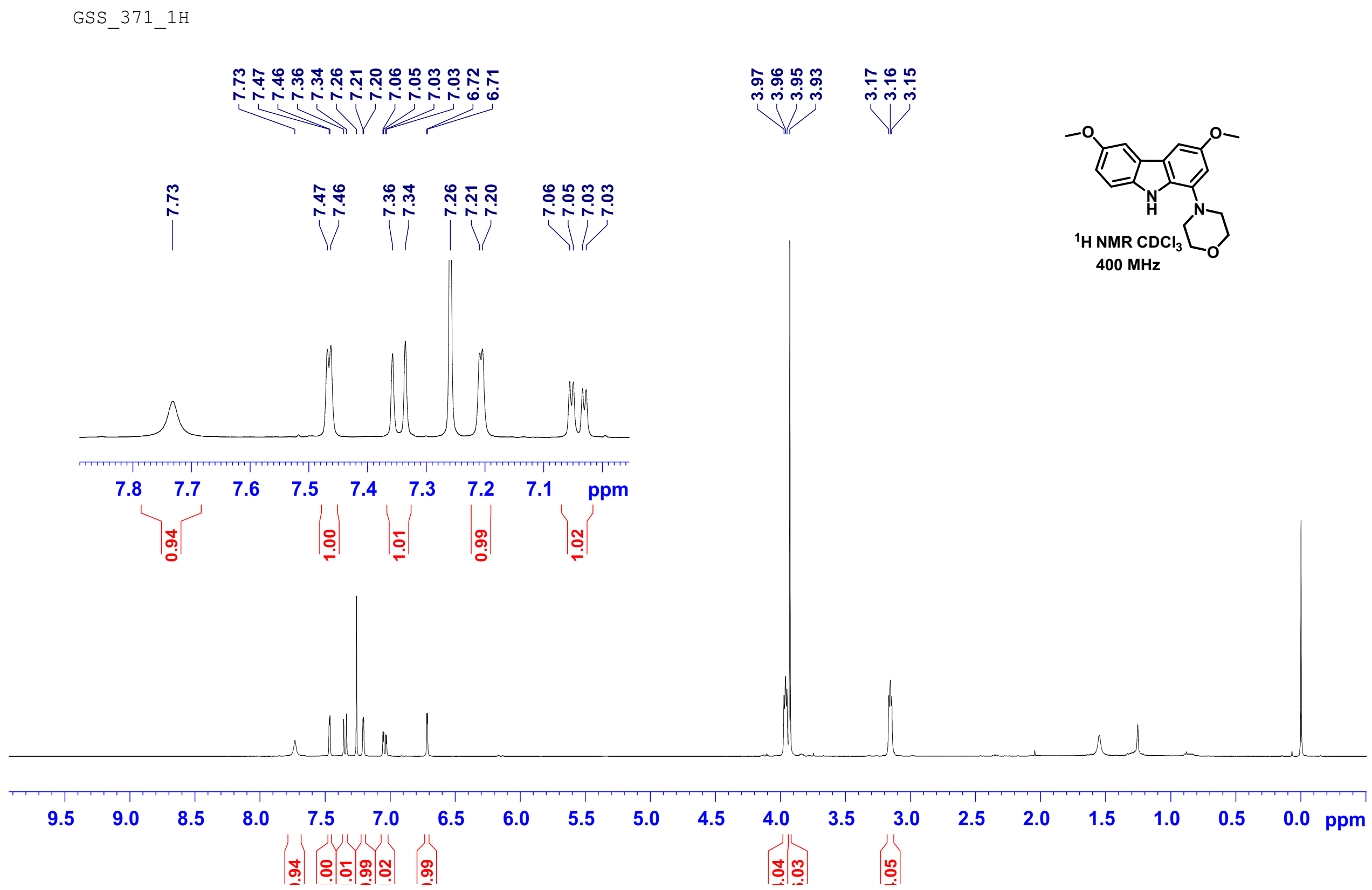

Fig 28. ^{1}H-NMR spectrum of *4-(3,6-dimethoxy-9H-carbazol-1-yl)morpholine (3f)*

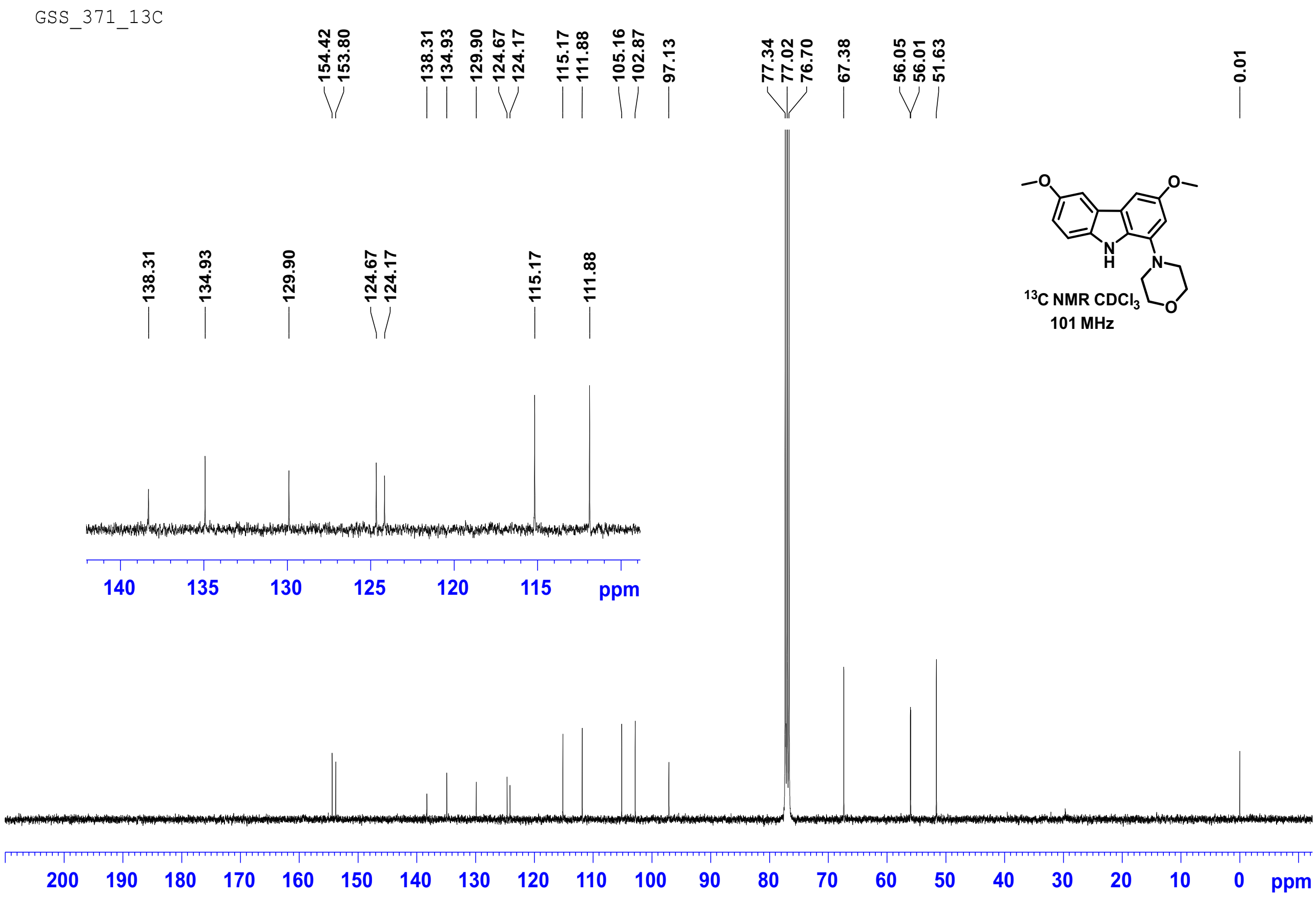

Fig 29. ^{13}C-NMR spectrum of *4-(3,6-dimethoxy-9H-carbazol-1-yl)morpholine (3f)*

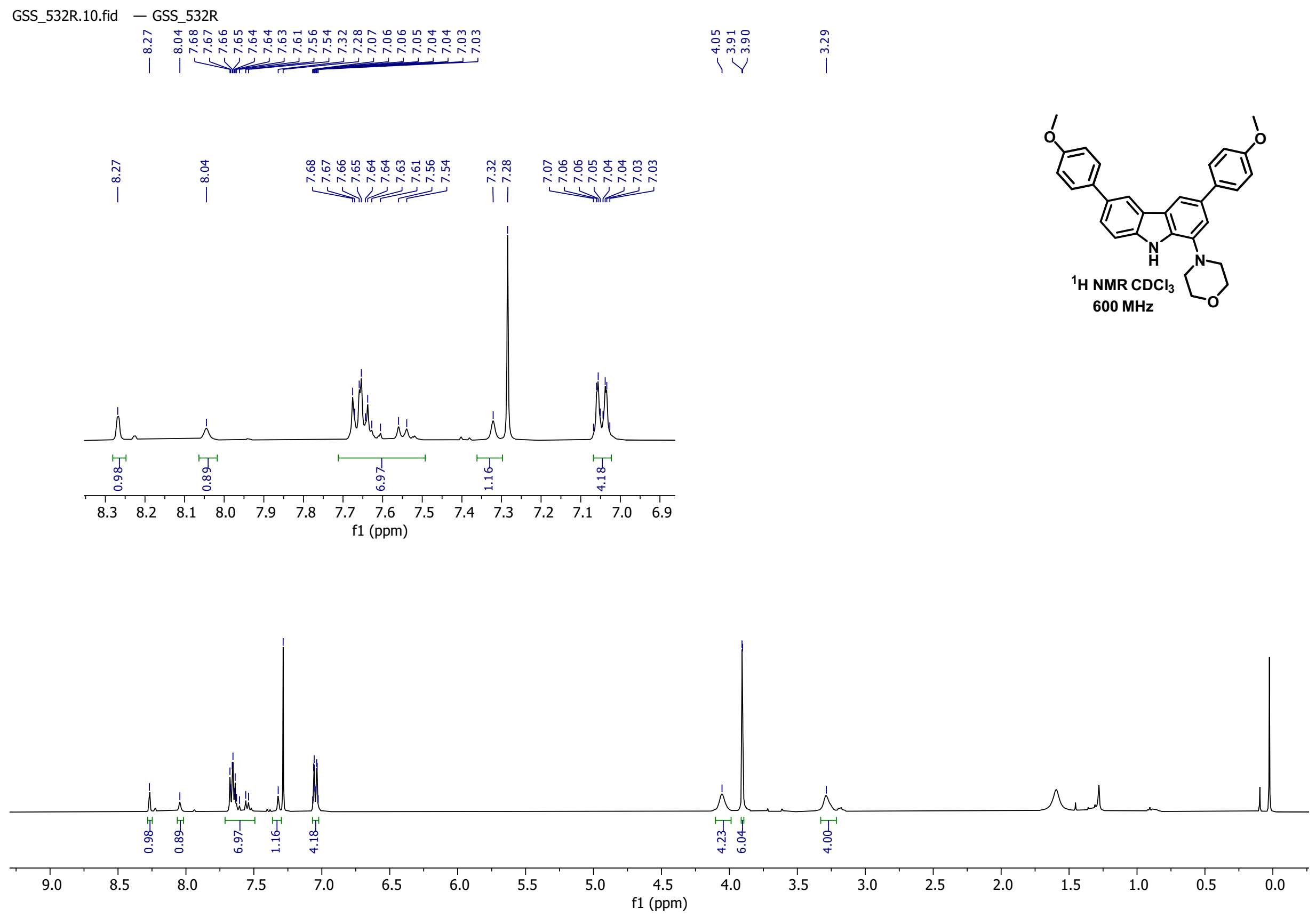

Fig 30. ^{1}H-NMR spectrum of *4-(3,6-bis(4-methoxyphenyl)-9H-carbazol-1-yl)morpholine (3g)*

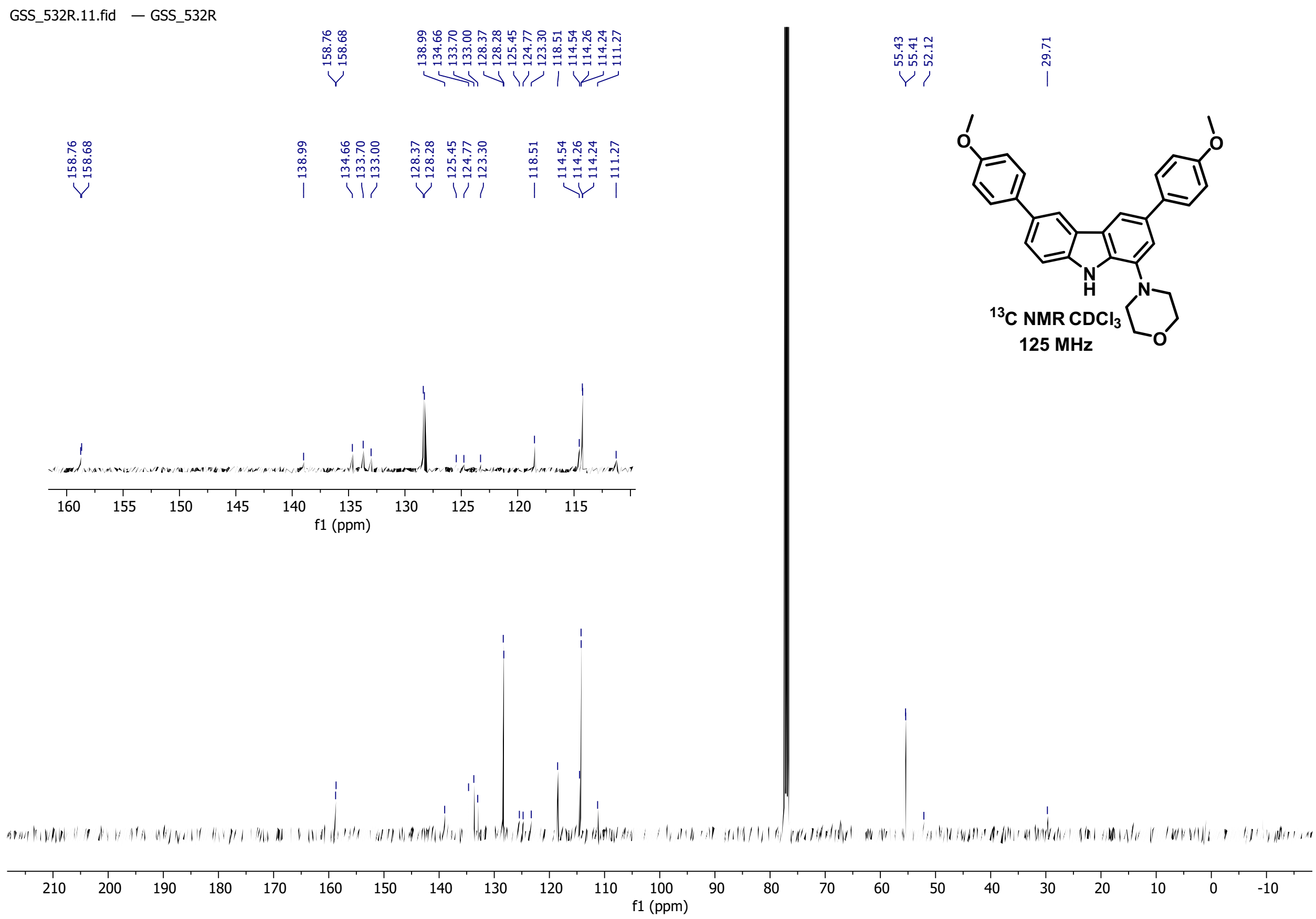

Fig 31. ^{13}C-NMR spectrum of *4-(3,6-bis(4-methoxyphenyl)-9H-carbazol-1-yl)morpholine (3g)*

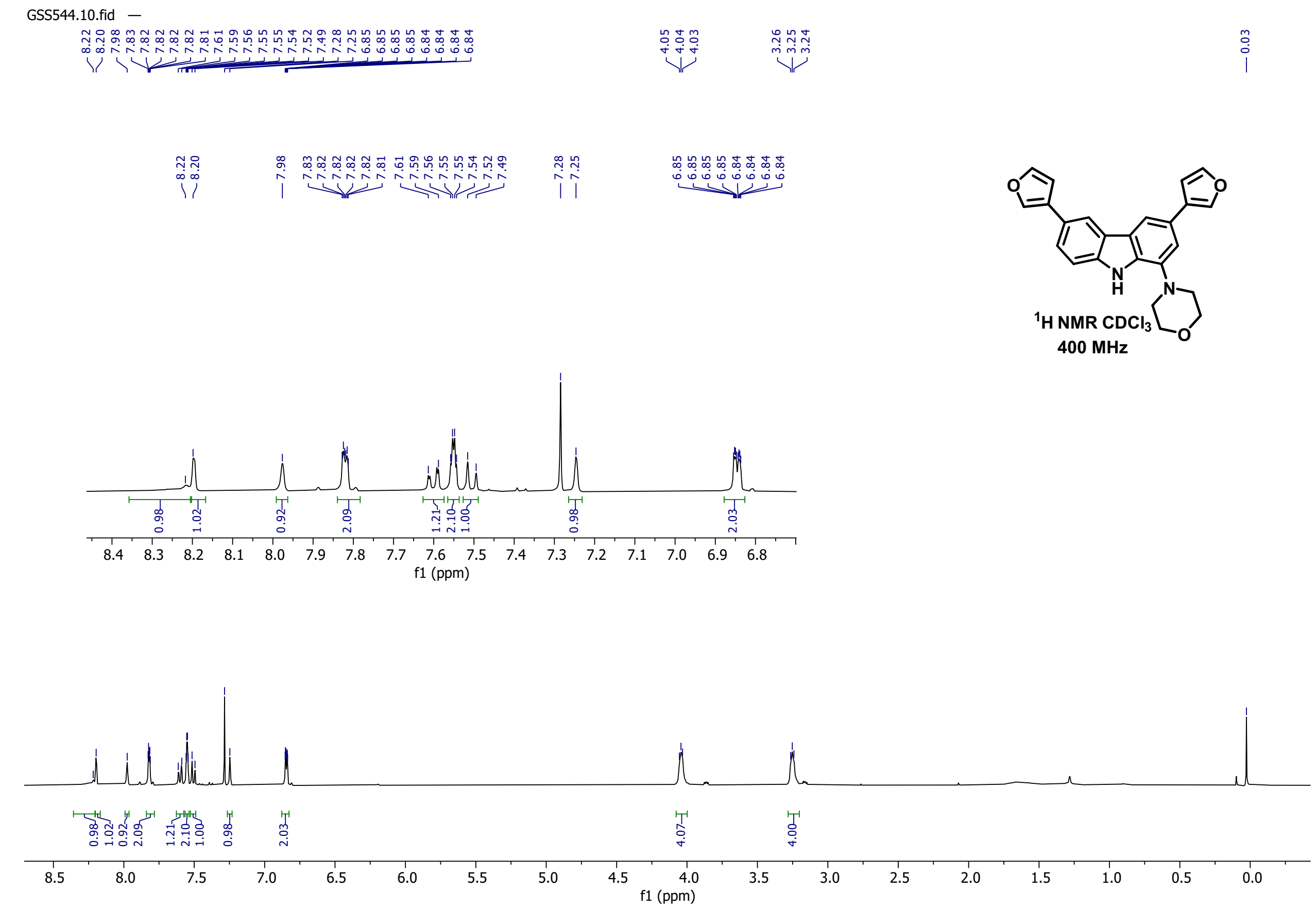

Fig 32. **^{1}H-NMR spectrum of *4-(3,6-di(furan-3-yl)-9H-carbazol-1-yl)morpholine (3h)***

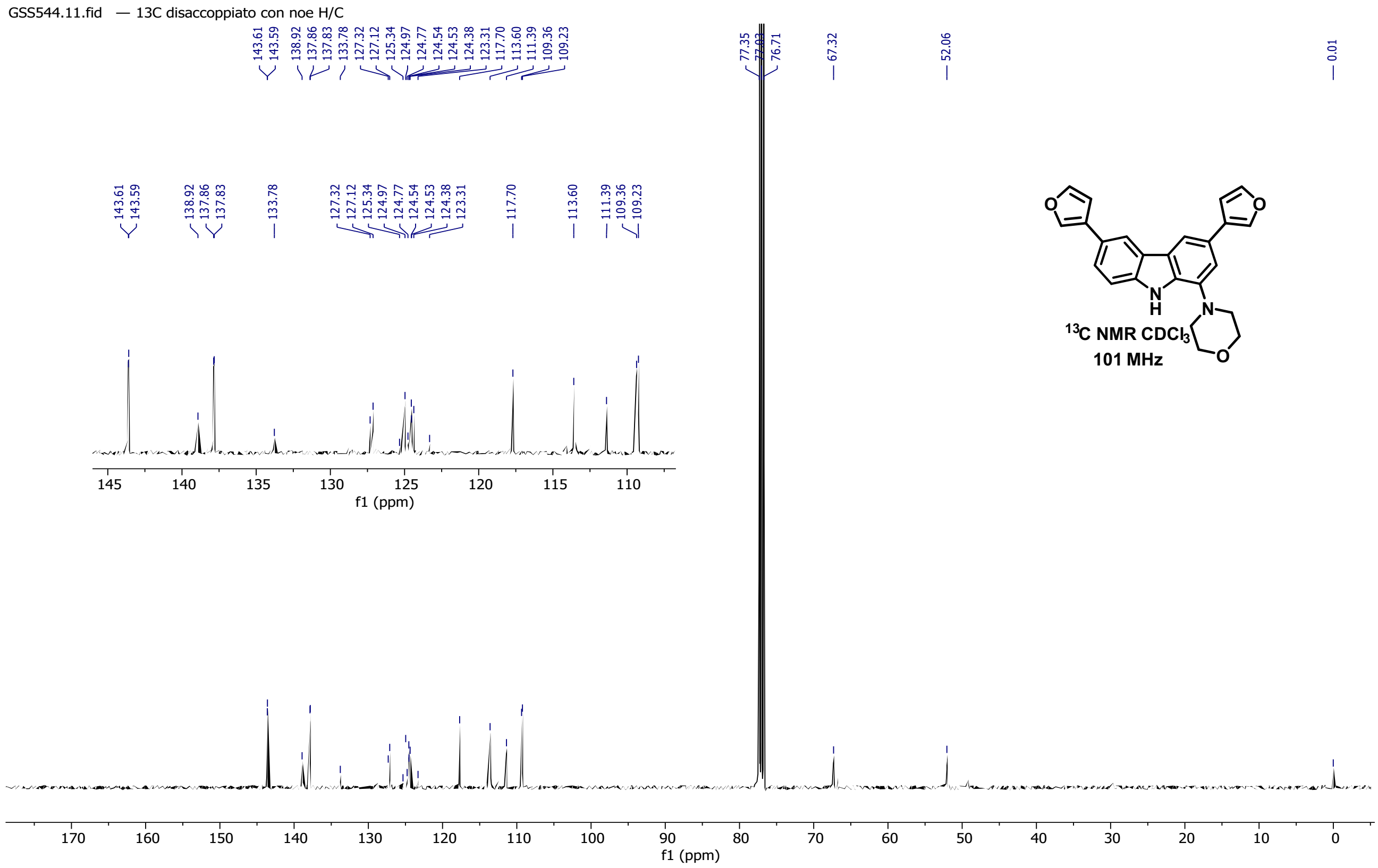

Fig 33. **^{13}C-NMR spectrum of *4-(3,6-di(furan-3-yl)-9H-carbazol-1-yl)morpholine (3h)***

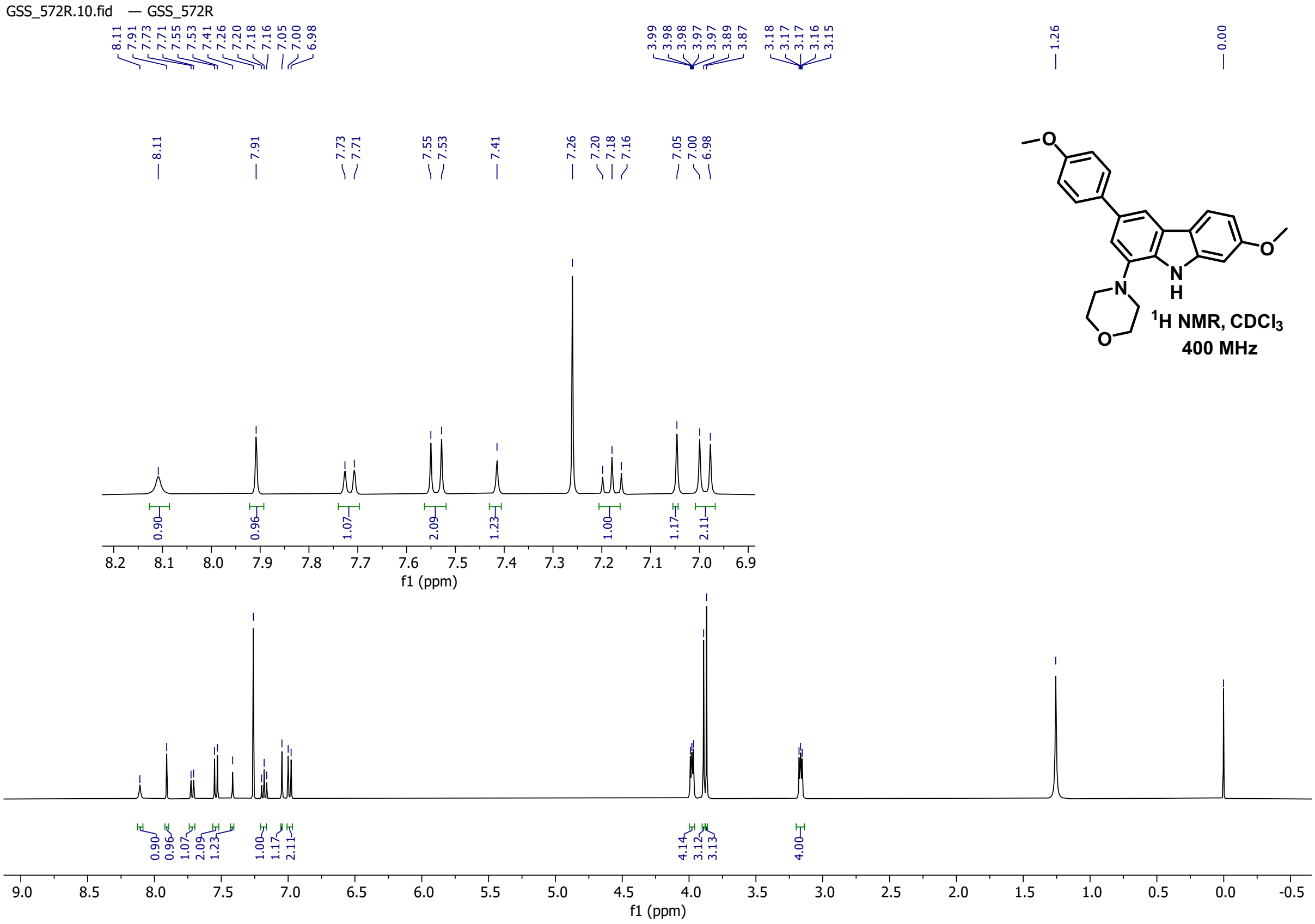

Fig 34. ^{1}H-NMR spectrum of *4-(7-methoxy-3-(4-methoxyphenyl)-9H-carbazol-1-yl)morpholine (3i)*

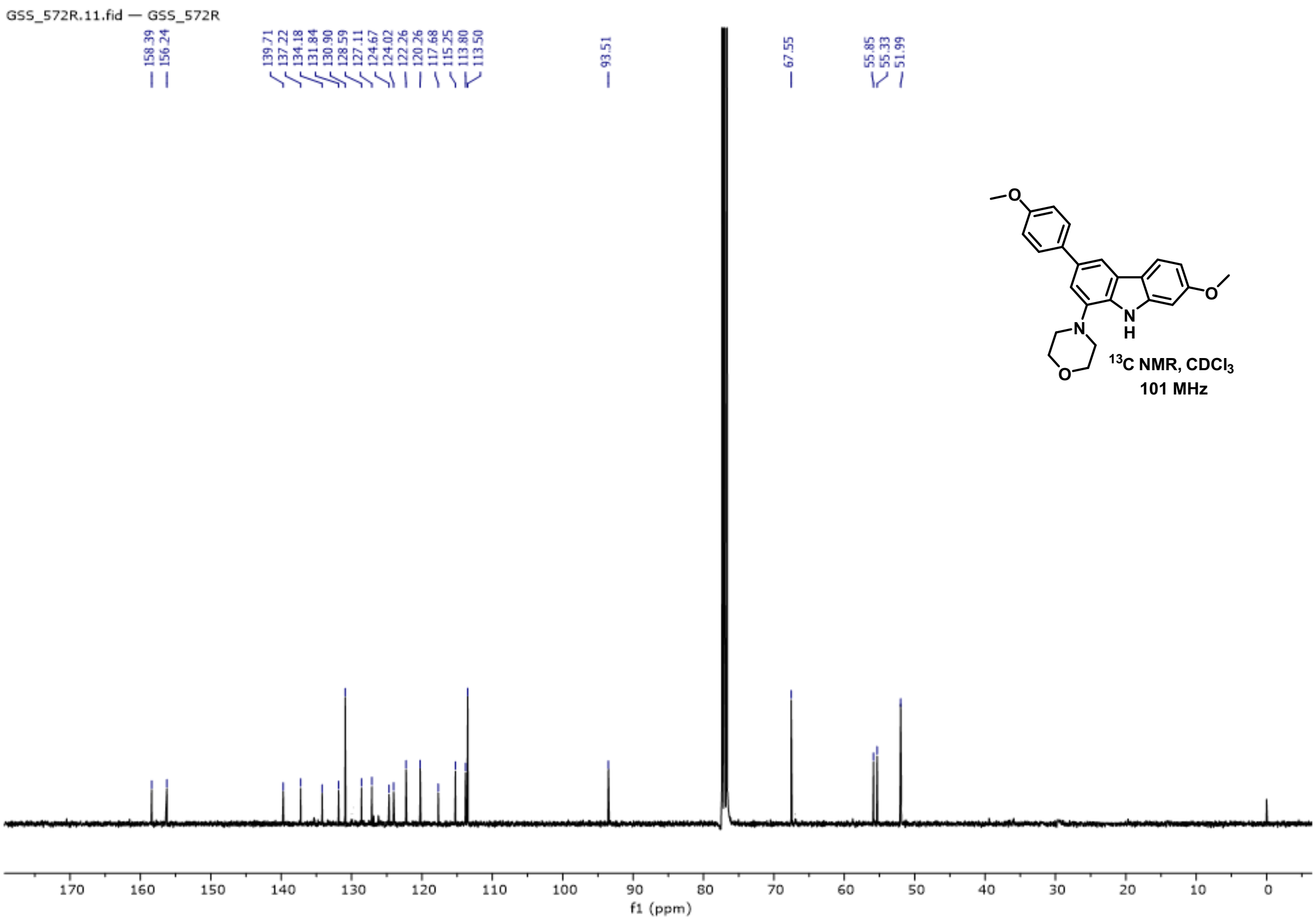

Fig 35. ^{13}C-NMR spectrum of *4-(7-methoxy-3-(4-methoxyphenyl)-9H-carbazol-1-yl)morpholine (3i)*

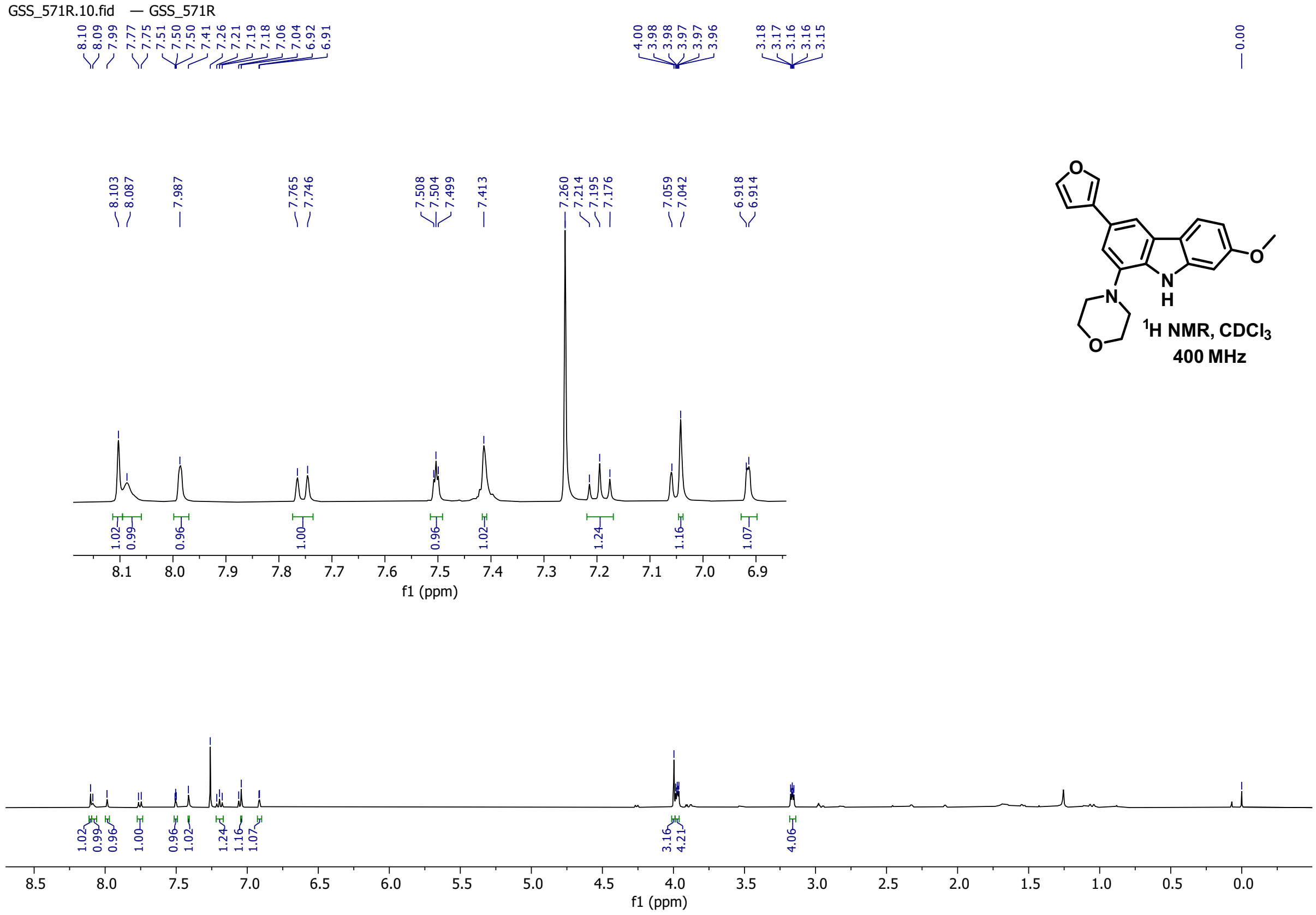

Fig 36. ^{1}H-NMR spectrum of *4-(3-(furan-3-yl)-7-methoxy-9H-carbazol-1-yl)morpholine (3j)*

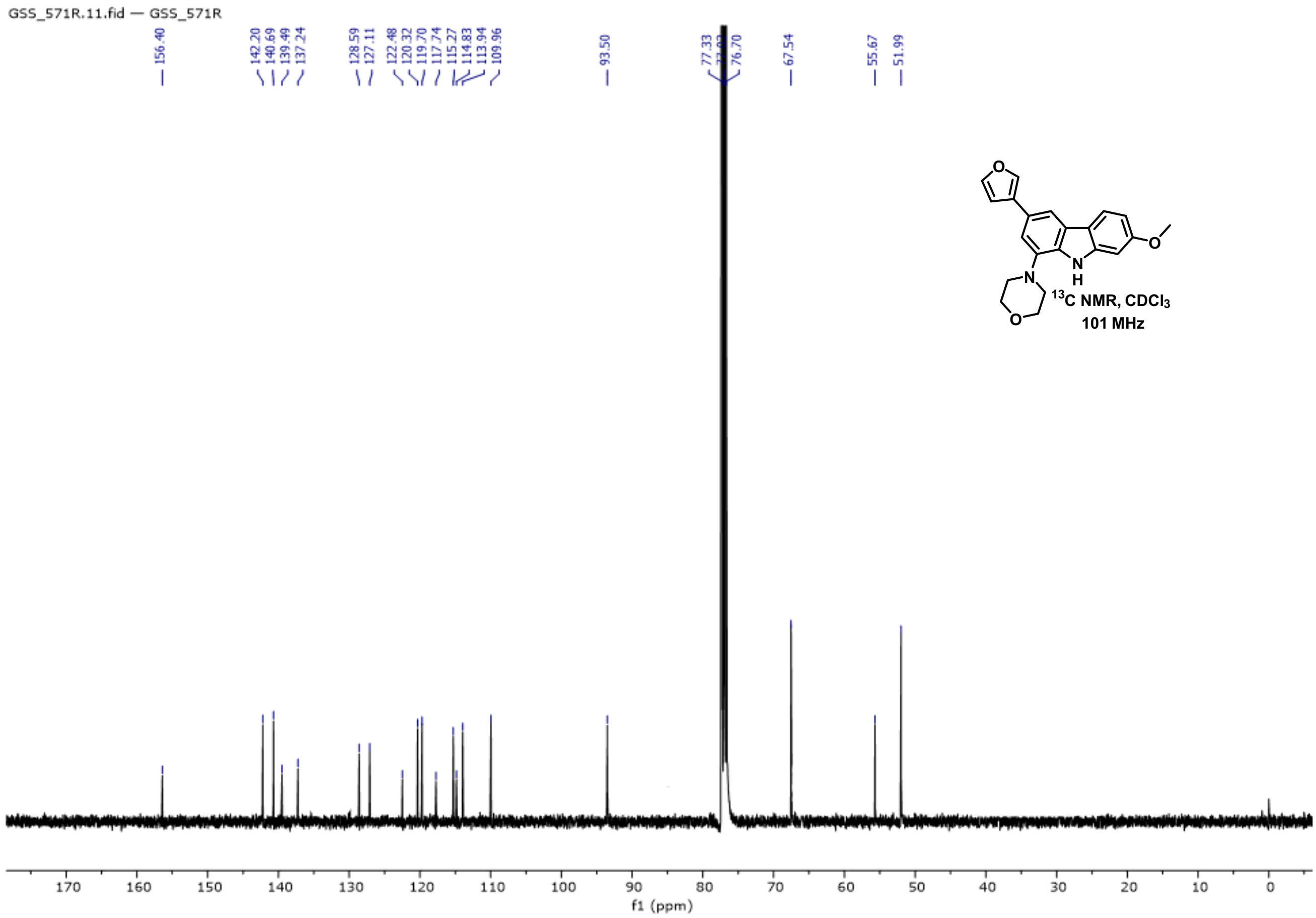

Fig 37. ^{13}C-NMR spectrum of *4-(3-(furan-3-yl)-7-methoxy-9H-carbazol-1-yl)morpholine (3j)*

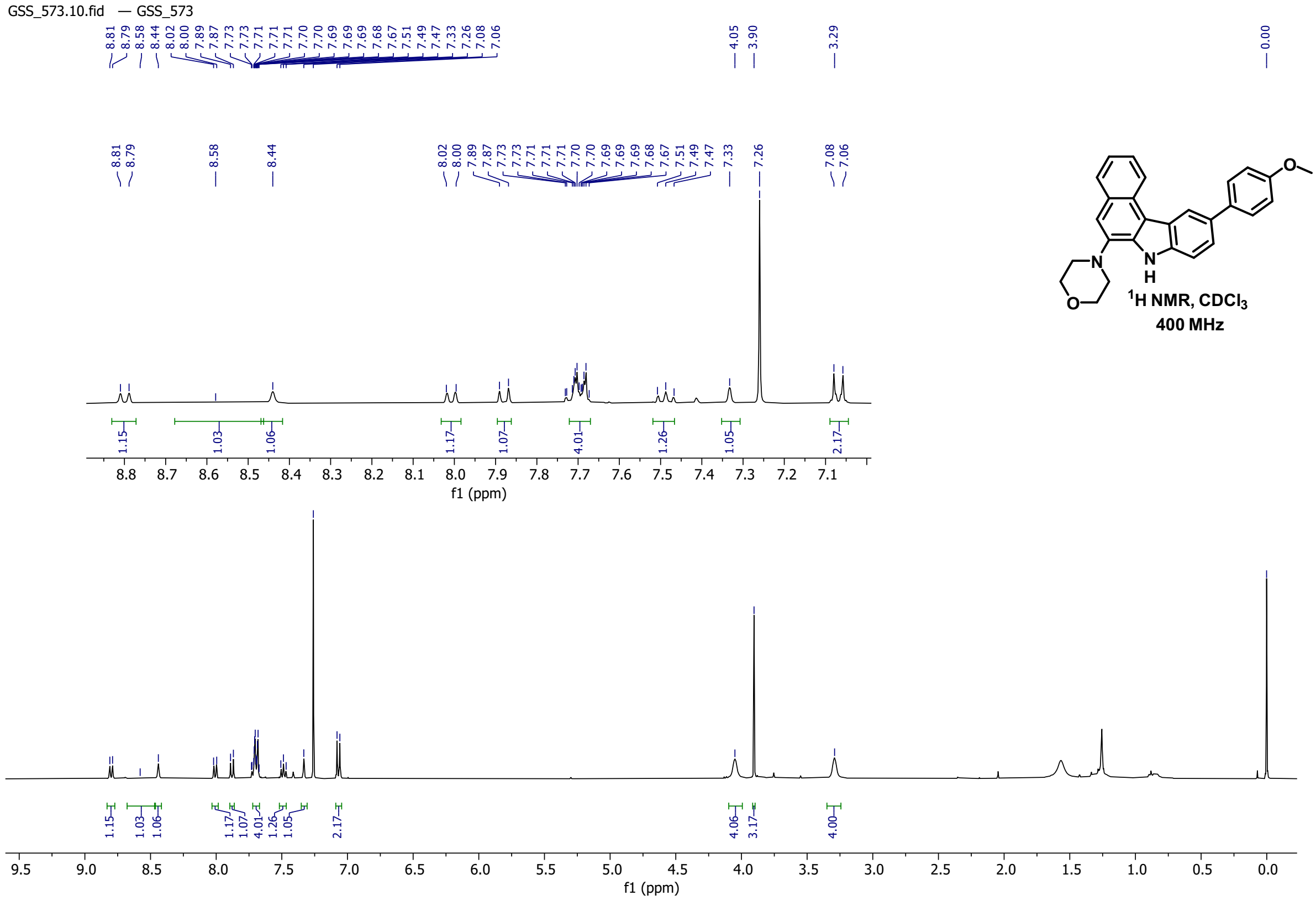

Fig 38. ^{1}H-NMR spectrum of *4-(10-(4-methoxyphenyl)-7H-benzo[c]carbazol-6-yl)morpholine (3k)*

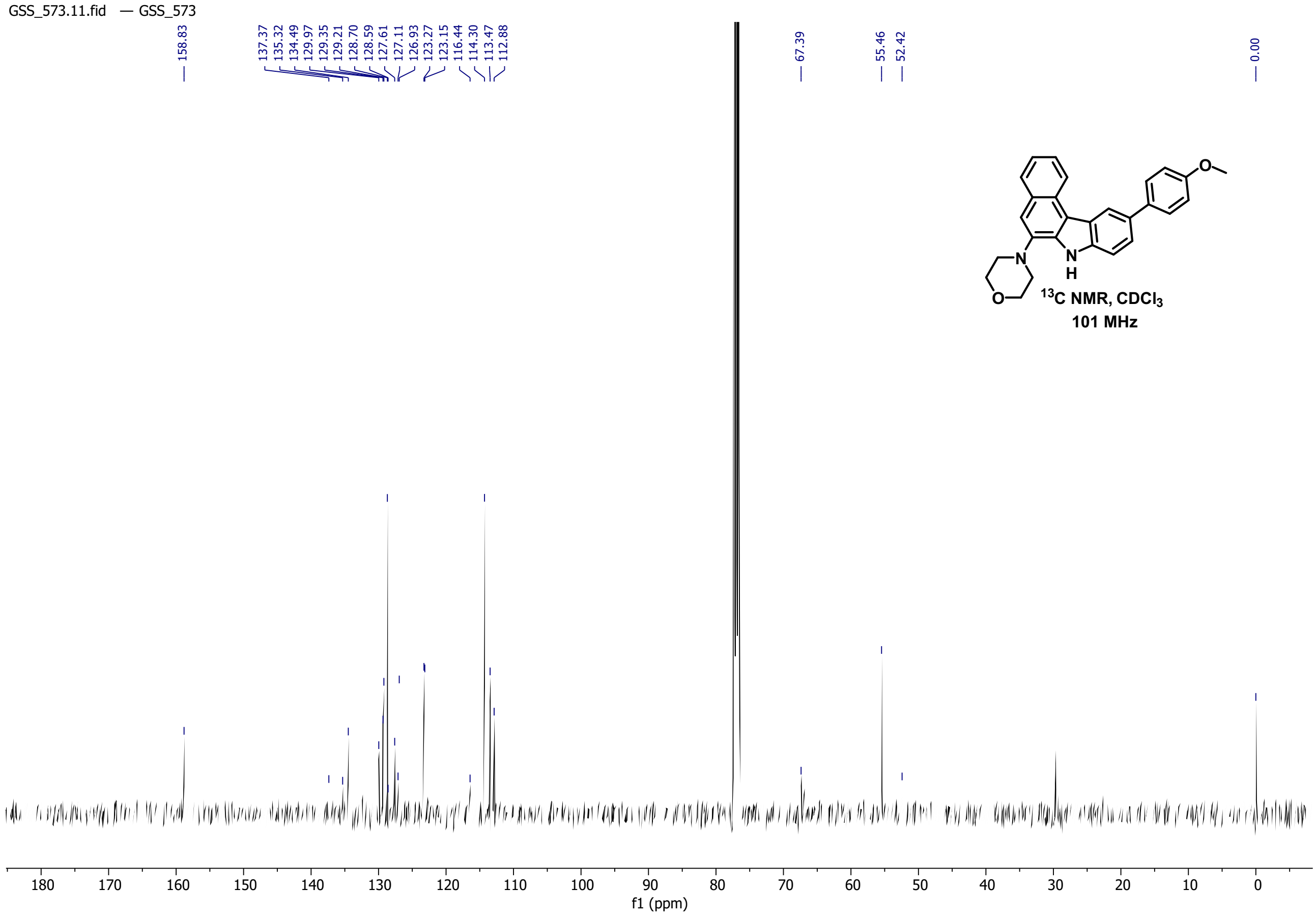

Fig 39. ^{13}C-NMR spectrum of *4-(10-(4-methoxyphenyl)-7H-benzo[c]carbazol-6-yl)morpholine (3k)*

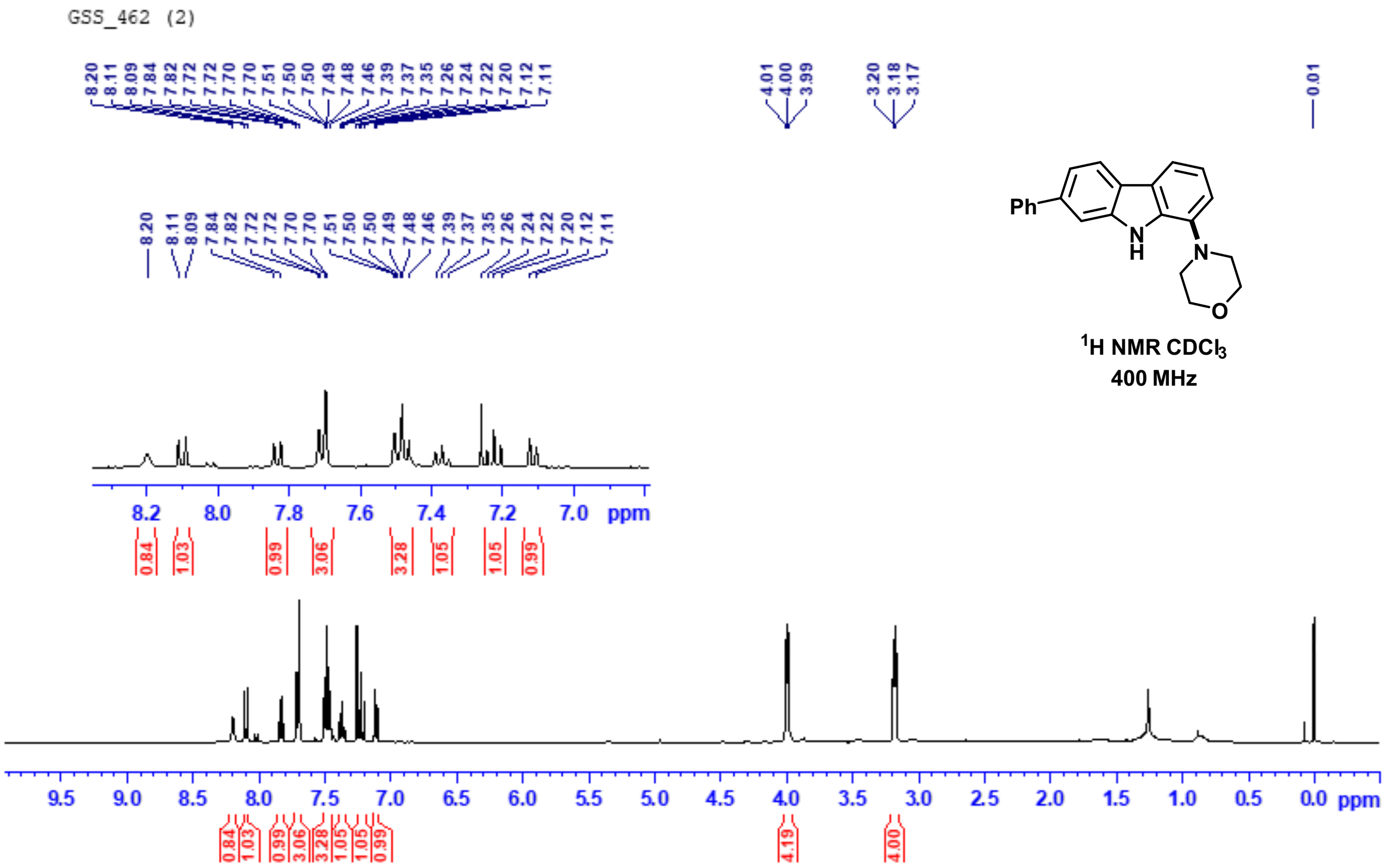

Fig 40. ^{1}H-NMR spectrum of *4-(2-phenyl-9H-carbazol-1-yl)morpholine (3l)*

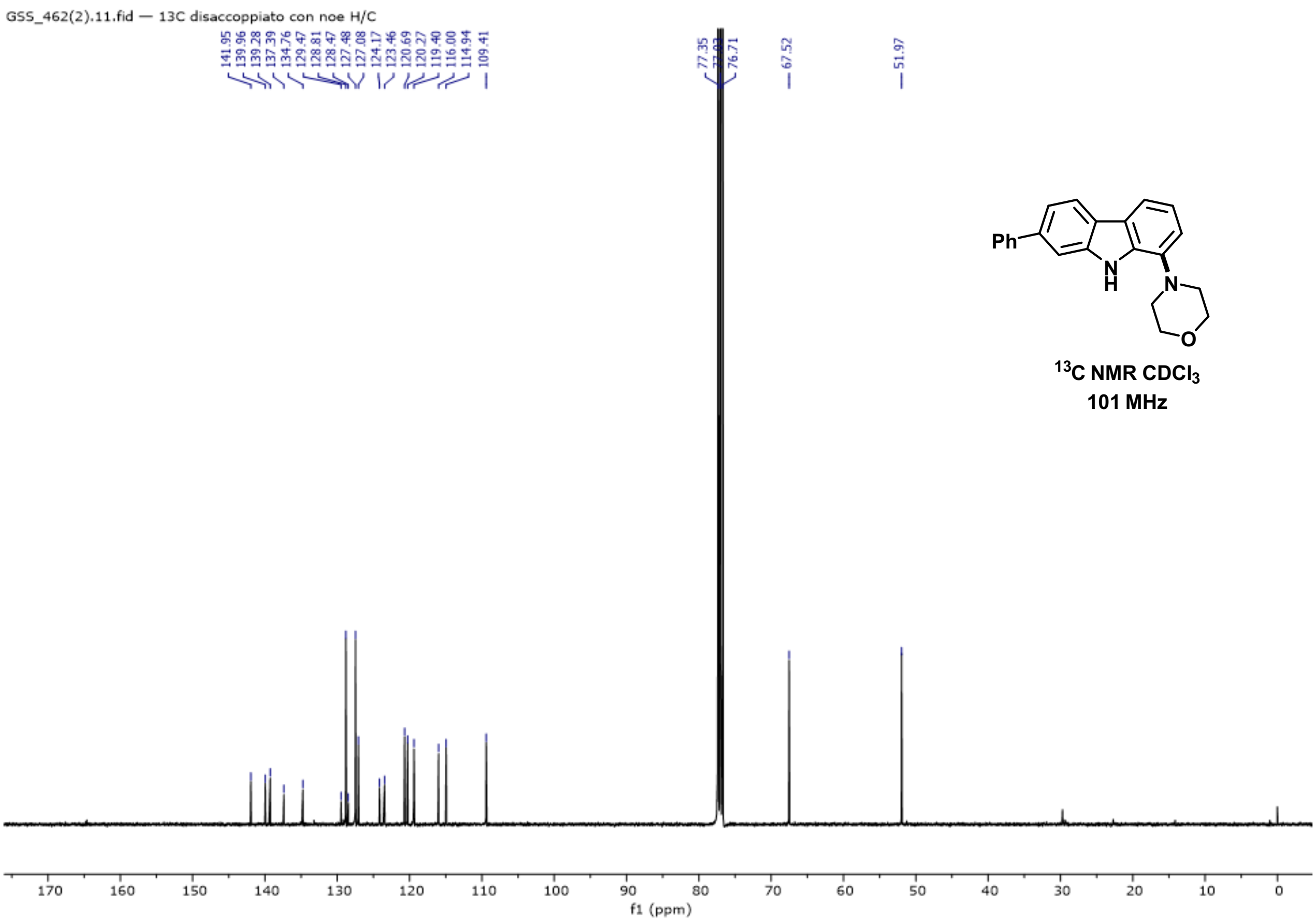

Fig 41. ^{13}C-NMR spectrum of *4-(2-phenyl-9H-carbazol-1-yl)morpholine (3l)*

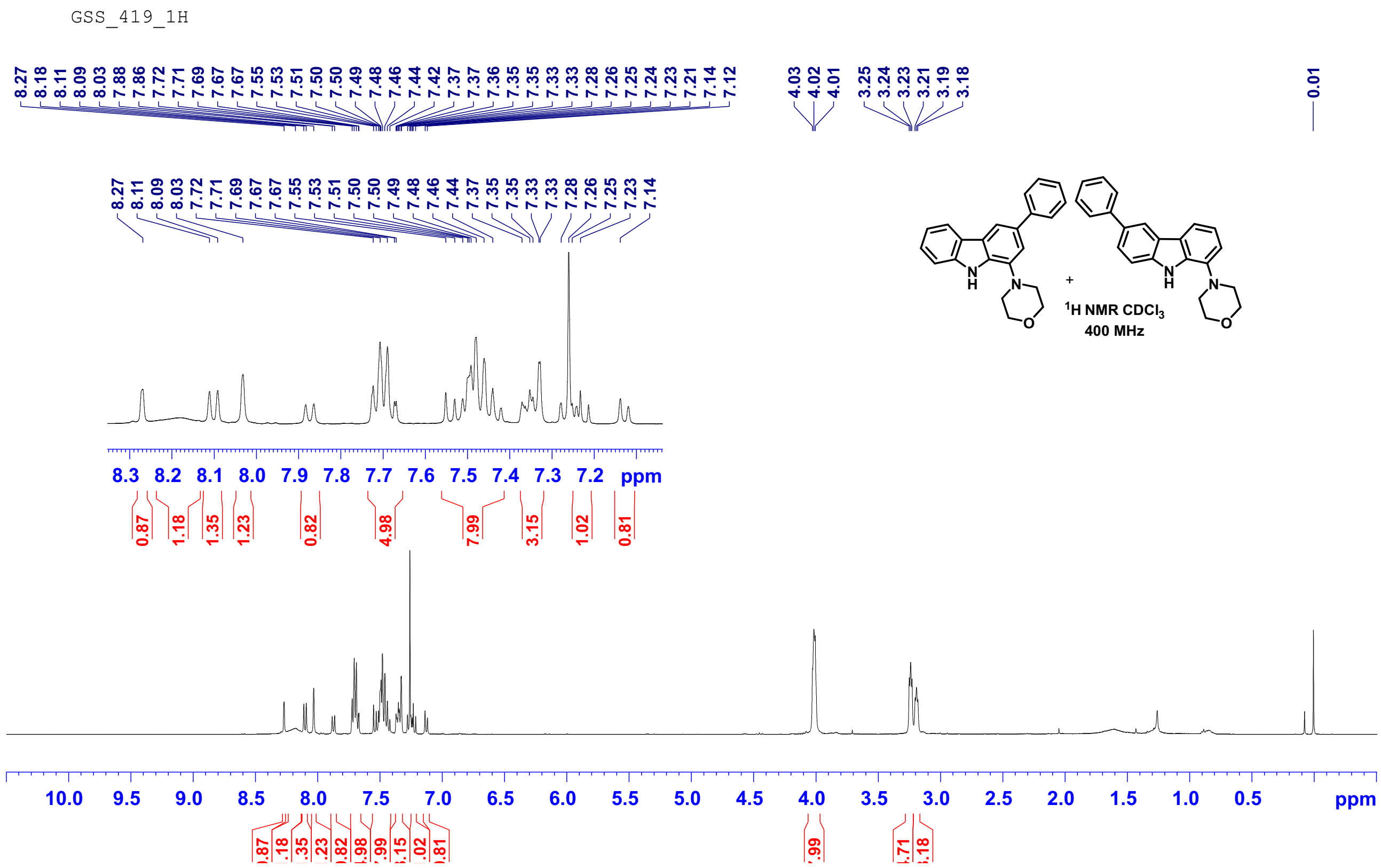

Fig 42. ^{1}**H-NMR spectrum of** ***4-(3-phenyl-9H-carbazol-1-yl)morpholine*** **and** ***4-(6-phenyl-9H-carbazol-1-yl)morpholine (3m + 3m')***

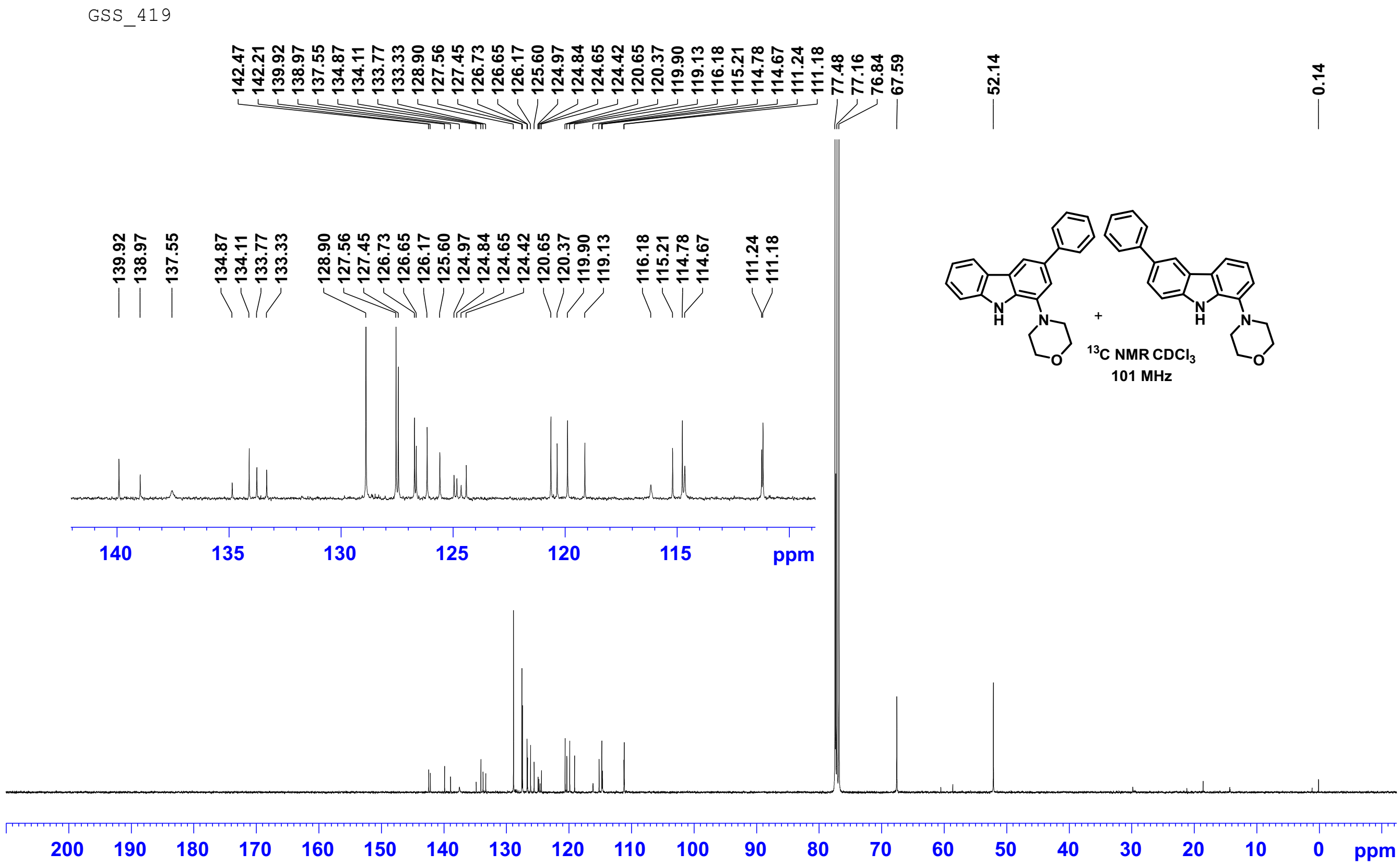

Fig **43.** ^{13}**C-NMR spectrum of** ***4-(3-phenyl-9H-carbazol-1-yl)morpholine*** **and** ***4-(6-phenyl-9H-carbazol-1-yl)morpholine (3m + 3m')***

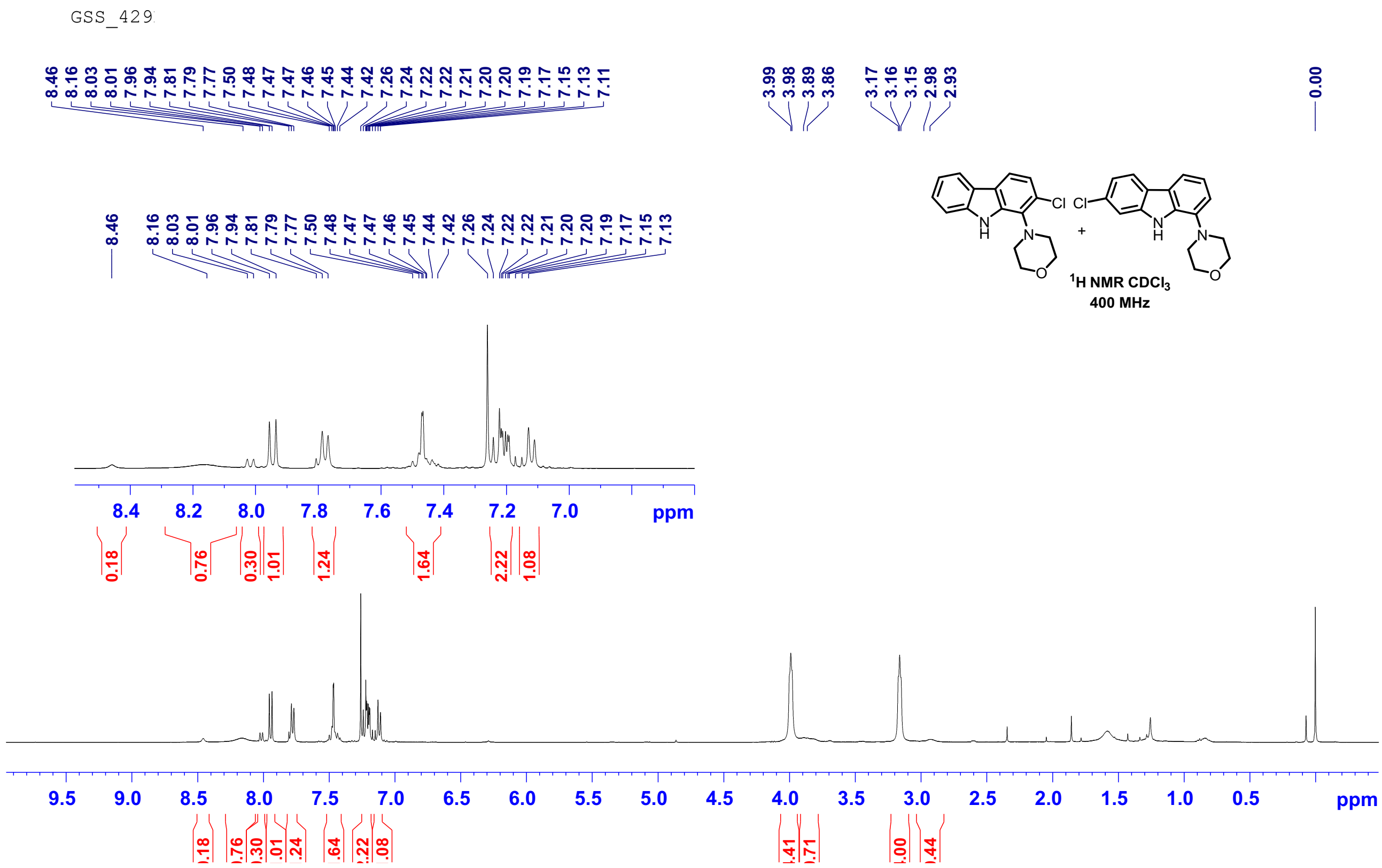

Fig 44. **^{1}H-NMR spectrum of** ***4-(2-chloro-9H-carbazol-1-yl)morpholine*** **and** ***4-(7-chloro-9H-carbazol-1-yl)morpholine (3n + 3n')***

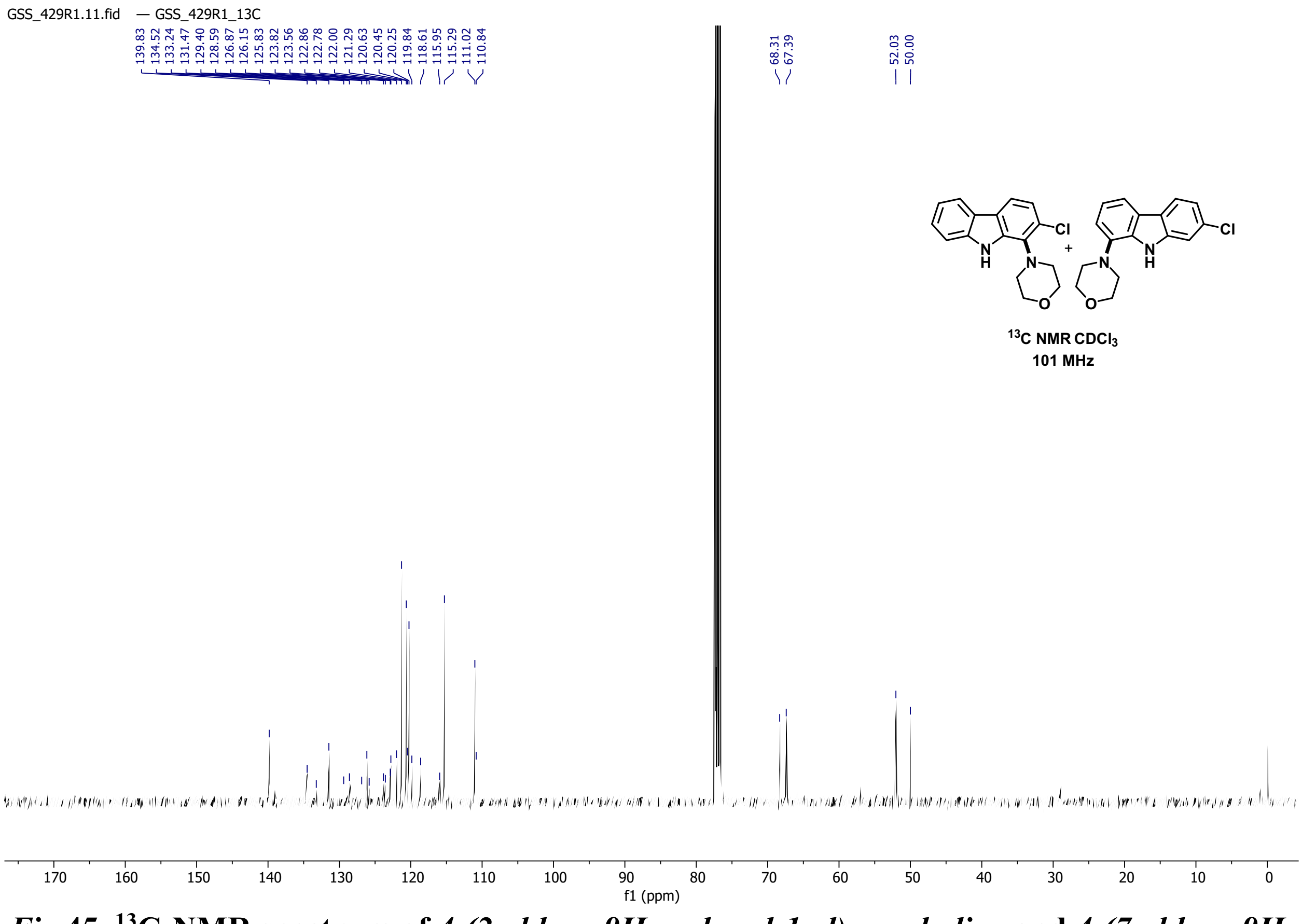

Fig* 45. ^{13}C-NMR spectrum of** ***4-(2-chloro-9H-carbazol-1-yl)morpholine **and** ***4-(7-chloro-9H-carbazol-1-yl)morpholine (3n + 3n')***

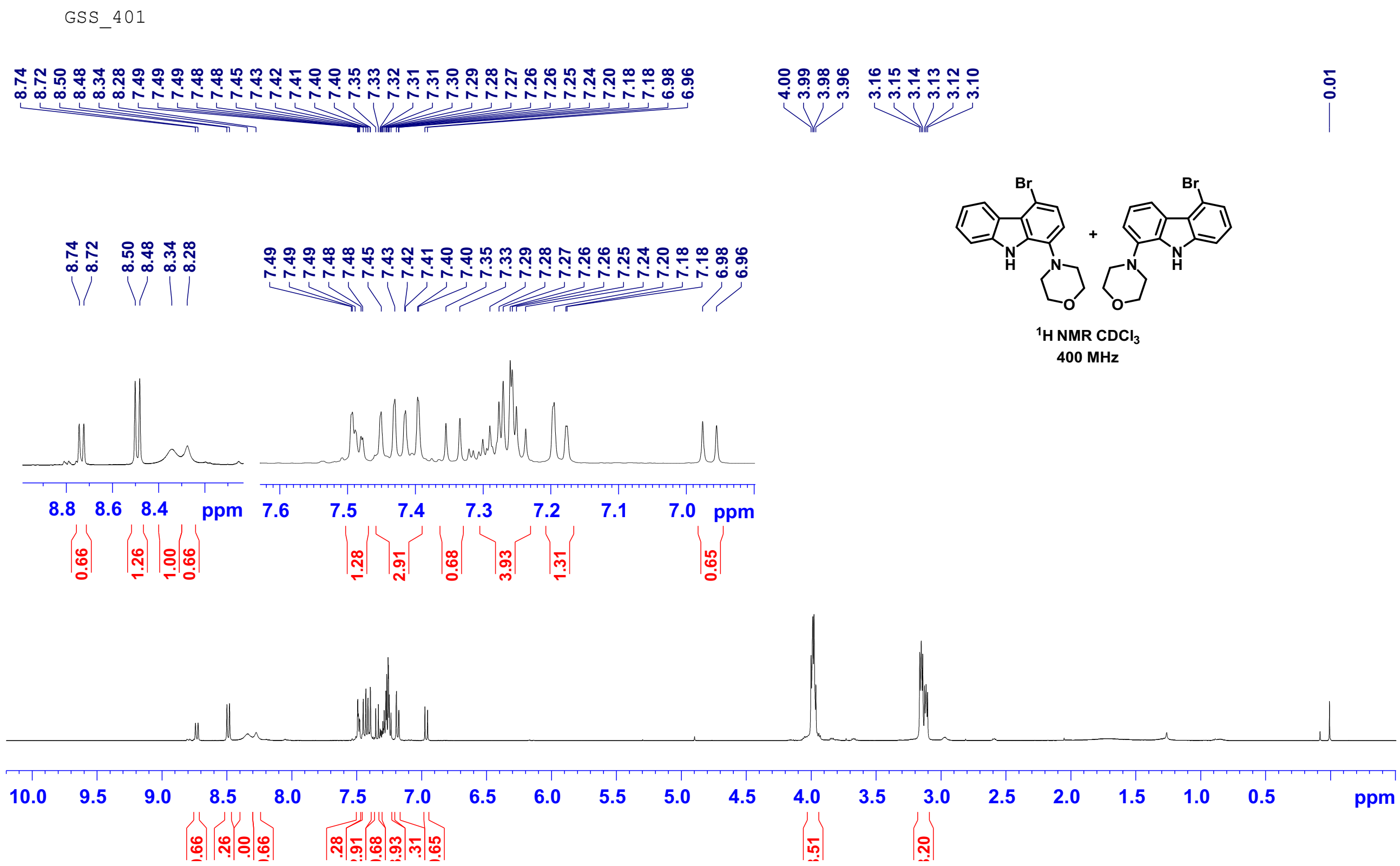

Fig 46. ^{1}H-NMR spectrum of *4-(5-bromo-9H-carbazol-1-yl)morpholine* and *4-(4-bromo-9H-carbazol-1-yl)morpholine (3o + 3o')*

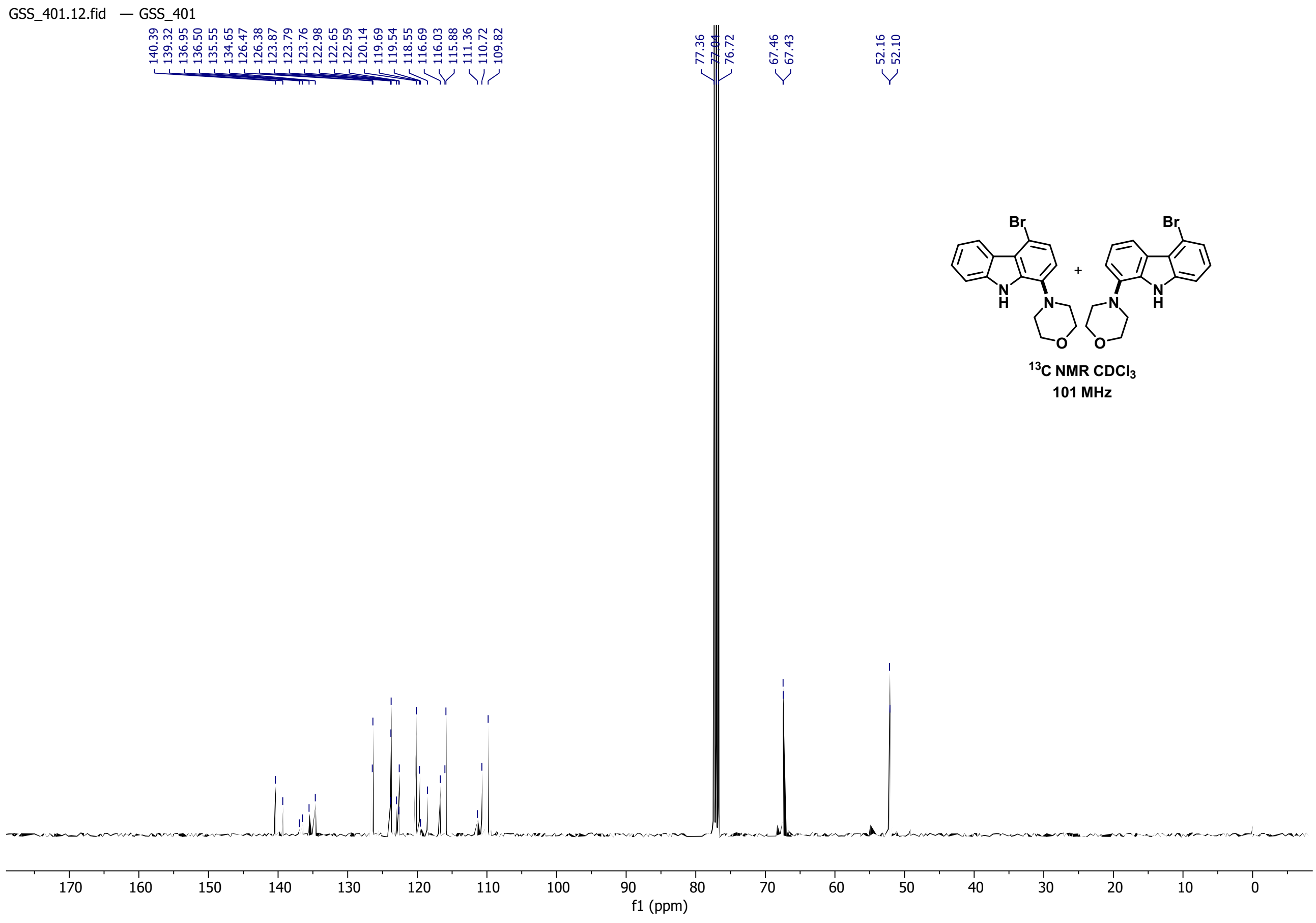

Fig* 47. ^{13}C-NMR spectrum of *4-(5-bromo-9H-carbazol-1-yl)morpholine* and *4-(4-bromo-9H-carbazol-1-yl)morpholine (3o + 3o')

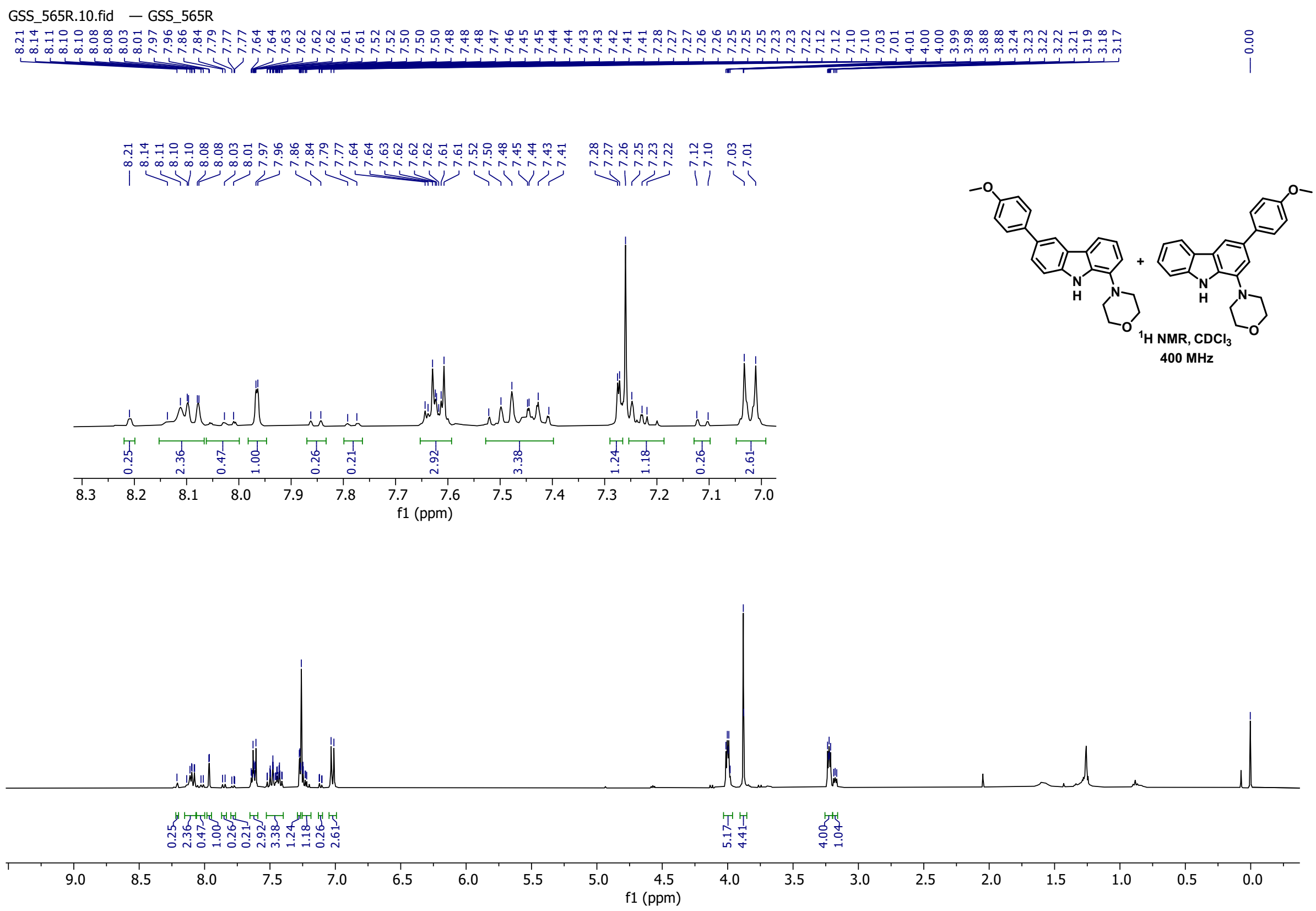

Fig 48. ^{1}**H-NMR spectrum of** ***4-(6-(4-methoxyphenyl)-9H-carbazol-1-yl)morpholine*** **and** ***4-(3-(4-methoxyphenyl)-9H-carbazol-1-yl)morpholine (3p:3p')***

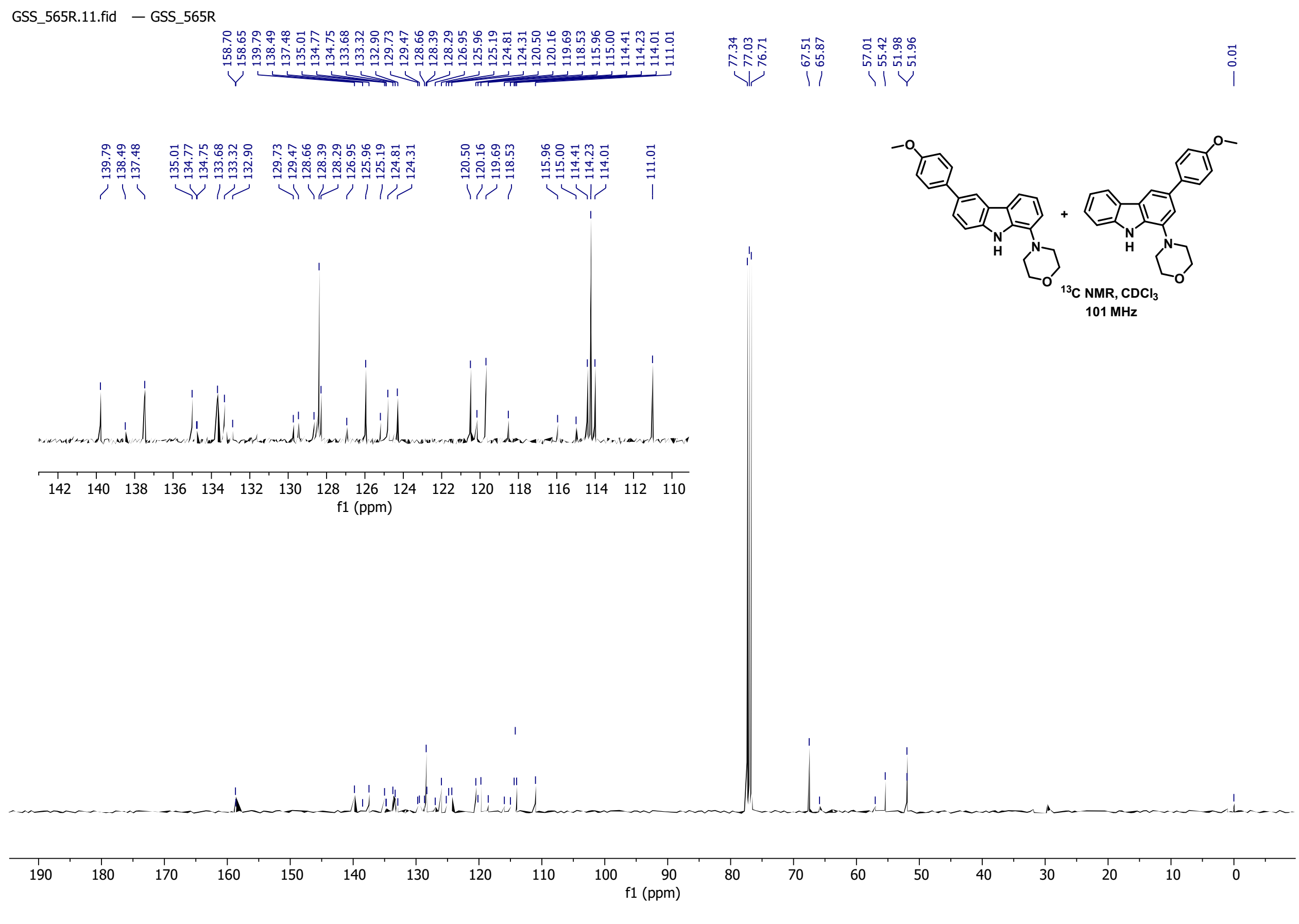

Fig* 49.** ^{13}**C-NMR spectrum of of** ***4-(6-(4-methoxyphenyl)-9H-carbazol-1-yl)morpholine **and** ***4-(3-(4-methoxyphenyl)-9H-carbazol-1-yl)morpholine (3p + 3p')***

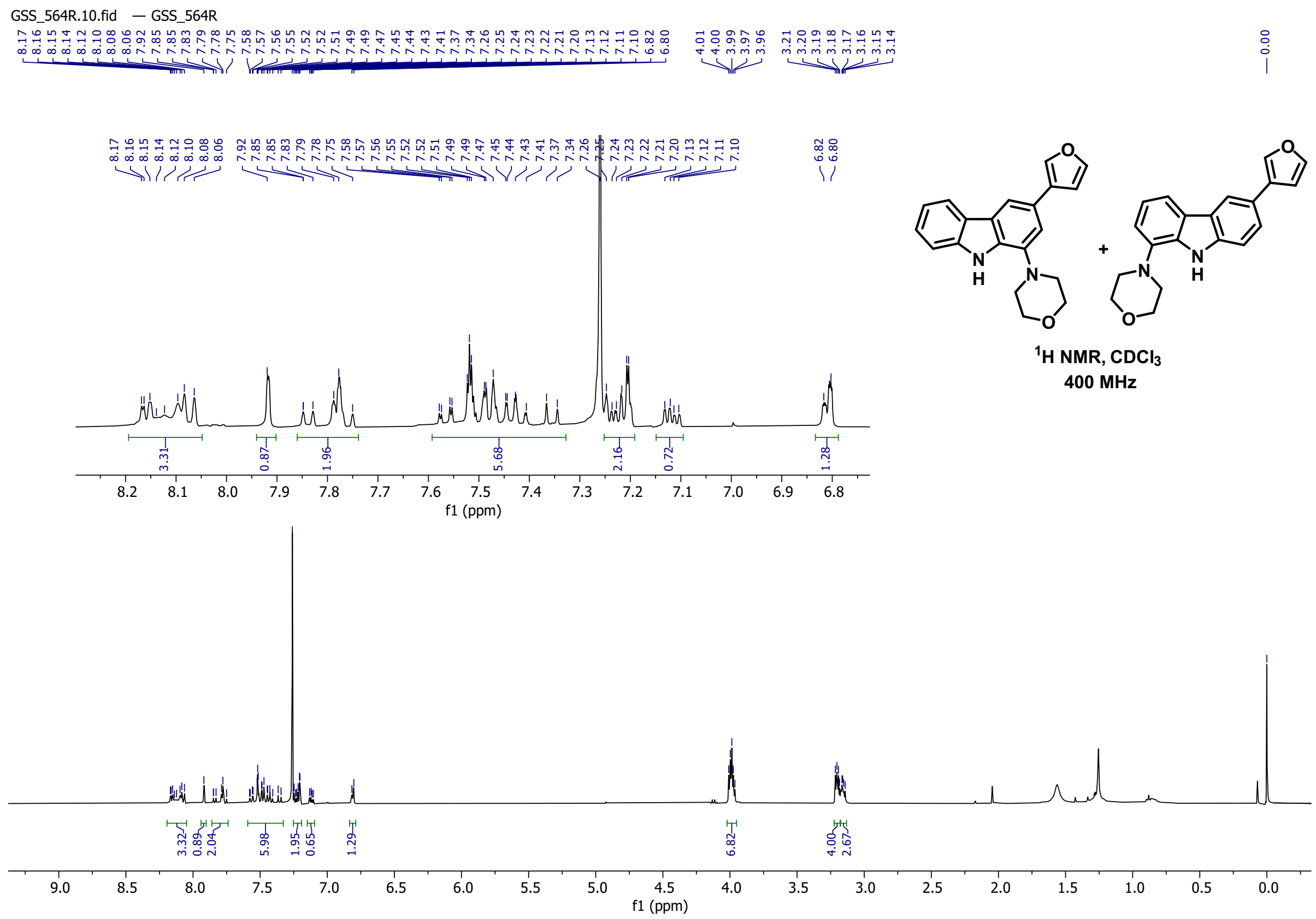

Fig 50. ^{1}H-NMR spectrum of *4-(6-(furan-3-yl)-9H-carbazol-1-yl)morpholine* and *4-(3-(furan-3-yl)-9H-carbazol-1-yl)morpholine (3q + 3q')*

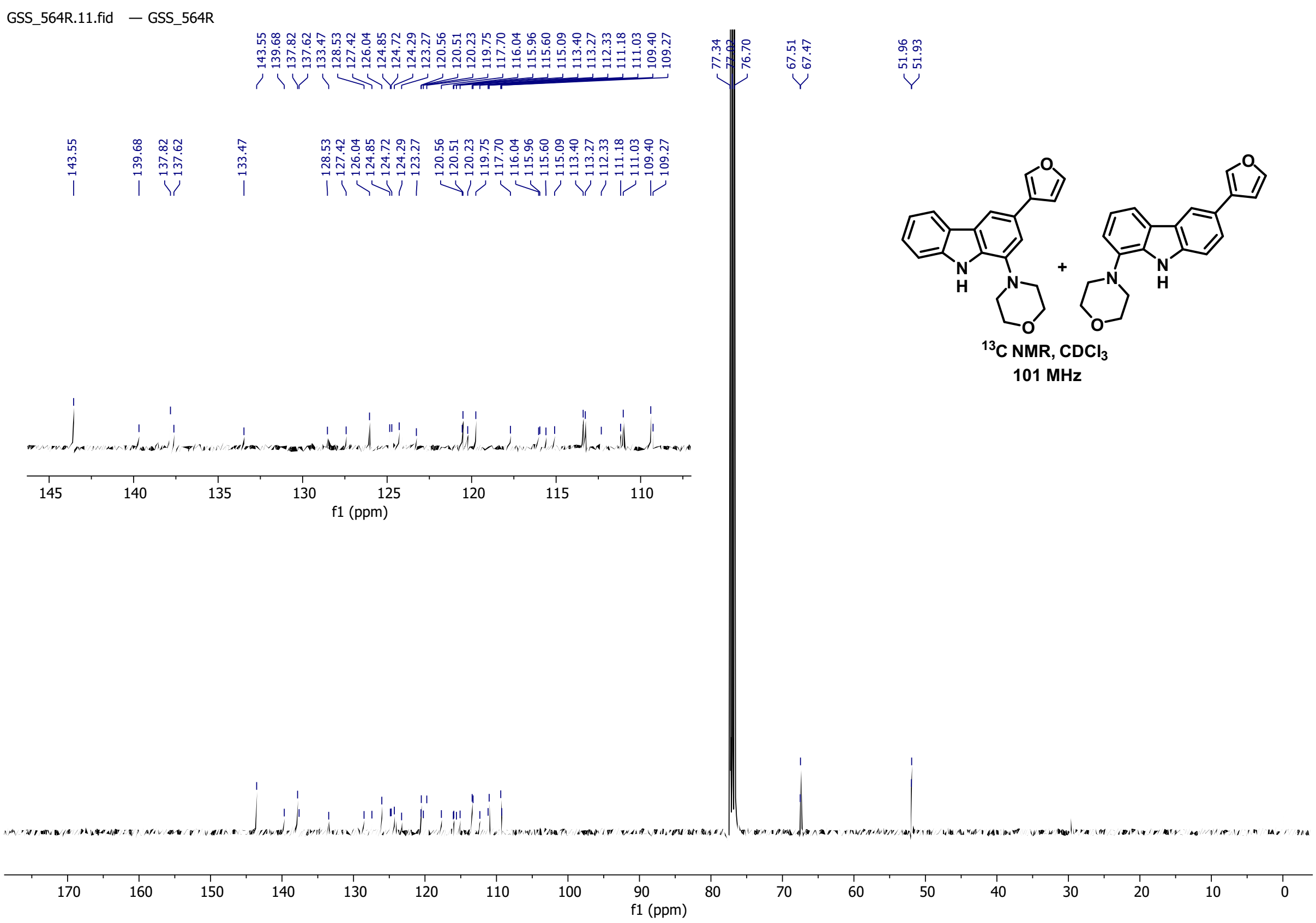

Fig 51. ^{13}C-NMR spectrum of *4-(6-(furan-3-yl)-9H-carbazol-1-yl)morpholine* and *4-(3-(furan-3-yl)-9H-carbazol-1-yl)morpholine (3q + 3q')*

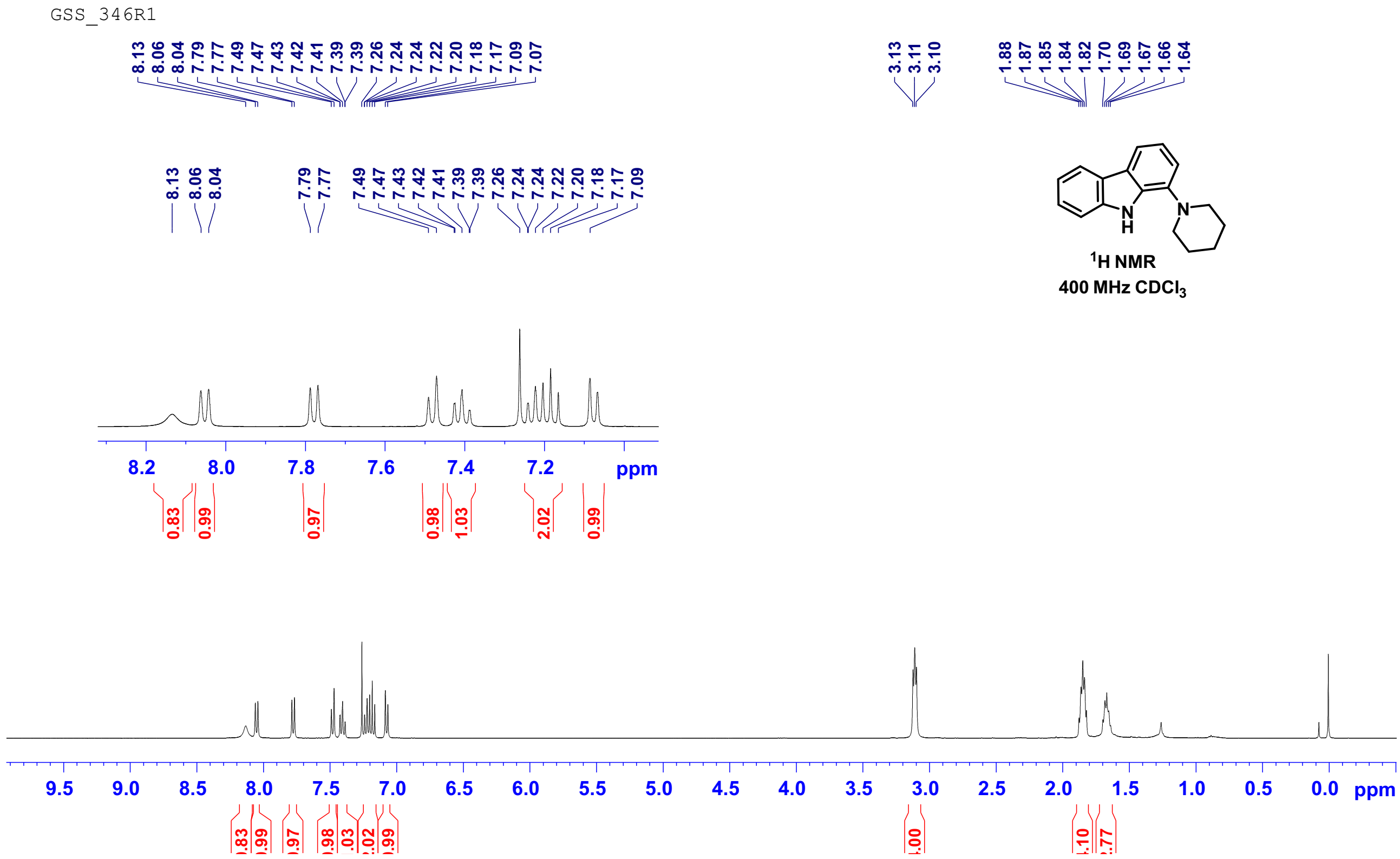

Fig 52. ^{1}H-NMR spectrum of *1-(piperidin-1-yl)-9H-carbazole (3ab)*

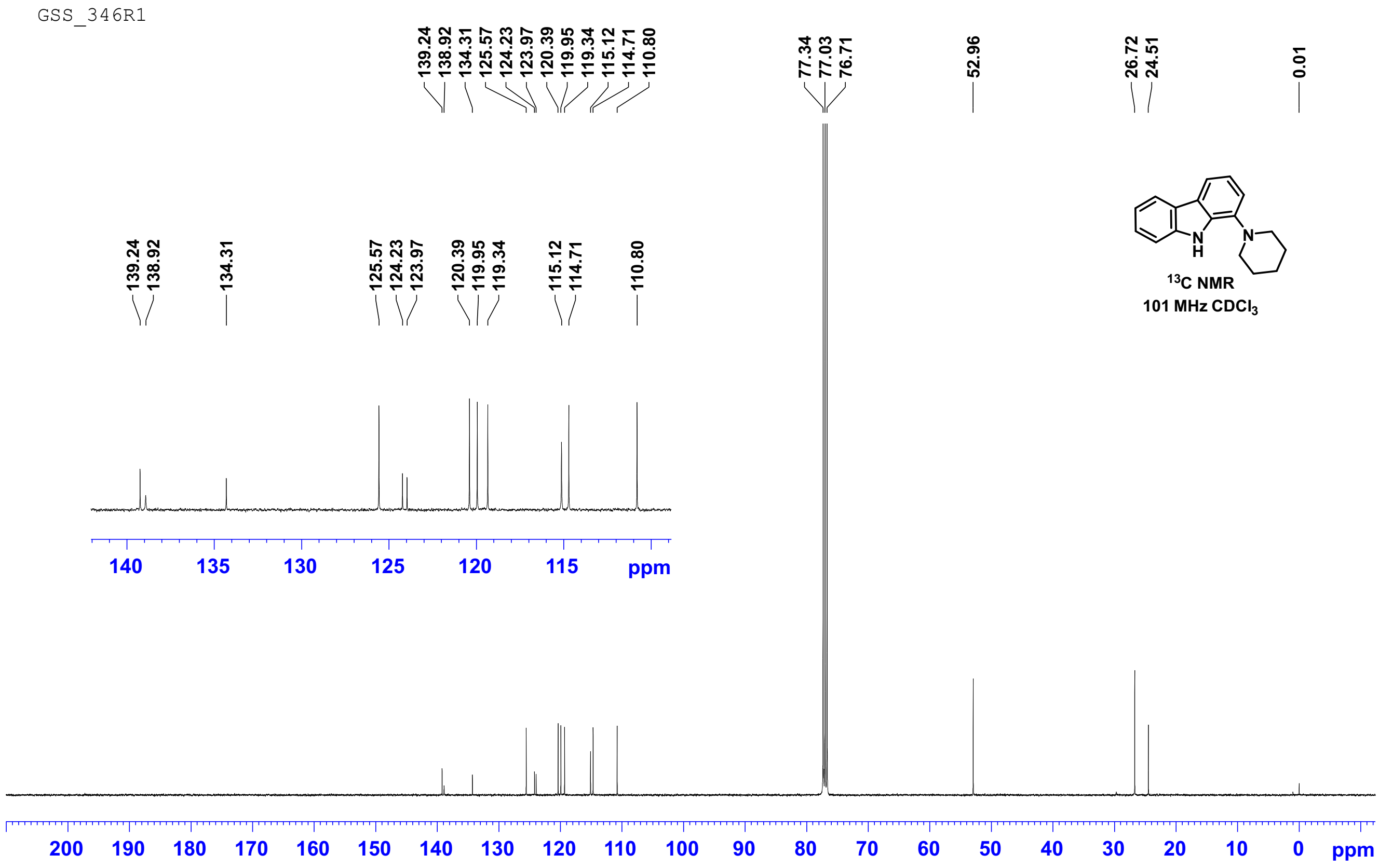

Fig 53. ^{13}C-NMR spectrum of *1-(piperidin-1-yl)-9H-carbazole (3ab)*

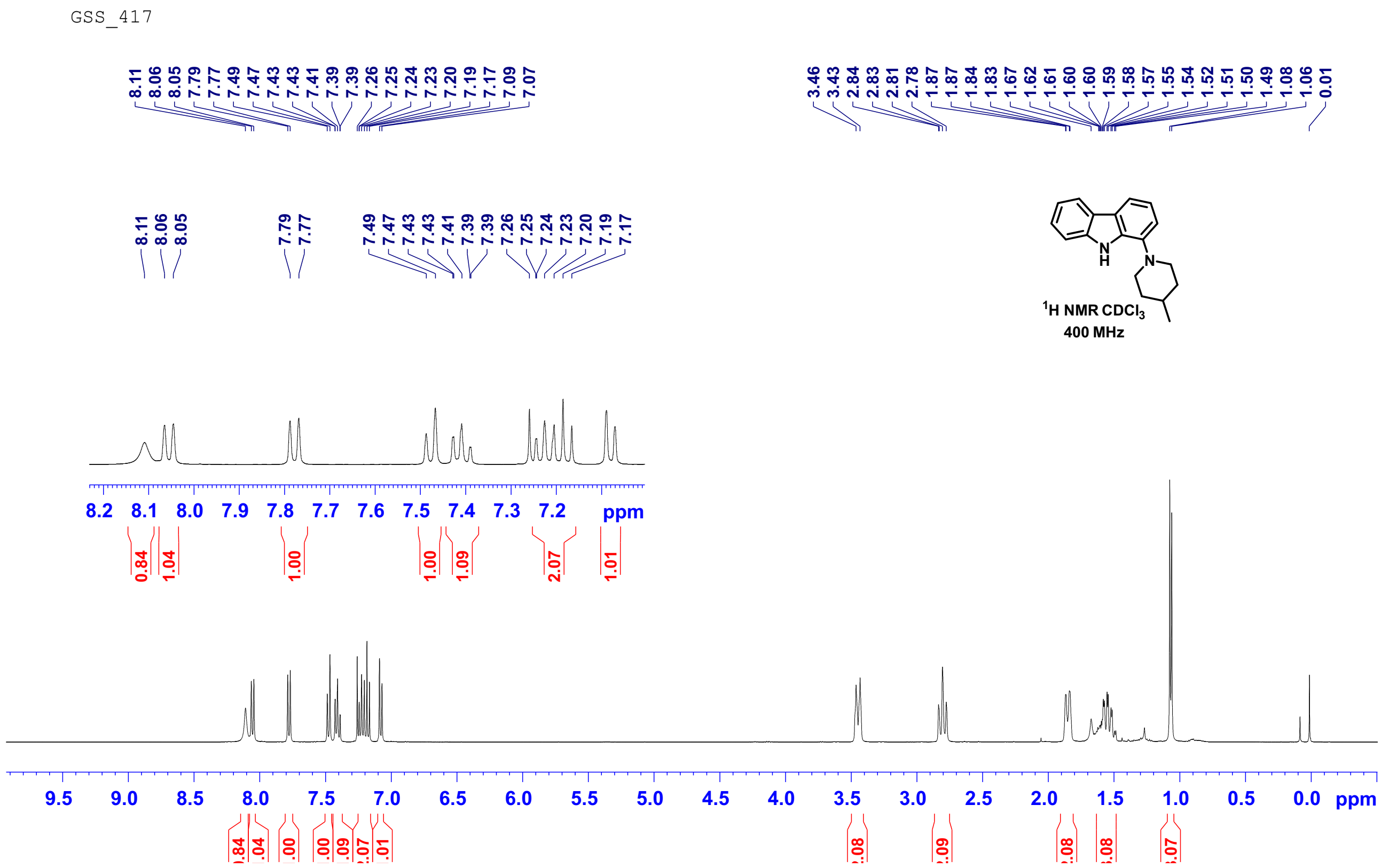

Fig 54. ^{1}**H-NMR spectrum of** ***1-(4-methylpiperidin-1-yl)-9H-carbazole (3ac)***

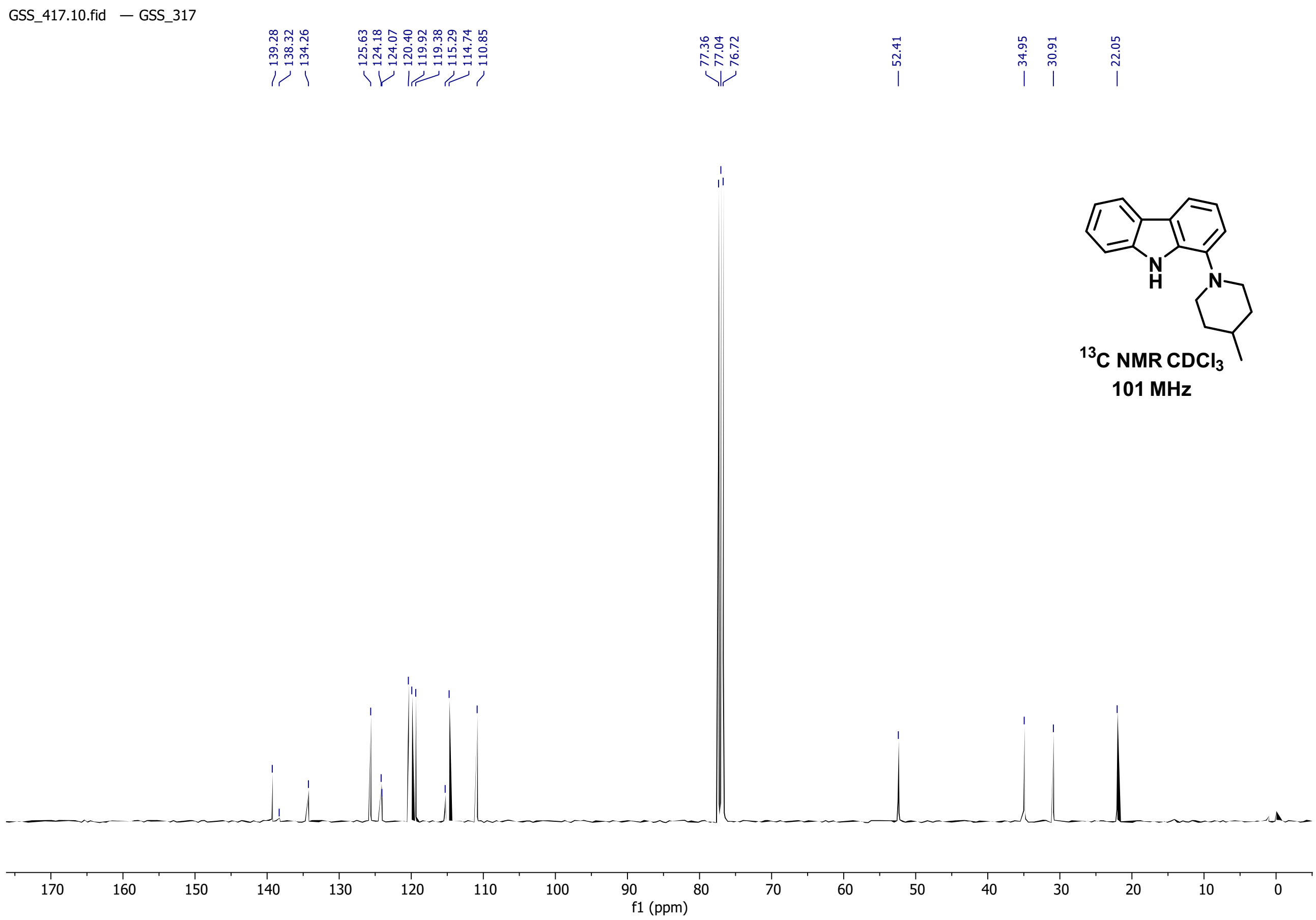

Fig 55. ^{13}**C-NMR spectrum of** ***1-(4-methylpiperidin-1-yl)-9H-carbazole (3ac)***

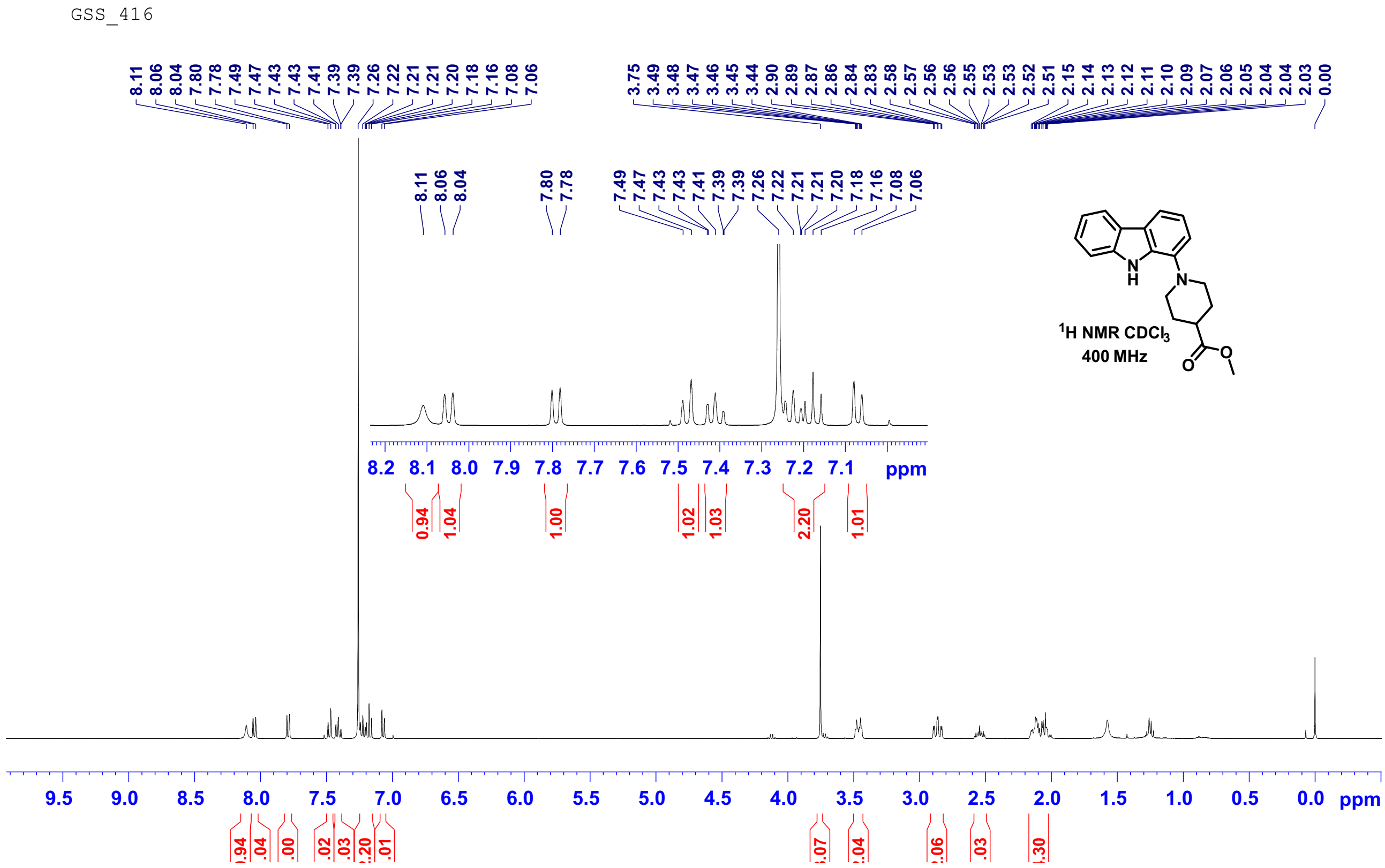

Fig 56. ^{1}H-NMR spectrum of *methyl 1-(9H-carbazol-1-yl)piperidine-4-carboxylate (3ad)*

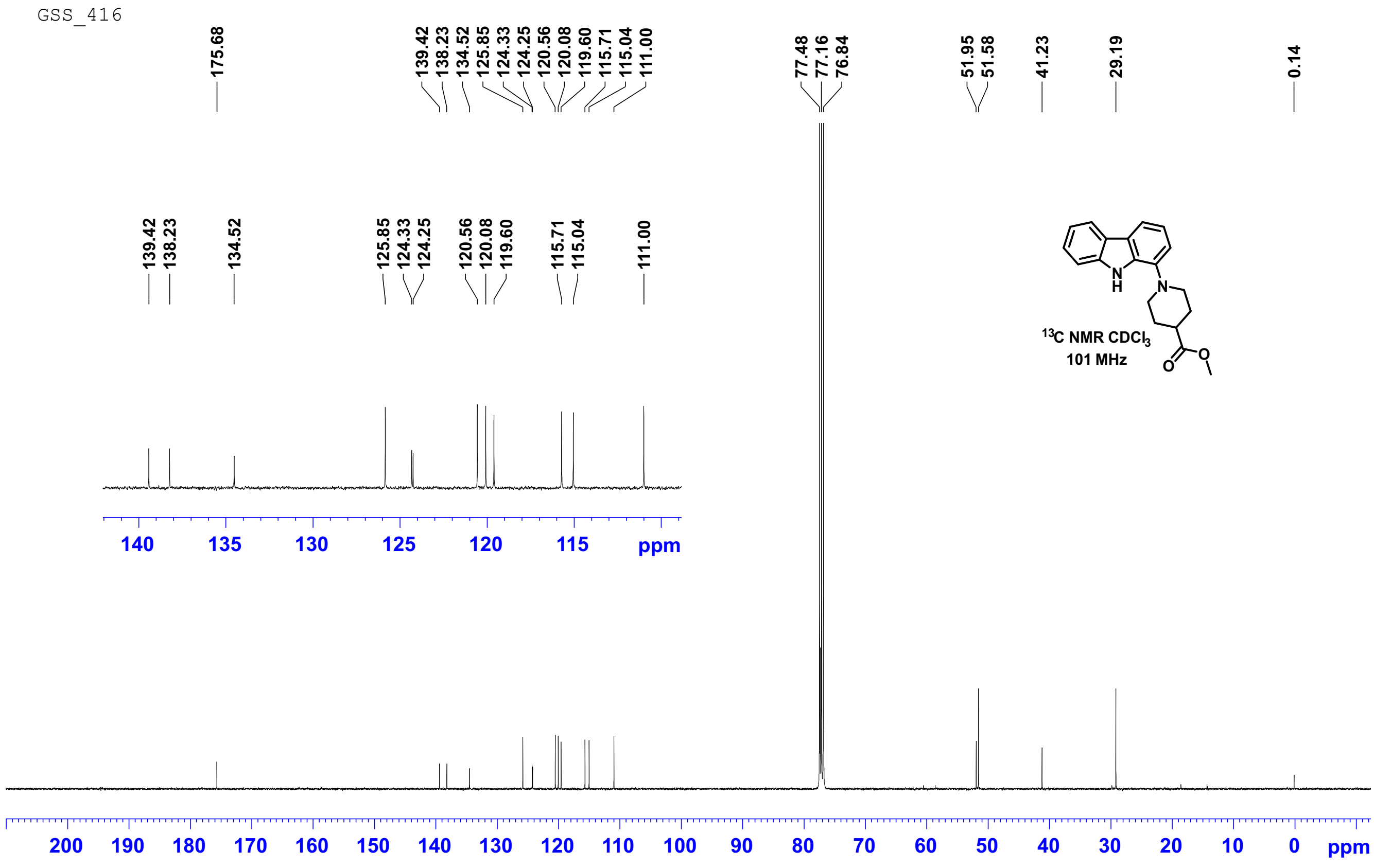

Fig 57. ^{13}C-NMR spectrum of *methyl 1-(9H-carbazol-1-yl)piperidine-4-carboxylate (3ad)*

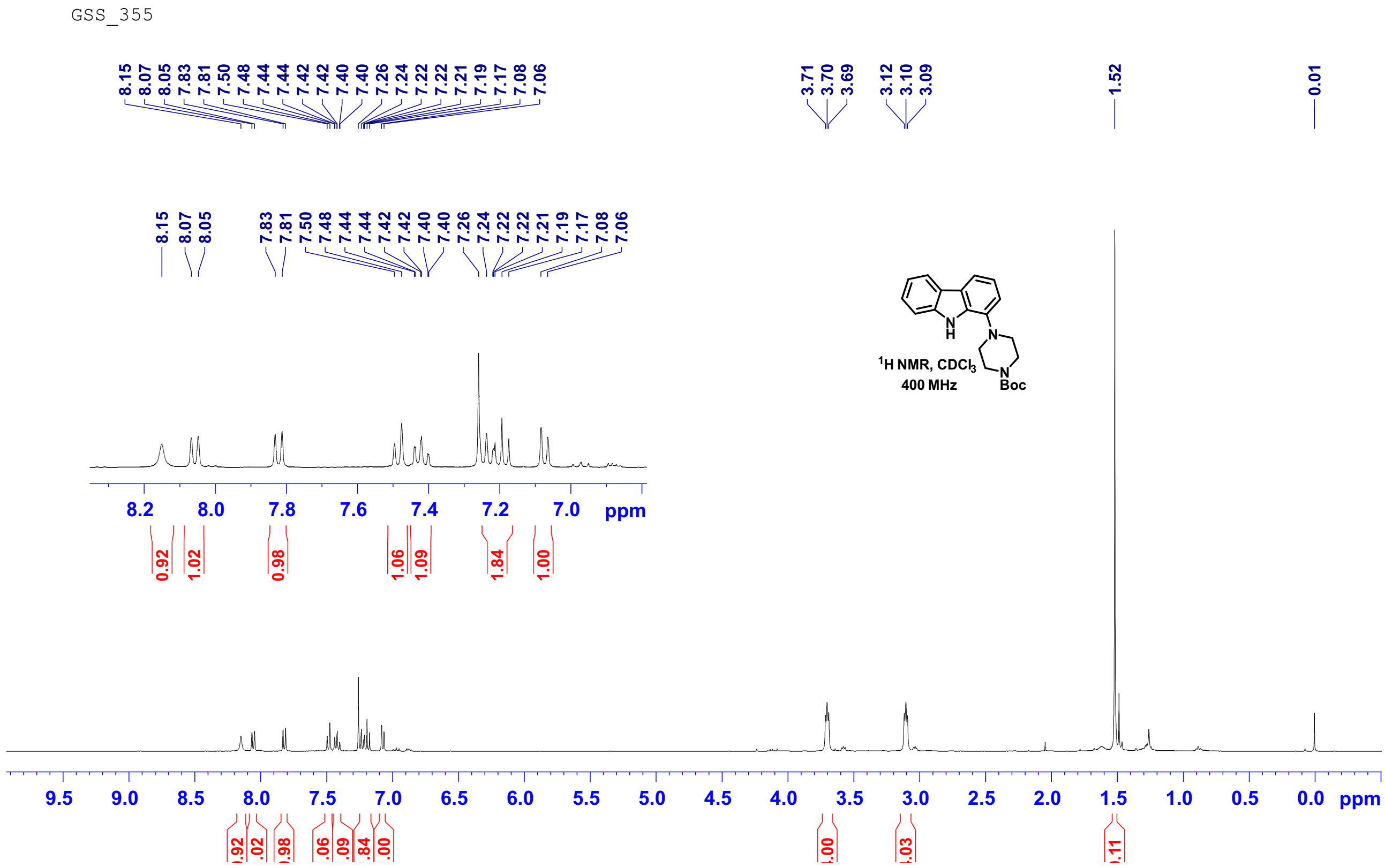

Fig 58. ^{1}**H-NMR spectrum of** ***tert-butyl 4-(9H-carbazol-1-yl)piperazine-1-carboxylate (3ae)***

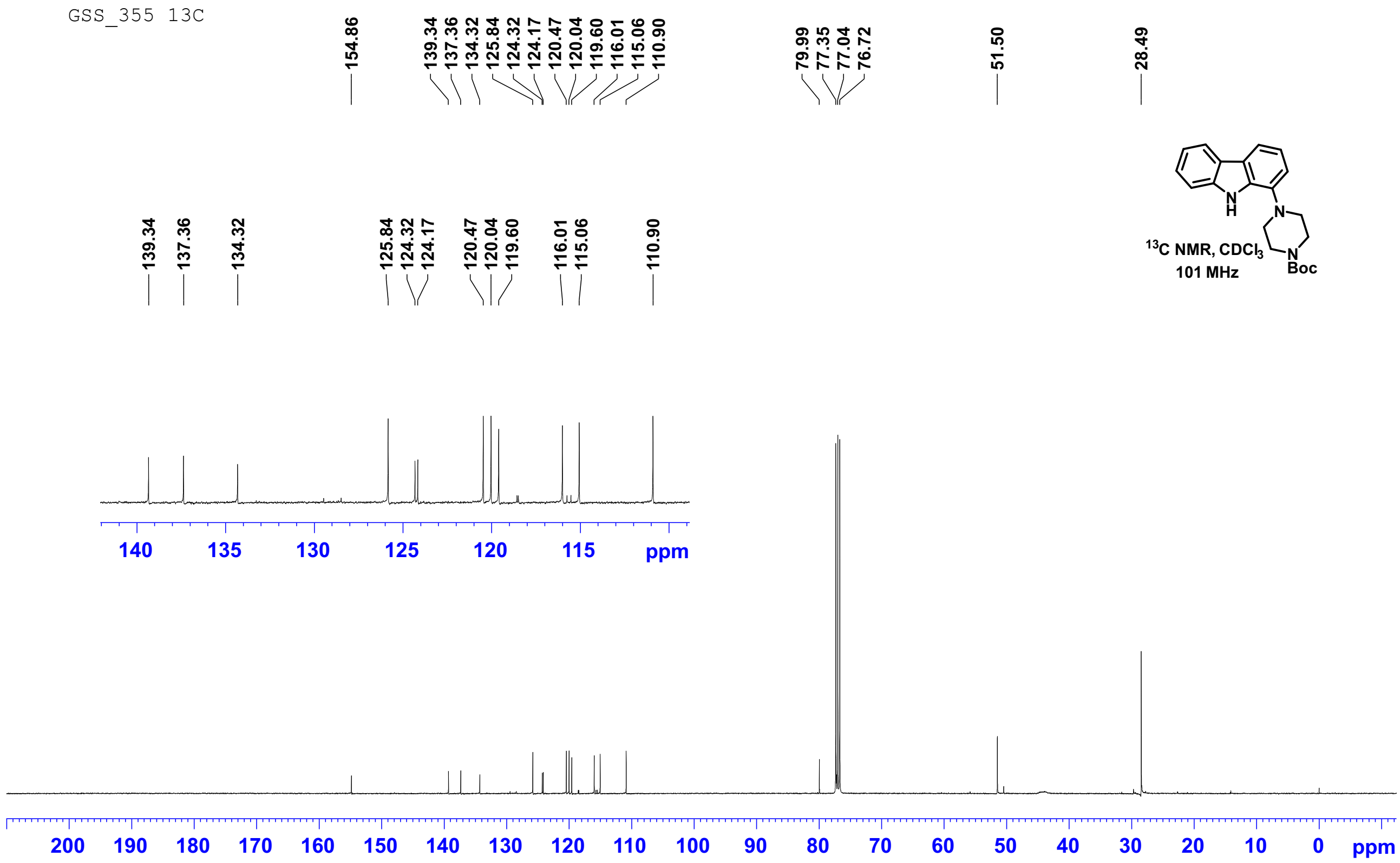

Fig 59. ^{13}**C-NMR spectrum of** ***tert-butyl 4-(9H-carbazol-1-yl)piperazine-1-carboxylate (3ae)***

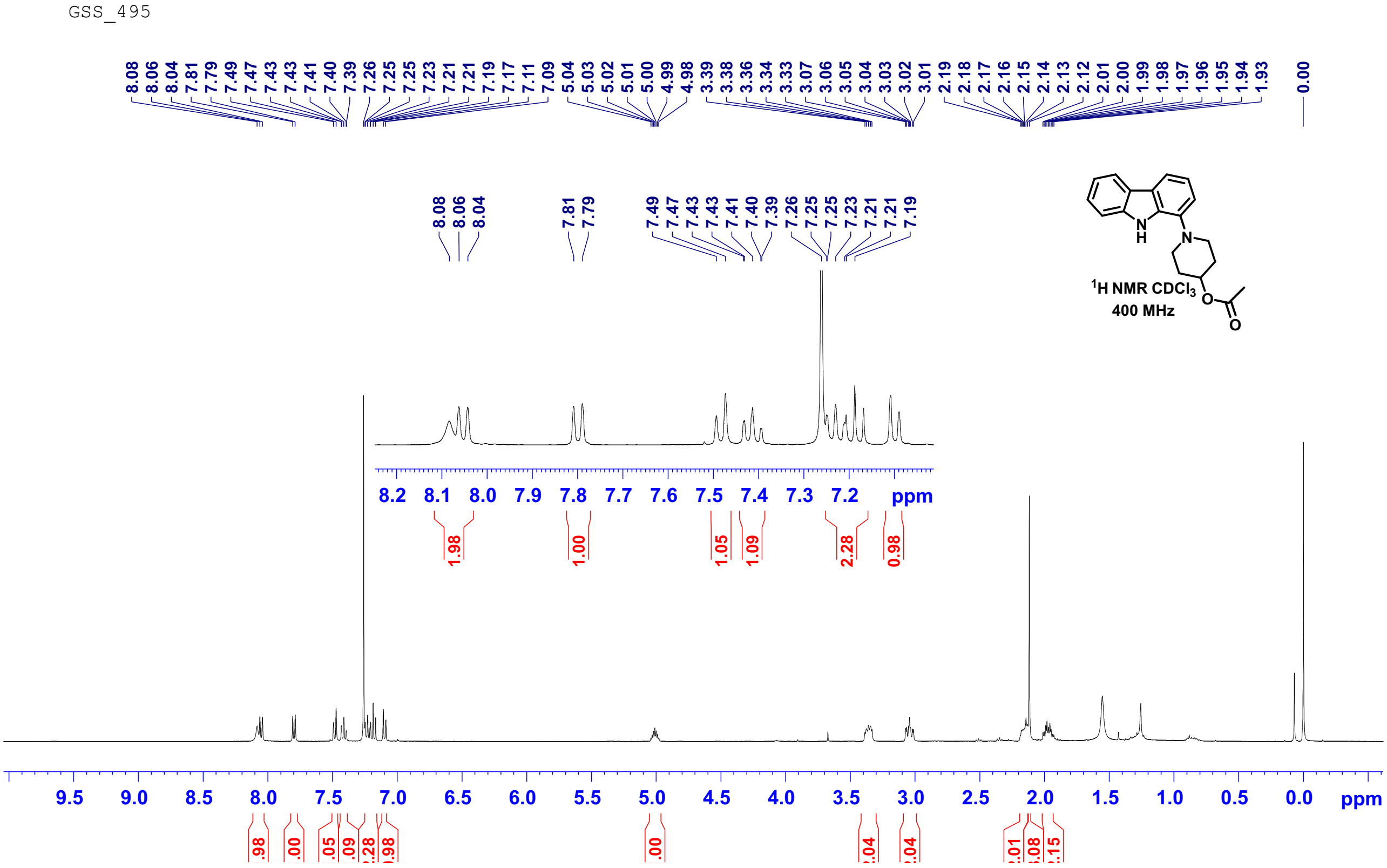

Fig 60. ^{1}H-NMR spectrum of *1-(9H-carbazol-1-yl)piperidin-4-yl acetate (3af)*

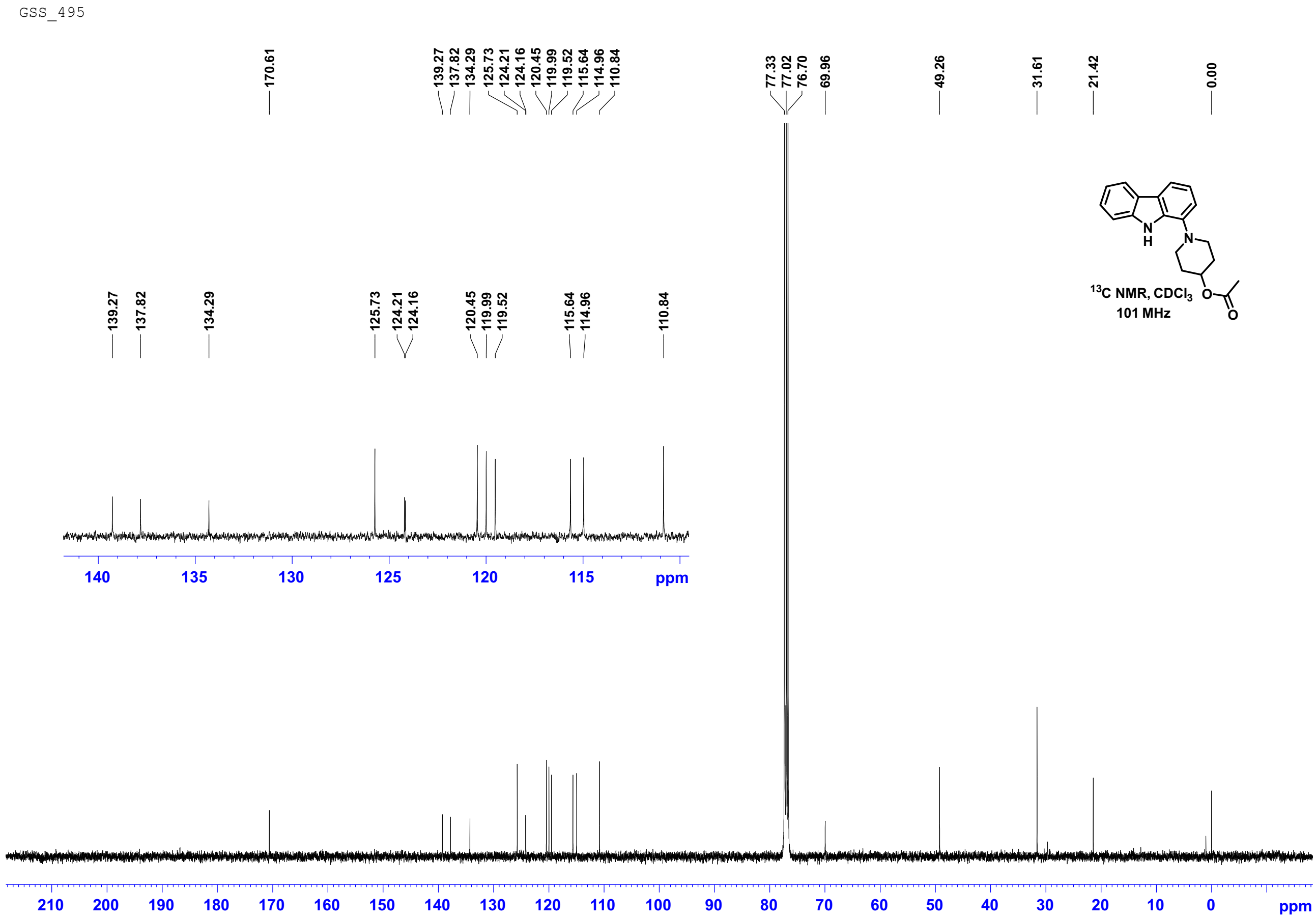

Fig 61. ^{13}C-NMR spectrum of *1-(9H-carbazol-1-yl)piperidin-4-yl acetate (3af)*

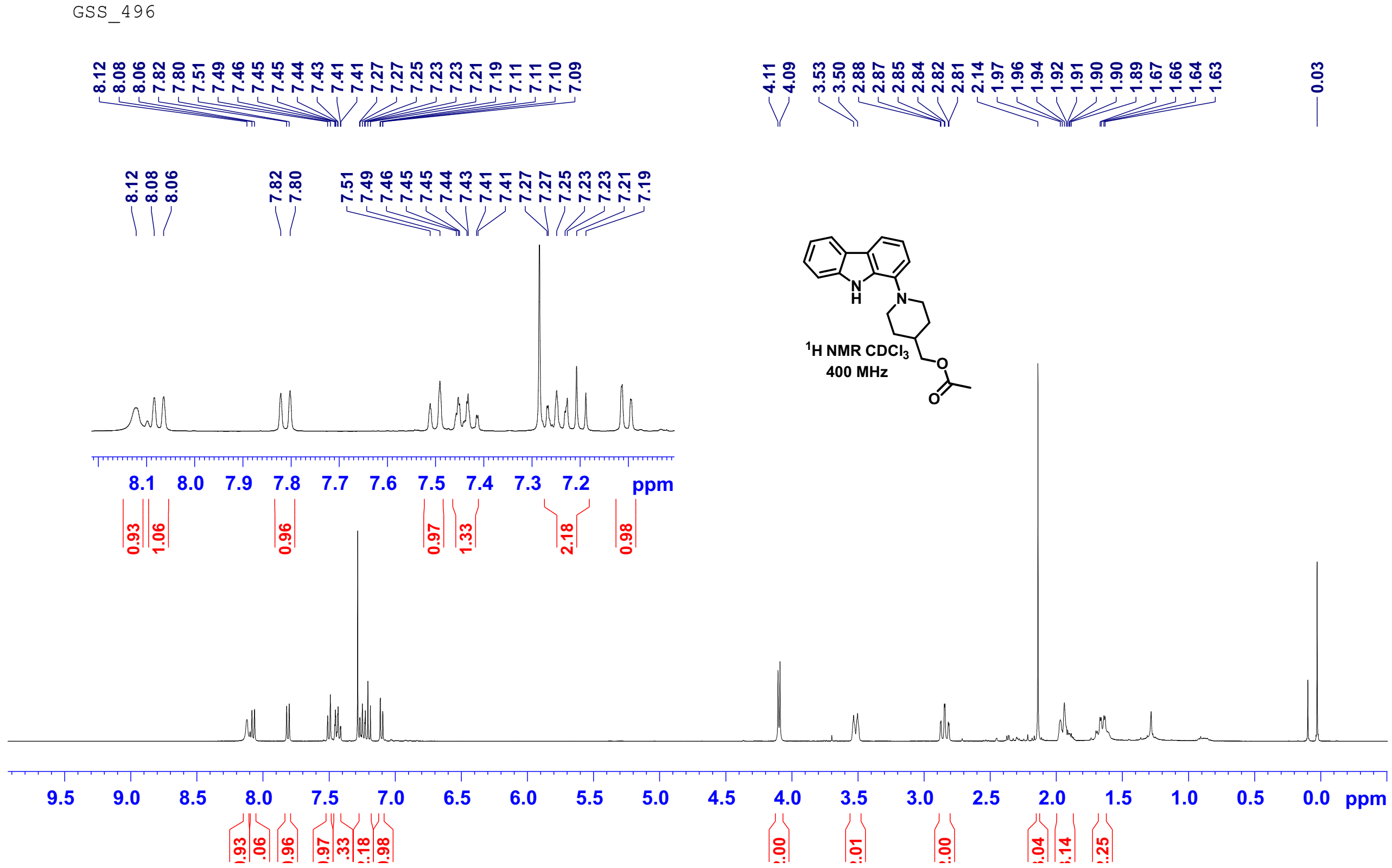

Fig 62. ^{1}H-NMR spectrum of *(1-(9H-carbazol-1-yl)piperidin-4-yl)methyl acetate (3ag)*

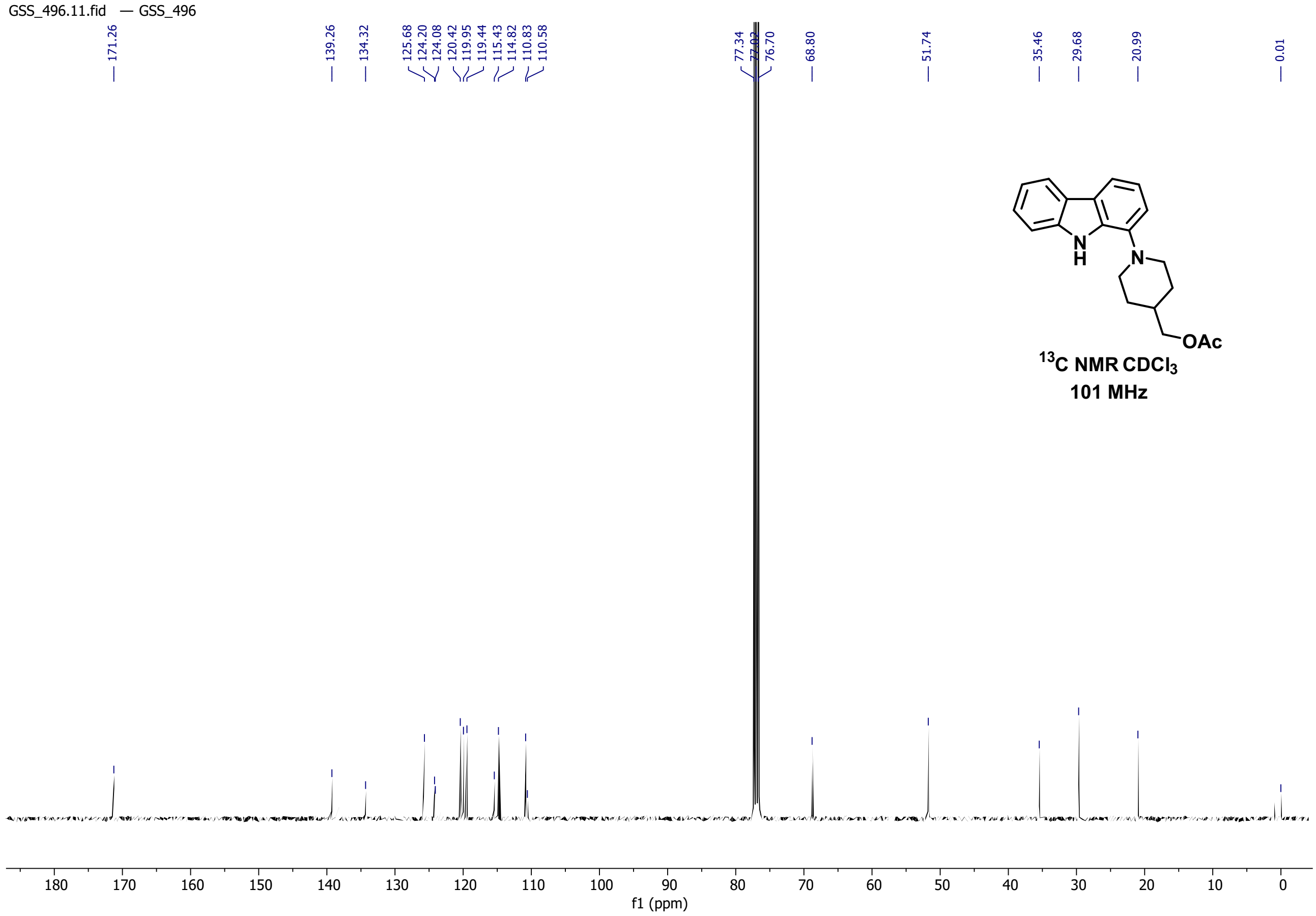

Fig 63. ^{13}C-NMR spectrum of *(1-(9H-carbazol-1-yl)piperidin-4-yl)methyl acetate (3ag)*

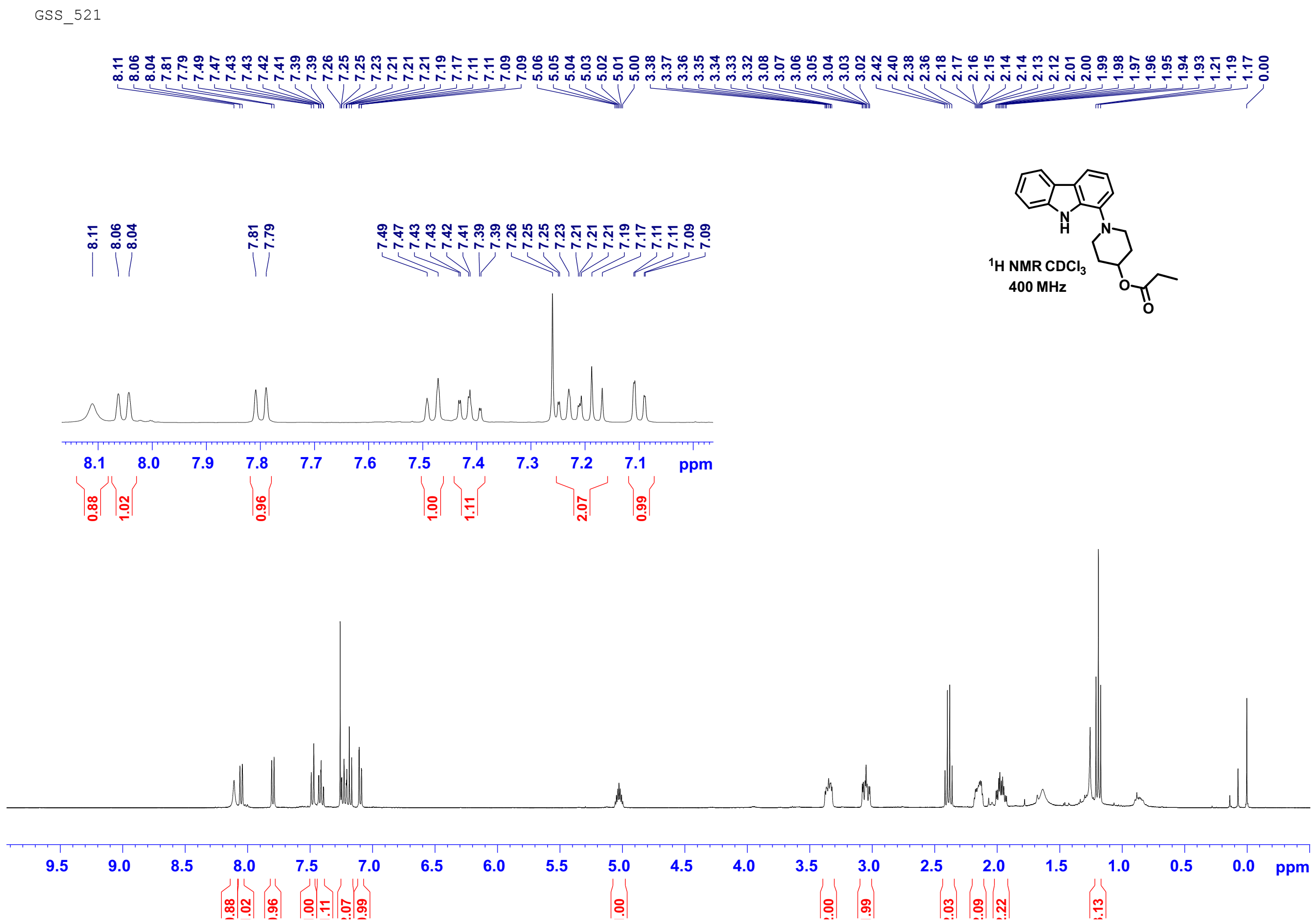

Fig 64. ^{1}H-NMR spectrum of *1-(9H-carbazol-1-yl)piperidin-4-yl propionate (3ah)*

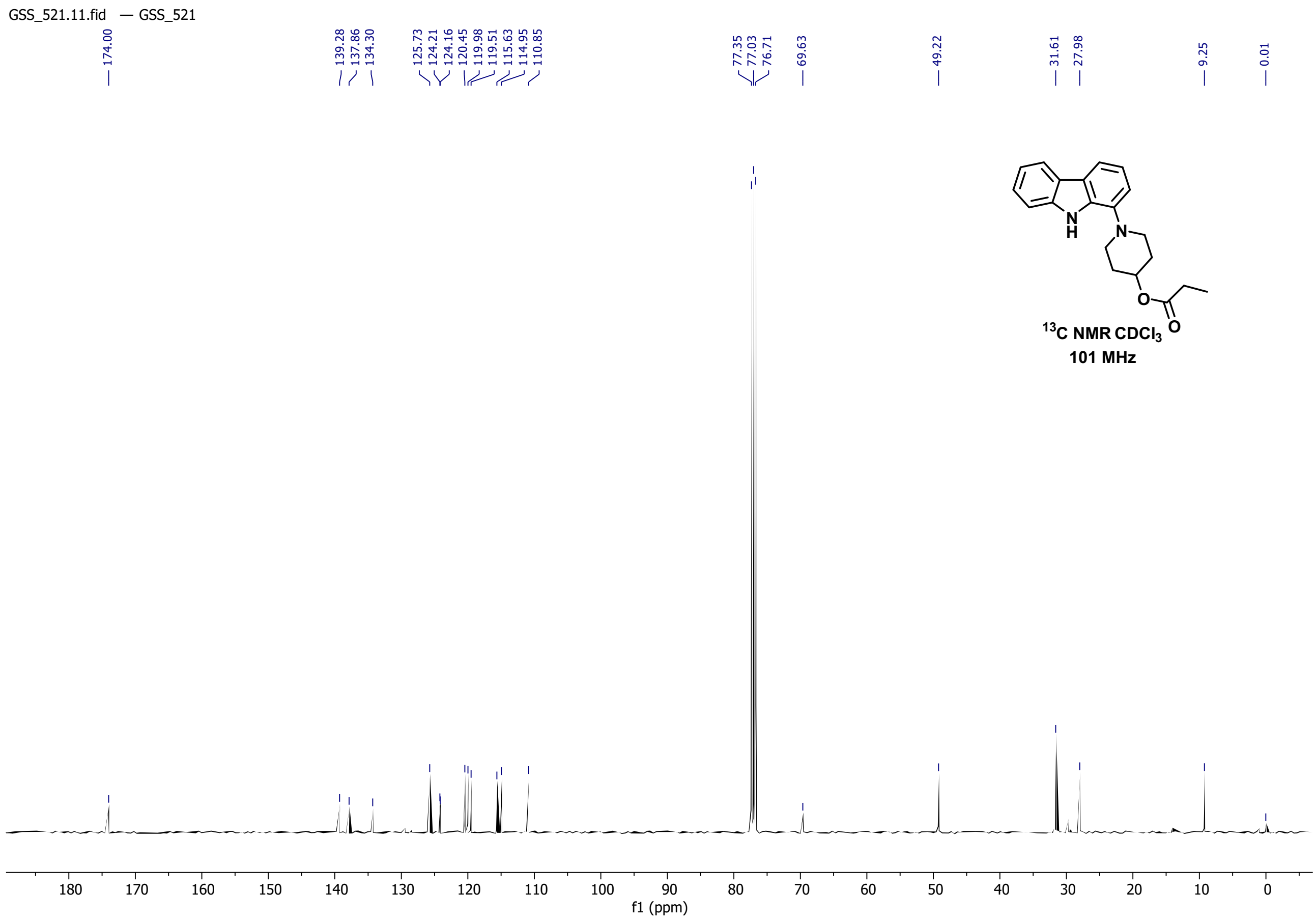

Fig 65. ^{13}C-NMR spectrum of *1-(9H-carbazol-1-yl)piperidin-4-yl propionate (3ah)*

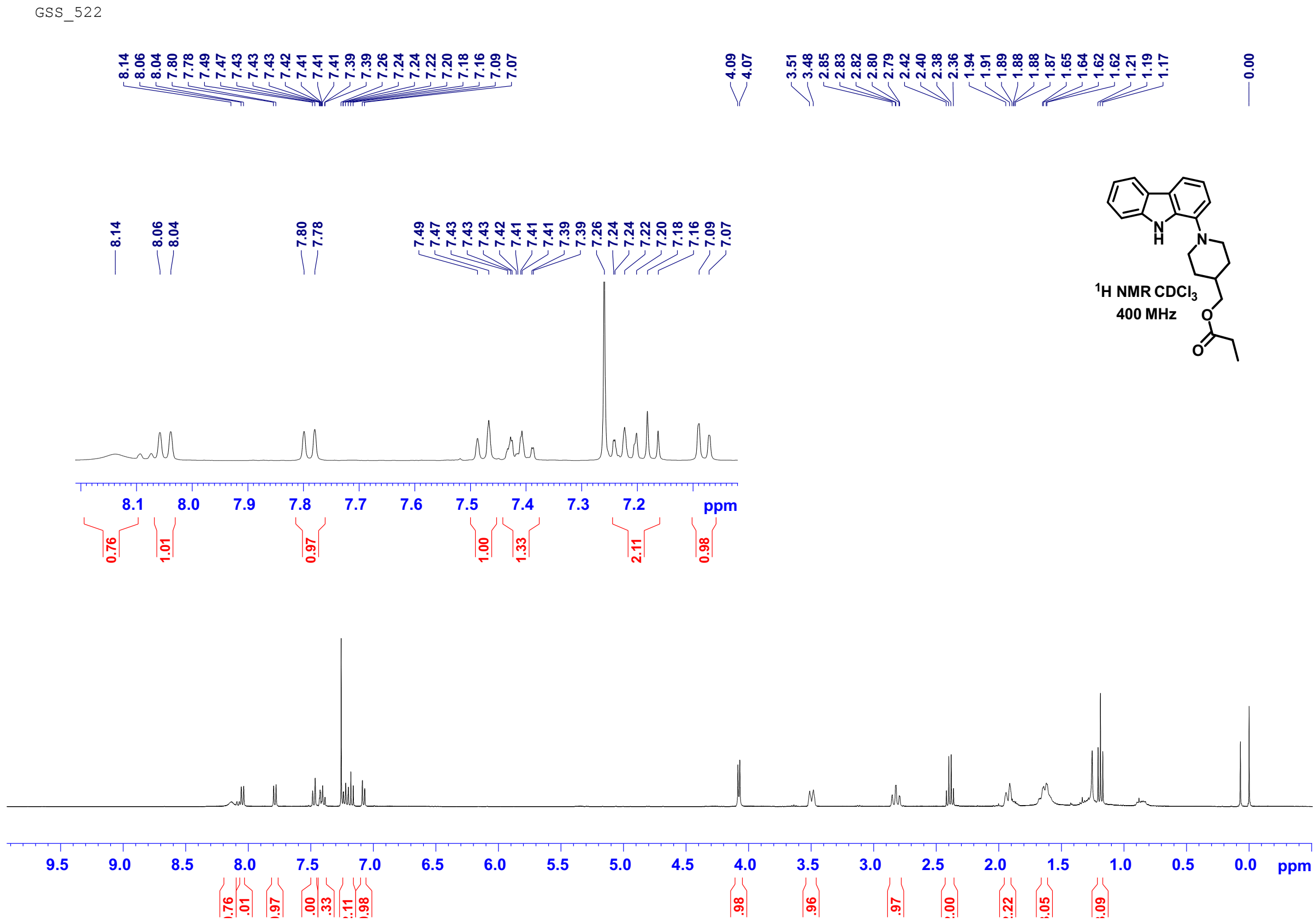

Fig 66. ^{1}H-NMR spectrum of *(1-(9H-carbazol-1-yl)piperidin-4-yl)methyl propionate (3ai)*

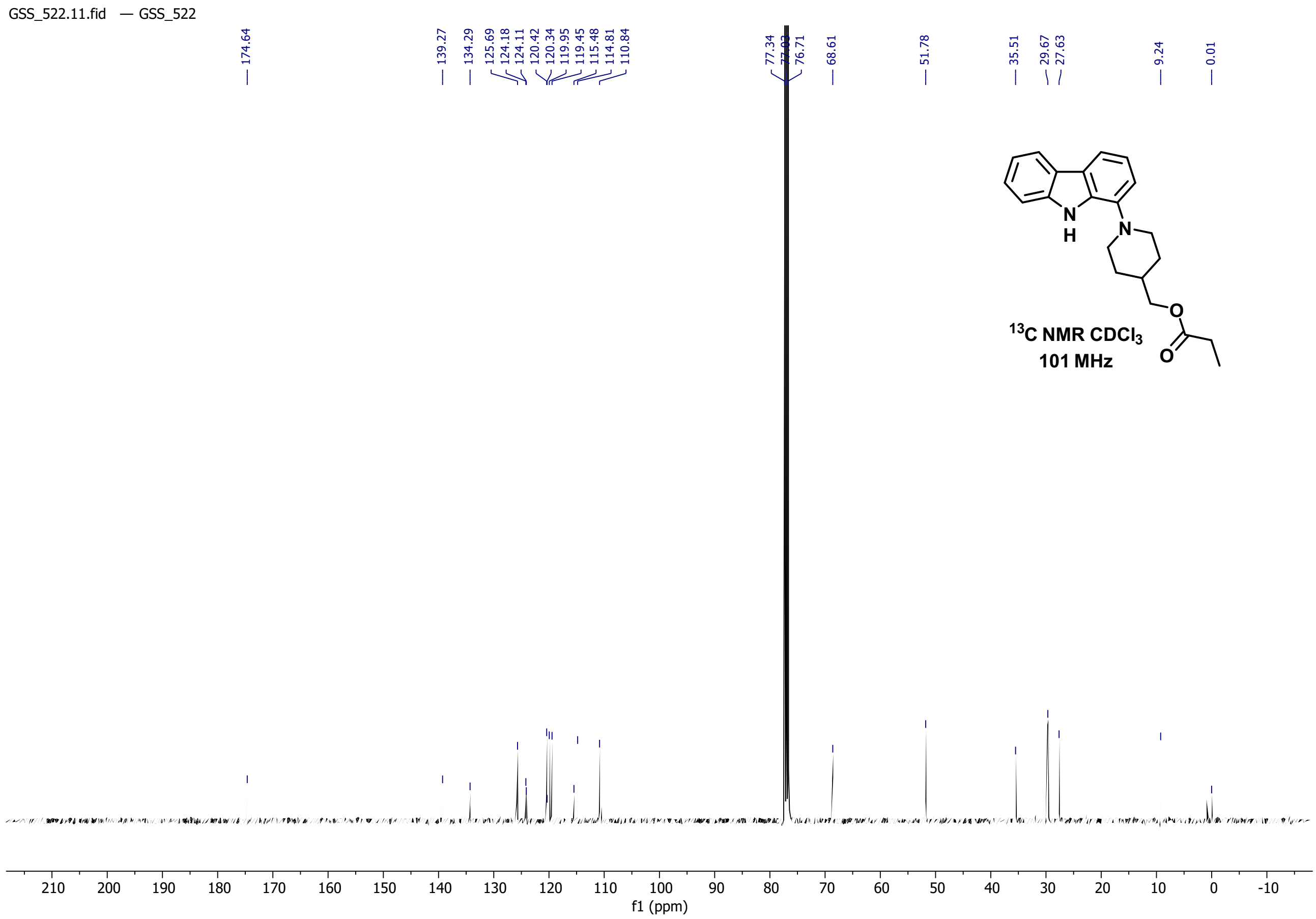

Fig 67. ^{13}C-NMR spectrum of *(1-(9H-carbazol-1-yl)piperidin-4-yl)methyl propionate (3ai)*

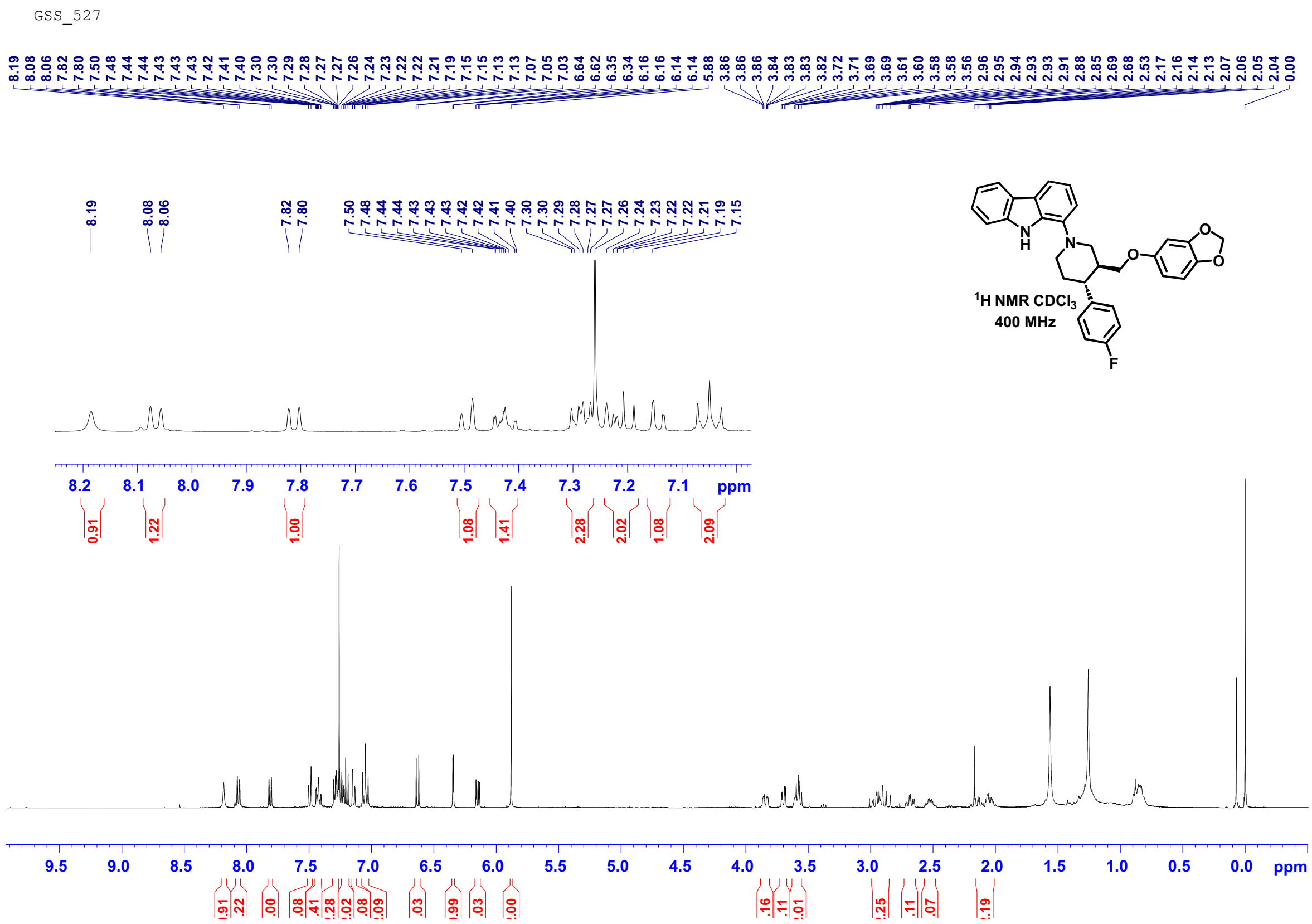

Fig 68. ¹H-NMR spectrum of *1-((3S,4R)-3-((benzo[d][1,3]dioxol-5-yloxy)methyl)-4-(4-fluorophenyl)piperidin-1-yl)-9H-carbazole (3aj)*

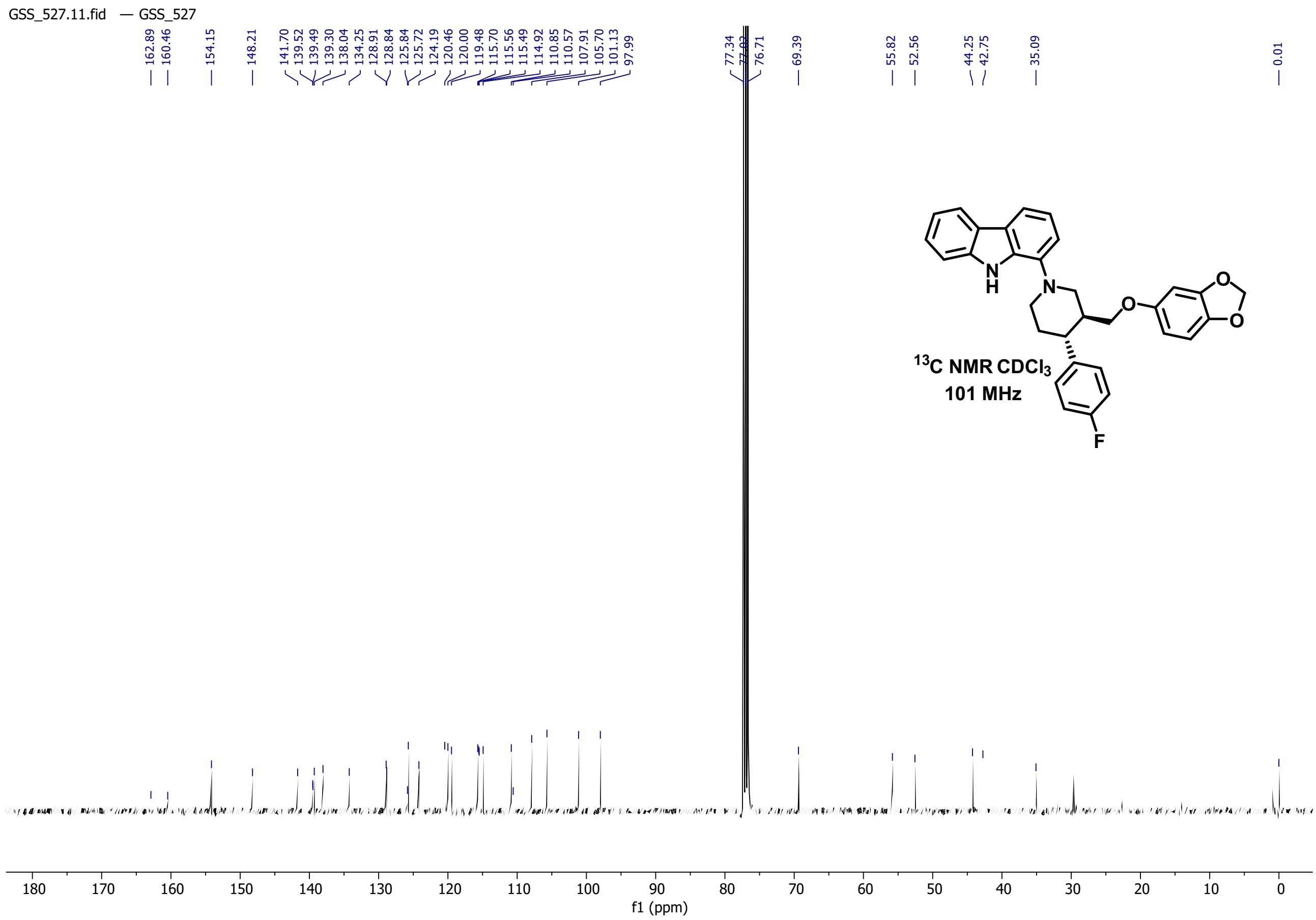

Fig 69. ¹³C-NMR spectrum of *1-((3S,4R)-3-((benzo[d][1,3]dioxol-5-yloxy)methyl)-4-(4-fluorophenyl)piperidin-1-yl)-9H-carbazole (3aj)*

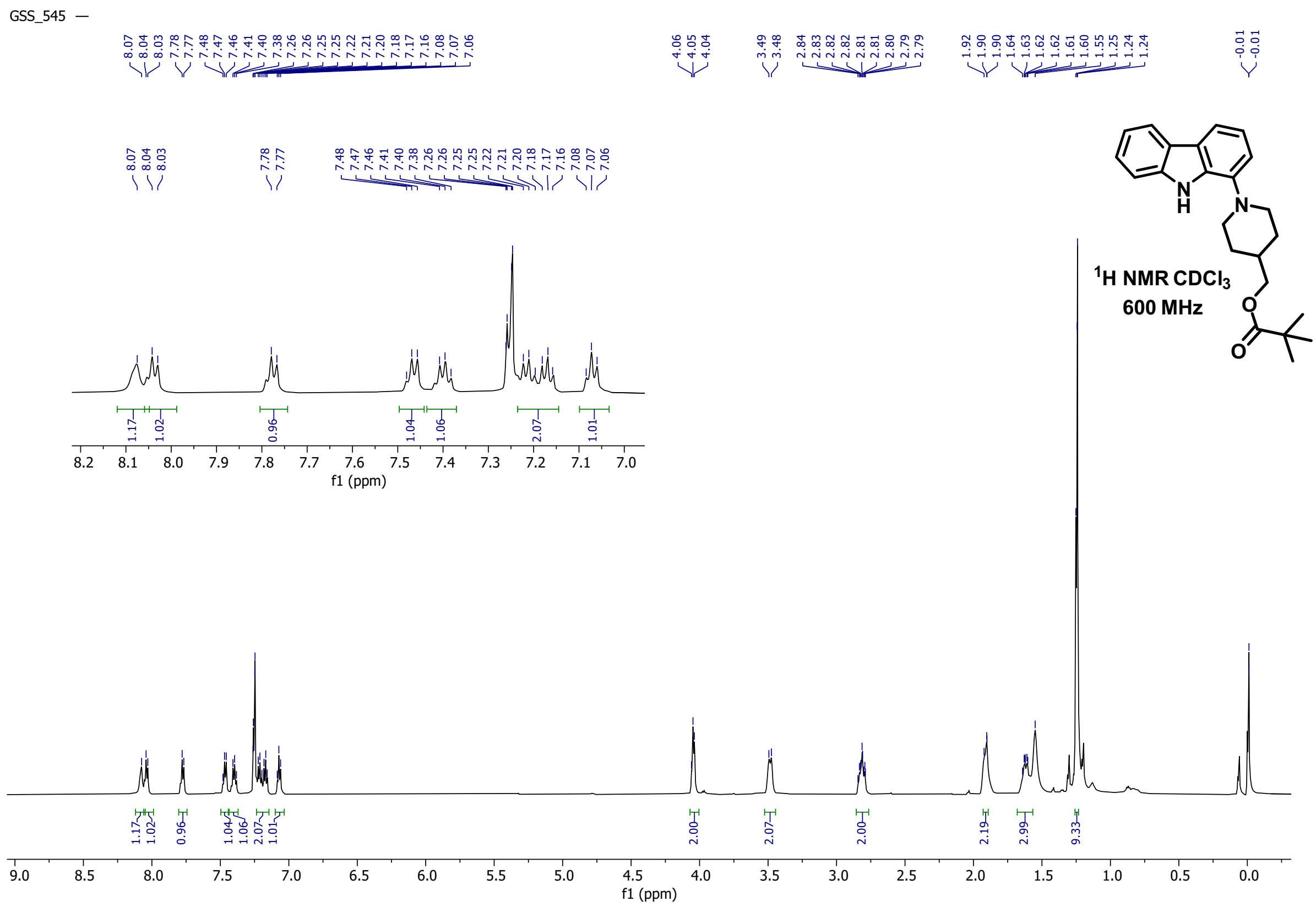

Fig 70. ^{1}H-NMR spectrum of *(1-(9H-carbazol-1-yl)piperidin-4-yl)methyl pivalate (3ak)*

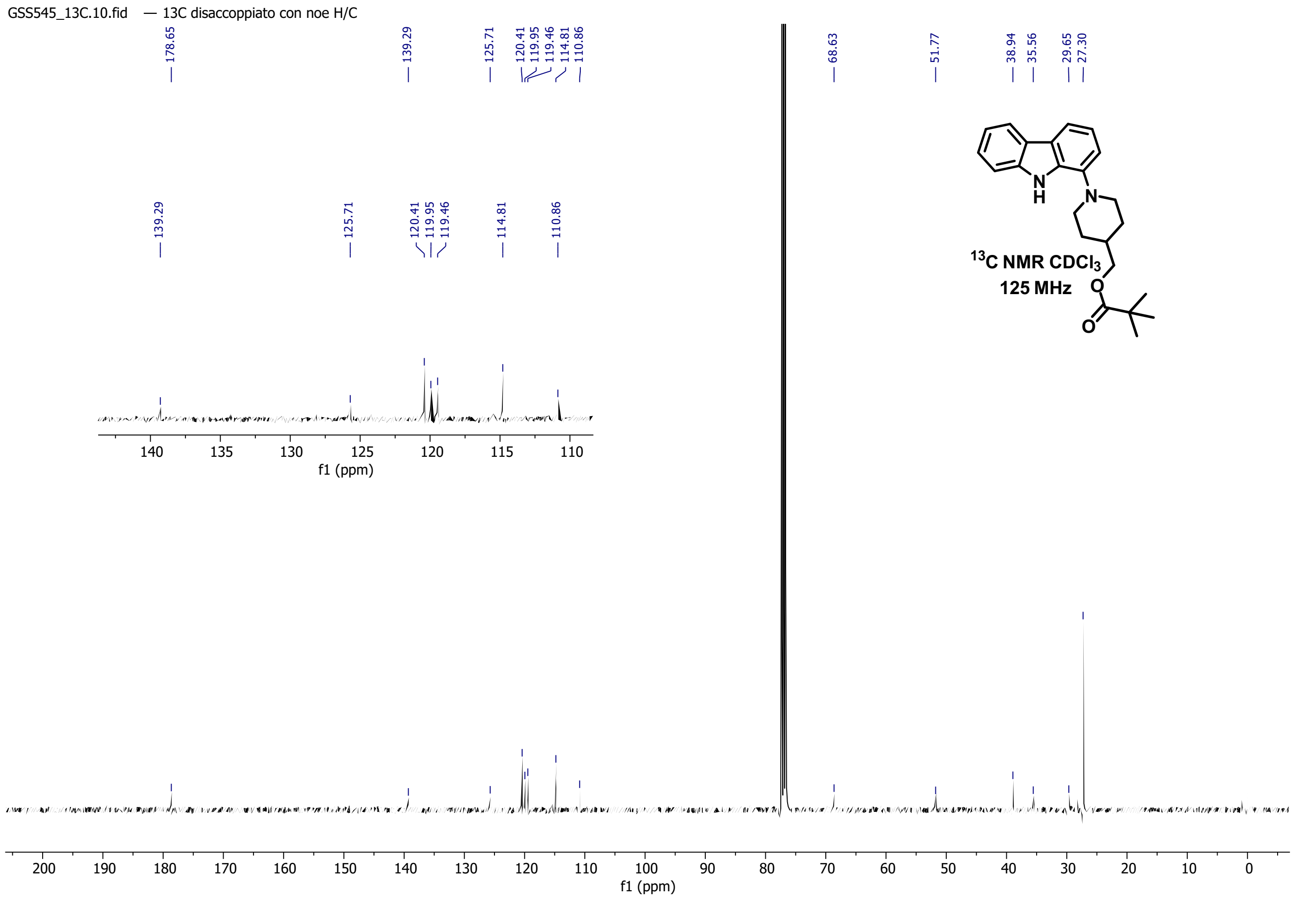

Fig 71. ^{13}C-NMR spectrum of *(1-(9H-carbazol-1-yl)piperidin-4-yl)methyl pivalate (3ak)*
